# Exploring the association between rheumatoid arthritis and non-small cell lung cancer risk: a transcriptomic and drug target-based analysis

**DOI:** 10.1186/s41065-025-00396-6

**Published:** 2025-02-27

**Authors:** Lyubo Wang, Yuxian Dong, Qingcheng Yang, Siyun Liu, Bencheng Wu, Dahang Zhang, Shuai Shen, Chenjun Xin, Zurui Liu, Qiuyang Wu, Guojian Huang, Lincan Duan

**Affiliations:** 1 Peking University Cancer Hospital Yunnan, Kunming, Yunnan, China; 2 The Third Affiliated Hospital of Kunming Medical University, Kunming(in Yunnan), China; 3 The First Affiliated Hospital of Kunming Medical University, Kunming, Yunnan, China; 4 First People’s Hospital of Yunnan Province, Kunming, Yunnan, China; 5 Kunming Dongchuan District People’s Hospital, Kunming, Yunnan, China; 6 Pu’er People’s Hospital, Pu’er, Yunnan, China

**Keywords:** Rheumatoid arthritis, Non-small cell lung cancer, Transcriptomics, Drug targets, Mendelian randomisation analysis

## Abstract

**Background:**

Non-small cell lung cancer (NSCLC) is a common subtype of lung cancer that has received considerable attention for its potential association with rheumatoid arthritis (RA). However, current understanding of the relationship between RA and NSCLC risk remains limited and in-depth studies of molecular mechanisms are lacking.

**Methods:**

We obtained transcriptomic data of NSCLC from the Gene Expression Omnibus (GEO) database and performed Gene Ontology (GO) and Kyoto Encyclopedia of Genes and Genomes (KEGG) analyses of differential genes. We then used Mendelian randomisation (MR) analysis to explore the causal relationship between RA and NSCLC, but the results showed no direct causal relationship between RA and NSCLC. In light of this finding, we shifted our research focus to investigate the effect of RA therapeutics on NSCLC risk. A drug-targeted MR analysis of drugs available for the treatment of RA was performed by searching for drugs that target NSCLC differential genes associated with RA.

**Results:**

We found that several of the drugs corresponding to NSCLC differential genes associated with RA are used to treat RA. By drug-targeted MR analysis of drugs, we found that some drugs do have an effect on the risk of developing NSCLC, increasing the risk of developing NSCLC.

**Conclusion:**

This study employed transcriptomic analysis and MR of drug targets to elucidate the potential correlation between RA and the risk of developing NSCLC. The identification of NSCLC differentially expressed genes associated with RA and their drug targets has provided new perspectives for an in-depth understanding of the pathogenesis of NSCLC. Furthermore, an additional immune infiltration analysis demonstrated that, in NSCLC tissues, the infiltration levels of specific immune cell subpopulations, including regulatory T cells (Tregs), activated natural killer cells (NK cells) and unpolarised macrophages (M0), exhibited notable differences. These findings emphasise the significant role that immune cell interactions between RA and NSCLC may play in disease progression. Furthermore, through the analysis of validation histology, we have further confirmed the potential role of differential genes associated with RA in the development of NSCLC. The expression levels of these genes demonstrated significant differences in NSCLC samples, providing a basis for possible future therapeutic targets and biomarkers.

**Supplementary Information:**

The online version contains supplementary material available at 10.1186/s41065-025-00396-6.

## Introduction

Non-small cell lung cancer (NSCLC) represents one of the most prevalent subtypes of lung cancer, with a global prevalence that rivals that of other cancers [1]. Despite extensive research into the aetiology of NSCLC, numerous questions remain unanswered, particularly with regard to its potential association with other diseases. In recent years, researchers have identified a number of potential associations between rheumatoid arthritis (RA) and NSCLC, which has prompted further investigation [2–4].

RA is a chronic autoimmune disease characterised by arthritis and systemic inflammation. Although RA primarily affects the joints, its systemic inflammation can also affect other organs, including the lungs [5]. It has also been shown that the incidence of lung cancer is significantly higher in patients with RA, but the exact mechanism is not yet clear [6].

In order to investigate the potential correlation between NSCLC and RA, we conducted a comprehensive analysis utilising NSCLC transcriptomics data from publicly accessible databases. By analysing differential genes and performing Gene Ontology (GO) and Kyoto Encyclopedia of Genes and Genomes (KEGG) functional enrichment analyses, we were able to identify biological processes and signalling pathways associated with RA in NSCLC. Nevertheless, despite these associations, no direct causal relationship between RA and NSCLC was identified through Mendelian randomisation (MR) analyses, which is also in accordance with the conclusions of previous investigators [7]. In light of these findings, our research focus shifted to investigating whether RA treatment affects the risk of developing NSCLC. Our findings revealed that numerous drugs targeting NSCLC differential genes associated with RA were employed for the treatment of RA, with tumour necrosis factor (TNF) inhibitors representing the primary pharmacological agents in this context. TNF is a multifunctional cytokine secreted mainly by macrophages, monocytes, neutrophils, CD4 + T cells and NK cells, and was originally named for its ability to induce necrosis in tumour cells [8]. Concurrently, we discovered that although there are cohort studies indicating that TNF inhibitors elevate the risk of cancer [9], there are also studies demonstrating that TNF inhibitors are not significantly associated with the risk of lung cancer development [10, 11]. Consequently, we conducted drug-target MR analyses for these drug targets and NSCLC to investigate the potential causal association between drug targets and NSCLC.

The objective of this study is to conduct a comprehensive investigation into the relationship between RA and NSCLC, with a particular focus on the molecular and cellular levels, in order to identify novel therapeutic targets for NSCLC. Furthermore, the comprehensive correlation analysis of RA drug targets and NSCLC offers more precise guidance for the management of RA patients in the future, with the aim of preventing the development of NSCLC. This analysis also provides insights into the pathogenesis of NSCLC, particularly with regard to the use of TNF inhibitors. It is our hope that this study will prove an invaluable reference point for future clinical practice and disease prevention.

## Materials and methods

### Data collection

Gene expression datasets and clinical phenotypic data matching the search terms ‘non-small cell lung cancer’, ‘Homo sapiens’ and ‘series’ were obtained through microarray dataset analysis.The sample sizes were selected in descending order, and the datasets that aligned with the study objectives were chosen in a sequential manner. All measured gene expression data and corresponding platform probe annotations are available for download from the Gene Expression Omnibus (GEO) database (https://www.ncbi.nlm.nih.gov/geo/). The dataset filtering criteria included a minimum of 20 samples, at least 10 cases and 10 controls, samples that had not been chemically treated or genetically modified, and the ability to locate the raw data or array gene expression profiling in the GEO database.

### Differential gene analysis

Batch correction and analysis of variance were conducted on 133 normal and 243 non-small cell lung cancer samples. The datasets GSE33532, GSE43458, and GSE75037 were read and pre-processed using the R software (version 4.3.1), and the datasets were merged after individual dataset standardisation. Principal component analysis (PCA) was conducted using the ‘sva’ and ‘ggpubr’ software packages to eliminate batch effects and facilitate visualisation. The ‘limma’ package was employed for classical Bayesian data analysis, with differentially expressed genes (DEGs) deemed significant at P < 0.05 and logFoldChange (LogFC) > 0.585. The ‘ggplot2’ and ‘pheatmap’ packages were used to generate volcano and heat maps of the identified differentially expressed genes (DEGs).

### GO and KEGG enrichment analysis

The functional annotation of co-expressed genes and KEGG pathway enrichment were conducted using the ‘clusterProfiler’ R software package to elucidate potential functional pathways and pathogenesis. The ‘clusterProfiler’ package is a universal enrichment tool for the interpretation of histological data with up-to-date gene annotations. The screening criteria for the study were set at *P* < 0.05.

### Data screening

In this study, data pertaining to exposure and outcome in relation to RA and NSCLC were obtained from the IEU Open GWAS database (https://gwas.mrcieu.ac.uk/). The GWAS ID for RA was EBI-a-GCST90018910, while the GWAS ID for NSCLC was FINN-b-C3_LUNG_NONSMALL_EXALLC. To control for false positives as much as possible, ensure sufficient power and minimise potential bias, we used the R package TwoSampleMR, which identifies highly correlated SNPs (*p* < 5e-08) as instrumental variables and clumps them using a pairwise linkage disequilibrium (LD) threshold of r2 < 0.001 and a window of 10,000 kb. To examine the causal associations of the three target genes with NSCLC, eqtl-a-ENSG00000125538, eqtl-a-ENSG00000232810 and eqtl-a-ENSG00000110944 were used as exposure data, and the resulting data were consistent. For MR analysis of the expression Quantitative Trait Locus (eQTL) data, we refer to previous studies [12, 13], genetic instruments were further filtered by the strength of association (*P* < 5e − 8) and clumped at a pairwise LD threshold of r2 < 0.3 and a window of 100 kb using the TwoSampleMR R package. F statistics were calculated for each SNP, and SNPs with F statistics < 10 were excluded to avoid weak instrument bias [14]. Further details on the data can be found in the IEU Open GWAS database.

### MR analysis

MR analyses were conducted using the ‘TwoSampleMR’ software package. The inverse variance weighting (IVW) method was employed to investigate the association between RA and NSCLC, with the MR-Egger, simple mode, weighted median, and weighted mode methods serving as references. In light of the aforementioned findings, a MR analysis was conducted to ascertain the causal relationship between drug target genes and NSCLC. Furthermore, a horizontal multiplicity test, heterogeneity test, and sensitivity analysis were conducted on the MR results to evaluate the robustness and reliability of the findings [15]. The visualisation of the data employed scatter plots, forest plots and funnel plots.

### Construction of ceRNA networks

In order to predict potential microRNAs (miRNAs) that can bind to mRNAs, we utilised the following online tools: Targetscan (http://www.targetscan.org/) (Li et al., 2017), miRDB (http://mirdb.org) (Chandrashekar et al., 2017), miRanda (http://www.microrna.org/) (Doron et al., 2008) and miRWalk (http://mirwalk.umm.uni-heidelberg.de/) (Sticht et al., In 2018, we employed the tool spongeScan (http://spongescan.rc.ufl.edu) (Furió-Tarí et al., 2016) to analyse the expression and prognosis of miRNAs and long non-coding RNAs (lncRNAs). Subsequently, we employed the Cytoscape software (version 3.10.1) for visualisation purposes.

### Immune cell analysis

We employed the CIBERSORT algorithm to evaluate the degree of immune cell infiltration in NSCLC. CIBERSORT facilitates immune cell analysis through the back-convolution of gene expression microarray data, offering a precise assessment of a defined subset of 22 closely related immune cell types by leveraging the genes characteristic of a vast number of tumour samples [16].

### Validation group analysis of variance

We performed data pre-processing and standardisation of The Cancer Genome Atlas (TCGA) lung cancer data using the same methodology as previously described. The aim of this process was to check whether there were significant differences in the expression of the relevant genes between the control and experimental groups, and to further analyse these results in comparison with the results of our MR analysis to increase the reliability and consistency of the conclusions.

### Survival analysis

To further explore the clinical significance of the genes of interest, we performed a survival analysis of the correlation between the expression levels of these genes and patient prognosis using clinical data from the TCGA database. Given the validity of the log-rank test in comparing differences in survival data between two groups, we chose this statistical method to assess the association between gene expression levels and patient survival time.

## Results

### Overview of the three GEO datasets

**Fig. 1:**
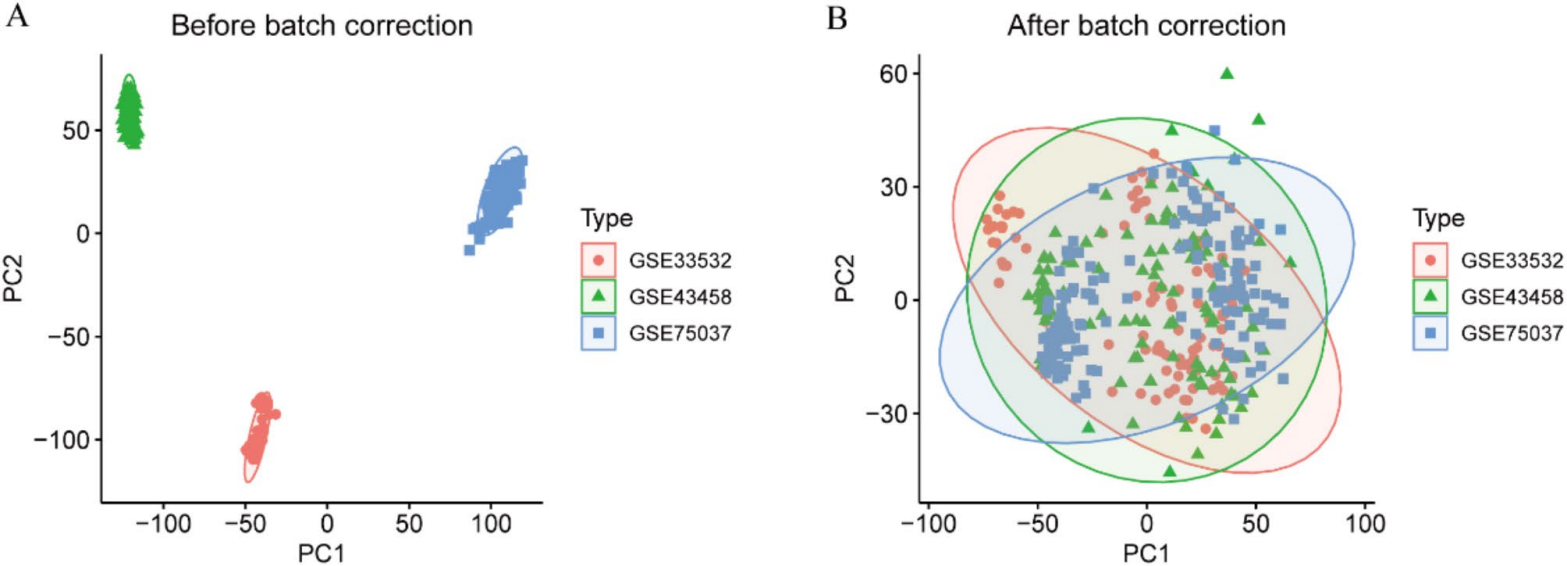
(**A**) Before batch correction. (**B**) After batch correction

In this study, three NSCLC microarray datasets were obtained from the GEO database and constituted the experimental groups. All three datasets were derived from lung tissues and the experimental type was array. The datasets comprised a total of 243 NSCLC tissues and 133 normal lung tissues. Further details of the three datasets are provided in Table 1. The expression values of each gene in the respective datasets were corrected and merged using R (version 4.3.1), and batch effects were eliminated by principal component analysis (PCA). As illustrated in Fig. 1, the three NSCLC gene datasets exhibited discernible batch effects. However, following PCA analysis, all samples within the datasets demonstrated satisfactory homogeneity.

**Table 1:** Characteristics of the three GEO datasets

GSE ID	Samples	Tissues	Platform	Experiment type Last update date
GSE33532	80 cases and 20 controls	lung tissue	GPL570	Nov 08, 2011
GSE43458	80 cases and 30 controls	lung tissue	GPL6244	Jan 11, 2013
GSE75037	83 cases and 83 controls	lung tissue	GPL6884	Nov 16, 2015

### Identification of differential genes

**Fig. 2:**
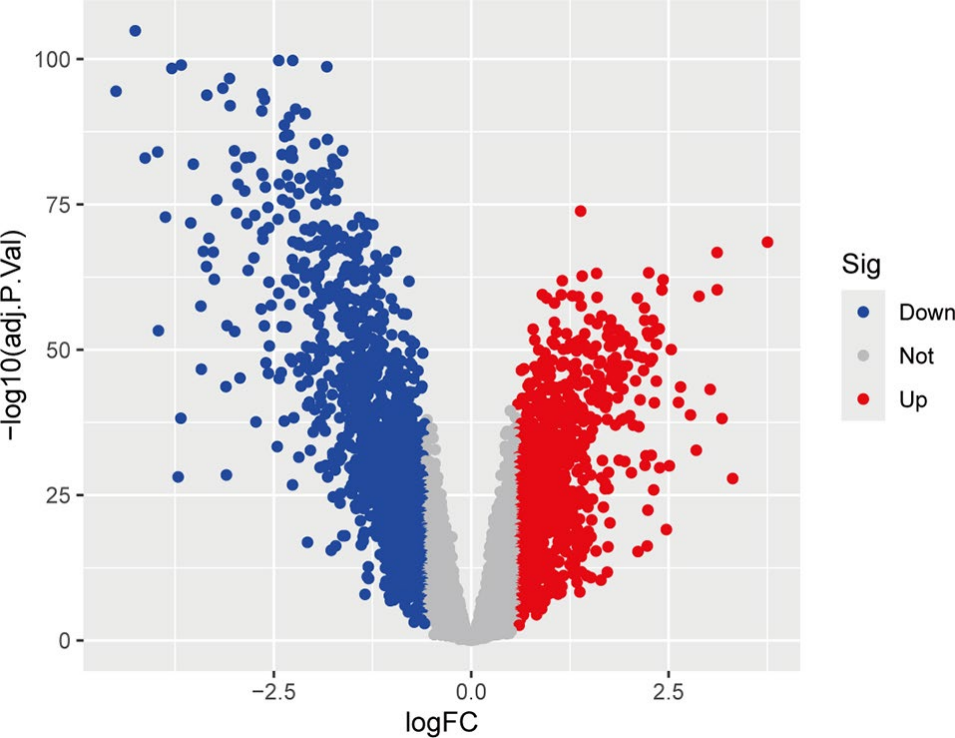
Differential gene volcano map

**Fig. 3:**
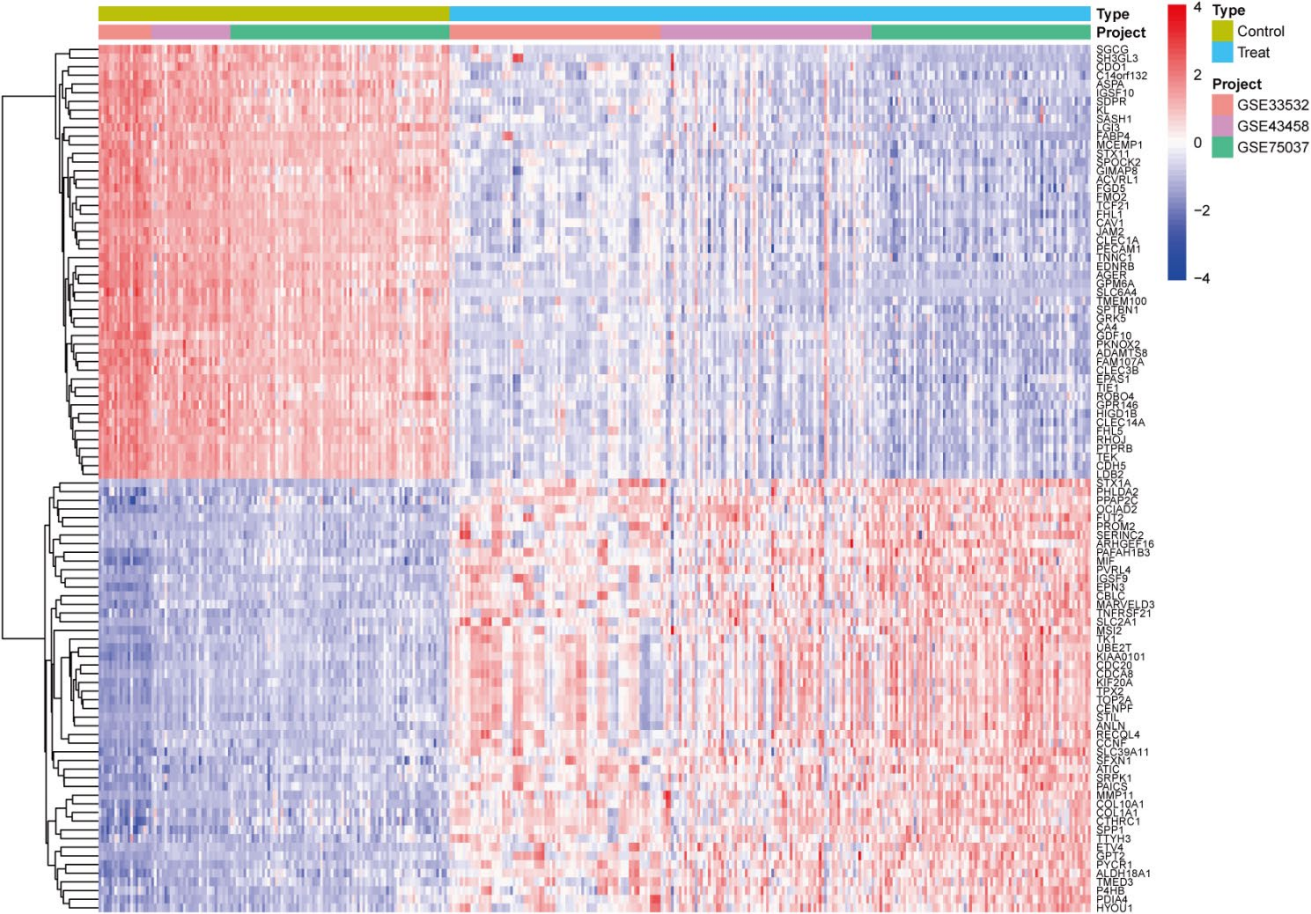
Heatmap of differential gene expression

A differential analysis was conducted on the dataset, with batch effects removed in accordance with the established P-values and logFC values. In conclusion, 1,092 up-regulated DEGs and 1,420 down-regulated DEGs were identified. Further details on these significantly differentially expressed genes can be found in Supplementary Table S1. A volcano plot of the integrated GEO dataset is presented in Figure 2. The heatmap of DEGs in Figure 3 illustrates the top 50 up-regulated and the top 50 down-regulated genes.

### GO/KEGG enrichment analysis

**Fig. 4:**
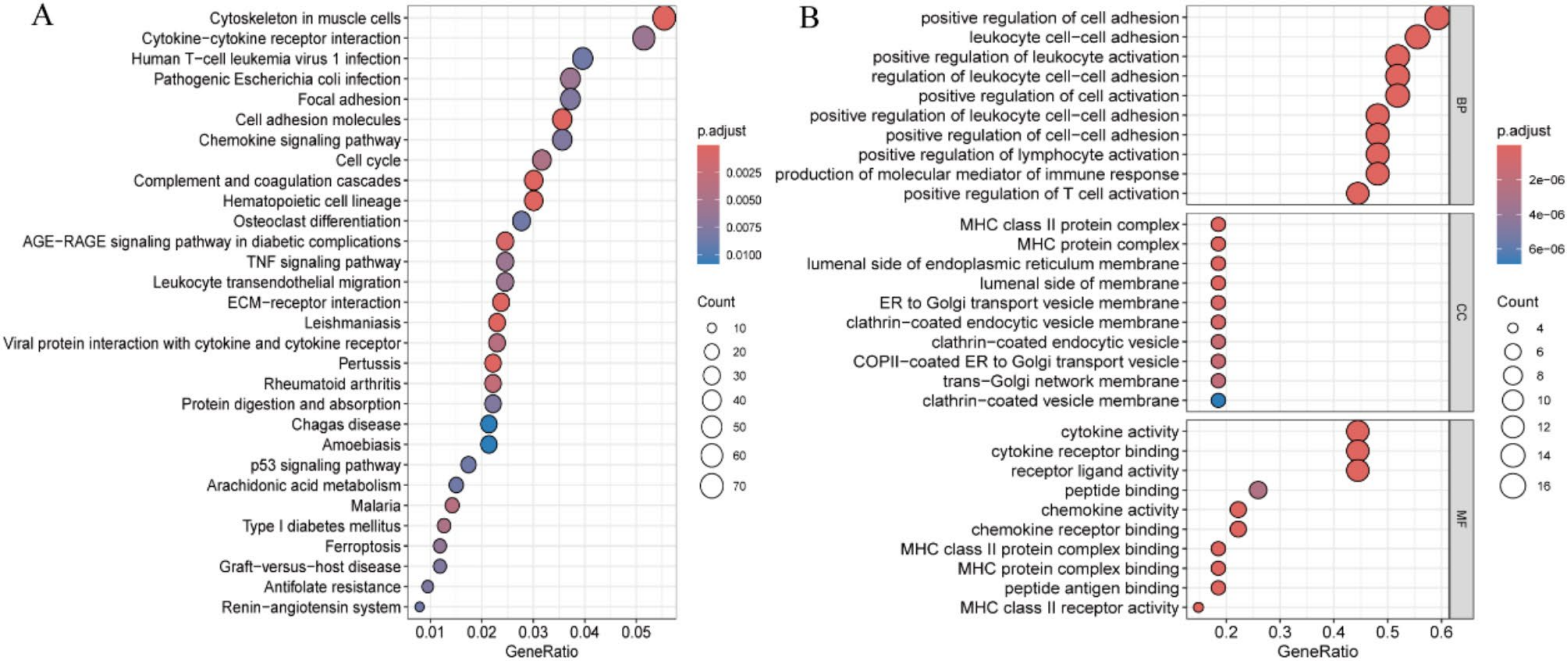
(**A**) KEGG enrichment analysis of differential genes. (**B**) GO enrichment analysis of RA-related differential genes in NSCLC

The potential roles of the differential genes were further explored through KEGG analysis (Fig. 4A). The KEGG results indicated that the differential genes mainly affected the functions of cytoskeleton, cytokine-cytokine receptor interactions, human T-cell leukaemia virus 1 infection, chemokine signalling pathway, cell cycle, and TNF signalling pathway in muscle cells. Secondly, 28 differential genes associated with RA were identified.

To gain further insight into the functions of the differential genes associated with RA, we conducted a GO enrichment analysis of 28 differential genes (Fig. 4B). This analysis revealed that the associated genes predominantly influenced the positive regulation of T cell and lymphocyte activation, the production of molecular mediators of immune response, the positive regulation of intercellular adhesion, as well as the functions of receptor-ligand activity and cytokine activity. In NSCLC and RA, this may result in aberrant immune responses, inflammation, and disease progression.In addition, we used KEGG enrichment analysis methods to map the target genes to the enriched pathways (Supplementary Figure S1). This analysis allowed us to gain more insight into the effects of these gene-drug interactions on specific pathways. Among the enriched pathways, we found that the IL-17 signalling pathway and the TNF signalling pathway were particularly associated with lung cancer. This suggests that these pathways may play an important role in the development and progression of lung cancer and that gene-drug interactions may influence the biological behaviour of lung cancer by modulating these pathways.

### Drug-gene interactions

The present study reveals the existence of potential interactions between a range of drugs and genes that are associated with non-small cell lung cancer (NSCLC). A target drug analysis of NSCLC differential genes associated with rheumatoid arthritis (RA) via the DGIdb database (https://dgidb.genome.wustl.edu/) revealed a variety of drugs used for the treatment of RA that interact with genes associated with NSCLC. In particular, we observed that genes such as IL1B, IL23A, and TNF interacted with drugs such as RILONACEPT, CANAKINUMAB, ADALIMUMAB, GOLIMUMAB, ETANERCEPT, CERTOLIZUMAB PEGOL, INFLIXIMAB, and USTEKINUMAB, as detailed in Table 2.

**Table 2:** Gene-drug interactions table

Gene	Drug	Match-type	Interaction
IL1B	RILONACEPT	Definite	inhibitor
	CANAKINUMAB	Definite	inhibitor
TNF	ADALIMUMAB	Definite	inhibitor
	GOLIMUMAB	Definite	inhibitor
	ETANERCEPT	Definite	inhibitor
	CERTOLIZUMAB PEGOL	Definite	inhibitor
IL23A	INFLIXIMAB	Definite	inhibitor
	USTEKINUMAB	Definite	inhibitor

### MR analysis

To further explore the relationship between NSCLC and RA, we used MR analysis to see the causal association between RA and NSCLC (visualisation results are shown in Supplementary Figure S2). However, the results showed no significant association between the two, which is consistent with previous studies [7].

**Fig. 5:**
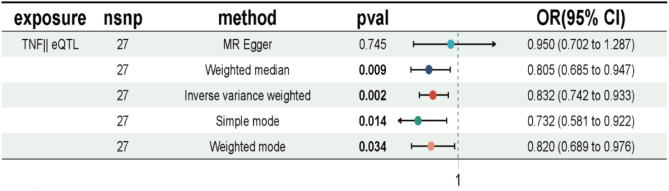
MR analysis between eQTL for TNF and NSCLC

In light of the aforementioned considerations, we elected to modify our study to investigate whether the treatment of RA would exert a differential effect on NSCLC. In the drug-gene interaction table of 28 differential genes (see Supplementary Table S2 for details), the three genes that are RA therapeutic drugs and would have differential effects on target genes are IL1B, IL23A, and TNF. A drug-targeted Mendelian randomisation analysis was performed between the three genes and NSCLC [17]. Although the number of SNPs for IL1B and IL23A was insufficient to support MR analysis, the p-values for TNF were less than 0.05, with the exception of the MR Egger method. Furthermore, the differences were statistically significant. The results of the MR analysis for TNF are presented in forest plots (Fig. 5) The inverse variance weighted method yielded a drug OR (Odds Ratio) value of 1.202 (CI: 1.072 to 1.348), while the remaining methods produced OR values exceeding 1, with the exception of the MR Egger p-value, which was greater than 0.05. These findings indicate that the utilisation of TNF inhibitors, which are employed for the treatment of RA, may potentially elevate the risk of developing NSCLC in patients.

Meanwhile, we calculated the Q-statistics for MR Egger and the inverse variance weighting method, which resulted in p-values greater than 0.05 (0.5628 and 0.5692 respectively), indicating that the heterogeneity was not significant. This suggests that the consistency between the selected instrumental variables is good and there is no significant heterogeneity problem. In addition, we used the MR Egger method to assess horizontal multicollinearity.The MR Egger method determines the presence of horizontal multicollinearity by assessing whether or not the intercept is significantly different from zero. In our analyses, the intercept was − 0.0246, the standard error was 0.0264, and the p-value was 0.3608.Since the p-value was greater than 0.05, we did not find significant horizontal multicollinearity. This suggests that the selected instrumental variables affect the outcome (NSCLC) mainly through exposure (TNF eQTL) and not through other unobserved pathways.

**Fig. 6:**
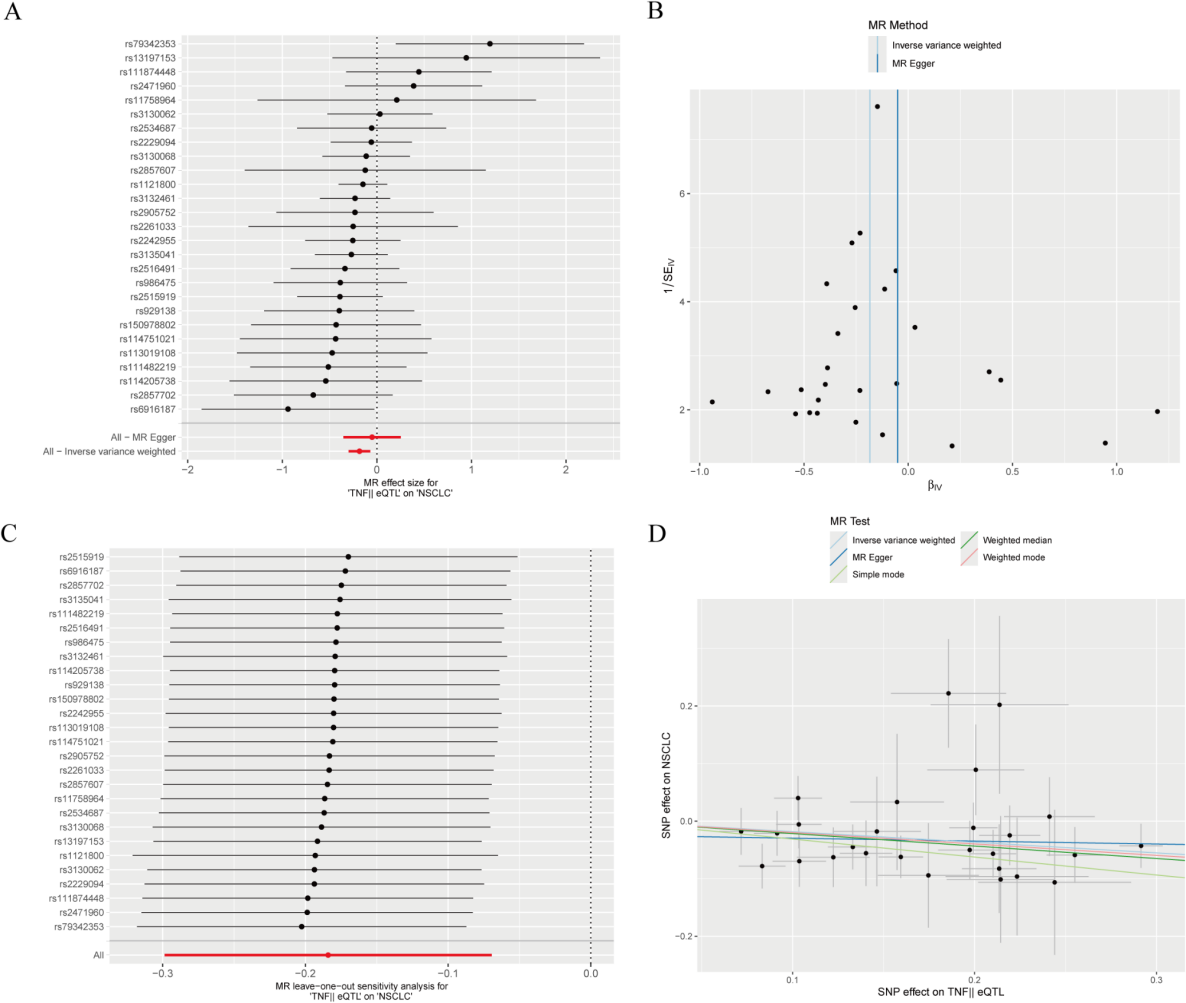
Mendelian randomization analysis suggests a causal relationship between TNF and non-small cell lung cancer: (**A**) the effect size for each SNP; (**B**) the potential for bias; (**C**) leave-one-out sensitivity analysis; (**D**) a negative correlation between TNF and non-small cell lung cancer

Furthermore, we performed sensitivity analyses using the MR-PRESSO method to assess the impact of horizontal pleiotropy on causal inference.MR-PRESSO identifies potential horizontal pleiotropy by detecting the residual sum of squares (RSS). In our analysis, the observed residual sum of squares (RSSobs) was 25.184, corresponding to a p-value of 0.636.As the p-value was greater than 0.05, we did not find significant horizontal pleiotropy. This further supports that the selected instrumental variables affect the outcome (NSCLC) mainly through exposure (TNF eQTL) and not through other unobserved pathways.All of the above results show that the analyses are robust. In addition, the data were visualised using scatter plots, forest plots, funnel plots and exclusion sensitivity analysis plots (see Fig. 6).

### CeRNA network construction

The four databases, miRanda, miRDB, miRWalk and TargetScan, were employed to identify common miRNAs, and subsequently, the spongeScan database was utilised to ascertain related lncRNAs. This resulted in the generation of a ceRNA network table comprising 22 mRNAs, 54 miRNAs and 211 lncRNAs (for further details, please refer to Supplementary Table S3). The majority of mRNAs and a subset of miRNAs have been corroborated by empirical evidence. For example, ATP6V1C2 has been demonstrated to promote or inhibit tumour proliferation in oesophageal cancer [18], colorectal cancer [19] and renal clear cell carcinoma [20]. It has been demonstrated that inhibition of miRNA-92b-5p exacerbates LPS-induced chondrocyte damage [21]. Furthermore, miR-125b-2-3p has been identified as a chemotherapeutic prognostic factor in advanced colorectal cancer [22]. Furthermore, the biological functions and mechanisms of miRNA-7-5p, miRNA-31-5p, miRNA-199a-5p, miRNA-181a-5p, miRNA-143-3p and miRNA-141-3p in different diseases have been documented, although there is a paucity of reports on lncRNA studies.

Meanwhile, miRNA-939-5p has been demonstrated to be a valuable diagnostic and prognostic indicator in cancer by regulating LIMK2 expression [23]. Additionally, in triple-negative breast cancer, miRNA-939-5p has been observed to interact with lncRNAs, influencing disease progression [24]. Furthermore, in pancreatic cancer, miRNA-939-5p has been demonstrated to promote tumour migration and invasion by targeting ARHGAP4 [25]. Nevertheless, its correlation with TNF and lncRNA in NSCLC, and its function in RA, have yet to be documented in scientific literature. Further research may reveal its potential as a new therapeutic target in rheumatoid arthritis and a possible new predictive target in non-small cell lung cancer.

In conclusion, our study identified 54 miRNAs and 211 lncRNAs that are associated with RA and NSCLC. These findings suggest that these molecules may represent novel targets for the treatment or prevention of RA and NSCLC. Visualisations were performed using Cytoscape (see Supplementary Figure S3 for details).

### Assessment of immune infiltration in non-small cell lung cancer

**Fig. 7:**
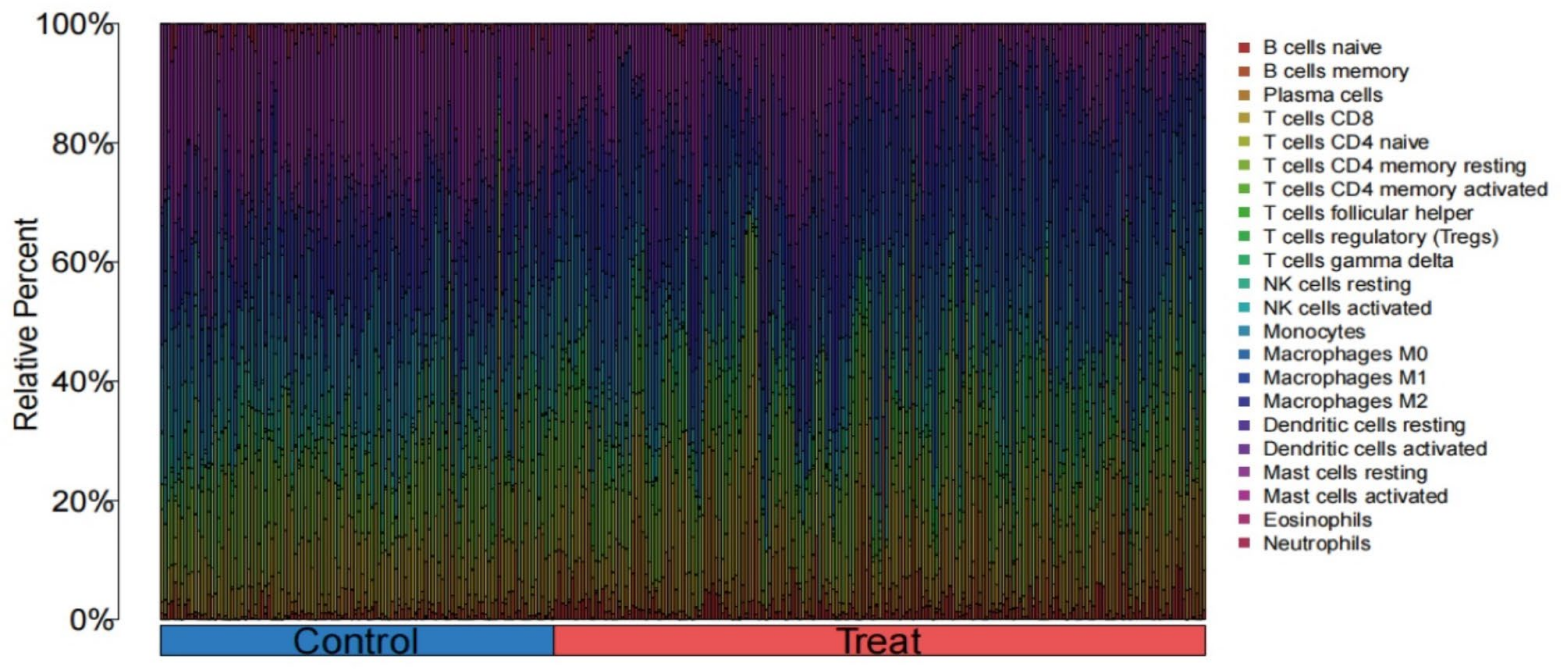
Stacked histogram of the proportions of immune cell between the NSCLC group and the control group

**Fig. 8:**
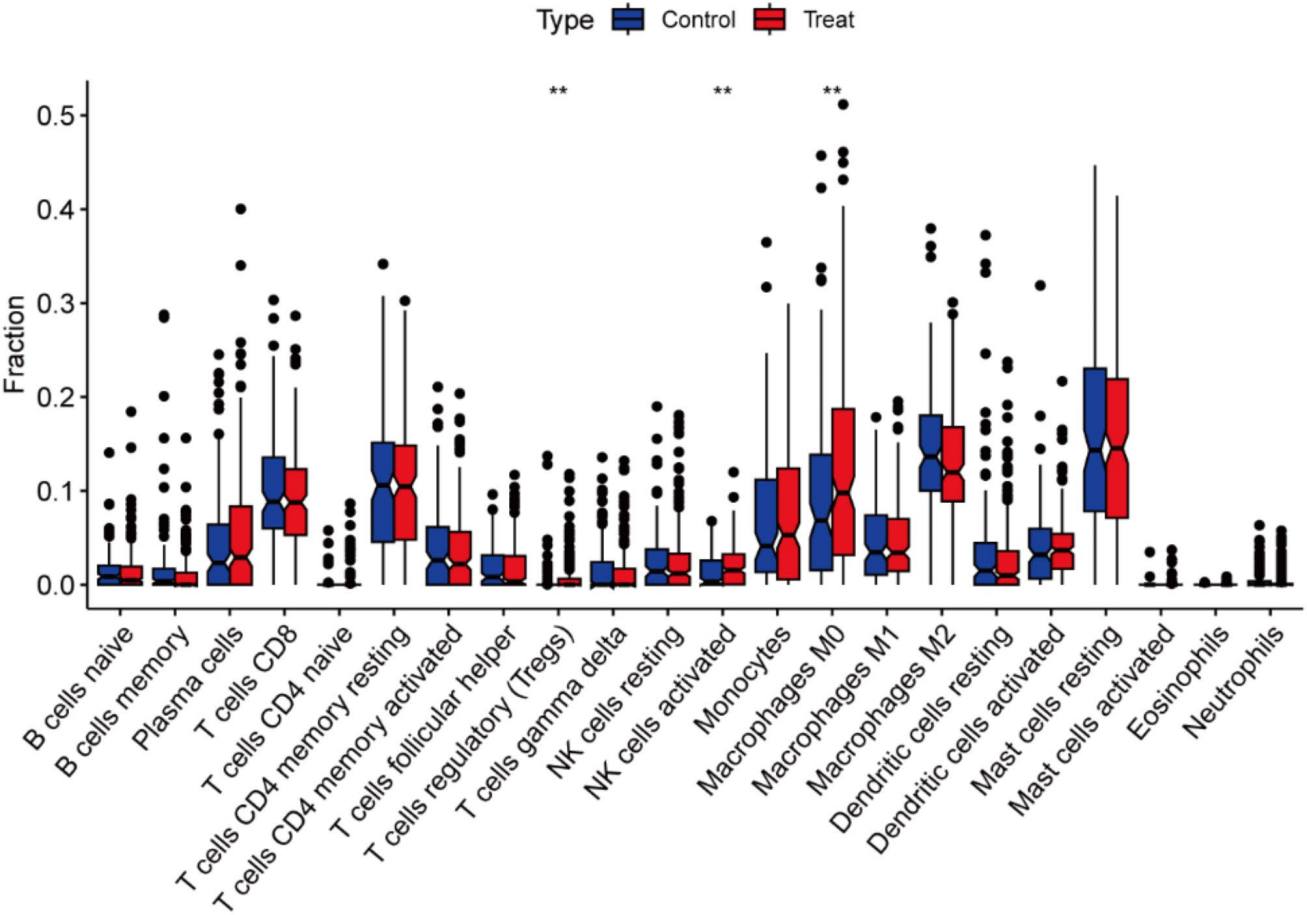
Box plot showing the comparison of 22 types of immune cells between the NSCLC group and control group. **p < 0.01

NSCLC and RA are closely associated with inflammatory and immune processes. In order to infer the characteristics of immune cells and to explore the correlation between genes associated with RA and immune cell infiltration in NSCLC, the CIBERSORT algorithm was employed. Figure 7 illustrates the proportion of immune cells in each of the 22 samples. Significant differences were identified in specific immune cell subtypes (T cells regulatory (Tregs), NK cells activated, and macrophages M0) between the NSCLC and control groups. In particular, there were significantly higher proportions of T cell regulatory cells, activated natural killer cells, and unpolarised macrophages in the NSCLC patient group (Fig. 8).

**Fig. 9:**
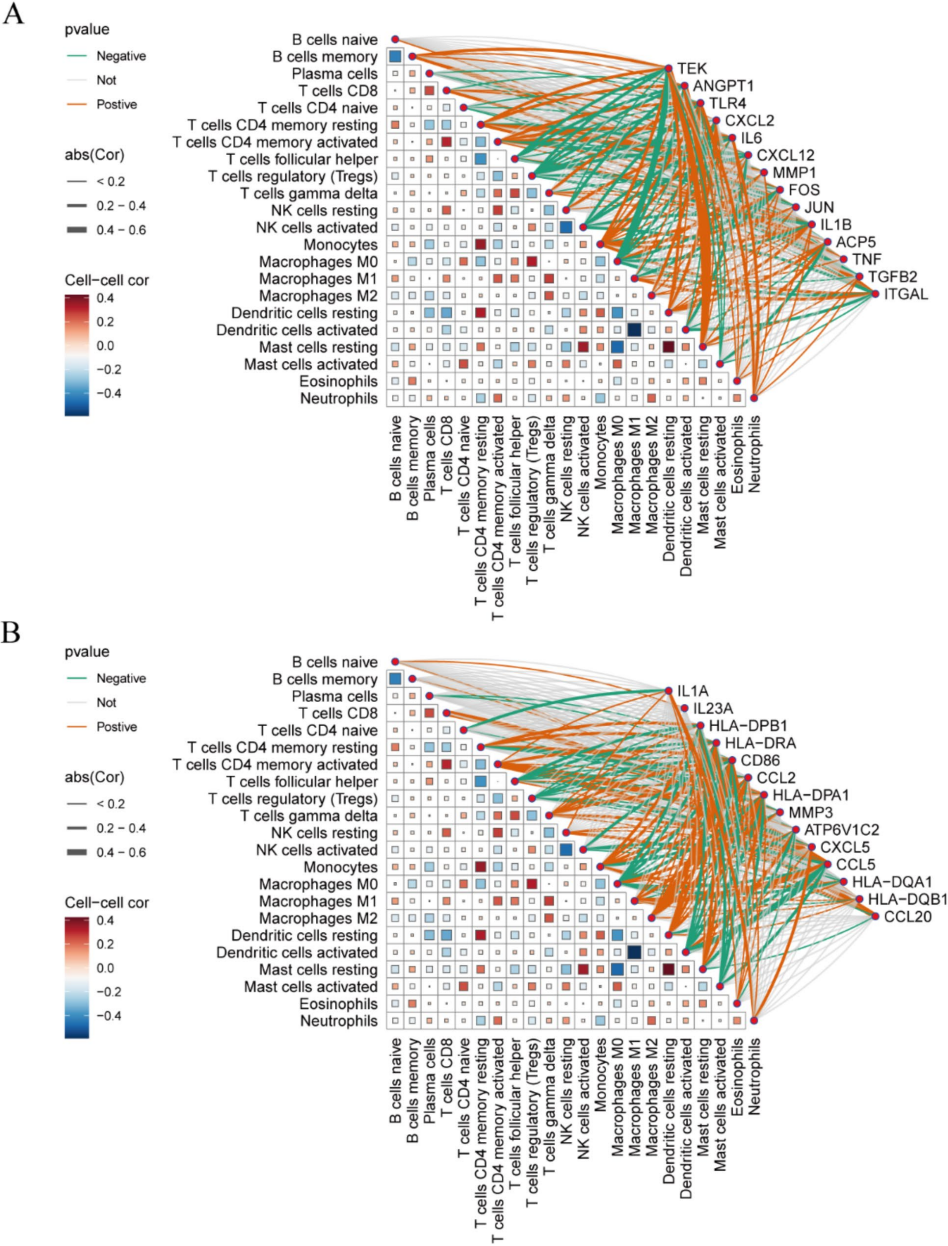
(**A**) Heatmap showing correlations between 22 immune cells and 14 related genes. (**B**) Heatmap showing correlations between 22 immune cells and additional related genes. **p < 0.01

Correlation analyses with 22 immune cells (Fig. 9A and B) revealed that, with the exception of MMP1, FOS, ACP5, TNF, IL1A, IL23A, MMP3, ATP6V1C2, HLA-DQB1 and CCL20, there was no correlation with T cells regulatory (Tregs). The remaining variables exhibited a negative correlation. TLR4, IL6, CXCL12, IL1B, ITGAL, CD86, CCL5, HLA-DQA1 and CCL20 were negatively correlated with NK cells activated, while the remaining variables demonstrated no significant correlation. While MMP1 and MMP3 demonstrated a positive correlation with macrophages M0, CXCL12, JUN, ACP5, TNF, IL1A, IL23A, ATP6V1C2, CXCL5, and CCL20 exhibited no significant correlation, while the remainder displayed a negative correlation.

Furthermore, the majority of the genes exhibited no correlation, either positive or negative, with B cells naïve, B cells memory, plasma cells, T cells CD4 naïve and T cells CD8. The majority of the genes exhibited a positive correlation, with only a few displaying a negative correlation or no correlation at all. The genes that showed a positive correlation included T cells CD4 memory resting, T cells CD4 memory activated, monocytes, macrophages M2, dendritic cells resting, eosinophils, and neutrophils. With regard to T cells follicular helper, dendritic cells activated and mast cells activated, the majority of genes exhibited a negative correlation, while only a small number demonstrated a positive correlation or no correlation.

The most strongly correlated genes with immune cells were TEK, ANGPT1, CD86, CCL5 and CCL20. TEK demonstrated the strongest negative correlation with macrophages M0 and the strongest positive correlation with ANGPT1 and mast cells resting. CCL5 exhibited the most robust negative correlation with dendritic cells activated, the most pronounced positive correlation with macrophages M1 and T cells CD8, and the most substantial positive correlation with T cells CD4 memory activated, a pattern mirrored by CCL20. CCL5 demonstrated the most robust positive correlation with activated dendritic cells.

### Validation group analysis of variance

**Fig. 10:**
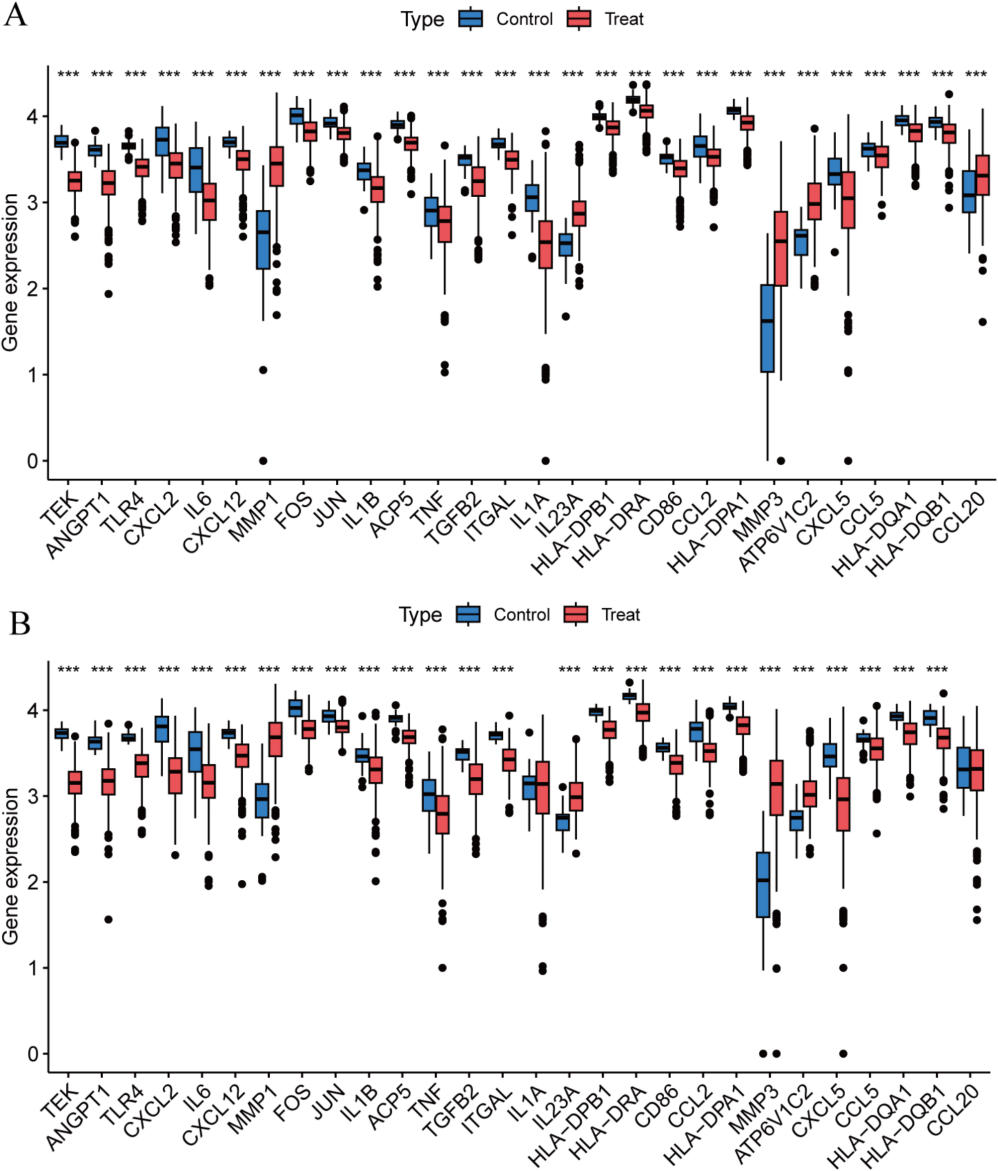
(**A**) Lung adenocarcinoma validation group difference analysis. (**B**) Analysis of differences in validation groups for squamous lung cancer. ***p < 0.001

The expression levels of the relevant genes identified in the KEGG enrichment analysis were validated (Fig. 10). The results showed that in the TCGA lung adenocarcinoma dataset, the expression levels of MMP1, IL23A, MMP3, ATP6V1C2 and CCL20 were significantly higher than those of the control group (*P* < 0.001), while the expression levels of other genes were significantly lower than those of the control group (*P* < 0.001, Fig. 10A). In the lung squamous cell carcinoma data set, the remaining genes, except IL1A and CCL20, also showed significant differences. Specifically, the expression levels of MMP1, IL23A, MMP3 and ATP6V1C2 were significantly higher than those of the control group (*P* < 0.001), whereas the expression levels of the other genes were significantly lower than those of the control group (*P* < 0.001, Fig. 10B).

Furthermore, the expression of TNF was found to be down-regulated in NSCLC samples, which is in accordance with the results of our MR analysis, thereby providing greater confidence in the MR results.

### Survival analysis

**Fig. 11:**
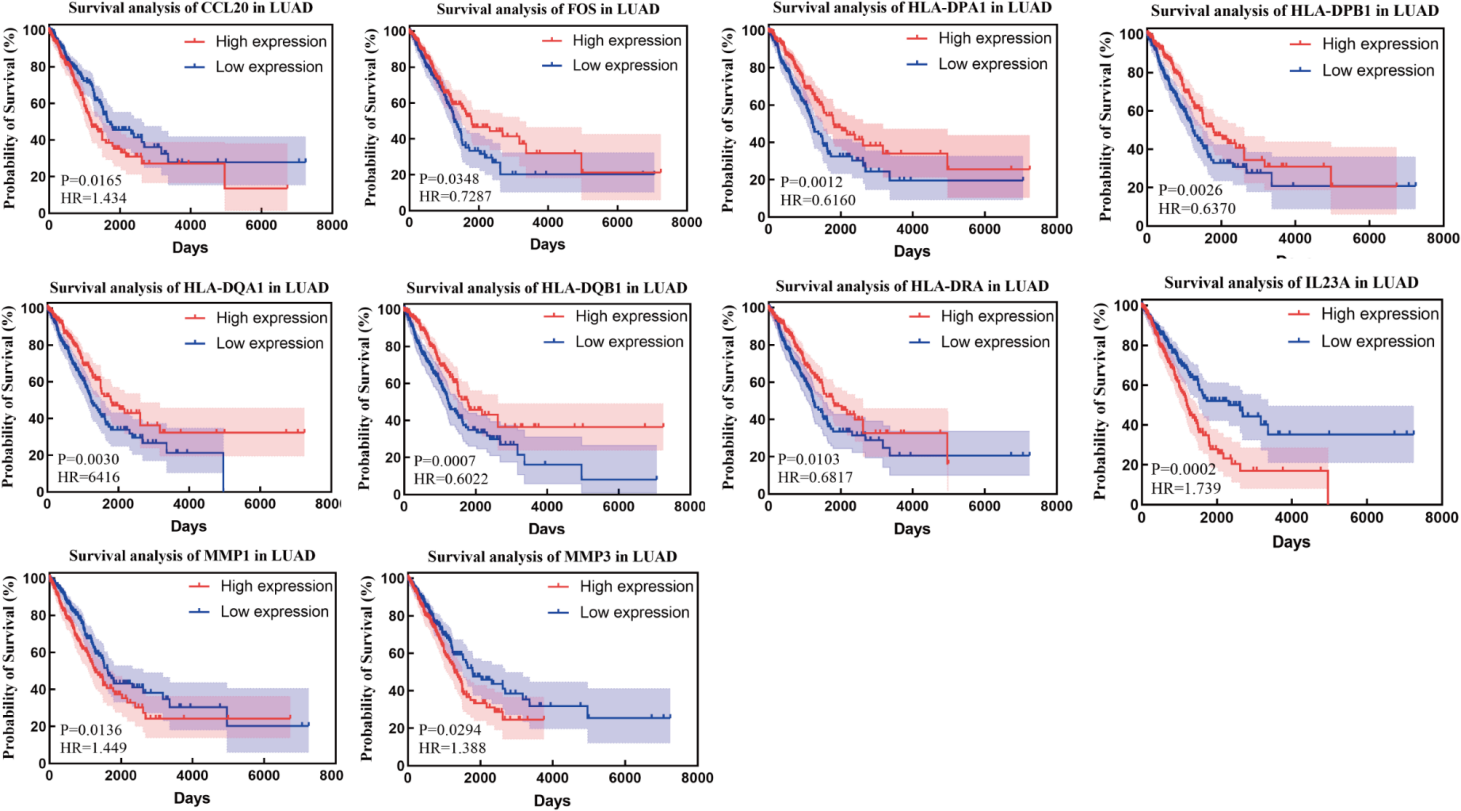
Survival analysis plots of statistically significant associated genes in lung adenocarcinoma

**Fig. 12:**
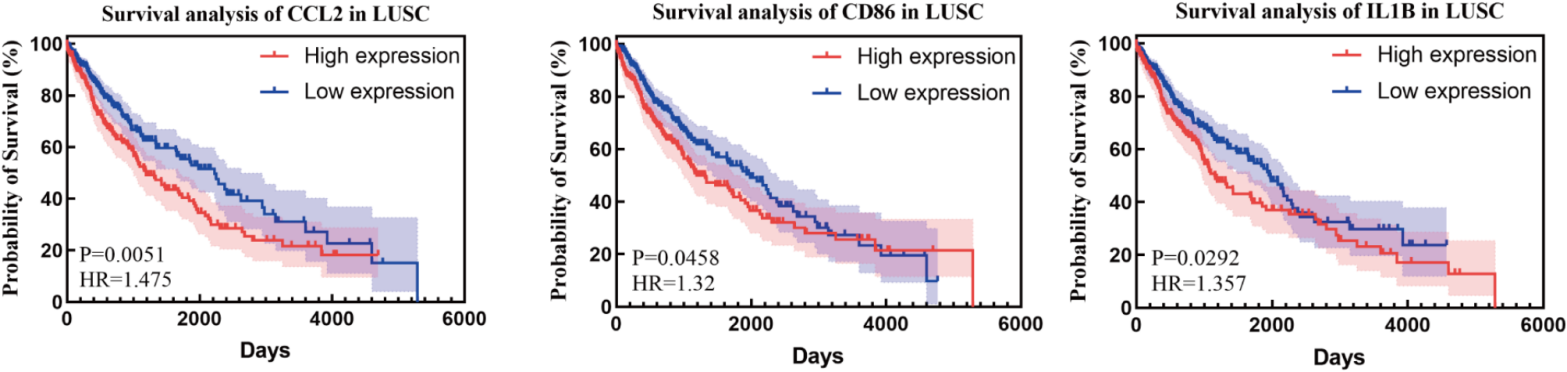
Survival analysis plots of statistically significant associated genes in squamous lung cancer

To explore the relationship between the genes of interest and clinical relevance, we integrated clinical data from the TCGA database and performed survival analyses of DEGs. The results showed that the expression levels of certain DEGs were significantly correlated with patient survival, which had important clinical implications for our study. Since the NSCLC data in the TCGA database included two subtypes, lung adenocarcinoma and lung squamous cell carcinoma, we analysed these two subtypes separately and plotted survival curves to obtain meaningful results (Figs. 11 and 12).

In lung adenocarcinoma, high expression of CCL20, IL23A, MMP1 and MMP3 was significantly associated with poor patient prognosis, whereas high expression of FOS, HLA-DPA1, HLA-DPB1, HLA-DQA1, HLA-DQB1 and HLA-DRA was significantly associated with improved patient prognosis. In squamous lung cancer, high expression of CCL2, CD86 and IL1B was significantly associated with poor patient prognosis. Through these analyses, we further validated the clinical relevance of the relevant genes in different lung cancer subtypes, thereby increasing the reliability and clinical application value of the findings.In addition, data from survival analysis results were collated for all genes (Supplementary Figure S4).

## Discussion

NSCLC represents a significant subtype of lung cancer, with a particularly poor prognosis in advanced stages. At present, there is no conclusive evidence to suggest that RA patients are at an elevated risk of developing NSCLC. Furthermore, the potential association between the two conditions has not been subjected to rigorous scientific investigation. The objective of this study was to investigate the potential association between RA and NSCLC through a comprehensive analysis of the GEO database, MR analysis, and assessment of immune cell infiltration. Additionally, we identified multiple NSCLC differential genes associated with RA through the construction of a competing endogenous RNA (ceRNA) network, which offers a novel direction for the treatment of NSCLC patients with RA and may serve as a future biomarker to support personalized therapy.

In our study, it was confirmed that the therapeutic agents for RA, the TNF inhibitors ADALIMUMAB, GOLIMUMAB, ETANERCEPT, CERTOLIZUMAB PEGOL, and INFLIXIMAB, increase the risk of NSCLC, a finding that provides an important addition to the existing studies on the association between RA and NSCLC. In addition, it has also been shown that the above drugs increase the risk of infection in patients [26–28], and pneumonia increases the risk of cancer in patients [29], indirectly confirming that TNF inhibitors may increase the risk of cancer. This also provides a clinical reference for the prevention of NSCLC in the treatment of RA patients.

In the KEGG enrichment analysis of related genes, we found that the IL-17 signalling pathway and the TNF signalling pathway were the significantly enriched pathways, suggesting that these genes may affect lung carcinogenesis through the immune pathway.IL-17, which is mainly secreted by Th17 cells, is able to promote lung carcinogenesis and progression by inducing tumour angiogenesis and enhancing tumour immune escape [30]. For example, IL-17 can promote tumour growth by activating the IL-6-Stat3 pathway [31]. Meanwhile, TNF can enhance the killing effect of T cells and other immune cells on tumour cells and increase neutrophil phagocytosis, thereby enhancing the body’s anti-tumour immune response [32]. In addition, TNF can induce tumour cell death by activating the intracellular apoptotic pathway. In the study of pancreatic ductal adenocarcinoma (PDAC), TNF promoted apoptosis of tumour cells by activating the CXCR2-MAPK-TNF signalling cascade response [33]. We also found that the expression of genes such as CXCL2, CXCL5 and TNF in the enriched pathway supported the function of this pathway. Therefore, we can infer that TNF inhibitors may inhibit the activation of this pathway by reducing the level of TNF, which may promote the development of NSCLC.

In immune cell analyses, T cells regulatory (Tregs), NK cells activated and Macrophages M0 were statistically significant.CCL5, IL6, TLR4, HLADQA1, IL1B, ITGAL, and CD86 were strongly associated with these three cells and showed inhibitory effects, and lower expression levels in NSCLC.

It is established that regulatory T cells (Tregs) have the capacity to suppress anticancer immunity, impede the protective immune surveillance of tumours and are strongly associated with poor prognosis [34]. In NSCLC, cytotoxic T-lymphocyte antigen-4 (CTLA-4) has been demonstrated to be a robust inducer of Treg function [35], and CD4 and CD8 Tregs may serve as potential prognostic biomarkers [36]. Nevertheless, augmenting the number of Tregs or fortifying their inhibitory capacity facilitates the treatment of autoimmune disorders [37], which is contrary to their role in NSCLC. Nevertheless, it has been demonstrated that the frequency of regulatory T cells (Tregs) in the peripheral blood of patients with early rheumatoid arthritis (RA) is markedly diminished [38]. Consequently, further research is required to gain a deeper understanding of the role of Tregs in RA patients, particularly in combination with NSCLC. NK cells possess the capacity to kill tumour cells and virus-infected cells in a non-specific manner. They can achieve this function by releasing lytic particles, secreting interferon gamma (IFNG) to induce target cell death, or by inducing death receptor-mediated cell death [39]. In NSCLC, immunotherapy targeting NK cells has significant potential, and NK cells may play a protective role in the pathogenesis of RA [40]. Macrophages are a heterogeneous population of cells, comprising multiple subtypes. M0 macrophages represent the basal state of macrophages, characterised by an inactive phenotype. Although not directly involved in the immune response in this state, they play a role in immune surveillance and cell signalling regulation. Tumour-associated macrophages are frequently the focus of therapeutic intervention [41, 42]. Conversely, in RA, M0-type macrophages can facilitate the onset and perpetuation of the inflammatory response by releasing inflammatory mediators [43, 44].

It can be observed that there is a strong correlation between the immune infiltration of tumour cells in NSCLC and the pathogenesis of RA. Furthermore, genes involved in immune regulation, particularly CCL5, IL6, TLR4, HLADQA1, IL1B, ITGAL and CD86, may represent potential future therapeutic targets.

In contrast, a previous stratification study of smoking and RA on NSCLC demonstrated that seropositive patients exhibited a two- to six-fold increased risk of NSCLC, even after adjusting for smoking in patients with RA [45]. Furthermore, patients who smoke, are seropositive or have interstitial lung disease are at an increased risk of recurrence of squamous cell carcinoma, even after surgical resection [46]. Furthermore, RA and NSCLC share numerous common risk factors, which gives rise to an intricate relationship between the two conditions. The findings of our study indicate that a specific class of RA therapeutic drugs may potentially increase the risk of developing NSCLC. However, they do not fully elucidate the underlying mechanisms of the association between NSCLC and RA, and they lack in vivo and in vitro experimental validation.

TNF plays a complex role in NSCLC. In RA patients, the use of anti-TNF drugs may increase the risk of NSCLC, a phenomenon that may be explained by the complex biological functions of TNF, the complexity of pharmacological intervention, and the differences between natural regulation and pharmacological intervention.

In the tumour microenvironment, TNF has a dual mechanism of action. On the one hand, TNF can activate T cells and other immune cells to enhance their ability to kill tumour cells. For example, TNF can enhance the phagocytic function of neutrophils, which boosts the body’s anti-tumour immune response [47]. In addition, TNF can induce tumour cell death by activating the intracellular apoptotic pathway. In pancreatic ductal adenocarcinoma (PDAC), TNF promotes tumour cell apoptosis by activating the CXCR2-MAPK-TNF signalling cascade response [33]. However, TNF can also promote tumour progression in certain circumstances. As a pro-inflammatory cytokine, TNF is capable of inducing a chronic inflammatory environment, which may provide favourable conditions for tumour development and progression [48]. In addition, TNF can promote tumour angiogenesis by inducing the expression of angiogenic factors that provide nutrients and oxygen to tumour cells, thereby promoting tumour growth [49].

In addition, the mechanism of pharmacological intervention is more complex: anti-TNF drugs treat inflammatory diseases such as rheumatoid arthritis by inhibiting the activity of TNF. However, this intervention may have the following complications: by inhibiting the activity of TNF, anti-TNF drugs may weaken the ability of the immune system to monitor and eliminate tumour cells, thereby increasing the risk of tumourigenesis [8]. TNF plays an important role in maintaining immune homeostasis. Anti-TNF drugs can disrupt this balance, resulting in the inability of the immune system to respond effectively to tumour cells [50].

At the same time, there is a significant difference in mechanism and effect between the natural regulation of TNF and pharmacological intervention. Under normal physiological conditions, the expression and activity of TNF is tightly regulated and can maintain a balance between anti-tumour and pro-inflammatory effects. This natural regulation helps to prevent tumour development and progression while avoiding excessive inflammatory responses [51]. In contrast, anti-TNF drugs treat inflammatory diseases by strongly inhibiting TNF activity. This can lead to low levels of TNF, which can weaken their anti-tumour effects and increase the risk of tumour formation [52].

In summary, the mechanism of action of TNF in NSCLC is extremely complex, and it has both a tumour-suppressing role in the tumour microenvironment and, under certain conditions, can also promote tumour development. Anti-TNF drugs treat inflammatory diseases by inhibiting TNF activity; however, this intervention may weaken the immune system’s ability to monitor and eliminate tumour cells, thereby increasing the risk of NSCLC.

There are several limitations to this study. Firstly, the lack of in vivo and in vitro experiments is a major shortcoming of this study. A follow-up study is planned to refine this part of the study to further validate the findings. Secondly, although the literature review suggests that merging data from different microarray platforms is feasible, such merging may still lead to biased results, which is another limitation of this study. In addition, the inherent limitations of bioinformatics tools and possible selection bias in the analysis process cannot be completely avoided.

## Conclusion

The findings of our study indicate that there is no causal association between RA and NSCLC. However, the therapeutic agent for RA, tumour necrosis factor (TNF) inhibitors, may potentially elevate the risk of developing NSCLC. Furthermore, our findings indicate a strong correlation between immune cell infiltration in NSCLC and RA. The genes identified through KEGG and ceRNA screening have the potential to serve as future biomarkers for NSCLC.

## Electronic supplementary material

Below is the link to the electronic supplementary material.

Supplementary Material 1

Supplementary Material 2

Supplementary Material 3

Supplementary Material 4

Supplementary Material 5

Supplementary Material 6

Supplementary Material 7

## Data Availability

No datasets were generated or analysed during the current study.

## Declarations

### Code involved

The relevant code is available at https://figshare.com/account/articles/28034132?file=51213800.
