# Supplementary figures and images for: Exploring the association between rheumatoid arthritis and non-small cell lung cancer risk: a transcriptomic and drug target-based analysis
Source: Hereditas. 2025 Feb 27;162:28. doi: 10.1186/s41065-025-00396-6 (PMC11866852; doi:10.1186/s41065-025-00396-6)

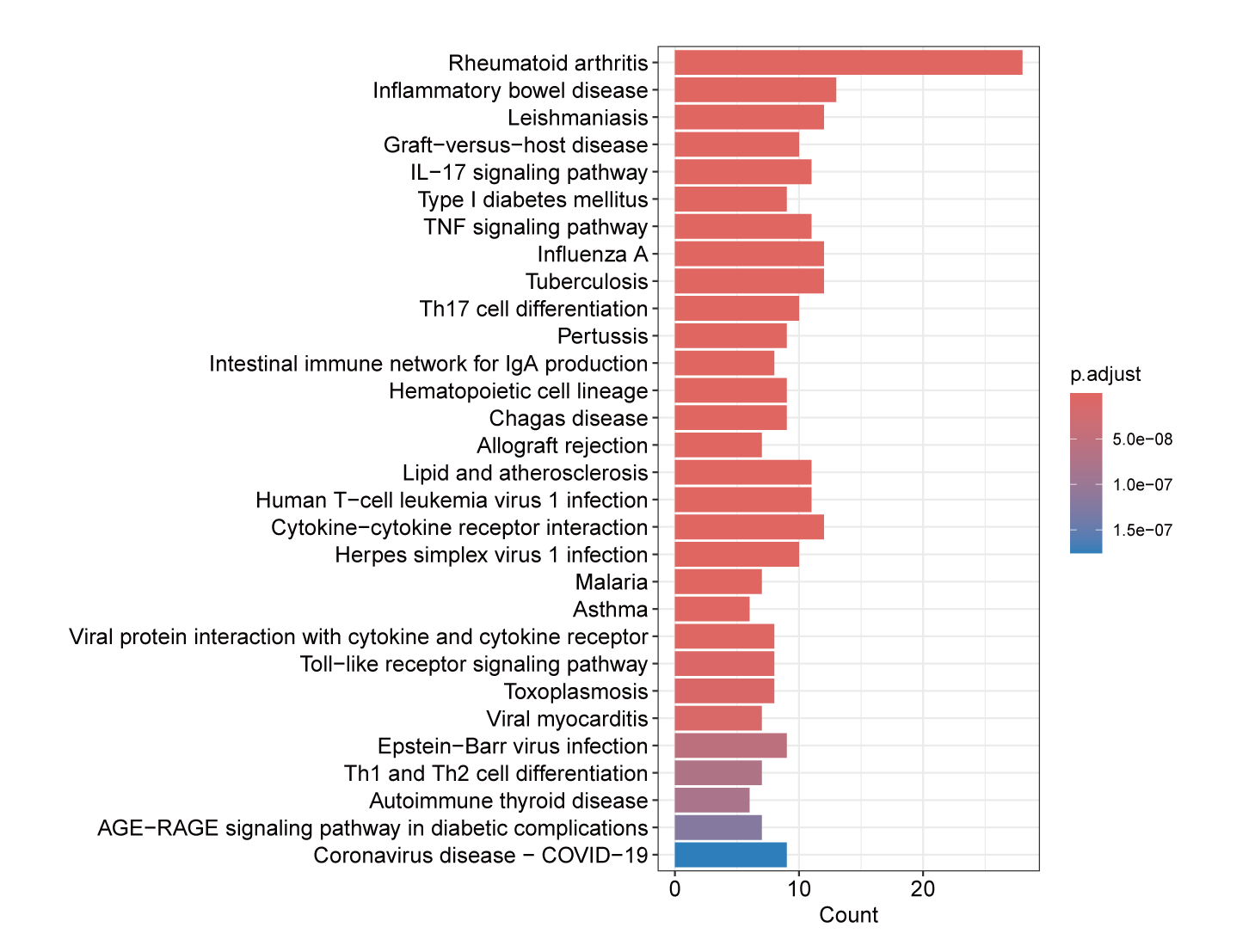

Supplement: Supplementary file 1 — Supplementary Material 1 [file 41065_2025_396_MOESM1_ESM.tif]

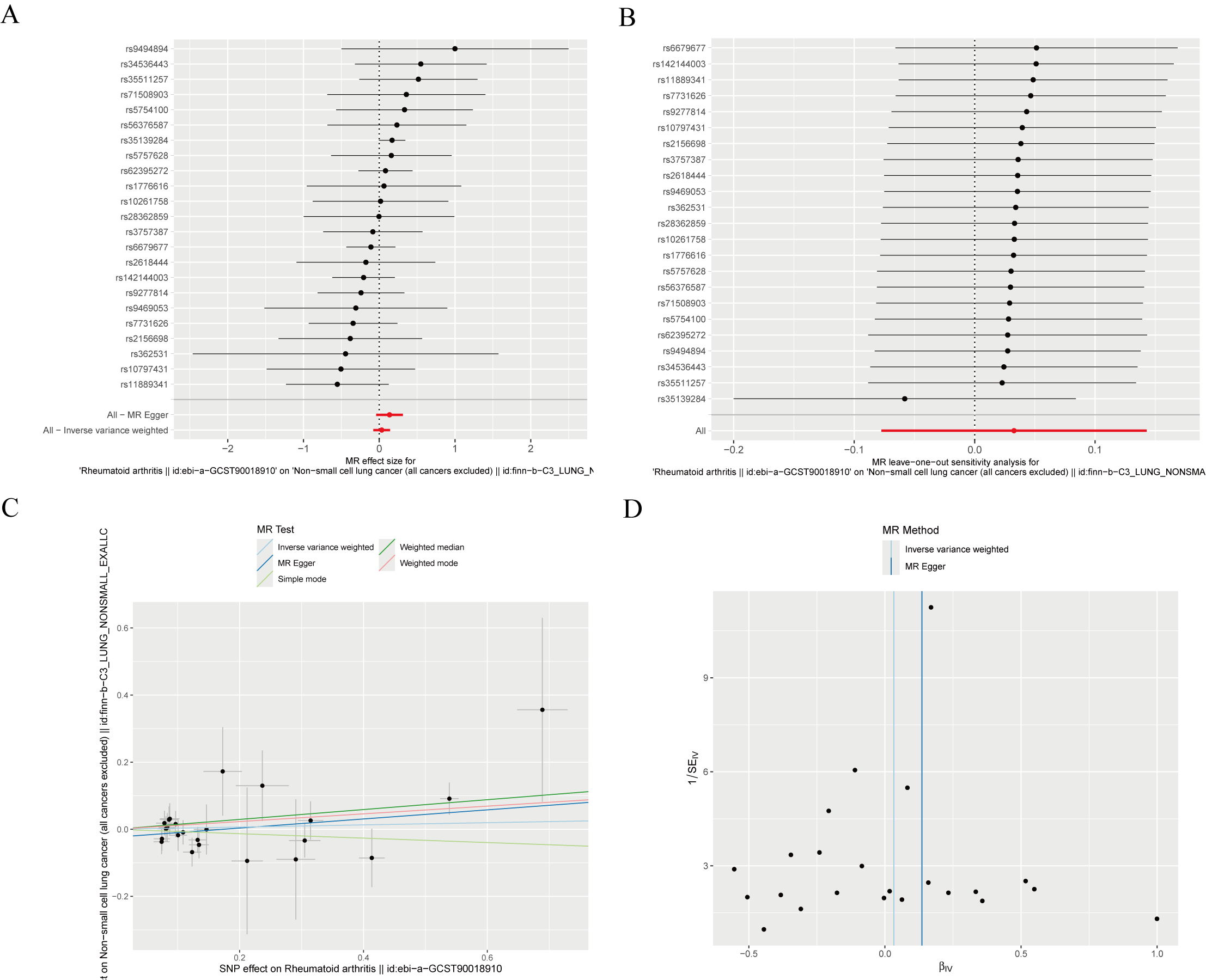

Supplement: Supplementary file 2 — Supplementary Material 2 [file 41065_2025_396_MOESM2_ESM.tif]

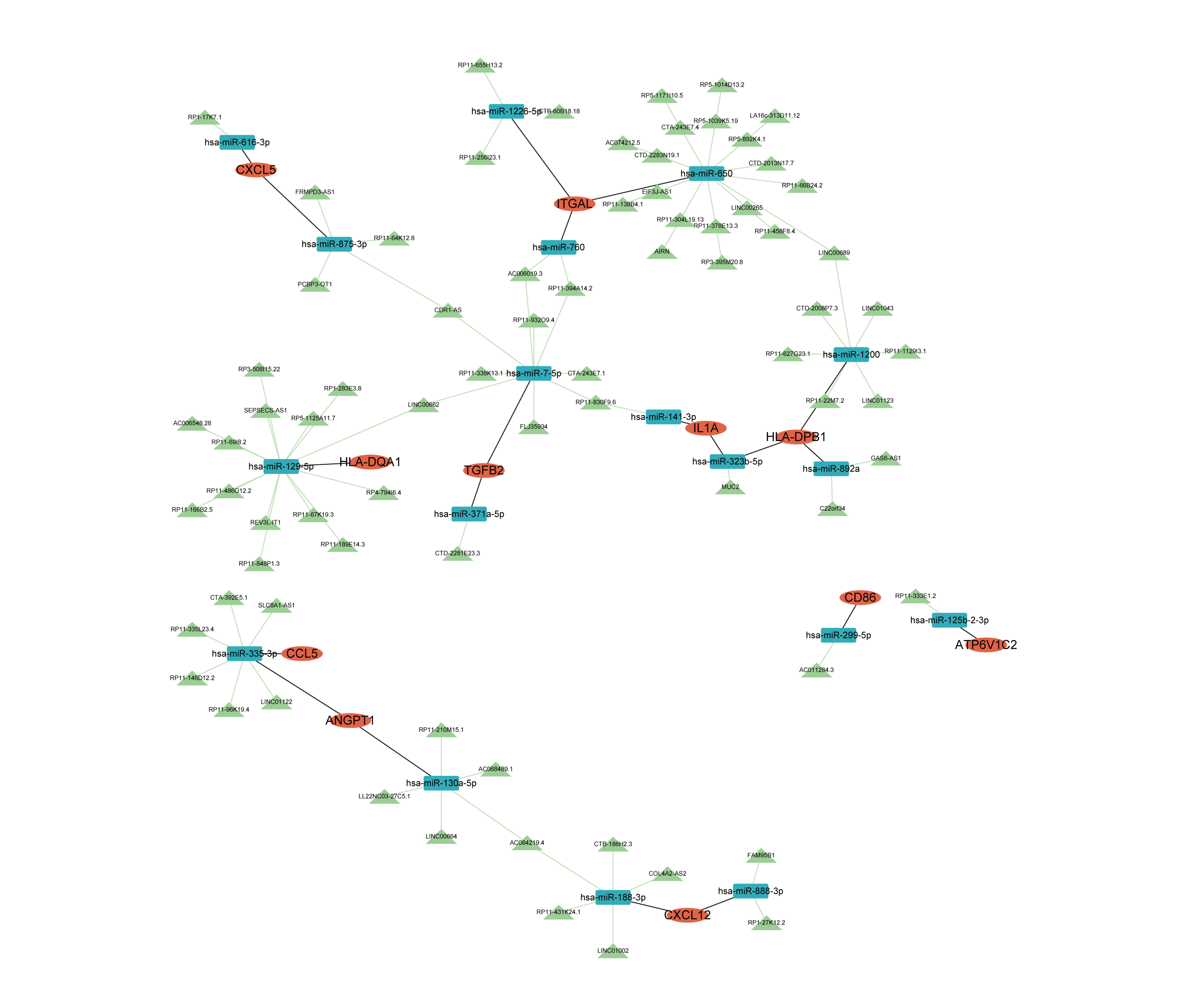

Supplement: Supplementary file 3 — Supplementary Material 3 [file 41065_2025_396_MOESM3_ESM.tif]

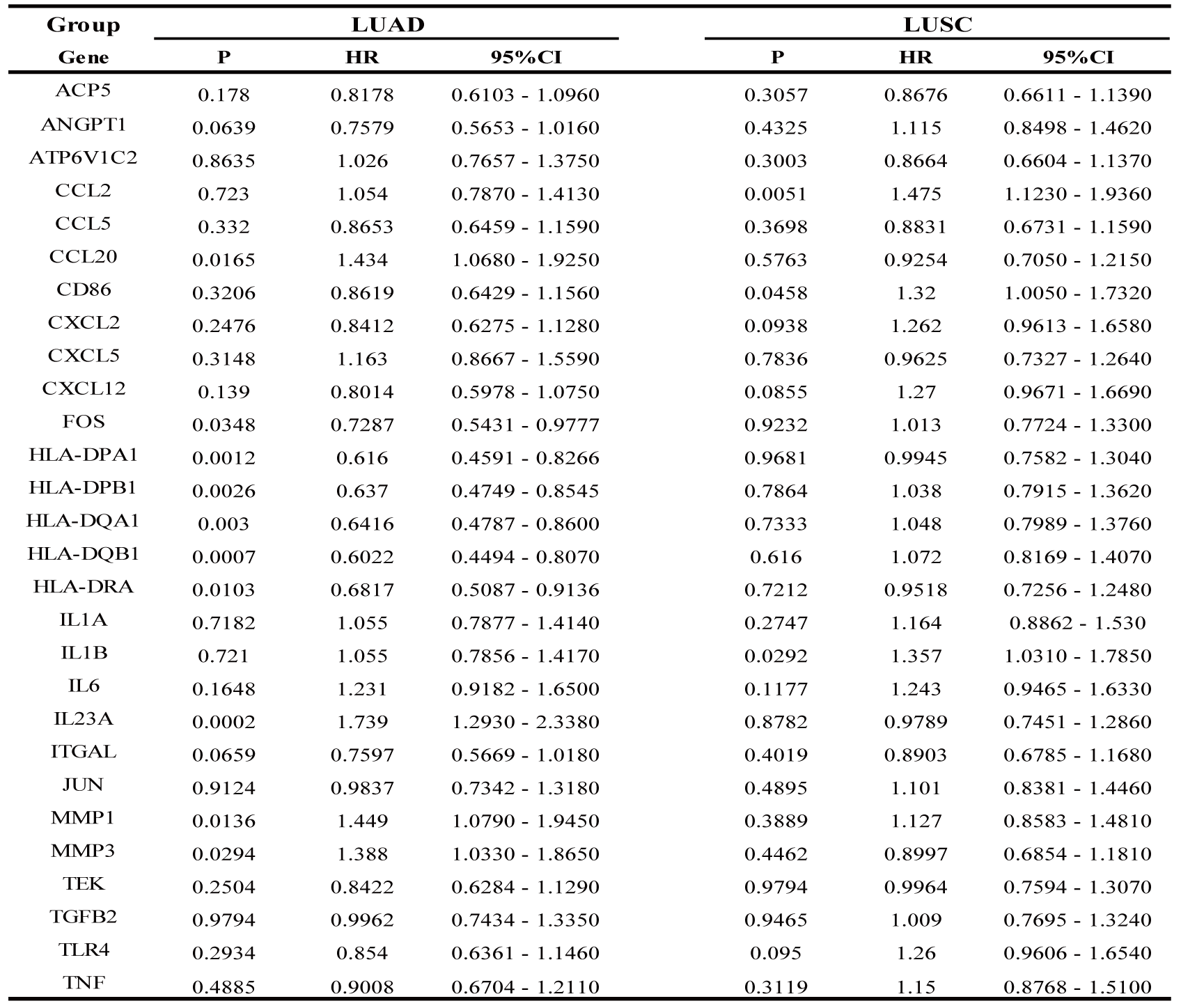

Supplement: Supplementary file 4 — Supplementary Material 4 [file 41065_2025_396_MOESM4_ESM.tif]
