# Supplementary material for: Exploring the association between rheumatoid arthritis and non-small cell lung cancer risk: a transcriptomic and drug target-based analysis
Source: Hereditas. 2025 Feb 27;162:28. doi: 10.1186/s41065-025-00396-6 (PMC11866852; doi:10.1186/s41065-025-00396-6)
Supplement: Supplementary file 5 — Supplementary Material 5 [file 41065_2025_396_MOESM5_ESM.docx]

**Supplementary Table S1**

**List of Differential Genes(logFC>0.585, P<0.01)**

| id | logFC | AveExpr | t | P.Value | adj.P.Val | B |
| --- | --- | --- | --- | --- | --- | --- |
| AGER | -4.254439965 | 7.960157572 | -31.98742572 | 1.23E-109 | 1.32E-105 | 239.7427191 |
| SGCG | -2.261149259 | 5.075695981 | -30.51889307 | 4.58E-104 | 1.72E-100 | 226.9707105 |
| GPM6A | -2.436662547 | 5.728990641 | -30.51412597 | 4.78E-104 | 1.72E-100 | 226.928812 |
| FAM107A | -3.672612104 | 8.191672745 | -30.27717756 | 3.88E-103 | 1.05E-99 | 224.8427078 |
| GRK5 | -1.828127005 | 7.579484114 | -30.17514514 | 9.59E-103 | 2.07E-99 | 223.9422738 |
| CA4 | -3.791518299 | 7.564183739 | -30.08011159 | 2.23E-102 | 4.00E-99 | 223.102451 |
| TEK | -3.061124632 | 7.886321681 | -29.61713306 | 1.38E-100 | 2.12E-97 | 218.9952135 |
| TCF21 | -3.145912309 | 7.171314886 | -29.17090848 | 7.51E-99 | 1.01E-95 | 215.0119183 |
| FABP4 | -4.49798775 | 7.155313301 | -29.02346985 | 2.83E-98 | 3.39E-95 | 213.6905049 |
| ADAMTS8 | -2.644054136 | 6.793625822 | -28.89415455 | 9.09E-98 | 9.79E-95 | 212.5293775 |
| CLEC3B | -3.348499644 | 8.705010508 | -28.8307948 | 1.61E-97 | 1.58E-94 | 211.9597389 |
| STX11 | -2.617149572 | 7.821195875 | -28.63116758 | 9.78E-97 | 8.79E-94 | 210.1618604 |
| FHL1 | -3.05327248 | 8.243746997 | -28.35286772 | 1.22E-95 | 1.01E-92 | 207.6475812 |
| CLEC1A | -2.222393829 | 6.843916122 | -28.19723521 | 5.03E-95 | 3.87E-92 | 206.2375694 |
| PTPRB | -2.65181604 | 7.441348191 | -28.10709334 | 1.14E-94 | 8.21E-92 | 205.4196036 |
| RHOJ | -2.103057799 | 7.09700183 | -27.99406482 | 3.20E-94 | 2.16E-91 | 204.3926276 |
| ACVRL1 | -2.102470507 | 7.866696548 | -27.97082318 | 3.96E-94 | 2.51E-91 | 204.1812711 |
| SPOCK2 | -2.302166062 | 9.487348473 | -27.81700997 | 1.62E-93 | 9.67E-91 | 202.7809447 |
| CDH5 | -2.366825207 | 9.178952338 | -27.46876099 | 3.93E-92 | 2.23E-89 | 199.6004663 |
| LDB2 | -2.308233112 | 9.269196301 | -27.03222825 | 2.19E-90 | 1.18E-87 | 195.5944128 |
| LGI3 | -2.362269293 | 6.236095809 | -26.96347918 | 4.14E-90 | 2.12E-87 | 194.9615748 |
| GPR146 | -1.820579044 | 6.360217734 | -26.83680599 | 1.34E-89 | 6.54E-87 | 193.7941841 |
| PECAM1 | -1.978502089 | 10.23173212 | -26.65144623 | 7.44E-89 | 3.48E-86 | 192.0827996 |
| CAV1 | -2.998837197 | 10.25957267 | -26.34391388 | 1.30E-87 | 5.82E-85 | 189.2352661 |
| SPTBN1 | -1.627850752 | 8.925582609 | -26.33954408 | 1.35E-87 | 5.82E-85 | 189.1947322 |
| FHL5 | -2.273041393 | 5.762339524 | -26.33480948 | 1.41E-87 | 5.84E-85 | 189.1508122 |
| SLC6A4 | -3.97038875 | 6.172017668 | -26.27946201 | 2.36E-87 | 9.42E-85 | 188.637212 |
| KL | -2.302750405 | 6.02928933 | -26.17737913 | 6.12E-87 | 2.35E-84 | 187.6890825 |
| CDO1 | -2.395392434 | 6.260620782 | -26.16355043 | 6.96E-87 | 2.58E-84 | 187.5605598 |
| SDPR | -2.795827832 | 8.436756553 | -26.0497119 | 2.01E-86 | 7.23E-84 | 186.5017969 |
| FMO2 | -2.85768389 | 9.12521734 | -26.023947 | 2.56E-86 | 8.90E-84 | 186.2619819 |
| JAM2 | -2.285450137 | 7.814027396 | -26.01719342 | 2.73E-86 | 9.18E-84 | 186.1991095 |
| TMEM100 | -4.129029917 | 7.262499436 | -26.00060898 | 3.18E-86 | 1.04E-83 | 186.0446968 |
| SH3GL3 | -2.26130229 | 5.528152231 | -25.99649239 | 3.31E-86 | 1.05E-83 | 186.006364 |
| SASH1 | -1.753435008 | 8.637874992 | -25.94714136 | 5.25E-86 | 1.62E-83 | 185.5466824 |
| ASPA | -1.705017024 | 4.779621537 | -25.75373388 | 3.21E-85 | 9.60E-83 | 183.7427782 |
| TIE1 | -1.74260257 | 7.149845457 | -25.73620415 | 3.78E-85 | 1.10E-82 | 183.579091 |
| MCEMP1 | -3.520034542 | 8.753460285 | -25.72676242 | 4.13E-85 | 1.17E-82 | 183.4909142 |
| TNNC1 | -2.974094486 | 7.624040949 | -25.59653368 | 1.40E-84 | 3.87E-82 | 182.2737844 |
| FGD5 | -1.881134727 | 7.709530194 | -25.35804457 | 1.32E-83 | 3.55E-81 | 180.0404618 |
| GDF10 | -2.649924756 | 6.360974011 | -25.32254702 | 1.84E-83 | 4.84E-81 | 179.7075672 |
| ROBO4 | -1.78455323 | 6.804879285 | -25.28301489 | 2.67E-83 | 6.85E-81 | 179.3366919 |
| IGSF10 | -2.326354862 | 5.779839285 | -25.25262817 | 3.55E-83 | 8.90E-81 | 179.051512 |
| C14orf132 | -2.018329116 | 7.82452616 | -25.23386231 | 4.24E-83 | 1.02E-80 | 178.8753493 |
| HIGD1B | -2.637806626 | 7.690096705 | -25.23295093 | 4.28E-83 | 1.02E-80 | 178.866793 |
| EDNRB | -2.168772011 | 6.55582034 | -25.10865051 | 1.38E-82 | 3.23E-80 | 177.6990663 |
| GIMAP8 | -1.973053469 | 8.155476811 | -25.10630494 | 1.41E-82 | 3.24E-80 | 177.6770168 |
| CLEC14A | -1.984449995 | 8.85848612 | -25.07793473 | 1.84E-82 | 4.14E-80 | 177.410282 |
| EPAS1 | -1.892796276 | 11.05636128 | -24.90698131 | 9.27E-82 | 2.04E-79 | 175.8013721 |
| PKNOX2 | -1.690576891 | 6.461741782 | -24.90356309 | 9.57E-82 | 2.06E-79 | 175.7691738 |
| LRRN3 | -2.429033081 | 5.944814266 | -24.86032658 | 1.44E-81 | 3.04E-79 | 175.3618084 |
| EMCN | -2.951831395 | 7.351757755 | -24.84965103 | 1.59E-81 | 3.30E-79 | 175.2611987 |
| LIMS2 | -1.803816288 | 7.592575886 | -24.82154785 | 2.08E-81 | 4.23E-79 | 174.996295 |
| GPD1 | -2.001145766 | 5.73922841 | -24.77403843 | 3.26E-81 | 6.50E-79 | 174.5482991 |
| RAMP3 | -2.292013392 | 8.090357944 | -24.72511195 | 5.18E-81 | 1.01E-78 | 174.086723 |
| STXBP6 | -2.606763911 | 6.569335306 | -24.71626413 | 5.63E-81 | 1.08E-78 | 174.0032285 |
| AGTR1 | -2.036010516 | 5.951506686 | -24.68343628 | 7.68E-81 | 1.45E-78 | 173.6933781 |
| FXYD1 | -1.854006061 | 6.808408846 | -24.55588079 | 2.57E-80 | 4.78E-78 | 172.4884948 |
| ACADL | -2.868441404 | 5.962273529 | -24.55293978 | 2.65E-80 | 4.83E-78 | 172.4606968 |
| BTNL9 | -2.184134603 | 6.10350912 | -24.43711996 | 7.94E-80 | 1.43E-77 | 171.3653666 |
| SEMA6A | -2.388273759 | 7.457730497 | -24.1851839 | 8.71E-79 | 1.54E-76 | 168.9786644 |
| RAMP2 | -1.831539477 | 7.654326729 | -24.17864655 | 9.27E-79 | 1.61E-76 | 168.9166597 |
| FIGF | -3.222699638 | 6.534943686 | -24.17399633 | 9.69E-79 | 1.66E-76 | 168.8725515 |
| MYCT1 | -1.717495078 | 6.038121346 | -24.16287651 | 1.08E-78 | 1.81E-76 | 168.7670706 |
| TGFBR3 | -2.302792352 | 8.511765431 | -24.04581649 | 3.28E-78 | 5.44E-76 | 167.6560157 |
| GSTM5 | -1.96629564 | 6.443636606 | -24.00062166 | 5.05E-78 | 8.25E-76 | 167.226746 |
| ABCA8 | -2.575088074 | 6.389152537 | -23.85641056 | 2.00E-77 | 3.21E-75 | 165.8558577 |
| PYCR1 | 1.384399978 | 6.924020372 | 23.69994546 | 8.91E-77 | 1.41E-74 | 164.3665472 |
| CD36 | -2.971086621 | 7.221724862 | -23.61759746 | 1.96E-76 | 3.06E-74 | 163.5819261 |
| CYYR1 | -2.239987011 | 7.951297008 | -23.5312847 | 4.47E-76 | 6.88E-74 | 162.758951 |
| CLIC5 | -2.739383772 | 7.965318577 | -23.52250057 | 4.86E-76 | 7.38E-74 | 162.6751631 |
| ADH1B | -3.872755793 | 7.508928322 | -23.44683837 | 1.00E-75 | 1.50E-73 | 161.9532077 |
| DES | -2.236469538 | 6.766571051 | -23.44162892 | 1.05E-75 | 1.56E-73 | 161.9034837 |
| ST6GALNAC3 | -1.415624183 | 5.371508809 | -23.43385255 | 1.14E-75 | 1.65E-73 | 161.8292547 |
| LRRC36 | -2.445997511 | 6.179516356 | -23.35297137 | 2.46E-75 | 3.54E-73 | 161.056932 |
| FCN3 | -3.55191796 | 8.601767327 | -23.19832971 | 1.09E-74 | 1.54E-72 | 159.5789049 |
| RSPO2 | -1.319866734 | 5.095158441 | -23.18630372 | 1.22E-74 | 1.71E-72 | 159.4638888 |
| MMRN1 | -2.839088168 | 6.521308463 | -23.16868487 | 1.44E-74 | 1.99E-72 | 159.2953636 |
| SYT15 | -1.245276403 | 6.815708795 | -23.12152944 | 2.27E-74 | 3.09E-72 | 158.8442075 |
| MFNG | -1.343944327 | 8.064821476 | -23.10820736 | 2.58E-74 | 3.47E-72 | 158.7167201 |
| GIMAP6 | -1.502086406 | 7.067481981 | -23.08508094 | 3.22E-74 | 4.28E-72 | 158.4953782 |
| VWF | -1.862046607 | 11.01254832 | -23.08156188 | 3.33E-74 | 4.38E-72 | 158.4616941 |
| CLDN5 | -2.000272537 | 9.250889524 | -23.02227598 | 5.89E-74 | 7.64E-72 | 157.8940804 |
| BCHE | -2.563916974 | 5.657242173 | -22.99329365 | 7.78E-74 | 9.97E-72 | 157.6165068 |
| ESAM | -1.840739826 | 8.590190548 | -22.94372794 | 1.25E-73 | 1.59E-71 | 157.1416615 |
| DPEP2 | -1.884227311 | 7.355190439 | -22.92251444 | 1.54E-73 | 1.92E-71 | 156.9383808 |
| SVEP1 | -2.10408024 | 8.157301215 | -22.92010913 | 1.57E-73 | 1.95E-71 | 156.9153297 |
| COX7A1 | -1.70631398 | 8.475214593 | -22.8863231 | 2.17E-73 | 2.66E-71 | 156.5915008 |
| C10orf67 | -2.033202598 | 5.509140085 | -22.8533083 | 2.99E-73 | 3.62E-71 | 156.2749872 |
| CALCRL | -2.638173798 | 7.7188195 | -22.80080104 | 4.95E-73 | 5.93E-71 | 155.7714445 |
| CORO2B | -1.330088261 | 5.420782763 | -22.63109474 | 2.54E-72 | 3.00E-70 | 154.1426922 |
| LEPR | -1.621557488 | 6.723916707 | -22.61749175 | 2.89E-72 | 3.39E-70 | 154.012055 |
| FXYD6 | -1.721049782 | 8.725151761 | -22.61474769 | 2.97E-72 | 3.44E-70 | 153.9857008 |
| RASIP1 | -1.815383511 | 8.254043944 | -22.58750374 | 3.86E-72 | 4.43E-70 | 153.7240204 |
| HBB | -3.321753463 | 10.77811838 | -22.53975708 | 6.12E-72 | 6.94E-70 | 153.2652936 |
| FEZ1 | -1.409083804 | 6.896971056 | -22.5331101 | 6.52E-72 | 7.32E-70 | 153.201421 |
| MFAP4 | -2.638251988 | 9.917923479 | -22.50751481 | 8.35E-72 | 9.27E-70 | 152.9554424 |
| ADAMTSL3 | -1.844376273 | 6.153216894 | -22.49779549 | 9.17E-72 | 1.01E-69 | 152.8620259 |
| CASQ2 | -1.938540251 | 5.593391505 | -22.41845252 | 1.97E-71 | 2.15E-69 | 152.0992031 |
| HEG1 | -1.779392185 | 8.937532521 | -22.4091845 | 2.16E-71 | 2.32E-69 | 152.0100723 |
| ANGPT1 | -2.256268192 | 7.133846426 | -22.3974074 | 2.41E-71 | 2.58E-69 | 151.8968039 |
| SH2D3C | -1.351022642 | 7.067396987 | -22.38619178 | 2.69E-71 | 2.84E-69 | 151.7889275 |
| DACH1 | -1.525594457 | 5.532508617 | -22.38498941 | 2.72E-71 | 2.85E-69 | 151.7773623 |
| SPP1 | 3.751285711 | 9.661389753 | 22.37639009 | 2.96E-71 | 3.06E-69 | 151.6946447 |
| PLAC9 | -2.179062061 | 7.975059069 | -22.35004156 | 3.81E-71 | 3.91E-69 | 151.4411676 |
| FOXF1 | -2.189651693 | 7.375568606 | -22.27903464 | 7.57E-71 | 7.70E-69 | 150.7578586 |
| TMOD1 | -1.936921625 | 6.977068931 | -22.26111722 | 9.00E-71 | 9.06E-69 | 150.5853888 |
| LTBP4 | -1.504599817 | 7.854815285 | -22.24252116 | 1.08E-70 | 1.07E-68 | 150.4063661 |
| HSPB8 | -2.148993449 | 7.848669213 | -22.23803371 | 1.12E-70 | 1.11E-68 | 150.3631628 |
| SCN4B | -2.011658739 | 6.468876279 | -22.21650895 | 1.38E-70 | 1.36E-68 | 150.1559144 |
| TACC1 | -1.43759981 | 10.11262103 | -22.17088926 | 2.15E-70 | 2.09E-68 | 149.7165805 |
| ADRB2 | -2.050956186 | 7.928935048 | -22.16649197 | 2.25E-70 | 2.16E-68 | 149.6742267 |
| FGFR4 | -1.645983415 | 6.210419104 | -22.13844232 | 2.94E-70 | 2.81E-68 | 149.4040309 |
| CAT | -1.496675769 | 9.685194832 | -22.11986282 | 3.52E-70 | 3.33E-68 | 149.2250339 |
| HSPA12B | -1.752568866 | 7.314123436 | -22.07087132 | 5.66E-70 | 5.30E-68 | 148.7529491 |
| MMRN2 | -1.568003275 | 6.134045926 | -22.01574023 | 9.64E-70 | 8.96E-68 | 148.2215401 |
| TMEM88 | -1.796134289 | 6.828931093 | -21.9954354 | 1.17E-69 | 1.08E-67 | 148.0257789 |
| SOSTDC1 | -3.392042636 | 6.638450082 | -21.98454956 | 1.30E-69 | 1.19E-67 | 147.9208177 |
| FZD4 | -1.459143206 | 8.558365979 | -21.97754068 | 1.40E-69 | 1.26E-67 | 147.8532348 |
| CRBN | -0.95440398 | 9.116502992 | -21.96768343 | 1.53E-69 | 1.38E-67 | 147.7581818 |
| MAMDC2 | -3.265966877 | 7.845951082 | -21.95109251 | 1.80E-69 | 1.60E-67 | 147.5981844 |
| ABCA9 | -1.483752917 | 5.60718496 | -21.93879912 | 2.03E-69 | 1.79E-67 | 147.4796213 |
| TOP2A | 3.112736191 | 7.73100091 | 21.93226089 | 2.16E-69 | 1.89E-67 | 147.4165602 |
| KCNT2 | -1.492468179 | 5.104925032 | -21.90317212 | 2.87E-69 | 2.49E-67 | 147.1359716 |
| OTUD1 | -1.465835509 | 7.593409096 | -21.84107606 | 5.23E-69 | 4.51E-67 | 146.5368446 |
| AFF3 | -1.853524598 | 5.551711418 | -21.8314878 | 5.74E-69 | 4.90E-67 | 146.444315 |
| CD300LG | -1.206215081 | 6.239922484 | -21.80645832 | 7.31E-69 | 6.20E-67 | 146.2027504 |
| DNASE1L3 | -2.752038466 | 6.246799114 | -21.71146678 | 1.83E-68 | 1.54E-66 | 145.2856723 |
| STARD8 | -1.434531838 | 7.226988971 | -21.65644194 | 3.13E-68 | 2.61E-66 | 144.7542357 |
| SNRK | -1.06186265 | 9.452176758 | -21.64285359 | 3.57E-68 | 2.95E-66 | 144.6229745 |
| CRYAB | -2.265384466 | 8.147347505 | -21.64211354 | 3.59E-68 | 2.95E-66 | 144.6158255 |
| NTNG1 | -2.112168559 | 5.728388375 | -21.5225773 | 1.14E-67 | 9.34E-66 | 143.4607342 |
| TGFBR2 | -1.15548591 | 9.406366928 | -21.49866846 | 1.44E-67 | 1.17E-65 | 143.2296188 |
| PGM5 | -1.860983264 | 6.763569199 | -21.41994956 | 3.10E-67 | 2.49E-65 | 142.4684934 |
| PPP1R15A | -1.482959096 | 9.767735319 | -21.38176646 | 4.48E-67 | 3.58E-65 | 142.0992032 |
| RASL12 | -1.576882125 | 8.162949673 | -21.35968939 | 5.56E-67 | 4.40E-65 | 141.8856539 |
| CLDN18 | -3.352687907 | 7.840179122 | -21.34949834 | 6.13E-67 | 4.82E-65 | 141.7870695 |
| C10orf54 | -1.501639311 | 7.991352681 | -21.32804448 | 7.55E-67 | 5.90E-65 | 141.5795183 |
| TCEAL2 | -2.233202812 | 6.16013941 | -21.30141144 | 9.78E-67 | 7.58E-65 | 141.3218342 |
| TBX3 | -1.161227804 | 6.50212846 | -21.29912601 | 1.00E-66 | 7.70E-65 | 141.2997204 |
| AOX1 | -2.137902771 | 6.101409505 | -21.28592416 | 1.14E-66 | 8.69E-65 | 141.1719753 |
| VIPR1 | -2.194520933 | 8.823182061 | -21.27822345 | 1.23E-66 | 9.30E-65 | 141.0974573 |
| SLIT2 | -1.928526004 | 9.029531434 | -21.2370322 | 1.83E-66 | 1.38E-64 | 140.6988171 |
| TBX4 | -1.599683477 | 5.892166835 | -21.20397777 | 2.52E-66 | 1.88E-64 | 140.378872 |
| DENND3 | -1.226831303 | 7.731018394 | -21.19923811 | 2.64E-66 | 1.96E-64 | 140.3329915 |
| ANKRD1 | -2.824765835 | 5.825159824 | -21.18643054 | 2.99E-66 | 2.20E-64 | 140.2090081 |
| RRAS | -1.362140124 | 10.37011349 | -21.16224477 | 3.78E-66 | 2.77E-64 | 139.9748601 |
| PDE2A | -1.484387072 | 6.222622617 | -21.15939776 | 3.88E-66 | 2.83E-64 | 139.947296 |
| EML1 | -1.460093153 | 6.481232614 | -21.15750635 | 3.96E-66 | 2.86E-64 | 139.9289836 |
| PRKCH | -1.073830715 | 9.205043048 | -21.14700924 | 4.38E-66 | 3.15E-64 | 139.8273493 |
| UBE2T | 2.247147845 | 7.239424601 | 21.08530722 | 7.98E-66 | 5.69E-64 | 139.2298545 |
| SMAD6 | -2.011548938 | 8.187192218 | -21.07743079 | 8.61E-66 | 6.10E-64 | 139.1535719 |
| OCIAD2 | 1.586113693 | 9.830494699 | 21.05681434 | 1.05E-65 | 7.41E-64 | 138.9538921 |
| PRKCE | -1.545668329 | 7.331365434 | -21.00915985 | 1.67E-65 | 1.17E-63 | 138.4922746 |
| GPM6B | -2.103924429 | 6.21869192 | -20.99559312 | 1.91E-65 | 1.33E-63 | 138.3608413 |
| CDH13 | -1.926671647 | 6.363964636 | -20.99499197 | 1.92E-65 | 1.33E-63 | 138.3550173 |
| SPARCL1 | -1.932526755 | 11.15375452 | -20.95379324 | 2.86E-65 | 1.97E-63 | 137.9558454 |
| LRRC32 | -1.623989721 | 9.061476008 | -20.94897829 | 3.00E-65 | 2.05E-63 | 137.9091897 |
| PAFAH1B3 | 1.404061633 | 9.239393465 | 20.94548546 | 3.10E-65 | 2.10E-63 | 137.8753444 |
| C8B | -2.101639007 | 5.547827694 | -20.88870097 | 5.39E-65 | 3.63E-63 | 137.3250462 |
| ANGPTL1 | -1.223138976 | 4.756429981 | -20.85812764 | 7.26E-65 | 4.86E-63 | 137.0287135 |
| CBX7 | -1.366735136 | 8.567450723 | -20.82059801 | 1.05E-64 | 6.96E-63 | 136.6649132 |
| CPB2 | -3.253560387 | 6.15095837 | -20.81039642 | 1.15E-64 | 7.63E-63 | 136.566014 |
| SELP | -1.967324772 | 6.53489884 | -20.79533031 | 1.34E-64 | 8.78E-63 | 136.4199495 |
| ETV4 | 2.429282349 | 6.566609492 | 20.78945868 | 1.42E-64 | 9.24E-63 | 136.3630226 |
| GHR | -2.287125638 | 6.283876793 | -20.77663796 | 1.60E-64 | 1.04E-62 | 136.2387186 |
| SLC19A3 | -2.336230006 | 6.326001029 | -20.7761211 | 1.61E-64 | 1.04E-62 | 136.2337072 |
| SRPK1 | 1.152564102 | 8.814380324 | 20.7506986 | 2.06E-64 | 1.32E-62 | 135.9872059 |
| ADCY4 | -1.466813803 | 7.807016298 | -20.73730355 | 2.35E-64 | 1.50E-62 | 135.8573165 |
| STX12 | -0.788233423 | 9.282504066 | -20.72726538 | 2.59E-64 | 1.64E-62 | 135.7599745 |
| KAL1 | -2.561075926 | 8.761031178 | -20.68910945 | 3.76E-64 | 2.37E-62 | 135.3899408 |
| SRPX | -2.243878865 | 8.348808598 | -20.62973405 | 6.70E-64 | 4.19E-62 | 134.8140337 |
| FAT3 | -1.679378669 | 5.24444763 | -20.62493266 | 7.02E-64 | 4.37E-62 | 134.7674583 |
| HSPB6 | -1.791272983 | 6.983308029 | -20.51132874 | 2.12E-63 | 1.31E-61 | 133.6652657 |
| AOC3 | -1.957893252 | 7.631213016 | -20.49123646 | 2.58E-63 | 1.59E-61 | 133.4702921 |
| RECK | -1.70216494 | 6.904972441 | -20.46856181 | 3.22E-63 | 1.97E-61 | 133.2502467 |
| GYPC | -1.226533032 | 7.970418505 | -20.403585 | 6.05E-63 | 3.68E-61 | 132.6196086 |
| C1QTNF7 | -1.71947081 | 5.311365702 | -20.37473215 | 8.02E-63 | 4.84E-61 | 132.3395413 |
| MMP11 | 3.114508534 | 7.461468529 | 20.37453503 | 8.03E-63 | 4.84E-61 | 132.3376278 |
| CENPF | 2.414282774 | 6.46114337 | 20.36760083 | 8.59E-63 | 5.14E-61 | 132.2703163 |
| CFD | -2.116945161 | 10.38957202 | -20.28972571 | 1.83E-62 | 1.09E-60 | 131.5142919 |
| ITM2A | -1.683062401 | 7.841747311 | -20.2372274 | 3.06E-62 | 1.81E-60 | 131.0045542 |
| ABI3BP | -2.438689818 | 8.809742771 | -20.23579464 | 3.10E-62 | 1.83E-60 | 130.9906419 |
| FBLN5 | -1.828822435 | 8.784676479 | -20.22910392 | 3.31E-62 | 1.94E-60 | 130.9256733 |
| PPP1R14A | -1.590357608 | 8.729482551 | -20.22305915 | 3.51E-62 | 2.05E-60 | 130.8669762 |
| MYH10 | -1.253532389 | 10.52896851 | -20.22061109 | 3.60E-62 | 2.08E-60 | 130.8432044 |
| GPX3 | -1.895766875 | 11.02153952 | -20.21978244 | 3.63E-62 | 2.09E-60 | 130.8351578 |
| SNX1 | -1.152424388 | 7.730076902 | -20.21011784 | 3.98E-62 | 2.28E-60 | 130.7413086 |
| ATIC | 0.899149741 | 10.00262382 | 20.17322662 | 5.71E-62 | 3.25E-60 | 130.3830552 |
| SLC39A11 | 1.139107979 | 8.728920428 | 20.16332992 | 6.29E-62 | 3.56E-60 | 130.2869431 |
| CCL23 | -1.765906727 | 6.304834173 | -20.14805223 | 7.30E-62 | 4.12E-60 | 130.1385696 |
| SFXN1 | 1.280156222 | 7.147583787 | 20.12313568 | 9.30E-62 | 5.22E-60 | 129.8965766 |
| WASF3 | -1.817290418 | 7.209670149 | -20.10994561 | 1.06E-61 | 5.90E-60 | 129.7684682 |
| COL10A1 | 2.884507832 | 6.865456 | 20.10220035 | 1.14E-61 | 6.33E-60 | 129.6932409 |
| PDIA4 | 1.360635057 | 9.158770239 | 20.08337072 | 1.37E-61 | 7.57E-60 | 129.5103505 |
| KIAA0101 | 1.593339659 | 8.057221356 | 20.05849127 | 1.75E-61 | 9.60E-60 | 129.2686893 |
| SESN1 | -1.331323661 | 8.560831646 | -20.04000271 | 2.09E-61 | 1.14E-59 | 129.089098 |
| GATA2 | -1.608522198 | 7.168470787 | -20.03386377 | 2.22E-61 | 1.21E-59 | 129.0294653 |
| MIF | 0.954181953 | 11.9045584 | 20.02531852 | 2.41E-61 | 1.30E-59 | 128.9464571 |
| CTNNAL1 | -1.530797559 | 8.593233681 | -20.02510859 | 2.42E-61 | 1.30E-59 | 128.9444178 |
| IGSF9 | 2.103358103 | 6.407563423 | 20.02498554 | 2.42E-61 | 1.30E-59 | 128.9432226 |
| DUOX1 | -1.811778688 | 6.652752875 | -20.00733832 | 2.87E-61 | 1.53E-59 | 128.7717944 |
| CGNL1 | -1.678339549 | 9.256997625 | -20.00446676 | 2.96E-61 | 1.57E-59 | 128.7438992 |
| ABHD6 | -1.01201018 | 7.001330649 | -19.99042702 | 3.39E-61 | 1.79E-59 | 128.6075106 |
| F10 | -1.578532396 | 6.415853713 | -19.98260771 | 3.66E-61 | 1.92E-59 | 128.5315489 |
| KLF4 | -1.883156169 | 7.536253452 | -19.97670368 | 3.87E-61 | 2.03E-59 | 128.4741929 |
| AHNAK | -1.305365302 | 8.732596415 | -19.97122269 | 4.09E-61 | 2.13E-59 | 128.4209461 |
| PEAR1 | -1.470046456 | 6.37995687 | -19.96334573 | 4.41E-61 | 2.29E-59 | 128.3444223 |
| PTPRM | -1.456316708 | 8.997316987 | -19.92640185 | 6.33E-61 | 3.26E-59 | 127.9855042 |
| LHFP | -1.658811762 | 9.423192691 | -19.91420202 | 7.13E-61 | 3.66E-59 | 127.8669761 |
| ICAM2 | -1.268375046 | 9.557796968 | -19.84254212 | 1.43E-60 | 7.32E-59 | 127.170722 |
| ALDH18A1 | 1.054814581 | 8.674239074 | 19.81724616 | 1.83E-60 | 9.32E-59 | 126.9249297 |
| MSRB3 | -1.648039394 | 6.8357875 | -19.80965975 | 1.97E-60 | 9.99E-59 | 126.8512137 |
| THBD | -1.933146506 | 7.150429838 | -19.79345758 | 2.31E-60 | 1.16E-58 | 126.6937778 |
| GPA33 | -1.901315598 | 6.198183348 | -19.77525498 | 2.76E-60 | 1.38E-58 | 126.5169005 |
| GNG11 | -2.296260046 | 9.449576303 | -19.7578025 | 3.27E-60 | 1.63E-58 | 126.3473092 |
| DENND2A | -1.320664812 | 7.512604008 | -19.7514971 | 3.48E-60 | 1.73E-58 | 126.2860369 |
| PTRF | -1.318839801 | 9.9631325 | -19.73436748 | 4.11E-60 | 2.03E-58 | 126.1195792 |
| SCARA5 | -2.533296527 | 6.385897654 | -19.72428427 | 4.54E-60 | 2.23E-58 | 126.0215939 |
| WFS1 | -1.180747143 | 9.384921681 | -19.71318017 | 5.06E-60 | 2.48E-58 | 125.913687 |
| PPAP2B | -1.346160041 | 9.062441316 | -19.70371685 | 5.55E-60 | 2.70E-58 | 125.8217241 |
| TNFRSF21 | 1.391784008 | 10.08507478 | 19.69751626 | 5.89E-60 | 2.86E-58 | 125.7614674 |
| PEBP4 | -3.423902095 | 8.210315029 | -19.68767831 | 6.49E-60 | 3.13E-58 | 125.6658625 |
| TMEM47 | -1.621197053 | 9.267262987 | -19.63156581 | 1.12E-59 | 5.39E-58 | 125.1205501 |
| FUT2 | 2.193098856 | 6.118480899 | 19.61394202 | 1.33E-59 | 6.37E-58 | 124.9492744 |
| PPM1F | -0.974725581 | 8.884110971 | -19.59024378 | 1.68E-59 | 7.99E-58 | 124.7189616 |
| GPC3 | -2.660160837 | 8.142164769 | -19.56980044 | 2.05E-59 | 9.71E-58 | 124.5202794 |
| SOX7 | -2.052047477 | 6.560885614 | -19.55529796 | 2.36E-59 | 1.11E-57 | 124.3793333 |
| TUBB1 | -1.488229829 | 4.886056215 | -19.46542771 | 5.66E-59 | 2.66E-57 | 123.5058889 |
| ARHGEF6 | -1.377497751 | 9.17496042 | -19.43912457 | 7.32E-59 | 3.43E-57 | 123.2502461 |
| ABCG2 | -1.573448234 | 5.414076239 | -19.43507001 | 7.61E-59 | 3.55E-57 | 123.2108393 |
| SLIT3 | -1.994524956 | 7.37609775 | -19.42090494 | 8.74E-59 | 4.06E-57 | 123.0731671 |
| PDE5A | -0.859774513 | 6.216899952 | -19.37136635 | 1.42E-58 | 6.54E-57 | 122.5916942 |
| ADARB1 | -1.115535445 | 7.211184896 | -19.37120909 | 1.42E-58 | 6.54E-57 | 122.5901658 |
| ANKS1A | -0.820485201 | 9.314288407 | -19.35449218 | 1.67E-58 | 7.66E-57 | 122.4276917 |
| GIMAP1 | -1.5735989 | 6.871985875 | -19.33827198 | 1.96E-58 | 8.93E-57 | 122.2700454 |
| PVRL4 | 1.653319139 | 6.22134533 | 19.2857273 | 3.27E-58 | 1.49E-56 | 121.7593597 |
| OLFML1 | -1.672167905 | 7.579548064 | -19.24743644 | 4.75E-58 | 2.15E-56 | 121.3872124 |
| WFDC1 | -1.920984787 | 7.419198867 | -19.23689137 | 5.26E-58 | 2.37E-56 | 121.2847261 |
| RAI2 | -1.383578027 | 6.841193489 | -19.22354167 | 5.99E-58 | 2.69E-56 | 121.1549827 |
| SHANK3 | -1.151576419 | 8.127421601 | -19.21610384 | 6.44E-58 | 2.88E-56 | 121.082696 |
| SOX17 | -1.920851175 | 6.38077712 | -19.21378314 | 6.59E-58 | 2.93E-56 | 121.0601417 |
| DAPK2 | -1.6332892 | 7.195532476 | -19.20165905 | 7.42E-58 | 3.29E-56 | 120.942311 |
| EPN3 | 1.496387441 | 6.001345106 | 19.14579474 | 1.28E-57 | 5.65E-56 | 120.3993909 |
| GPT2 | 1.766990387 | 7.653535713 | 19.11698004 | 1.69E-57 | 7.45E-56 | 120.1193614 |
| CDC20 | 2.298294664 | 7.795636157 | 19.10527462 | 1.90E-57 | 8.31E-56 | 120.0056064 |
| COL1A1 | 2.196078008 | 10.50762416 | 19.09929194 | 2.01E-57 | 8.78E-56 | 119.9474663 |
| FAM65A | -1.112914844 | 9.203656566 | -19.07887365 | 2.46E-57 | 1.07E-55 | 119.7490418 |
| FGR | -1.276157131 | 7.983350604 | -19.06756232 | 2.74E-57 | 1.19E-55 | 119.63912 |
| LMO2 | -1.489115093 | 8.142443436 | -19.0271437 | 4.07E-57 | 1.75E-55 | 119.2463473 |
| MSI2 | 1.05171168 | 6.438913306 | 19.01978686 | 4.37E-57 | 1.88E-55 | 119.174858 |
| EPB41L2 | -1.196579789 | 7.316981886 | -18.98000961 | 6.44E-57 | 2.75E-55 | 118.7883362 |
| RECQL4 | 1.594171683 | 6.244344548 | 18.9615967 | 7.70E-57 | 3.28E-55 | 118.6094208 |
| HBEGF | -1.937887691 | 9.135392021 | -18.93952161 | 9.55E-57 | 4.05E-55 | 118.3949258 |
| FRMD3 | -1.554598623 | 6.275100753 | -18.91482403 | 1.22E-56 | 5.14E-55 | 118.1549559 |
| MT1M | -3.092423646 | 8.001150146 | -18.88201326 | 1.67E-56 | 7.04E-55 | 117.8361678 |
| MARCO | -2.619989134 | 8.971168229 | -18.87013414 | 1.88E-56 | 7.88E-55 | 117.7207543 |
| EMR1 | -1.623477417 | 5.483129309 | -18.84555689 | 2.39E-56 | 9.97E-55 | 117.4819765 |
| PDK4 | -2.39292559 | 8.201809354 | -18.84022319 | 2.52E-56 | 1.05E-54 | 117.4301587 |
| STIL | 1.770333513 | 6.710841537 | 18.83467538 | 2.66E-56 | 1.10E-54 | 117.3762611 |
| PRICKLE2 | -1.211590842 | 8.188411037 | -18.83453565 | 2.66E-56 | 1.10E-54 | 117.3749035 |
| ANKRD29 | -2.34531239 | 7.339500455 | -18.8234621 | 2.96E-56 | 1.22E-54 | 117.2673243 |
| PTPN21 | -1.316031187 | 6.275877867 | -18.76421741 | 5.28E-56 | 2.16E-54 | 116.6917965 |
| MYOC | -1.340540284 | 4.979111843 | -18.75808857 | 5.60E-56 | 2.29E-54 | 116.6322616 |
| STX1A | 1.737995013 | 6.823145508 | 18.75662643 | 5.68E-56 | 2.31E-54 | 116.6180587 |
| ANLN | 2.379690214 | 6.507980654 | 18.74770031 | 6.20E-56 | 2.51E-54 | 116.5313528 |
| TMED3 | 0.782749647 | 10.76607883 | 18.73319647 | 7.14E-56 | 2.88E-54 | 116.3904694 |
| CBLC | 1.797204349 | 6.260728011 | 18.73178026 | 7.24E-56 | 2.91E-54 | 116.3767133 |
| MYADM | -1.141043907 | 8.673603622 | -18.71487006 | 8.54E-56 | 3.42E-54 | 116.2124609 |
| SEMA3G | -1.483199529 | 6.031203617 | -18.71382061 | 8.62E-56 | 3.44E-54 | 116.2022676 |
| PROM2 | 1.869996147 | 8.048264556 | 18.69839618 | 1.00E-55 | 3.98E-54 | 116.0524516 |
| WIF1 | -3.961629782 | 7.575646864 | -18.67092679 | 1.31E-55 | 5.19E-54 | 115.7856555 |
| AQP4 | -2.994587465 | 7.267791726 | -18.63903086 | 1.79E-55 | 7.05E-54 | 115.4758854 |
| TPX2 | 2.308143016 | 6.589492676 | 18.61961416 | 2.16E-55 | 8.49E-54 | 115.2873226 |
| PAICS | 1.078566593 | 8.118944827 | 18.60452188 | 2.50E-55 | 9.80E-54 | 115.1407613 |
| SLC2A1 | 2.228900319 | 8.48487266 | 18.59213929 | 2.82E-55 | 1.10E-53 | 115.0205175 |
| RAPGEF4 | -1.362152052 | 6.014439769 | -18.56593748 | 3.64E-55 | 1.42E-53 | 114.7660904 |
| PPARG | -1.53176616 | 6.979502261 | -18.55628126 | 4.00E-55 | 1.55E-53 | 114.6723297 |
| PPAP2C | 1.436215816 | 7.482593856 | 18.54477432 | 4.48E-55 | 1.73E-53 | 114.5606014 |
| MARVELD3 | 1.206599532 | 6.498867707 | 18.5438387 | 4.52E-55 | 1.74E-53 | 114.5515171 |
| NPNT | -1.868932971 | 7.930174234 | -18.53314155 | 5.02E-55 | 1.92E-53 | 114.4476546 |
| PTGER4 | -1.386221033 | 8.441249181 | -18.49867623 | 7.02E-55 | 2.68E-53 | 114.1130372 |
| CD34 | -1.006079492 | 7.523391168 | -18.49831899 | 7.04E-55 | 2.68E-53 | 114.109569 |
| HYAL1 | -1.428861427 | 7.075011918 | -18.47114022 | 9.18E-55 | 3.48E-53 | 113.8457163 |
| TAL1 | -0.855245271 | 5.518234878 | -18.44878261 | 1.14E-54 | 4.31E-53 | 113.6286819 |
| CDCA8 | 1.724239022 | 6.406662386 | 18.44520061 | 1.18E-54 | 4.45E-53 | 113.5939111 |
| CTHRC1 | 2.250424429 | 9.062608045 | 18.44276141 | 1.21E-54 | 4.54E-53 | 113.5702338 |
| PHLDA2 | 1.944795971 | 7.932013511 | 18.44075985 | 1.23E-54 | 4.61E-53 | 113.5508048 |
| BAI3 | -1.291707839 | 4.689115109 | -18.38877549 | 2.05E-54 | 7.63E-53 | 113.0462338 |
| HHIP | -1.902255304 | 5.688149098 | -18.36035991 | 2.70E-54 | 1.00E-52 | 112.7704582 |
| FLI1 | -1.362232882 | 6.172496755 | -18.35226459 | 2.92E-54 | 1.08E-52 | 112.6918966 |
| MEIS1 | -1.254661587 | 7.84108034 | -18.34998165 | 2.99E-54 | 1.10E-52 | 112.669742 |
| P4HB | 0.804955502 | 11.67694499 | 18.2799634 | 5.91E-54 | 2.17E-52 | 111.9903318 |
| CCNF | 1.364184515 | 7.160186923 | 18.25899171 | 7.24E-54 | 2.65E-52 | 111.7868659 |
| GAS6 | -1.169776813 | 8.626513856 | -18.25788775 | 7.32E-54 | 2.67E-52 | 111.7761558 |
| SERINC2 | 1.82591364 | 8.265718364 | 18.25308939 | 7.67E-54 | 2.79E-52 | 111.7296046 |
| CCDC85A | -0.764262016 | 5.092801564 | -18.24849254 | 8.02E-54 | 2.91E-52 | 111.685009 |
| KLF6 | -1.218391073 | 9.442459508 | -18.24365023 | 8.41E-54 | 3.04E-52 | 111.6380329 |
| HYOU1 | 1.024519371 | 9.164916463 | 18.22963768 | 9.64E-54 | 3.47E-52 | 111.502099 |
| TK1 | 1.880564284 | 7.840452984 | 18.22687454 | 9.90E-54 | 3.56E-52 | 111.4752949 |
| KCNA5 | -1.352066248 | 5.629492957 | -18.22547689 | 1.00E-53 | 3.59E-52 | 111.4617369 |
| ARHGEF16 | 1.331162107 | 6.944627997 | 18.19510204 | 1.35E-53 | 4.81E-52 | 111.1671011 |
| KIF20A | 2.035456622 | 6.379907029 | 18.19167895 | 1.39E-53 | 4.96E-52 | 111.1338991 |
| FRMD4A | -1.181606797 | 7.004954061 | -18.18909959 | 1.43E-53 | 5.07E-52 | 111.108881 |
| TRPV2 | -1.20469156 | 7.989955468 | -18.15429083 | 2.01E-53 | 7.09E-52 | 110.771282 |
| NDRG2 | -1.440869582 | 7.754352516 | -18.13054504 | 2.53E-53 | 8.90E-52 | 110.5410037 |
| ATP1A2 | -1.35777116 | 5.112542157 | -18.12973081 | 2.55E-53 | 8.94E-52 | 110.533108 |
| TTYH3 | 1.255260889 | 8.444385997 | 18.1287571 | 2.57E-53 | 8.99E-52 | 110.5236657 |
| INPP5A | -0.720042453 | 6.674341197 | -18.12853416 | 2.58E-53 | 8.99E-52 | 110.5215038 |
| GRASP | -1.408468444 | 7.534718989 | -18.11691348 | 2.89E-53 | 1.00E-51 | 110.4088188 |
| AURKB | 2.019344143 | 6.588061234 | 18.10878371 | 3.12E-53 | 1.08E-51 | 110.3299878 |
| VIM | -1.115674845 | 11.5584491 | -18.10813069 | 3.14E-53 | 1.09E-51 | 110.3236558 |
| ASPM | 2.343810006 | 6.109243566 | 18.1013746 | 3.36E-53 | 1.16E-51 | 110.2581469 |
| MAOB | -1.875389118 | 7.400067566 | -18.09994382 | 3.41E-53 | 1.17E-51 | 110.2442738 |
| PC | 1.055956699 | 6.089951657 | 18.09186592 | 3.68E-53 | 1.26E-51 | 110.1659509 |
| MCM4 | 1.434117297 | 7.461644535 | 18.08080665 | 4.10E-53 | 1.40E-51 | 110.0587246 |
| SCN7A | -1.499935803 | 5.493508934 | -18.05167122 | 5.45E-53 | 1.85E-51 | 109.7762611 |
| CRTAC1 | -2.556005804 | 8.162826556 | -18.03323516 | 6.52E-53 | 2.21E-51 | 109.5975433 |
| ERO1L | 1.578238336 | 8.92776662 | 18.02544437 | 7.03E-53 | 2.37E-51 | 109.5220239 |
| PRC1 | 1.787586209 | 7.761470077 | 18.02510772 | 7.05E-53 | 2.37E-51 | 109.5187606 |
| PLEK2 | 1.749214715 | 7.303555261 | 18.01669425 | 7.65E-53 | 2.57E-51 | 109.4372082 |
| NOSTRIN | -2.084680004 | 7.836834676 | -18.01008742 | 8.16E-53 | 2.73E-51 | 109.3731696 |
| C1orf162 | -1.602463907 | 9.893545848 | -17.99663195 | 9.30E-53 | 3.10E-51 | 109.242754 |
| SIRPB1 | -1.208401972 | 5.556076989 | -17.99473196 | 9.48E-53 | 3.15E-51 | 109.2243391 |
| SLC39A8 | -2.015723335 | 9.517108215 | -17.96276968 | 1.29E-52 | 4.29E-51 | 108.9145801 |
| MS4A2 | -2.06746474 | 6.131637106 | -17.9599877 | 1.33E-52 | 4.39E-51 | 108.8876208 |
| SPTBN2 | 1.386652853 | 5.998489729 | 17.95867937 | 1.35E-52 | 4.43E-51 | 108.8749423 |
| TIMP1 | 1.215935525 | 12.60851266 | 17.92484085 | 1.87E-52 | 6.14E-51 | 108.5470518 |
| S100A3 | -1.90397783 | 6.91063496 | -17.91886957 | 1.98E-52 | 6.49E-51 | 108.4891959 |
| NPM3 | 1.163495594 | 8.059597242 | 17.89471746 | 2.51E-52 | 8.18E-51 | 108.2552013 |
| IL33 | -1.938564634 | 6.17044413 | -17.88518807 | 2.75E-52 | 8.95E-51 | 108.162884 |
| TMPRSS4 | 2.53153583 | 6.827767997 | 17.88008513 | 2.89E-52 | 9.38E-51 | 108.1134501 |
| IRAK1 | 0.853635562 | 9.152551926 | 17.87008104 | 3.18E-52 | 1.03E-50 | 108.0165407 |
| STOM | -1.227930878 | 10.36577386 | -17.86856479 | 3.23E-52 | 1.04E-50 | 108.0018532 |
| PSAT1 | 1.479055242 | 6.017868816 | 17.83524965 | 4.47E-52 | 1.44E-50 | 107.6791643 |
| REEP1 | -1.778174144 | 6.181511495 | -17.8269497 | 4.84E-52 | 1.55E-50 | 107.5987791 |
| MAL | -1.649488314 | 7.778384255 | -17.82340311 | 5.01E-52 | 1.60E-50 | 107.5644313 |
| C19orf48 | 1.333765578 | 6.977175628 | 17.82220726 | 5.07E-52 | 1.62E-50 | 107.5528499 |
| IL3RA | -1.23794735 | 6.54832642 | -17.81389381 | 5.50E-52 | 1.75E-50 | 107.4723391 |
| TLR4 | -1.408003263 | 6.636096189 | -17.80867959 | 5.78E-52 | 1.83E-50 | 107.4218439 |
| TFAP2A | 1.476869159 | 6.116165176 | 17.79698359 | 6.48E-52 | 2.05E-50 | 107.3085833 |
| BDNF | -0.769391978 | 4.706861678 | -17.7758493 | 7.96E-52 | 2.51E-50 | 107.1039412 |
| LAD1 | 0.933640264 | 6.848890168 | 17.74317241 | 1.09E-51 | 3.43E-50 | 106.7875746 |
| MAP3K3 | -0.613453597 | 7.05832329 | -17.73351457 | 1.20E-51 | 3.76E-50 | 106.6940805 |
| GFRA2 | -0.951092245 | 6.688800755 | -17.73137673 | 1.23E-51 | 3.83E-50 | 106.6733854 |
| NDST1 | -1.198052472 | 7.572366524 | -17.72872041 | 1.26E-51 | 3.92E-50 | 106.6476716 |
| CCNB1 | 2.004362328 | 6.152616019 | 17.72834786 | 1.26E-51 | 3.92E-50 | 106.6440653 |
| ENG | -1.134300035 | 9.457000194 | -17.72176044 | 1.35E-51 | 4.17E-50 | 106.5802992 |
| KIF26B | 1.530111984 | 5.665779218 | 17.69804783 | 1.69E-51 | 5.23E-50 | 106.350779 |
| DPYSL2 | -1.269669313 | 11.36884866 | -17.66845537 | 2.26E-51 | 6.95E-50 | 106.0643859 |
| CD300C | -1.577857663 | 6.669944822 | -17.65659085 | 2.54E-51 | 7.78E-50 | 105.9495747 |
| KIAA1324L | -1.916875934 | 7.847345338 | -17.65638149 | 2.54E-51 | 7.78E-50 | 105.9475488 |
| CCNB2 | 2.101254155 | 7.203460367 | 17.62075912 | 3.59E-51 | 1.10E-49 | 105.6028803 |
| CYBRD1 | -1.57791126 | 10.10262179 | -17.58185296 | 5.24E-51 | 1.59E-49 | 105.2265155 |
| MELK | 2.16784943 | 6.359668673 | 17.55726291 | 6.65E-51 | 2.02E-49 | 104.9886819 |
| VSIG4 | -2.158406526 | 9.108614386 | -17.55399735 | 6.86E-51 | 2.08E-49 | 104.9571 |
| CELSR3 | 1.83000387 | 6.529731686 | 17.55270651 | 6.95E-51 | 2.10E-49 | 104.9446162 |
| SEPP1 | -1.434875152 | 8.224684779 | -17.54191321 | 7.72E-51 | 2.32E-49 | 104.840237 |
| NDRG4 | -1.04767875 | 5.959518338 | -17.53462742 | 8.28E-51 | 2.49E-49 | 104.7697818 |
| FAM83A | 2.29290491 | 6.26524466 | 17.51159038 | 1.04E-50 | 3.10E-49 | 104.5470277 |
| LAMP3 | -2.29199328 | 9.93073534 | -17.50224938 | 1.13E-50 | 3.38E-49 | 104.4567145 |
| GLDN | -2.094264368 | 7.263330112 | -17.49184303 | 1.25E-50 | 3.73E-49 | 104.3561067 |
| PLSCR4 | -1.497011278 | 8.524209279 | -17.45336152 | 1.82E-50 | 5.41E-49 | 103.9841244 |
| CD52 | -1.868100626 | 10.2026275 | -17.44372275 | 2.00E-50 | 5.92E-49 | 103.8909645 |
| CRIM1 | -1.350165896 | 7.55664917 | -17.44354468 | 2.00E-50 | 5.92E-49 | 103.8892435 |
| SGPL1 | 1.02484883 | 8.234566362 | 17.44085592 | 2.06E-50 | 6.06E-49 | 103.8632573 |
| UBE2C | 1.787826129 | 7.002680726 | 17.43425071 | 2.19E-50 | 6.44E-49 | 103.7994214 |
| C1orf198 | -1.099185361 | 9.914023069 | -17.42100921 | 2.49E-50 | 7.30E-49 | 103.6714569 |
| PRX | -0.974080498 | 6.088805699 | -17.41336357 | 2.69E-50 | 7.84E-49 | 103.597575 |
| STX2 | -0.887826496 | 8.343733793 | -17.40909608 | 2.80E-50 | 8.15E-49 | 103.5563386 |
| CDT1 | 1.636295713 | 6.10729042 | 17.40581415 | 2.89E-50 | 8.39E-49 | 103.5246263 |
| CSTF2 | 0.906382433 | 7.79332446 | 17.38043125 | 3.70E-50 | 1.07E-48 | 103.279381 |
| ANKRD22 | 2.266311374 | 6.994565556 | 17.38007971 | 3.71E-50 | 1.07E-48 | 103.2759848 |
| CACNA2D2 | -2.264763741 | 7.8009815 | -17.37376346 | 3.94E-50 | 1.14E-48 | 103.2149645 |
| MDK | 1.166614296 | 7.656218426 | 17.35499505 | 4.73E-50 | 1.36E-48 | 103.0336602 |
| KRT4 | -1.937991842 | 6.12322538 | -17.34470917 | 5.23E-50 | 1.50E-48 | 102.934307 |
| HSD17B6 | -2.599470847 | 8.327603492 | -17.32753257 | 6.17E-50 | 1.76E-48 | 102.7684097 |
| HMGCLL1 | -0.773376739 | 4.494887082 | -17.32096512 | 6.58E-50 | 1.88E-48 | 102.7049839 |
| SOCS2 | -1.93761358 | 8.141236436 | -17.31001055 | 7.32E-50 | 2.08E-48 | 102.5991953 |
| BAIAP2L1 | 1.324716488 | 8.950088029 | 17.30287778 | 7.84E-50 | 2.22E-48 | 102.530318 |
| TROAP | 1.61754586 | 6.015212069 | 17.30123671 | 7.97E-50 | 2.25E-48 | 102.5144715 |
| PHACTR1 | -1.213936124 | 6.44201166 | -17.29724998 | 8.28E-50 | 2.34E-48 | 102.4759756 |
| KIF2C | 1.785998154 | 6.172970649 | 17.23640561 | 1.49E-49 | 4.20E-48 | 101.8885891 |
| ALOX5 | -1.003232505 | 7.67139441 | -17.23615047 | 1.50E-49 | 4.20E-48 | 101.8861265 |
| SH3BP5 | -0.749927454 | 6.496966904 | -17.21426221 | 1.85E-49 | 5.18E-48 | 101.6748787 |
| CEP55 | 1.975302503 | 6.61296992 | 17.19928587 | 2.14E-49 | 5.97E-48 | 101.5303575 |
| HMMR | 1.913296841 | 5.792317856 | 17.19539541 | 2.22E-49 | 6.18E-48 | 101.4928172 |
| GMFG | -1.300867459 | 9.223330739 | -17.1904624 | 2.33E-49 | 6.46E-48 | 101.4452184 |
| RALGPS2 | 1.264446548 | 6.071521481 | 17.19019979 | 2.34E-49 | 6.46E-48 | 101.4426846 |
| ZBED2 | -2.103546196 | 6.61837834 | -17.19003425 | 2.34E-49 | 6.46E-48 | 101.4410873 |
| OGN | -1.96403009 | 5.413684809 | -17.18849914 | 2.37E-49 | 6.54E-48 | 101.4262754 |
| PKMYT1 | 1.141543696 | 5.896824072 | 17.18733889 | 2.40E-49 | 6.60E-48 | 101.4150805 |
| CLEC12A | -1.504336885 | 5.773616543 | -17.17967039 | 2.59E-49 | 7.09E-48 | 101.3410917 |
| GFOD1 | -1.180263316 | 7.637736404 | -17.17295019 | 2.76E-49 | 7.55E-48 | 101.2762558 |
| PAK1 | 0.805212398 | 8.194688311 | 17.16323259 | 3.03E-49 | 8.27E-48 | 101.1825068 |
| VGLL3 | -1.957053769 | 6.854191997 | -17.16047721 | 3.11E-49 | 8.47E-48 | 101.1559258 |
| ADAMTS1 | -2.04673739 | 8.297623543 | -17.15617377 | 3.25E-49 | 8.81E-48 | 101.114412 |
| FAM46B | -1.433474331 | 7.126178638 | -17.15218455 | 3.38E-49 | 9.14E-48 | 101.0759304 |
| CDCA5 | 1.744123297 | 7.136309774 | 17.13792464 | 3.87E-49 | 1.05E-47 | 100.9383827 |
| GIMAP4 | -1.424727535 | 9.142320112 | -17.13055669 | 4.16E-49 | 1.12E-47 | 100.8673187 |
| FGF2 | -0.932997576 | 5.737354457 | -17.12989453 | 4.19E-49 | 1.13E-47 | 100.8609323 |
| TM6SF1 | -1.57833517 | 7.114146513 | -17.12154276 | 4.54E-49 | 1.22E-47 | 100.7803844 |
| RNF125 | -1.324646982 | 6.563140801 | -17.1032227 | 5.42E-49 | 1.45E-47 | 100.6037152 |
| SEMA5A | -1.641971745 | 7.355799481 | -17.09631253 | 5.80E-49 | 1.55E-47 | 100.5370833 |
| MRPL17 | 0.666705602 | 9.809785896 | 17.07722425 | 6.97E-49 | 1.85E-47 | 100.3530404 |
| CYP4B1 | -3.418270671 | 8.718554795 | -17.05751347 | 8.44E-49 | 2.24E-47 | 100.163023 |
| CHRDL1 | -2.004726854 | 6.500943449 | -17.05589338 | 8.57E-49 | 2.27E-47 | 100.1474061 |
| SALL4 | 1.795243358 | 5.702781638 | 17.04660189 | 9.38E-49 | 2.48E-47 | 100.0578444 |
| MCM2 | 1.478369225 | 7.759155332 | 17.04454204 | 9.57E-49 | 2.52E-47 | 100.0379901 |
| KLF9 | -1.288279588 | 9.595447316 | -17.04162024 | 9.84E-49 | 2.59E-47 | 100.0098283 |
| VEPH1 | -2.042033539 | 6.131648261 | -17.03589769 | 1.04E-48 | 2.73E-47 | 99.95467322 |
| PKIG | -0.928407471 | 6.90832083 | -17.01893885 | 1.23E-48 | 3.20E-47 | 99.79123454 |
| SAMD10 | 0.998402539 | 6.052943758 | 17.0186827 | 1.23E-48 | 3.20E-47 | 99.7887661 |
| COASY | 0.635359535 | 8.978781125 | 17.00857804 | 1.35E-48 | 3.53E-47 | 99.69139398 |
| HMGB3 | 2.181844681 | 8.47426683 | 17.00459301 | 1.41E-48 | 3.66E-47 | 99.6529949 |
| DIXDC1 | -0.674729246 | 6.384749633 | -16.99550776 | 1.54E-48 | 3.98E-47 | 99.56545527 |
| ST14 | 1.308345143 | 8.990133011 | 16.98133068 | 1.76E-48 | 4.56E-47 | 99.42886651 |
| TUBB6 | -1.434341545 | 8.840300606 | -16.97793725 | 1.82E-48 | 4.70E-47 | 99.39617476 |
| ABCB1 | -1.476025593 | 5.465855423 | -16.97656335 | 1.85E-48 | 4.75E-47 | 99.38293911 |
| OLR1 | -2.184789517 | 9.47944412 | -16.9644847 | 2.07E-48 | 5.32E-47 | 99.26658383 |
| EFEMP1 | -1.589209479 | 8.755038912 | -16.96273931 | 2.11E-48 | 5.40E-47 | 99.24977117 |
| KDELR2 | 0.919025774 | 10.4023585 | 16.95044518 | 2.38E-48 | 6.07E-47 | 99.13135317 |
| GAPDH | 0.843085908 | 12.61438398 | 16.93964565 | 2.64E-48 | 6.72E-47 | 99.02734075 |
| AURKA | 1.787754181 | 7.253152777 | 16.93472152 | 2.77E-48 | 7.03E-47 | 98.97991843 |
| LPL | -2.405482983 | 8.657595564 | -16.91974565 | 3.20E-48 | 8.11E-47 | 98.83570339 |
| RASAL1 | 1.331725347 | 5.931814457 | 16.90698519 | 3.62E-48 | 9.15E-47 | 98.71283604 |
| DOK2 | -1.019457791 | 6.654059846 | -16.89570182 | 4.03E-48 | 1.02E-46 | 98.60420178 |
| SCARA3 | -1.196931825 | 6.37069187 | -16.89360957 | 4.12E-48 | 1.04E-46 | 98.58405902 |
| TSPAN7 | -2.56839472 | 8.622236521 | -16.88235929 | 4.59E-48 | 1.15E-46 | 98.47575521 |
| ST6GALNAC5 | -1.520981676 | 6.601928997 | -16.87432546 | 4.96E-48 | 1.24E-46 | 98.39842141 |
| TPPP | -1.441052267 | 6.212289309 | -16.84363599 | 6.67E-48 | 1.67E-46 | 98.10305129 |
| LDHA | 0.857879483 | 12.45785006 | 16.84243236 | 6.75E-48 | 1.68E-46 | 98.09146855 |
| ODF3L1 | -0.837525794 | 5.001472553 | -16.83619892 | 7.17E-48 | 1.78E-46 | 98.03148479 |
| MYL9 | -1.382876226 | 9.17032846 | -16.83403992 | 7.32E-48 | 1.82E-46 | 98.01070964 |
| MRPL12 | 0.968850175 | 8.916181088 | 16.81968281 | 8.41E-48 | 2.08E-46 | 97.872567 |
| DOCK11 | -1.203228171 | 8.660313726 | -16.81762046 | 8.58E-48 | 2.12E-46 | 97.8527246 |
| TMEM132A | 1.313869959 | 7.084217495 | 16.80932367 | 9.29E-48 | 2.29E-46 | 97.77290279 |
| ECT2 | 1.578605711 | 7.457142519 | 16.80335509 | 9.84E-48 | 2.42E-46 | 97.71548362 |
| FOXM1 | 1.910506978 | 6.156343186 | 16.79875913 | 1.03E-47 | 2.53E-46 | 97.67127147 |
| FCN1 | -1.800224112 | 8.478123891 | -16.79602642 | 1.06E-47 | 2.59E-46 | 97.64498413 |
| BIRC5 | 1.595642429 | 5.796239109 | 16.79375806 | 1.08E-47 | 2.64E-46 | 97.62316411 |
| SPATS2 | 0.936747786 | 7.388642952 | 16.77329886 | 1.32E-47 | 3.21E-46 | 97.42638023 |
| ATAD3A | 0.998948436 | 7.857831867 | 16.7721158 | 1.33E-47 | 3.24E-46 | 97.41500219 |
| KIFC1 | 1.620112779 | 6.725179814 | 16.75416793 | 1.58E-47 | 3.84E-46 | 97.24240347 |
| MSR1 | -1.598113719 | 6.613884513 | -16.74573132 | 1.72E-47 | 4.16E-46 | 97.16128069 |
| ZHX3 | -0.848678328 | 7.09632942 | -16.74102877 | 1.80E-47 | 4.34E-46 | 97.11606561 |
| CNTN6 | -1.326361575 | 5.857854309 | -16.73152041 | 1.97E-47 | 4.75E-46 | 97.02464824 |
| PLEKHH2 | -1.630815623 | 7.104448189 | -16.72888093 | 2.02E-47 | 4.86E-46 | 96.99927254 |
| KIF11 | 1.774133005 | 6.092766864 | 16.72573535 | 2.08E-47 | 5.00E-46 | 96.96903207 |
| USHBP1 | -0.891321778 | 5.61071825 | -16.71463465 | 2.32E-47 | 5.55E-46 | 96.86232043 |
| MAP3K8 | -1.328812777 | 8.011168492 | -16.70808706 | 2.47E-47 | 5.90E-46 | 96.79938301 |
| PLA2G1B | -2.928142266 | 7.637729463 | -16.68553319 | 3.07E-47 | 7.32E-46 | 96.58261642 |
| PAPSS2 | -1.296910177 | 9.827083824 | -16.68370036 | 3.12E-47 | 7.43E-46 | 96.5650029 |
| PCOLCE2 | -2.43230208 | 7.448733814 | -16.66779206 | 3.64E-47 | 8.64E-46 | 96.4121362 |
| COX4I2 | -1.433009321 | 7.32469596 | -16.66534861 | 3.73E-47 | 8.83E-46 | 96.3886584 |
| CSRP1 | -0.726723327 | 11.19157288 | -16.66282874 | 3.82E-47 | 9.03E-46 | 96.3644469 |
| IL18R1 | -1.595668019 | 6.276536247 | -16.62923453 | 5.28E-47 | 1.25E-45 | 96.04171908 |
| QKI | -0.924909341 | 6.850914785 | -16.61456933 | 6.09E-47 | 1.43E-45 | 95.90086684 |
| CHPF | 1.280836532 | 8.825394644 | 16.60856435 | 6.45E-47 | 1.51E-45 | 95.84319742 |
| ACACB | -1.05185564 | 7.772185077 | -16.6023651 | 6.84E-47 | 1.60E-45 | 95.78366565 |
| CCNA2 | 1.770596489 | 6.472418019 | 16.60167981 | 6.89E-47 | 1.61E-45 | 95.77708497 |
| PRELP | -1.42803901 | 7.109418441 | -16.59389565 | 7.43E-47 | 1.73E-45 | 95.70233857 |
| ABCA6 | -1.384519846 | 6.480562069 | -16.5925314 | 7.53E-47 | 1.75E-45 | 95.68923912 |
| KIF4A | 1.85197156 | 5.546209266 | 16.58574065 | 8.03E-47 | 1.87E-45 | 95.62403702 |
| TIMP3 | -1.707799507 | 10.2026961 | -16.57877416 | 8.59E-47 | 1.99E-45 | 95.55715195 |
| TTK | 2.08048895 | 5.537632572 | 16.56707132 | 9.62E-47 | 2.22E-45 | 95.44480314 |
| RETN | -2.066037829 | 6.795256428 | -16.56573143 | 9.74E-47 | 2.25E-45 | 95.43194078 |
| CDH3 | 2.342937142 | 8.264230245 | 16.55339471 | 1.10E-46 | 2.53E-45 | 95.31352168 |
| ID2 | -1.280943989 | 9.295287 | -16.54687756 | 1.17E-46 | 2.68E-45 | 95.25096969 |
| STAC | -1.419096502 | 6.42740967 | -16.54017723 | 1.25E-46 | 2.86E-45 | 95.18666353 |
| ENO1 | 0.816231627 | 12.58601989 | 16.53730986 | 1.28E-46 | 2.93E-45 | 95.15914539 |
| FGF11 | 1.616118522 | 6.00401166 | 16.52901342 | 1.39E-46 | 3.17E-45 | 95.07952859 |
| EZH2 | 1.426375315 | 6.382966178 | 16.52058336 | 1.51E-46 | 3.43E-45 | 94.99863605 |
| PMP22 | -1.501441207 | 8.885114064 | -16.51911404 | 1.53E-46 | 3.47E-45 | 94.98453747 |
| LRRK2 | -2.06697972 | 7.427913992 | -16.51697435 | 1.56E-46 | 3.54E-45 | 94.96400697 |
| MYH11 | -1.727507125 | 8.444468816 | -16.50842824 | 1.69E-46 | 3.83E-45 | 94.88201038 |
| RASGRP2 | -0.908517487 | 6.16694887 | -16.50670115 | 1.72E-46 | 3.88E-45 | 94.86544052 |
| CDCA3 | 1.652257712 | 5.935202625 | 16.50656551 | 1.72E-46 | 3.88E-45 | 94.86413912 |
| SSTR1 | -1.399335382 | 5.391236 | -16.48880606 | 2.04E-46 | 4.60E-45 | 94.69376906 |
| SPC24 | 1.444911414 | 6.546995934 | 16.48682666 | 2.08E-46 | 4.68E-45 | 94.6747821 |
| ARMCX1 | -1.340480553 | 7.158879859 | -16.47430019 | 2.35E-46 | 5.27E-45 | 94.55463326 |
| UBL3 | -0.972618176 | 9.196609207 | -16.46834142 | 2.49E-46 | 5.56E-45 | 94.49748429 |
| TRAP1 | 0.736445348 | 8.625134197 | 16.45794006 | 2.75E-46 | 6.13E-45 | 94.39773578 |
| SLC35F2 | 1.270999463 | 8.066735229 | 16.4578617 | 2.75E-46 | 6.13E-45 | 94.3969844 |
| LIFR | -1.891600441 | 7.384916989 | -16.43739338 | 3.35E-46 | 7.45E-45 | 94.20072445 |
| EPHB2 | 1.194026424 | 6.269497859 | 16.41890323 | 4.01E-46 | 8.88E-45 | 94.02346663 |
| JAM3 | -1.050898394 | 8.956065888 | -16.41872534 | 4.01E-46 | 8.88E-45 | 94.02176143 |
| C1orf115 | -1.33417352 | 8.463576556 | -16.41107741 | 4.32E-46 | 9.54E-45 | 93.94845354 |
| LMOD1 | -1.347127366 | 6.669038548 | -16.40425142 | 4.61E-46 | 1.02E-44 | 93.88302895 |
| GJA4 | -1.227817688 | 7.664814811 | -16.39248787 | 5.17E-46 | 1.14E-44 | 93.77029042 |
| CD97 | -1.018978122 | 8.659628431 | -16.38962224 | 5.31E-46 | 1.17E-44 | 93.74282904 |
| LSR | 0.984875917 | 7.524880548 | 16.38694098 | 5.45E-46 | 1.19E-44 | 93.71713525 |
| F8 | -1.111392924 | 6.129496569 | -16.38388115 | 5.61E-46 | 1.23E-44 | 93.68781453 |
| XPR1 | 1.36123139 | 9.371985418 | 16.38277452 | 5.67E-46 | 1.24E-44 | 93.67721047 |
| PPAT | 1.226468668 | 7.056223484 | 16.37563681 | 6.08E-46 | 1.32E-44 | 93.6088181 |
| GARS | 0.744451791 | 10.21060656 | 16.37191409 | 6.30E-46 | 1.37E-44 | 93.57314957 |
| SRPRB | 0.719391103 | 9.611869069 | 16.35542508 | 7.38E-46 | 1.60E-44 | 93.41517958 |
| SFN | 1.838539207 | 9.553303702 | 16.35242159 | 7.60E-46 | 1.64E-44 | 93.38640815 |
| KIAA1462 | -1.017941364 | 5.977563125 | -16.34725771 | 7.98E-46 | 1.72E-44 | 93.3369436 |
| CXCL2 | -2.155482117 | 8.451702686 | -16.34236274 | 8.37E-46 | 1.80E-44 | 93.29005739 |
| GFPT1 | 1.014360562 | 9.208761168 | 16.34157611 | 8.43E-46 | 1.81E-44 | 93.28252294 |
| HYAL2 | -0.624272589 | 7.301907019 | -16.34105861 | 8.47E-46 | 1.82E-44 | 93.27756627 |
| SERPING1 | -0.895873296 | 9.512919237 | -16.34047112 | 8.52E-46 | 1.82E-44 | 93.27193926 |
| STARD13 | -0.97334189 | 6.328557082 | -16.3299783 | 9.42E-46 | 2.01E-44 | 93.17144439 |
| RBMS3 | -1.197591894 | 6.688304816 | -16.32801176 | 9.60E-46 | 2.05E-44 | 93.15261109 |
| HMGA1 | 0.72549792 | 6.87610392 | 16.32346029 | 1.00E-45 | 2.14E-44 | 93.1090238 |
| MYO7A | 1.137162006 | 6.351593295 | 16.32118877 | 1.03E-45 | 2.18E-44 | 93.08727127 |
| ITLN1 | -3.106116341 | 6.128472931 | -16.31905369 | 1.05E-45 | 2.22E-44 | 93.06682577 |
| GJB2 | 2.65035537 | 6.464939202 | 16.30536306 | 1.19E-45 | 2.53E-44 | 92.93573558 |
| LILRA2 | -1.637214419 | 6.21641991 | -16.29981446 | 1.26E-45 | 2.66E-44 | 92.88261209 |
| WISP2 | -1.881470505 | 7.828505008 | -16.29618489 | 1.30E-45 | 2.75E-44 | 92.8478636 |
| PHACTR2 | -1.227040676 | 8.692087322 | -16.28757798 | 1.42E-45 | 2.98E-44 | 92.7654686 |
| MASP1 | -0.916574035 | 5.037223713 | -16.28481863 | 1.45E-45 | 3.06E-44 | 92.73905459 |
| TSTA3 | 1.068057101 | 8.642152074 | 16.282895 | 1.48E-45 | 3.11E-44 | 92.72064104 |
| KIF14 | 1.641669464 | 5.524069981 | 16.2760472 | 1.58E-45 | 3.31E-44 | 92.65509496 |
| TINF2 | -0.632892025 | 9.520209479 | -16.25828503 | 1.88E-45 | 3.92E-44 | 92.48510048 |
| SERINC1 | -0.896107438 | 10.07384176 | -16.25734853 | 1.89E-45 | 3.95E-44 | 92.47613849 |
| ZWINT | 1.13656946 | 6.349034971 | 16.25423516 | 1.95E-45 | 4.06E-44 | 92.44634531 |
| MUSTN1 | -1.342774145 | 6.851483316 | -16.2535499 | 1.96E-45 | 4.08E-44 | 92.43978796 |
| TBX2 | -1.251495719 | 8.531305668 | -16.25004475 | 2.03E-45 | 4.21E-44 | 92.40624717 |
| OSR1 | -1.495609736 | 7.228256566 | -16.2417049 | 2.20E-45 | 4.55E-44 | 92.32644818 |
| SHMT2 | 0.909664061 | 9.563598194 | 16.24122442 | 2.21E-45 | 4.56E-44 | 92.32185098 |
| MME | -1.485331257 | 6.099408375 | -16.22986763 | 2.47E-45 | 5.08E-44 | 92.21319683 |
| IKBKE | 1.028942627 | 7.004086644 | 16.22905254 | 2.49E-45 | 5.11E-44 | 92.20539911 |
| POLQ | 1.567841814 | 5.545527981 | 16.22891961 | 2.49E-45 | 5.11E-44 | 92.2041275 |
| PODXL2 | 1.920320313 | 7.285191864 | 16.2172379 | 2.78E-45 | 5.70E-44 | 92.09237999 |
| GCNT3 | 3.025042488 | 7.271978184 | 16.20167998 | 3.23E-45 | 6.61E-44 | 91.94357475 |
| CAD | 0.942528092 | 7.526394426 | 16.19241227 | 3.53E-45 | 7.21E-44 | 91.85494497 |
| BZW2 | 0.97460918 | 9.654394832 | 16.18443268 | 3.82E-45 | 7.77E-44 | 91.77864106 |
| KLF13 | -0.945234867 | 9.184094106 | -16.17060984 | 4.36E-45 | 8.86E-44 | 91.64647781 |
| C1orf21 | -1.518954712 | 7.843731947 | -16.16273212 | 4.70E-45 | 9.53E-44 | 91.57116637 |
| ARHGEF15 | -0.867985793 | 6.324763239 | -16.15336407 | 5.14E-45 | 1.04E-43 | 91.48161581 |
| FRMD4B | -0.774144937 | 6.364564652 | -16.10211124 | 8.41E-45 | 1.70E-43 | 90.99185034 |
| KLF2 | -1.150627625 | 10.17167909 | -16.10050621 | 8.54E-45 | 1.72E-43 | 90.97651758 |
| SH3D19 | -0.873611044 | 8.548296516 | -16.09652109 | 8.87E-45 | 1.79E-43 | 90.93844889 |
| TCF3 | 0.881704664 | 8.023554968 | 16.08739041 | 9.68E-45 | 1.95E-43 | 90.85123297 |
| AMICA1 | -1.474119166 | 8.647324096 | -16.08118864 | 1.03E-44 | 2.06E-43 | 90.79199907 |
| KLF11 | -0.721166808 | 8.032857146 | -16.07432954 | 1.10E-44 | 2.20E-43 | 90.72649186 |
| CHEK1 | 1.555843806 | 5.98781234 | 16.00703968 | 2.09E-44 | 4.18E-43 | 90.0841244 |
| PGM2L1 | 1.245820162 | 7.240463128 | 16.00246056 | 2.18E-44 | 4.36E-43 | 90.04042939 |
| CNN1 | -1.591755564 | 7.489308588 | -15.99193868 | 2.42E-44 | 4.81E-43 | 89.94003614 |
| PLA2G4F | -1.759415935 | 7.198167907 | -15.98322437 | 2.63E-44 | 5.22E-43 | 89.85689925 |
| HOOK1 | 1.495025773 | 7.831785769 | 15.97626855 | 2.81E-44 | 5.57E-43 | 89.79054501 |
| DUSP1 | -1.399957936 | 11.60807519 | -15.97581404 | 2.82E-44 | 5.59E-43 | 89.78620949 |
| KLRF1 | -1.307870338 | 4.920339138 | -15.97184926 | 2.93E-44 | 5.79E-43 | 89.74839067 |
| ST6GALNAC6 | -0.859926829 | 8.821537875 | -15.95887866 | 3.32E-44 | 6.55E-43 | 89.62468057 |
| LIMK1 | 0.813748864 | 6.291599019 | 15.94600537 | 3.75E-44 | 7.39E-43 | 89.50191786 |
| CCT3 | 0.742216649 | 9.94174837 | 15.93153953 | 4.31E-44 | 8.47E-43 | 89.36399111 |
| MNDA | -1.922157157 | 7.018531726 | -15.921786 | 4.73E-44 | 9.28E-43 | 89.27100833 |
| SLC25A10 | 1.227440657 | 7.26438959 | 15.91320243 | 5.14E-44 | 1.01E-42 | 89.18918834 |
| SLC1A1 | -1.907937668 | 6.948644088 | -15.91269138 | 5.16E-44 | 1.01E-42 | 89.18431722 |
| NEK2 | 1.682327089 | 5.600155864 | 15.8994168 | 5.86E-44 | 1.14E-42 | 89.05779953 |
| KNTC1 | 1.25491963 | 7.303113668 | 15.89085461 | 6.36E-44 | 1.24E-42 | 88.97620582 |
| RERG | -1.619700045 | 7.168581114 | -15.88971813 | 6.43E-44 | 1.25E-42 | 88.96537635 |
| GNAQ | -1.364740509 | 8.145765947 | -15.88568535 | 6.68E-44 | 1.30E-42 | 88.9269493 |
| ETS2 | -1.133917494 | 9.986447144 | -15.85351885 | 9.09E-44 | 1.76E-42 | 88.62051499 |
| BOP1 | 0.880713457 | 8.777624194 | 15.8526975 | 9.16E-44 | 1.77E-42 | 88.61269199 |
| SOX4 | 1.069483491 | 9.077169511 | 15.84779859 | 9.60E-44 | 1.85E-42 | 88.56603381 |
| THBS2 | 1.85497246 | 9.740621234 | 15.84578086 | 9.79E-44 | 1.88E-42 | 88.54681742 |
| BUB1 | 1.727022798 | 5.905293798 | 15.8457552 | 9.79E-44 | 1.88E-42 | 88.546573 |
| MRAS | -0.942746346 | 6.684291093 | -15.84406119 | 9.95E-44 | 1.91E-42 | 88.53044003 |
| SRD5A1 | 1.295108107 | 7.236103668 | 15.83677285 | 1.07E-43 | 2.05E-42 | 88.46103313 |
| LYPLA2 | 0.647736195 | 8.294953758 | 15.82944706 | 1.14E-43 | 2.19E-42 | 88.39127598 |
| KCNAB1 | -0.793001459 | 4.909607649 | -15.82450928 | 1.20E-43 | 2.29E-42 | 88.34426147 |
| FRMD5 | 0.882969028 | 5.147281074 | 15.81945664 | 1.26E-43 | 2.40E-42 | 88.29615633 |
| SPAG4 | 1.521601524 | 6.737991949 | 15.81652723 | 1.29E-43 | 2.46E-42 | 88.26826744 |
| PODXL | -0.845037902 | 8.153136819 | -15.80711028 | 1.42E-43 | 2.69E-42 | 88.17862232 |
| PDLIM1 | -0.84832997 | 10.74826599 | -15.78427552 | 1.76E-43 | 3.34E-42 | 87.96129066 |
| CD33 | -1.487200971 | 7.148454691 | -15.77198848 | 1.98E-43 | 3.75E-42 | 87.84437423 |
| ADAM8 | 1.521953782 | 7.582216109 | 15.76495299 | 2.12E-43 | 4.01E-42 | 87.77743692 |
| SPDEF | 2.137537196 | 7.095300729 | 15.76084747 | 2.20E-43 | 4.16E-42 | 87.73837882 |
| EXO1 | 1.095955292 | 5.264390037 | 15.74364051 | 2.60E-43 | 4.89E-42 | 87.57470233 |
| WDR46 | 0.656006422 | 8.35451562 | 15.73731337 | 2.76E-43 | 5.19E-42 | 87.51452646 |
| ITGA11 | 1.673721569 | 6.39185783 | 15.66750332 | 5.37E-43 | 1.01E-41 | 86.85091396 |
| ANXA3 | -2.050227652 | 8.56306492 | -15.6659034 | 5.46E-43 | 1.02E-41 | 86.8357124 |
| KCNK3 | -1.626447508 | 7.202472854 | -15.65915027 | 5.82E-43 | 1.09E-41 | 86.77155153 |
| CRABP2 | 2.623812836 | 7.957900316 | 15.65323364 | 6.16E-43 | 1.15E-41 | 86.71534301 |
| SLC31A2 | -1.34089997 | 8.946199048 | -15.65045657 | 6.32E-43 | 1.18E-41 | 86.6889621 |
| ABCB6 | 1.100867736 | 7.669857391 | 15.6503721 | 6.33E-43 | 1.18E-41 | 86.68815972 |
| SIX4 | 1.615694095 | 7.490280678 | 15.64846351 | 6.44E-43 | 1.20E-41 | 86.67002961 |
| P2RY14 | -0.919553903 | 5.582349029 | -15.64419998 | 6.71E-43 | 1.24E-41 | 86.62953105 |
| GREM1 | 2.318850898 | 6.718741809 | 15.64002281 | 6.98E-43 | 1.29E-41 | 86.58985515 |
| DCN | -1.89900438 | 10.53210251 | -15.63404856 | 7.39E-43 | 1.37E-41 | 86.53311394 |
| DKC1 | 0.849531116 | 9.012911649 | 15.62336787 | 8.19E-43 | 1.51E-41 | 86.43168421 |
| EIF4E3 | -0.960625945 | 7.830633513 | -15.61927588 | 8.51E-43 | 1.57E-41 | 86.39282838 |
| NARF | 0.83618653 | 9.121092649 | 15.61819893 | 8.60E-43 | 1.58E-41 | 86.38260243 |
| PLA2G5 | -1.083038003 | 5.760721638 | -15.60598166 | 9.66E-43 | 1.77E-41 | 86.26660687 |
| HS6ST2 | 1.713845763 | 5.603677465 | 15.60325706 | 9.92E-43 | 1.82E-41 | 86.24074118 |
| PILRA | -1.180190026 | 7.435645838 | -15.60060849 | 1.02E-42 | 1.86E-41 | 86.21559808 |
| H1F0 | 0.976428607 | 10.4421302 | 15.59450418 | 1.08E-42 | 1.97E-41 | 86.15765298 |
| C17orf53 | 1.331079215 | 6.32385266 | 15.59435924 | 1.08E-42 | 1.97E-41 | 86.15627724 |
| TMED9 | 0.590225134 | 10.77058941 | 15.57390206 | 1.31E-42 | 2.39E-41 | 85.96212366 |
| ID3 | -1.408657104 | 9.647258003 | -15.56531828 | 1.42E-42 | 2.59E-41 | 85.88067372 |
| ZFP36 | -1.477826601 | 11.39803747 | -15.55796294 | 1.53E-42 | 2.77E-41 | 85.81088803 |
| CETP | -0.893184621 | 5.748508301 | -15.53260363 | 1.94E-42 | 3.52E-41 | 85.57034013 |
| SDCBP | -1.01017714 | 10.55961194 | -15.5323005 | 1.95E-42 | 3.52E-41 | 85.56746521 |
| ZBTB16 | -2.073186208 | 7.179253508 | -15.51816731 | 2.23E-42 | 4.03E-41 | 85.43344149 |
| DKK2 | -1.45258321 | 5.359072332 | -15.51742091 | 2.25E-42 | 4.05E-41 | 85.42636424 |
| IPO4 | 0.87513816 | 7.610191439 | 15.50261191 | 2.59E-42 | 4.65E-41 | 85.28596219 |
| HELLS | 1.227183641 | 5.234515104 | 15.49497453 | 2.78E-42 | 5.00E-41 | 85.21356478 |
| AK1 | -1.161658544 | 8.534269654 | -15.48690963 | 3.00E-42 | 5.39E-41 | 85.1371234 |
| NIPSNAP1 | 0.813578574 | 8.550672077 | 15.48071731 | 3.19E-42 | 5.70E-41 | 85.07843691 |
| CENPM | 1.128088402 | 6.701985689 | 15.47897288 | 3.24E-42 | 5.79E-41 | 85.06190534 |
| C9orf72 | -0.78155061 | 6.173526734 | -15.4703933 | 3.52E-42 | 6.27E-41 | 84.98060466 |
| PRAM1 | -1.300459589 | 6.655363269 | -15.46133471 | 3.83E-42 | 6.82E-41 | 84.8947757 |
| COL13A1 | -1.113609513 | 6.792331686 | -15.4592944 | 3.91E-42 | 6.95E-41 | 84.87544563 |
| PREX1 | -1.058207457 | 7.442486497 | -15.45042279 | 4.25E-42 | 7.55E-41 | 84.79140162 |
| GIMAP7 | -1.181221887 | 7.295303136 | -15.4480592 | 4.35E-42 | 7.70E-41 | 84.76901238 |
| HPCAL1 | -0.905329103 | 7.824878162 | -15.44782858 | 4.36E-42 | 7.71E-41 | 84.76682782 |
| XDH | 1.699235292 | 5.65873721 | 15.44002146 | 4.69E-42 | 8.29E-41 | 84.6928799 |
| CAB39L | -0.983376406 | 6.160992168 | -15.43947342 | 4.72E-42 | 8.32E-41 | 84.68768929 |
| PDE8B | -1.299938273 | 5.955102777 | -15.43687939 | 4.84E-42 | 8.51E-41 | 84.66312111 |
| SDF2L1 | 0.70895841 | 9.600386822 | 15.43134813 | 5.10E-42 | 8.96E-41 | 84.6107373 |
| SMPDL3B | 1.40342096 | 7.342059572 | 15.42882203 | 5.22E-42 | 9.16E-41 | 84.58681528 |
| XPO5 | 0.842186347 | 7.686421686 | 15.41738341 | 5.82E-42 | 1.02E-40 | 84.47850324 |
| APOL3 | -0.795233999 | 6.660409149 | -15.41254073 | 6.09E-42 | 1.07E-40 | 84.43265352 |
| SSH2 | -0.753907372 | 8.490701723 | -15.41117803 | 6.17E-42 | 1.08E-40 | 84.41975229 |
| PIP5K1B | -1.31289392 | 5.911695755 | -15.40167947 | 6.76E-42 | 1.18E-40 | 84.3298327 |
| REM1 | -1.14627353 | 6.483728734 | -15.39538876 | 7.17E-42 | 1.25E-40 | 84.27028762 |
| RBP4 | -1.785850076 | 6.199833215 | -15.39009158 | 7.54E-42 | 1.31E-40 | 84.22015121 |
| PARP1 | 0.752739907 | 9.939472899 | 15.37731658 | 8.52E-42 | 1.48E-40 | 84.09925527 |
| SLCO2A1 | -1.581129307 | 9.668369886 | -15.37434675 | 8.76E-42 | 1.52E-40 | 84.0711537 |
| ADRB1 | -1.14794078 | 5.422836673 | -15.37175852 | 8.98E-42 | 1.55E-40 | 84.04666389 |
| ROR1 | -1.223127158 | 6.899131484 | -15.36618263 | 9.47E-42 | 1.63E-40 | 83.99390816 |
| CANT1 | 0.792234585 | 8.959522596 | 15.35714812 | 1.03E-41 | 1.78E-40 | 83.90843834 |
| DBNDD1 | 1.003075934 | 7.769111019 | 15.35272346 | 1.08E-41 | 1.85E-40 | 83.86658367 |
| CES1 | -1.981067921 | 8.517717747 | -15.34457297 | 1.16E-41 | 2.00E-40 | 83.78949205 |
| OSCAR | -1.286730457 | 7.376152085 | -15.32407519 | 1.41E-41 | 2.42E-40 | 83.5956551 |
| FAM64A | 1.396652206 | 5.803877287 | 15.32069916 | 1.46E-41 | 2.50E-40 | 83.56373548 |
| TIMELESS | 1.029431868 | 7.67321246 | 15.31634968 | 1.52E-41 | 2.60E-40 | 83.52261452 |
| NRN1 | -1.455782794 | 6.15634187 | -15.31361452 | 1.56E-41 | 2.66E-40 | 83.4967571 |
| PCDH12 | -1.034956637 | 6.712625191 | -15.31051118 | 1.61E-41 | 2.74E-40 | 83.46742037 |
| PFKP | 1.309214003 | 9.539352354 | 15.29567553 | 1.85E-41 | 3.14E-40 | 83.32719369 |
| MND1 | 1.376477745 | 4.931930269 | 15.29167771 | 1.92E-41 | 3.26E-40 | 83.28941165 |
| GYLTL1B | 1.166010817 | 6.285043306 | 15.2908139 | 1.94E-41 | 3.28E-40 | 83.28124844 |
| F12 | 1.410917866 | 6.806470128 | 15.28759838 | 2.00E-41 | 3.38E-40 | 83.25086179 |
| KIAA0355 | -0.68875415 | 8.931578516 | -15.28100459 | 2.13E-41 | 3.59E-40 | 83.18855515 |
| MCM10 | 1.289811425 | 5.26721117 | 15.26861428 | 2.39E-41 | 4.03E-40 | 83.07149262 |
| SRGN | -1.405143437 | 10.43796824 | -15.25764692 | 2.65E-41 | 4.47E-40 | 82.96789257 |
| TSC22D3 | -1.013964472 | 8.449923553 | -15.25522651 | 2.71E-41 | 4.56E-40 | 82.94503123 |
| CALCOCO1 | -0.789525954 | 8.239739388 | -15.23105677 | 3.41E-41 | 5.73E-40 | 82.71678955 |
| TLCD1 | 1.272726402 | 7.505068662 | 15.22726723 | 3.54E-41 | 5.93E-40 | 82.68101171 |
| GABARAPL1 | -0.946366097 | 9.429606237 | -15.21151469 | 4.11E-41 | 6.88E-40 | 82.53231108 |
| B3GALNT1 | -1.357827088 | 7.472033763 | -15.20703452 | 4.29E-41 | 7.16E-40 | 82.49002595 |
| PYGO2 | 1.109353773 | 7.313070846 | 15.185563 | 5.26E-41 | 8.76E-40 | 82.2874131 |
| ALDH2 | -1.083970886 | 11.04519884 | -15.17646365 | 5.73E-41 | 9.54E-40 | 82.20156923 |
| REV3L | -1.051972709 | 6.580732434 | -15.1747992 | 5.82E-41 | 9.68E-40 | 82.18586804 |
| DUS1L | 0.873850511 | 8.237305614 | 15.16878921 | 6.16E-41 | 1.02E-39 | 82.12917767 |
| CA2 | -1.846771561 | 8.510098356 | -15.16474314 | 6.40E-41 | 1.06E-39 | 82.09101538 |
| LMO7 | -1.380525286 | 8.090848059 | -15.16392783 | 6.45E-41 | 1.07E-39 | 82.08332572 |
| TGM1 | -1.05447732 | 5.835549612 | -15.16072629 | 6.65E-41 | 1.10E-39 | 82.05313115 |
| SRM | 0.736781815 | 9.238624934 | 15.15015024 | 7.35E-41 | 1.21E-39 | 81.95339657 |
| SLC11A1 | -1.055363539 | 6.867572207 | -15.14668276 | 7.60E-41 | 1.25E-39 | 81.92070117 |
| HM13 | 0.70214226 | 7.573005027 | 15.13971575 | 8.11E-41 | 1.33E-39 | 81.8550136 |
| LMCD1 | -1.09132834 | 9.052098441 | -15.11879724 | 9.89E-41 | 1.62E-39 | 81.65783026 |
| MMP12 | 2.775389029 | 6.450950282 | 15.11781144 | 9.98E-41 | 1.64E-39 | 81.64853947 |
| SLC1A4 | 1.047852035 | 7.20412975 | 15.11664214 | 1.01E-40 | 1.65E-39 | 81.63751946 |
| ARHGEF19 | 0.978506747 | 7.876472423 | 15.0997595 | 1.18E-40 | 1.94E-39 | 81.47843356 |
| CRTAP | -0.75656905 | 7.898745077 | -15.09640126 | 1.22E-40 | 2.00E-39 | 81.44679389 |
| METTL7B | 2.007744341 | 6.645608561 | 15.08570934 | 1.35E-40 | 2.20E-39 | 81.34607161 |
| SGPP2 | 1.375808752 | 7.319671771 | 15.08315713 | 1.39E-40 | 2.26E-39 | 81.32203131 |
| LSAMP | -0.996095274 | 6.718147125 | -15.04465491 | 1.99E-40 | 3.24E-39 | 80.959486 |
| MYLK | -0.941828254 | 8.152902676 | -15.04019013 | 2.08E-40 | 3.38E-39 | 80.91745959 |
| GCAT | 1.01551312 | 7.468150625 | 15.03946051 | 2.09E-40 | 3.39E-39 | 80.91059207 |
| CDCA7 | 1.503060512 | 6.354283545 | 15.03769906 | 2.13E-40 | 3.45E-39 | 80.89401285 |
| PLCL1 | -0.863040521 | 5.234846915 | -15.03017395 | 2.29E-40 | 3.69E-39 | 80.8231899 |
| IDH2 | 0.903693913 | 9.848106399 | 15.02515125 | 2.40E-40 | 3.87E-39 | 80.77592337 |
| BCL6B | -0.993932222 | 6.778628694 | -15.01293113 | 2.69E-40 | 4.34E-39 | 80.66094166 |
| ASF1B | 1.452201084 | 6.749106205 | 15.00643223 | 2.86E-40 | 4.60E-39 | 80.59980173 |
| CPA3 | -2.247081389 | 8.831046431 | -14.99855431 | 3.08E-40 | 4.94E-39 | 80.52569728 |
| ALDH1A2 | -1.665693341 | 6.565209386 | -14.9959211 | 3.16E-40 | 5.06E-39 | 80.50092988 |
| KDELR3 | 1.288515722 | 8.028575178 | 14.99317963 | 3.24E-40 | 5.19E-39 | 80.47514546 |
| BMPR2 | -0.9650942 | 8.839535566 | -14.99157416 | 3.29E-40 | 5.26E-39 | 80.46004601 |
| RAB8B | -0.913229769 | 8.845737019 | -14.98546446 | 3.49E-40 | 5.56E-39 | 80.40258798 |
| RASSF2 | -0.842108874 | 7.546401729 | -14.98445362 | 3.52E-40 | 5.61E-39 | 80.39308224 |
| SFTPC | -3.677258906 | 11.27885489 | -14.97515039 | 3.85E-40 | 6.11E-39 | 80.30560389 |
| RGS18 | -1.226515113 | 5.77610692 | -14.97462403 | 3.87E-40 | 6.13E-39 | 80.30065493 |
| WDR62 | 0.985021898 | 5.680423215 | 14.96636513 | 4.18E-40 | 6.62E-39 | 80.22300872 |
| CST1 | 3.174605037 | 6.597700154 | 14.96353717 | 4.29E-40 | 6.79E-39 | 80.19642403 |
| MKI67 | 1.312257308 | 5.829370223 | 14.96071263 | 4.41E-40 | 6.96E-39 | 80.16987285 |
| PBK | 1.937635305 | 5.455140402 | 14.95998946 | 4.44E-40 | 7.00E-39 | 80.16307517 |
| EGR2 | -1.425092145 | 8.330127202 | -14.94647905 | 5.04E-40 | 7.94E-39 | 80.03609405 |
| CD300LF | -1.51477272 | 7.87101037 | -14.94310106 | 5.21E-40 | 8.19E-39 | 80.00434971 |
| DKK3 | -0.773471035 | 7.328172878 | -14.94287102 | 5.22E-40 | 8.19E-39 | 80.00218792 |
| TSPAN4 | -0.755876171 | 8.211616245 | -14.93700879 | 5.51E-40 | 8.65E-39 | 79.94710275 |
| SEMA4B | 0.956722429 | 7.843133503 | 14.93402794 | 5.67E-40 | 8.88E-39 | 79.919095 |
| NTN4 | -1.399389073 | 7.798581609 | -14.92707384 | 6.06E-40 | 9.47E-39 | 79.85376046 |
| ALOX5AP | -1.541490015 | 10.73336784 | -14.92486036 | 6.18E-40 | 9.66E-39 | 79.83296616 |
| TUBB3 | 1.722376491 | 8.711548059 | 14.92219229 | 6.34E-40 | 9.89E-39 | 79.80790236 |
| CIRBP | -0.763988913 | 10.65283272 | -14.91682471 | 6.67E-40 | 1.04E-38 | 79.75748298 |
| KIF15 | 1.524161181 | 5.284894436 | 14.9163959 | 6.70E-40 | 1.04E-38 | 79.75345518 |
| FBXO7 | -0.80413027 | 8.50967566 | -14.9029453 | 7.60E-40 | 1.18E-38 | 79.62713076 |
| RRM2 | 1.581580229 | 6.058971226 | 14.8991597 | 7.88E-40 | 1.22E-38 | 79.59158274 |
| PROS1 | -1.320868716 | 9.389170899 | -14.89652067 | 8.08E-40 | 1.25E-38 | 79.56680277 |
| FBLN1 | -1.058345538 | 8.240081455 | -14.89351216 | 8.31E-40 | 1.28E-38 | 79.53855488 |
| COL11A1 | 1.900766982 | 5.602530545 | 14.89284315 | 8.36E-40 | 1.29E-38 | 79.53227352 |
| C5AR1 | -1.52427575 | 7.917050114 | -14.88414093 | 9.08E-40 | 1.40E-38 | 79.45057465 |
| SGCE | -1.346310691 | 9.211700699 | -14.87903381 | 9.53E-40 | 1.46E-38 | 79.40263332 |
| NEXN | -1.240689851 | 7.237498157 | -14.87380462 | 1.00E-39 | 1.54E-38 | 79.35355055 |
| SEC22C | -0.823997203 | 8.090963923 | -14.87096857 | 1.03E-39 | 1.58E-38 | 79.32693242 |
| HIST1H2BD | 1.187458167 | 7.449814061 | 14.86538579 | 1.08E-39 | 1.66E-38 | 79.27453834 |
| NOTCH1 | -0.824279032 | 9.065590896 | -14.85783528 | 1.16E-39 | 1.78E-38 | 79.20368529 |
| C7 | -2.367302437 | 8.545152096 | -14.85308364 | 1.22E-39 | 1.86E-38 | 79.15910133 |
| SUSD2 | -1.913581539 | 10.28162608 | -14.85284062 | 1.22E-39 | 1.86E-38 | 79.15682122 |
| PAK6 | 1.110836473 | 6.677203133 | 14.84543452 | 1.31E-39 | 1.99E-38 | 79.08733861 |
| WFDC3 | 1.420544902 | 5.964391886 | 14.83420506 | 1.45E-39 | 2.21E-38 | 78.98200328 |
| ABCC3 | 1.236326144 | 7.831143779 | 14.81994908 | 1.66E-39 | 2.52E-38 | 78.84830858 |
| FOSB | -2.725694937 | 9.871000261 | -14.81661422 | 1.72E-39 | 2.60E-38 | 78.81703865 |
| RUSC1 | 0.697592277 | 9.446748795 | 14.80901239 | 1.84E-39 | 2.79E-38 | 78.74576557 |
| ZNF25 | -0.787662569 | 7.773180319 | -14.80797042 | 1.86E-39 | 2.81E-38 | 78.73599708 |
| BTG2 | -1.363438651 | 9.572625412 | -14.80789694 | 1.86E-39 | 2.81E-38 | 78.73530825 |
| CHTF18 | 1.22000576 | 6.948219545 | 14.79926831 | 2.02E-39 | 3.04E-38 | 78.65442143 |
| IER5L | 1.067065834 | 7.588091005 | 14.79333182 | 2.14E-39 | 3.21E-38 | 78.59877867 |
| PZP | -1.334348394 | 5.829428314 | -14.7889161 | 2.23E-39 | 3.35E-38 | 78.55739401 |
| ARHGAP24 | -0.871424496 | 6.685664721 | -14.77940499 | 2.44E-39 | 3.65E-38 | 78.46826569 |
| STK39 | 1.126192592 | 9.124865253 | 14.77690758 | 2.49E-39 | 3.74E-38 | 78.44486509 |
| ABLIM3 | -1.305437014 | 6.869540896 | -14.77192681 | 2.61E-39 | 3.91E-38 | 78.39819865 |
| FANCD2 | 0.78022515 | 5.89045366 | 14.76391021 | 2.82E-39 | 4.21E-38 | 78.32309732 |
| ABCC10 | 0.632926628 | 7.343148734 | 14.75263223 | 3.13E-39 | 4.67E-38 | 78.21746119 |
| PDLIM2 | -0.590950178 | 6.247249761 | -14.73612164 | 3.66E-39 | 5.45E-38 | 78.06285233 |
| SULT1A2 | -0.828774117 | 7.126230053 | -14.72746232 | 3.97E-39 | 5.90E-38 | 77.98178306 |
| IL18RAP | -1.332000378 | 6.38226421 | -14.72237583 | 4.16E-39 | 6.18E-38 | 77.93416891 |
| TMEM54 | 0.930026709 | 9.119555638 | 14.70004981 | 5.14E-39 | 7.61E-38 | 77.72522981 |
| PPBP | -1.951062661 | 5.243893625 | -14.68844273 | 5.73E-39 | 8.48E-38 | 77.61663838 |
| CDKN3 | 1.826366666 | 5.984532505 | 14.68327763 | 6.01E-39 | 8.89E-38 | 77.5683232 |
| BST1 | -1.029254438 | 6.061102758 | -14.68175455 | 6.10E-39 | 9.00E-38 | 77.55407691 |
| MMP9 | 2.052307854 | 8.781254082 | 14.67630496 | 6.42E-39 | 9.46E-38 | 77.50310693 |
| NXF3 | -1.274959723 | 5.132359649 | -14.67457573 | 6.53E-39 | 9.61E-38 | 77.48693457 |
| C1orf186 | -1.532234843 | 6.60283058 | -14.6722447 | 6.67E-39 | 9.81E-38 | 77.46513475 |
| KRT8 | 1.252531924 | 10.83146909 | 14.66230404 | 7.32E-39 | 1.08E-37 | 77.37218025 |
| GAB1 | -0.598539073 | 5.976223566 | -14.6373725 | 9.26E-39 | 1.36E-37 | 77.13912276 |
| ABCA3 | -1.890918105 | 9.739026516 | -14.63095533 | 9.83E-39 | 1.44E-37 | 77.07915334 |
| ABHD5 | -0.857658946 | 7.624643968 | -14.63043889 | 9.88E-39 | 1.44E-37 | 77.07432746 |
| SMUG1 | 0.608547816 | 8.462933918 | 14.62772292 | 1.01E-38 | 1.48E-37 | 77.04894872 |
| RGS17 | 2.121983784 | 6.202659088 | 14.61150284 | 1.18E-38 | 1.72E-37 | 76.89741125 |
| A2M | -1.212941996 | 11.96128624 | -14.60470499 | 1.26E-38 | 1.83E-37 | 76.83391558 |
| SYNE1 | -0.69136601 | 5.63376596 | -14.59555217 | 1.37E-38 | 1.99E-37 | 76.74843608 |
| GNG2 | -1.153946038 | 5.948159194 | -14.59272451 | 1.41E-38 | 2.04E-37 | 76.72203122 |
| MOCS1 | -0.701920857 | 6.918047181 | -14.57635081 | 1.64E-38 | 2.38E-37 | 76.56916034 |
| MSRA | -0.913365566 | 8.188390798 | -14.5747404 | 1.67E-38 | 2.41E-37 | 76.55412759 |
| NCF2 | -1.469755304 | 7.880204027 | -14.57263301 | 1.70E-38 | 2.46E-37 | 76.53445624 |
| CLIC3 | -1.536611848 | 8.861170372 | -14.56179459 | 1.88E-38 | 2.72E-37 | 76.43329834 |
| METTL7A | -1.424066515 | 8.550457787 | -14.56078672 | 1.90E-38 | 2.74E-37 | 76.42389271 |
| TLR8 | -1.337016313 | 6.31361125 | -14.53761813 | 2.36E-38 | 3.40E-37 | 76.20772894 |
| C1QTNF6 | 0.857038141 | 6.000141098 | 14.53699134 | 2.37E-38 | 3.41E-37 | 76.20188226 |
| IFT57 | -1.316083119 | 8.546665064 | -14.53027321 | 2.53E-38 | 3.62E-37 | 76.13922072 |
| RHOD | 1.205174487 | 8.148419779 | 14.49852506 | 3.40E-38 | 4.87E-37 | 75.84320915 |
| TTLL7 | -1.212733773 | 5.505016899 | -14.49172666 | 3.63E-38 | 5.18E-37 | 75.77984653 |
| SLC39A7 | 0.724775544 | 7.609688396 | 14.47877073 | 4.09E-38 | 5.84E-37 | 75.65911767 |
| NR2F6 | 0.692700049 | 8.901562348 | 14.4766504 | 4.18E-38 | 5.95E-37 | 75.6393624 |
| KCNN4 | 1.533362399 | 6.930628577 | 14.47429802 | 4.27E-38 | 6.08E-37 | 75.61744618 |
| ZMYND19 | 0.710762594 | 8.021811117 | 14.46240673 | 4.77E-38 | 6.78E-37 | 75.50667496 |
| ITIH5 | -0.882088167 | 6.464318777 | -14.46027868 | 4.87E-38 | 6.91E-37 | 75.48685424 |
| CDC25C | 1.025584949 | 4.920685617 | 14.45353798 | 5.18E-38 | 7.35E-37 | 75.42407667 |
| HDGF | 0.653198341 | 10.34687175 | 14.44286411 | 5.73E-38 | 8.11E-37 | 75.32468578 |
| SCAMP5 | 0.857759941 | 6.844199596 | 14.43581353 | 6.12E-38 | 8.65E-37 | 75.25904516 |
| SLC44A2 | -0.703363648 | 10.40671934 | -14.4348363 | 6.18E-38 | 8.70E-37 | 75.24994786 |
| FLJ22184 | 1.329190127 | 7.097734899 | 14.43480149 | 6.18E-38 | 8.70E-37 | 75.24962379 |
| RGS22 | -1.33609008 | 5.197323883 | -14.43476759 | 6.18E-38 | 8.70E-37 | 75.24930821 |
| UBE2O | 0.804826679 | 7.359570082 | 14.42762366 | 6.61E-38 | 9.29E-37 | 75.1828093 |
| FPR1 | -1.848628684 | 7.937369021 | -14.42441658 | 6.81E-38 | 9.56E-37 | 75.15295948 |
| BEX1 | -1.24374787 | 5.905631492 | -14.42050405 | 7.06E-38 | 9.91E-37 | 75.11654617 |
| AP1S2 | -0.985202716 | 8.418391588 | -14.39243145 | 9.18E-38 | 1.28E-36 | 74.85536326 |
| MAP6 | -0.813080178 | 5.63544934 | -14.38394296 | 9.94E-38 | 1.39E-36 | 74.77641679 |
| SYNJ2 | 0.906913295 | 6.328284731 | 14.3805891 | 1.03E-37 | 1.43E-36 | 74.74522827 |
| AQP1 | -2.000810621 | 8.783697186 | -14.37732954 | 1.06E-37 | 1.47E-36 | 74.7149186 |
| PYCRL | 0.906789353 | 5.943443984 | 14.3749492 | 1.08E-37 | 1.50E-36 | 74.69278591 |
| KRTCAP2 | 0.592344055 | 11.12208908 | 14.37378025 | 1.09E-37 | 1.52E-36 | 74.68191725 |
| TJP2 | -0.909113248 | 7.87773837 | -14.36651486 | 1.17E-37 | 1.62E-36 | 74.61437081 |
| TSPAN18 | -0.821876244 | 6.450609777 | -14.35987103 | 1.24E-37 | 1.73E-36 | 74.55261174 |
| NDN | -1.104754116 | 8.862501737 | -14.35502784 | 1.30E-37 | 1.80E-36 | 74.50759622 |
| EPRS | 0.691010897 | 9.842992035 | 14.35403041 | 1.31E-37 | 1.82E-36 | 74.49832604 |
| LDLR | -1.129029739 | 10.2616843 | -14.33676792 | 1.54E-37 | 2.13E-36 | 74.33791749 |
| ETS1 | -0.885015025 | 9.269379021 | -14.33054117 | 1.64E-37 | 2.26E-36 | 74.28007045 |
| RGN | -1.042763979 | 6.155038271 | -14.32672393 | 1.70E-37 | 2.34E-36 | 74.24461167 |
| CX3CR1 | -1.549695137 | 6.41367816 | -14.32363864 | 1.75E-37 | 2.40E-36 | 74.21595404 |
| ADHFE1 | -1.066461523 | 6.883649051 | -14.31982626 | 1.81E-37 | 2.49E-36 | 74.18054543 |
| SLC5A6 | 0.753271811 | 7.955184987 | 14.31429997 | 1.90E-37 | 2.61E-36 | 74.12922323 |
| RAD54L | 1.033104976 | 5.94480392 | 14.31137478 | 1.96E-37 | 2.68E-36 | 74.1020596 |
| PTGFRN | 0.95562127 | 8.029399037 | 14.3108866 | 1.97E-37 | 2.69E-36 | 74.09752645 |
| PDZRN3 | -0.843440822 | 5.819626689 | -14.31084803 | 1.97E-37 | 2.69E-36 | 74.09716833 |
| NEDD9 | -1.098117979 | 8.025471441 | -14.30836633 | 2.01E-37 | 2.75E-36 | 74.07412454 |
| DNAJC18 | -0.696723723 | 6.326451644 | -14.29632728 | 2.25E-37 | 3.07E-36 | 73.962353 |
| CHAF1B | 1.180889189 | 6.241928428 | 14.29447296 | 2.29E-37 | 3.12E-36 | 73.94513979 |
| CYP2U1 | -0.864665485 | 6.195762205 | -14.29312538 | 2.32E-37 | 3.16E-36 | 73.93263098 |
| ADAM28 | 1.247044808 | 6.38733937 | 14.28658842 | 2.47E-37 | 3.35E-36 | 73.87195701 |
| TJP1 | -0.829455529 | 9.218580109 | -14.27940462 | 2.64E-37 | 3.57E-36 | 73.80528883 |
| PUSL1 | 0.779109447 | 7.392128441 | 14.27856657 | 2.66E-37 | 3.60E-36 | 73.7975121 |
| HDC | -1.375797897 | 6.837987755 | -14.27180906 | 2.83E-37 | 3.82E-36 | 73.73481024 |
| RGS13 | -0.838269484 | 4.612534202 | -14.27014477 | 2.87E-37 | 3.88E-36 | 73.71936892 |
| FER1L4 | 1.195794954 | 6.02860633 | 14.26356086 | 3.06E-37 | 4.12E-36 | 73.65828853 |
| TPK1 | -0.877376124 | 5.956317729 | -14.25530268 | 3.30E-37 | 4.44E-36 | 73.58168743 |
| LRRC18 | -0.933964152 | 5.033599835 | -14.24870197 | 3.51E-37 | 4.72E-36 | 73.5204702 |
| BLM | 1.105535166 | 5.98274421 | 14.24431833 | 3.66E-37 | 4.91E-36 | 73.47981955 |
| SLC22A18AS | 1.427410704 | 6.279689303 | 14.23314524 | 4.06E-37 | 5.44E-36 | 73.37622556 |
| QDPR | -0.815138538 | 9.079028011 | -14.22545243 | 4.36E-37 | 5.83E-36 | 73.30491401 |
| IL11RA | -0.595457476 | 6.663340912 | -14.2180942 | 4.67E-37 | 6.24E-36 | 73.23671491 |
| DPP3 | 0.665895218 | 8.040452803 | 14.21215817 | 4.93E-37 | 6.59E-36 | 73.18170499 |
| MAPRE2 | -0.714058165 | 8.843795521 | -14.20308705 | 5.37E-37 | 7.16E-36 | 73.09765537 |
| GSN | -1.044356594 | 8.766290415 | -14.19849083 | 5.60E-37 | 7.46E-36 | 73.05507471 |
| PRDX4 | 0.733705602 | 10.41144021 | 14.19250753 | 5.92E-37 | 7.88E-36 | 72.99964999 |
| MARCKSL1 | 1.031740026 | 10.56206088 | 14.19144984 | 5.98E-37 | 7.95E-36 | 72.98985305 |
| NEBL | -0.965654522 | 6.697619399 | -14.19014956 | 6.06E-37 | 8.04E-36 | 72.97780945 |
| RTN1 | -0.900080489 | 5.948582136 | -14.19001992 | 6.06E-37 | 8.04E-36 | 72.9766087 |
| PLEKHG6 | 1.219143501 | 5.927167423 | 14.17964066 | 6.68E-37 | 8.84E-36 | 72.88048517 |
| DENR | 0.903258796 | 9.01226908 | 14.17535213 | 6.95E-37 | 9.19E-36 | 72.84077483 |
| GRIA1 | -0.990234464 | 5.041096476 | -14.17173053 | 7.19E-37 | 9.49E-36 | 72.80724281 |
| RAB26 | 1.25408999 | 6.126392814 | 14.16176614 | 7.89E-37 | 1.04E-35 | 72.71499726 |
| COL3A1 | 1.49599229 | 10.94035222 | 14.15898361 | 8.09E-37 | 1.07E-35 | 72.68924146 |
| LST1 | -1.013572472 | 7.889626457 | -14.14226373 | 9.45E-37 | 1.24E-35 | 72.53451058 |
| DSP | 1.506483454 | 7.89974775 | 14.14151728 | 9.52E-37 | 1.25E-35 | 72.52760396 |
| SEC14L1 | -0.813861715 | 8.148287883 | -14.14118304 | 9.55E-37 | 1.25E-35 | 72.52451144 |
| NAP1L5 | -1.054964594 | 6.57408154 | -14.13853729 | 9.79E-37 | 1.28E-35 | 72.50003259 |
| UACA | -0.689676731 | 6.332459867 | -14.13518514 | 1.01E-36 | 1.32E-35 | 72.46902003 |
| HLF | -1.633606689 | 6.856173261 | -14.1270176 | 1.09E-36 | 1.42E-35 | 72.39346712 |
| SGCA | -1.176904558 | 6.629523753 | -14.12623109 | 1.10E-36 | 1.43E-35 | 72.38619226 |
| EPB41L3 | -1.325621237 | 7.932332606 | -14.11671936 | 1.20E-36 | 1.56E-35 | 72.29822355 |
| C10orf32 | -0.901322008 | 8.181580117 | -14.11610719 | 1.21E-36 | 1.57E-35 | 72.29256257 |
| PSMD11 | 0.747216279 | 7.989323423 | 14.10969027 | 1.28E-36 | 1.66E-35 | 72.23322712 |
| ADPRH | -0.853163666 | 5.47185408 | -14.10769627 | 1.30E-36 | 1.69E-35 | 72.2147909 |
| SLC25A25 | -0.694942886 | 6.661545971 | -14.10744058 | 1.31E-36 | 1.69E-35 | 72.21242686 |
| E2F3 | 0.88315178 | 7.842844266 | 14.09723794 | 1.44E-36 | 1.86E-35 | 72.11810773 |
| PTTG1 | 1.255877211 | 8.220493801 | 14.09499126 | 1.47E-36 | 1.90E-35 | 72.09734094 |
| SKIV2L | 0.639435906 | 8.861681596 | 14.08805433 | 1.56E-36 | 2.02E-35 | 72.03322718 |
| LRRC49 | -1.104018416 | 6.503349691 | -14.08068552 | 1.68E-36 | 2.16E-35 | 71.96513254 |
| DOK6 | -1.061116086 | 5.270478207 | -14.07141905 | 1.83E-36 | 2.35E-35 | 71.87951744 |
| ZNF331 | -1.034262591 | 6.986433745 | -14.06734713 | 1.90E-36 | 2.44E-35 | 71.8419016 |
| RPGR | -0.759567828 | 5.890665473 | -14.06348099 | 1.97E-36 | 2.53E-35 | 71.80618988 |
| B3GNTL1 | 0.770100478 | 6.091647532 | 14.05070416 | 2.21E-36 | 2.84E-35 | 71.68819127 |
| SSR4 | 0.61374428 | 11.54942439 | 14.04909734 | 2.25E-36 | 2.88E-35 | 71.67335414 |
| PIK3R1 | -0.653164937 | 7.276551367 | -14.04343758 | 2.37E-36 | 3.03E-35 | 71.62109685 |
| L2HGDH | 0.980679167 | 6.78922137 | 14.0283786 | 2.72E-36 | 3.48E-35 | 71.4820874 |
| CKS1B | 1.05435321 | 9.581716548 | 14.01387093 | 3.11E-36 | 3.98E-35 | 71.34821143 |
| S100A4 | -1.388939147 | 11.13679241 | -14.01360495 | 3.12E-36 | 3.99E-35 | 71.34575738 |
| KIAA0907 | 0.805720804 | 8.588110085 | 14.00857988 | 3.27E-36 | 4.17E-35 | 71.2993968 |
| CCBE1 | -1.133166123 | 7.104050346 | -14.00744286 | 3.31E-36 | 4.21E-35 | 71.28890754 |
| CD83 | -1.189801397 | 8.798166527 | -13.99650863 | 3.66E-36 | 4.65E-35 | 71.18805044 |
| FLAD1 | 0.63650234 | 7.352655415 | 13.99639538 | 3.66E-36 | 4.65E-35 | 71.18700596 |
| MORC2 | 0.678914343 | 8.015942684 | 13.98968038 | 3.90E-36 | 4.95E-35 | 71.12507949 |
| KIAA0040 | -0.85757983 | 7.034348106 | -13.9755086 | 4.44E-36 | 5.63E-35 | 70.99441664 |
| AKAP12 | -1.14971143 | 6.454753779 | -13.97311502 | 4.54E-36 | 5.75E-35 | 70.97235215 |
| CBX8 | 0.768251525 | 6.238818957 | 13.96169286 | 5.05E-36 | 6.38E-35 | 70.86707698 |
| ABCB9 | 0.734723945 | 6.023192657 | 13.95724784 | 5.26E-36 | 6.64E-35 | 70.82611572 |
| RHPN1 | 0.798448214 | 6.914041657 | 13.95366107 | 5.44E-36 | 6.85E-35 | 70.79306633 |
| CKAP4 | 0.671725996 | 10.05282592 | 13.95357305 | 5.45E-36 | 6.85E-35 | 70.79225536 |
| PPP1R16B | -1.133166966 | 7.421354545 | -13.93727334 | 6.33E-36 | 7.96E-35 | 70.64210037 |
| FRY | -1.126319069 | 6.515698918 | -13.92600485 | 7.03E-36 | 8.81E-35 | 70.5383264 |
| H2AFX | 0.81025 | 7.94031083 | 13.92307416 | 7.22E-36 | 9.05E-35 | 70.5113414 |
| PTPRH | 1.430395786 | 5.652300184 | 13.9179012 | 7.58E-36 | 9.47E-35 | 70.46371478 |
| BTK | -1.196782067 | 7.71626309 | -13.91783247 | 7.58E-36 | 9.47E-35 | 70.46308204 |
| HLA-E | -0.819279261 | 11.57242493 | -13.90975826 | 8.17E-36 | 1.02E-34 | 70.38875542 |
| UNC5CL | 1.38966506 | 5.650101553 | 13.90115975 | 8.84E-36 | 1.10E-34 | 70.30961771 |
| CNFN | 1.398802437 | 6.754421803 | 13.90108739 | 8.85E-36 | 1.10E-34 | 70.3089518 |
| CENPA | 1.122042834 | 6.291639489 | 13.8992301 | 9.00E-36 | 1.12E-34 | 70.29186009 |
| BCL2L2 | -0.742665942 | 8.435720564 | -13.89874459 | 9.04E-36 | 1.12E-34 | 70.28739224 |
| MTMR9 | -0.677833592 | 7.593922066 | -13.89845808 | 9.07E-36 | 1.13E-34 | 70.28475577 |
| RPP40 | 0.928577519 | 7.695140753 | 13.89622936 | 9.26E-36 | 1.15E-34 | 70.26424724 |
| DEPDC1B | 1.217651647 | 4.905537468 | 13.88816907 | 9.97E-36 | 1.23E-34 | 70.19008588 |
| ECM2 | -1.185508671 | 6.818976011 | -13.88228292 | 1.05E-35 | 1.30E-34 | 70.13593718 |
| TTLL12 | 0.863563461 | 8.567901418 | 13.87210545 | 1.16E-35 | 1.43E-34 | 70.04232876 |
| CCDC69 | -1.014942024 | 6.626716258 | -13.86991328 | 1.18E-35 | 1.46E-34 | 70.02216898 |
| KCNJ8 | -1.070890231 | 7.890344662 | -13.86361992 | 1.25E-35 | 1.54E-34 | 69.9642992 |
| CDC25A | 0.816321428 | 5.183981261 | 13.85580884 | 1.35E-35 | 1.65E-34 | 69.89248529 |
| PTGDS | -1.543610819 | 9.910470673 | -13.85412717 | 1.37E-35 | 1.68E-34 | 69.87702594 |
| CHI3L2 | -1.255786135 | 6.354067173 | -13.85279131 | 1.38E-35 | 1.70E-34 | 69.86474609 |
| BCL9 | 0.73215987 | 7.860171202 | 13.84866046 | 1.44E-35 | 1.76E-34 | 69.82677567 |
| NES | -0.997544113 | 7.967389481 | -13.8379069 | 1.59E-35 | 1.94E-34 | 69.72794713 |
| C10orf35 | 0.708262995 | 8.08836409 | 13.8365316 | 1.61E-35 | 1.96E-34 | 69.71530947 |
| E2F2 | 1.12649813 | 6.654927144 | 13.8349445 | 1.63E-35 | 1.99E-34 | 69.70072618 |
| SLC5A9 | -0.913358462 | 5.047147051 | -13.82183915 | 1.84E-35 | 2.24E-34 | 69.58032632 |
| NGEF | 1.299985127 | 5.621050301 | 13.81557454 | 1.95E-35 | 2.37E-34 | 69.52278604 |
| GRTP1 | 1.19766254 | 6.665974367 | 13.80344349 | 2.18E-35 | 2.65E-34 | 69.41138702 |
| TYMS | 1.426507674 | 7.899077125 | 13.79881866 | 2.28E-35 | 2.76E-34 | 69.36892579 |
| BYSL | 0.858161997 | 7.715602285 | 13.78232687 | 2.65E-35 | 3.21E-34 | 69.2175505 |
| ZNF687 | 0.783978995 | 6.048866878 | 13.77729985 | 2.78E-35 | 3.36E-34 | 69.17142021 |
| COLEC12 | -1.161978251 | 7.726586178 | -13.76609359 | 3.08E-35 | 3.72E-34 | 69.06860643 |
| EPB41L5 | -1.073428355 | 7.450952715 | -13.76596392 | 3.08E-35 | 3.72E-34 | 69.06741691 |
| PDLIM3 | -1.340103358 | 8.422157436 | -13.75732279 | 3.34E-35 | 4.03E-34 | 68.98815641 |
| FEN1 | 0.948750064 | 7.634081947 | 13.75390572 | 3.44E-35 | 4.15E-34 | 68.95681802 |
| PROK2 | -1.523859986 | 5.621275625 | -13.75281167 | 3.48E-35 | 4.19E-34 | 68.94678491 |
| PRSS22 | 1.073836652 | 6.344028564 | 13.7476123 | 3.65E-35 | 4.38E-34 | 68.89910705 |
| TCEAL7 | -0.768445228 | 5.095838684 | -13.74710689 | 3.67E-35 | 4.40E-34 | 68.89447278 |
| C1orf112 | 1.105127679 | 6.397737109 | 13.74269445 | 3.82E-35 | 4.58E-34 | 68.85401621 |
| IL6 | -2.45633573 | 8.021533646 | -13.74226083 | 3.83E-35 | 4.59E-34 | 68.85004066 |
| KLF10 | -0.933964061 | 7.595223766 | -13.72866069 | 4.35E-35 | 5.20E-34 | 68.7253728 |
| FMO3 | -1.661320116 | 7.811906593 | -13.71876214 | 4.76E-35 | 5.69E-34 | 68.63466192 |
| B3GNT3 | 0.966130727 | 5.972702444 | 13.71808095 | 4.79E-35 | 5.72E-34 | 68.62842029 |
| PPP2CB | -0.78717381 | 8.990117088 | -13.71629724 | 4.87E-35 | 5.81E-34 | 68.61207688 |
| CDH1 | 0.952359167 | 10.28974416 | 13.71502087 | 4.93E-35 | 5.87E-34 | 68.60038241 |
| FAM49A | -0.87650096 | 6.792708492 | -13.6986952 | 5.73E-35 | 6.80E-34 | 68.45083465 |
| ID4 | -1.219981055 | 6.289642128 | -13.69461391 | 5.95E-35 | 7.06E-34 | 68.4134582 |
| CCNE1 | 1.190375295 | 5.877282641 | 13.67833197 | 6.91E-35 | 8.17E-34 | 68.26438539 |
| CHPT1 | -1.210918317 | 8.324342223 | -13.67816509 | 6.92E-35 | 8.17E-34 | 68.26285778 |
| RNF43 | 1.252754636 | 6.941597941 | 13.67660173 | 7.02E-35 | 8.28E-34 | 68.24854733 |
| IL7R | -1.478503682 | 9.434650508 | -13.67458672 | 7.15E-35 | 8.43E-34 | 68.2301034 |
| DDX56 | 0.653757556 | 9.032284261 | 13.66838834 | 7.57E-35 | 8.91E-34 | 68.1733737 |
| TSPAN32 | -0.784626861 | 6.300972707 | -13.66464514 | 7.84E-35 | 9.22E-34 | 68.13911887 |
| SPINT2 | 0.784634804 | 11.34812669 | 13.65729953 | 8.38E-35 | 9.85E-34 | 68.07190675 |
| MANEAL | 0.956888604 | 5.817438739 | 13.65707242 | 8.40E-35 | 9.86E-34 | 68.06982897 |
| BTN3A3 | -1.089050835 | 7.737515851 | -13.65307004 | 8.72E-35 | 1.02E-33 | 68.03321263 |
| CABLES2 | 0.808976785 | 6.848164356 | 13.64932675 | 9.02E-35 | 1.06E-33 | 67.99896994 |
| GADD45B | -1.225445164 | 9.021396043 | -13.64899066 | 9.05E-35 | 1.06E-33 | 67.99589564 |
| KLRD1 | -0.901155601 | 5.275154181 | -13.638366 | 9.98E-35 | 1.16E-33 | 67.89872207 |
| ELMO1 | -0.81404586 | 6.897636694 | -13.63787224 | 1.00E-34 | 1.17E-33 | 67.89420673 |
| SHE | -1.398287854 | 7.417404572 | -13.62084882 | 1.17E-34 | 1.36E-33 | 67.73856525 |
| SLC7A7 | -1.204659954 | 9.303937165 | -13.612425 | 1.27E-34 | 1.47E-33 | 67.6615725 |
| MCM8 | 0.852560409 | 6.948525245 | 13.60323831 | 1.38E-34 | 1.60E-33 | 67.57762576 |
| DPP6 | -0.680573788 | 4.665188239 | -13.59055913 | 1.55E-34 | 1.79E-33 | 67.46179691 |
| RDX | -0.779194313 | 8.613809997 | -13.5861912 | 1.61E-34 | 1.86E-33 | 67.42190296 |
| SNCA | -1.347817182 | 6.690461511 | -13.58278458 | 1.66E-34 | 1.92E-33 | 67.39079191 |
| EEF1A2 | 2.848916974 | 6.887009785 | 13.58109117 | 1.69E-34 | 1.95E-33 | 67.37532784 |
| AADAC | -2.036762658 | 5.933977625 | -13.5778221 | 1.74E-34 | 2.00E-33 | 67.34547685 |
| PPIL1 | 0.837147006 | 8.00955584 | 13.57674174 | 1.76E-34 | 2.02E-33 | 67.3356123 |
| DNAJB4 | -1.189578503 | 6.282589285 | -13.55913742 | 2.07E-34 | 2.37E-33 | 67.17490803 |
| KIAA1522 | 0.790973027 | 8.688596949 | 13.54673041 | 2.31E-34 | 2.66E-33 | 67.06169151 |
| KIF1C | -0.774224215 | 7.891270649 | -13.53593506 | 2.56E-34 | 2.93E-33 | 66.96321091 |
| ADM2 | 0.633348105 | 7.272472907 | 13.5341734 | 2.60E-34 | 2.98E-33 | 66.94714274 |
| TRIM46 | 1.101754616 | 5.696511426 | 13.53391505 | 2.60E-34 | 2.98E-33 | 66.94478633 |
| TENC1 | -0.762142645 | 6.818655234 | -13.53385334 | 2.60E-34 | 2.98E-33 | 66.94422349 |
| CTDSPL | -0.705931051 | 9.437403005 | -13.53083031 | 2.68E-34 | 3.06E-33 | 66.91665221 |
| GYG2 | 0.903124029 | 5.514850231 | 13.52883919 | 2.73E-34 | 3.11E-33 | 66.89849354 |
| PPME1 | 0.646018178 | 8.813494263 | 13.5286369 | 2.73E-34 | 3.11E-33 | 66.89664877 |
| ANK2 | -0.967361207 | 5.592165649 | -13.52737139 | 2.76E-34 | 3.15E-33 | 66.88510809 |
| ADCK5 | 0.830010035 | 6.779106 | 13.52562084 | 2.81E-34 | 3.19E-33 | 66.86914475 |
| CD69 | -1.683152968 | 7.688226141 | -13.52443345 | 2.84E-34 | 3.22E-33 | 66.85831728 |
| DNASE2B | -0.983199251 | 4.811652809 | -13.52300618 | 2.88E-34 | 3.26E-33 | 66.84530281 |
| CALU | 0.783224416 | 9.498641391 | 13.52194547 | 2.90E-34 | 3.29E-33 | 66.8356312 |
| DECR2 | 0.6671455 | 8.310635418 | 13.50859361 | 3.28E-34 | 3.71E-33 | 66.71391001 |
| PRF1 | -1.292094501 | 7.616993271 | -13.50534341 | 3.38E-34 | 3.82E-33 | 66.68428613 |
| EMP1 | -1.137141697 | 9.962150875 | -13.50364324 | 3.44E-34 | 3.87E-33 | 66.66879094 |
| MPZL1 | 0.677051783 | 8.142681819 | 13.50001069 | 3.55E-34 | 4.00E-33 | 66.63568655 |
| DAP | 0.661491554 | 9.754639314 | 13.49841321 | 3.60E-34 | 4.05E-33 | 66.62112926 |
| CYP27A1 | -1.258116824 | 9.595945271 | -13.49465083 | 3.73E-34 | 4.19E-33 | 66.58684638 |
| TMEM87B | 0.754017824 | 7.060834479 | 13.49208499 | 3.82E-34 | 4.29E-33 | 66.56346829 |
| ZNF408 | 0.651203125 | 6.660294263 | 13.49170418 | 3.83E-34 | 4.30E-33 | 66.55999876 |
| CYB5A | -1.133754157 | 11.34362091 | -13.48917266 | 3.92E-34 | 4.39E-33 | 66.53693509 |
| PARS2 | 0.676338856 | 6.418948838 | 13.48371495 | 4.12E-34 | 4.61E-33 | 66.48721731 |
| PLXNB3 | 1.224492699 | 5.670044537 | 13.47620072 | 4.42E-34 | 4.94E-33 | 66.41877676 |
| SAP18 | -0.818862638 | 8.390935723 | -13.47337706 | 4.53E-34 | 5.06E-33 | 66.39306197 |
| SAMHD1 | -0.874033243 | 6.921140162 | -13.46131763 | 5.06E-34 | 5.64E-33 | 66.28325926 |
| C6 | -1.75323953 | 5.825034851 | -13.45019546 | 5.60E-34 | 6.24E-33 | 66.18202102 |
| LGR4 | 1.187492064 | 6.211262915 | 13.43642746 | 6.36E-34 | 7.07E-33 | 66.05674014 |
| CYP27B1 | 1.355786498 | 6.045424428 | 13.42674831 | 6.94E-34 | 7.71E-33 | 65.96869249 |
| UBE2E2 | -0.787402785 | 8.986803543 | -13.42150232 | 7.28E-34 | 8.08E-33 | 65.92098104 |
| PDGFB | -0.685753869 | 6.665164181 | -13.41549526 | 7.70E-34 | 8.52E-33 | 65.86635585 |
| AGTPBP1 | -0.665884045 | 6.977724681 | -13.41041499 | 8.06E-34 | 8.92E-33 | 65.82016517 |
| RCE1 | 0.664599645 | 7.367720362 | 13.40870069 | 8.19E-34 | 9.05E-33 | 65.80457991 |
| MRGPRF | -1.040190601 | 6.719818032 | -13.40316038 | 8.61E-34 | 9.51E-33 | 65.75421585 |
| HK3 | -1.226291085 | 7.396420314 | -13.40038274 | 8.84E-34 | 9.74E-33 | 65.72896848 |
| SLC9A7 | 0.852780715 | 6.011790902 | 13.39620577 | 9.18E-34 | 1.01E-32 | 65.6910055 |
| PTGES | 1.394714394 | 7.369830519 | 13.39201292 | 9.54E-34 | 1.05E-32 | 65.65290239 |
| SBK1 | 0.742740211 | 6.27471854 | 13.38930868 | 9.78E-34 | 1.07E-32 | 65.62832951 |
| EMP3 | -0.934216314 | 10.24266072 | -13.38478396 | 1.02E-33 | 1.12E-32 | 65.58721821 |
| ZNF217 | 0.70744665 | 8.754373423 | 13.3821285 | 1.04E-33 | 1.15E-32 | 65.56309319 |
| B3GNT1 | -1.15888757 | 7.13830959 | -13.36953004 | 1.17E-33 | 1.28E-32 | 65.44865859 |
| TACC3 | 0.973355135 | 7.218192245 | 13.36585064 | 1.21E-33 | 1.33E-32 | 65.41524502 |
| WDR77 | 0.704888455 | 7.300001069 | 13.36152423 | 1.26E-33 | 1.38E-32 | 65.37595994 |
| ARHGAP30 | -0.852809728 | 7.386785949 | -13.36085998 | 1.27E-33 | 1.39E-32 | 65.36992879 |
| CLASP2 | -0.870756197 | 7.235892439 | -13.35992127 | 1.28E-33 | 1.40E-32 | 65.36140579 |
| PITX1 | 2.279021332 | 6.663437088 | 13.35861124 | 1.29E-33 | 1.41E-32 | 65.34951181 |
| EGFL7 | -1.024324686 | 7.405099681 | -13.35733484 | 1.31E-33 | 1.43E-32 | 65.33792345 |
| BIK | 1.334114289 | 6.411248021 | 13.35653589 | 1.32E-33 | 1.44E-32 | 65.3306701 |
| MAD2L1 | 1.274477843 | 5.914487766 | 13.35310417 | 1.36E-33 | 1.48E-32 | 65.29951652 |
| SETDB1 | 0.628511692 | 8.072029577 | 13.34223458 | 1.50E-33 | 1.63E-32 | 65.20085994 |
| TRAK2 | -0.63314373 | 9.234913184 | -13.33986506 | 1.54E-33 | 1.66E-32 | 65.1793571 |
| LAMA4 | -1.023791488 | 8.208003237 | -13.33689609 | 1.58E-33 | 1.71E-32 | 65.15241631 |
| NMU | 2.211239008 | 5.604921537 | 13.33673238 | 1.58E-33 | 1.71E-32 | 65.1509308 |
| EPS15 | -0.612079018 | 8.835640077 | -13.33393996 | 1.62E-33 | 1.75E-32 | 65.12559415 |
| XRCC3 | 0.879269634 | 5.990844694 | 13.31989993 | 1.84E-33 | 1.99E-32 | 64.99823254 |
| LEPREL1 | -1.750641298 | 8.975996697 | -13.30432158 | 2.12E-33 | 2.29E-32 | 64.85697291 |
| FGF14 | -0.821625572 | 4.75307684 | -13.29855132 | 2.24E-33 | 2.41E-32 | 64.80466509 |
| SLC2A5 | 1.326290479 | 6.377306338 | 13.29254039 | 2.36E-33 | 2.54E-32 | 64.75018432 |
| AQP9 | -1.641598592 | 8.253994777 | -13.27936643 | 2.67E-33 | 2.87E-32 | 64.6308119 |
| PREB | 0.593693219 | 8.350387604 | 13.27700436 | 2.72E-33 | 2.93E-32 | 64.60941307 |
| SFTPD | -2.182414487 | 11.08366397 | -13.27364488 | 2.81E-33 | 3.01E-32 | 64.57898081 |
| LAMC3 | -1.285077348 | 7.952354819 | -13.271374 | 2.87E-33 | 3.07E-32 | 64.55841144 |
| DGCR5 | 1.260796555 | 5.85815717 | 13.26549858 | 3.02E-33 | 3.24E-32 | 64.50519826 |
| MGP | -1.317976916 | 10.85725673 | -13.26479936 | 3.04E-33 | 3.26E-32 | 64.49886607 |
| RPN2 | 0.74333847 | 10.50301083 | 13.26239576 | 3.11E-33 | 3.32E-32 | 64.47709975 |
| PHKA1 | 1.051524206 | 6.370582965 | 13.25360163 | 3.37E-33 | 3.60E-32 | 64.39747488 |
| IL4I1 | 0.896787994 | 6.196108686 | 13.25200975 | 3.42E-33 | 3.65E-32 | 64.38306357 |
| CSF3R | -1.34335805 | 7.731578343 | -13.25134021 | 3.44E-33 | 3.67E-32 | 64.37700233 |
| PLA2G4C | -1.023026199 | 7.424264441 | -13.25022637 | 3.48E-33 | 3.70E-32 | 64.3669193 |
| RAD51 | 0.818409386 | 6.265962824 | 13.23767939 | 3.90E-33 | 4.14E-32 | 64.25335883 |
| POLR2H | 0.672237843 | 10.1307738 | 13.23522687 | 3.98E-33 | 4.23E-32 | 64.23116612 |
| TRHDE | -0.828737108 | 4.234234197 | -13.23297666 | 4.07E-33 | 4.32E-32 | 64.21080543 |
| HCN3 | 0.894537456 | 6.250911878 | 13.21096838 | 4.97E-33 | 5.26E-32 | 64.01173341 |
| CENPE | 1.210951506 | 5.535048489 | 13.20757098 | 5.12E-33 | 5.42E-32 | 63.98101362 |
| ILF2 | 0.64554575 | 10.96108751 | 13.20459753 | 5.26E-33 | 5.56E-32 | 63.95412973 |
| CXCL12 | -1.456300372 | 8.068139176 | -13.16013362 | 7.88E-33 | 8.31E-32 | 63.55238281 |
| SYT11 | -0.846187671 | 7.911189277 | -13.15775337 | 8.05E-33 | 8.48E-32 | 63.53089053 |
| TSPAN12 | -1.318813418 | 7.341295987 | -13.15046082 | 8.61E-33 | 9.05E-32 | 63.46505207 |
| ESPN | 1.44006698 | 6.349030412 | 13.14872982 | 8.74E-33 | 9.19E-32 | 63.44942633 |
| RASGRF1 | -0.776732272 | 5.476030239 | -13.14515879 | 9.03E-33 | 9.48E-32 | 63.41719294 |
| ATP10B | 1.872742411 | 5.761760372 | 13.13927887 | 9.52E-33 | 9.98E-32 | 63.36412573 |
| DPT | -1.666484119 | 8.102700888 | -13.13233207 | 1.01E-32 | 1.06E-31 | 63.3014412 |
| ZFP36L2 | -1.092858014 | 8.896900037 | -13.12841429 | 1.05E-32 | 1.10E-31 | 63.26609455 |
| SULF1 | 1.660132978 | 8.984338202 | 13.12559997 | 1.08E-32 | 1.13E-31 | 63.24070576 |
| EPHB3 | 0.910792235 | 6.710803931 | 13.12123511 | 1.12E-32 | 1.17E-31 | 63.20133324 |
| MYOM2 | -1.099967479 | 6.224334229 | -13.12059765 | 1.13E-32 | 1.18E-31 | 63.19558356 |
| OASL | -1.108899955 | 6.529827649 | -13.11938385 | 1.14E-32 | 1.19E-31 | 63.18463572 |
| TMEM106C | 0.820699953 | 9.495199322 | 13.1155471 | 1.18E-32 | 1.23E-31 | 63.15003281 |
| LCP1 | -0.956893285 | 10.7954333 | -13.11361006 | 1.20E-32 | 1.25E-31 | 63.13256442 |
| NCALD | -0.879097858 | 7.459151907 | -13.1109538 | 1.23E-32 | 1.28E-31 | 63.10861164 |
| JMJD4 | 0.620057476 | 7.218283146 | 13.1085421 | 1.26E-32 | 1.31E-31 | 63.08686575 |
| PPP3CC | -0.761031398 | 7.882243662 | -13.10033373 | 1.36E-32 | 1.41E-31 | 63.01286345 |
| ARRDC4 | -1.181090324 | 7.269442237 | -13.09971547 | 1.36E-32 | 1.41E-31 | 63.00729029 |
| LONRF1 | -0.753090291 | 7.474928431 | -13.09765942 | 1.39E-32 | 1.44E-31 | 62.98875703 |
| CD59 | -0.875892216 | 8.850198691 | -13.095791 | 1.41E-32 | 1.46E-31 | 62.97191605 |
| SCG5 | 1.94413397 | 6.634143051 | 13.07988255 | 1.63E-32 | 1.68E-31 | 62.82856213 |
| PCDHB4 | -1.099172357 | 5.685695646 | -13.07889857 | 1.65E-32 | 1.69E-31 | 62.81969739 |
| CDCA2 | 1.081870856 | 5.34307934 | 13.07829037 | 1.66E-32 | 1.70E-31 | 62.8142183 |
| ARRB1 | -0.880576635 | 8.84671996 | -13.07079037 | 1.77E-32 | 1.82E-31 | 62.74666021 |
| MMP28 | -1.389994967 | 7.09370583 | -13.05129545 | 2.11E-32 | 2.17E-31 | 62.57112317 |
| PCNXL2 | 0.675319032 | 5.815624223 | 13.04826876 | 2.17E-32 | 2.23E-31 | 62.54387898 |
| HCK | -1.10085451 | 8.725338112 | -13.04152193 | 2.31E-32 | 2.37E-31 | 62.48315728 |
| SNX25 | -1.089178997 | 8.016157399 | -13.03300039 | 2.49E-32 | 2.55E-31 | 62.40648012 |
| PTK6 | 1.146259436 | 6.815936338 | 13.01955279 | 2.82E-32 | 2.88E-31 | 62.28551657 |
| ATAD2 | 1.232833373 | 6.66990104 | 13.01923939 | 2.83E-32 | 2.89E-31 | 62.28269807 |
| LRRC20 | 0.638117663 | 6.894301037 | 13.01827214 | 2.85E-32 | 2.91E-31 | 62.27399937 |
| PLA1A | -1.451736501 | 6.722999691 | -13.00648471 | 3.17E-32 | 3.23E-31 | 62.16801243 |
| ATOH8 | -1.400445081 | 7.320166654 | -12.99897759 | 3.39E-32 | 3.46E-31 | 62.10053097 |
| DDR1 | 0.664268336 | 8.164596628 | 12.99534715 | 3.51E-32 | 3.57E-31 | 62.06790235 |
| NXPH3 | -0.68391697 | 5.857154612 | -12.9883145 | 3.74E-32 | 3.79E-31 | 62.00470611 |
| PLK1 | 0.945498428 | 6.689643806 | 12.98336255 | 3.91E-32 | 3.96E-31 | 61.96021514 |
| RUNX1T1 | -0.717790818 | 5.482003428 | -12.97858124 | 4.08E-32 | 4.14E-31 | 61.91726351 |
| ACTG2 | -1.480763718 | 9.590993832 | -12.97613747 | 4.17E-32 | 4.22E-31 | 61.89531284 |
| MTA3 | 0.691175482 | 7.9188865 | 12.96918358 | 4.44E-32 | 4.49E-31 | 61.83285965 |
| HEY1 | -1.262685113 | 7.75432884 | -12.96161614 | 4.76E-32 | 4.80E-31 | 61.76491068 |
| DOCK9 | -1.068856561 | 7.934788096 | -12.95509499 | 5.04E-32 | 5.08E-31 | 61.70636869 |
| GBP4 | -1.383476317 | 8.39283729 | -12.95235695 | 5.17E-32 | 5.20E-31 | 61.681792 |
| NLN | 0.775545137 | 6.182802287 | 12.94786227 | 5.38E-32 | 5.41E-31 | 61.64145203 |
| ZNF423 | -0.86701534 | 5.618331293 | -12.93358203 | 6.13E-32 | 6.14E-31 | 61.51332179 |
| SLC29A4 | 1.217256731 | 6.640141431 | 12.93213796 | 6.21E-32 | 6.21E-31 | 61.50036784 |
| NSUN2 | 0.660118916 | 9.830259383 | 12.92561118 | 6.58E-32 | 6.58E-31 | 61.44182662 |
| SELE | -1.477645663 | 5.396044755 | -12.92362385 | 6.70E-32 | 6.70E-31 | 61.42400376 |
| FAM43A | -1.035528565 | 8.139191372 | -12.91031956 | 7.55E-32 | 7.54E-31 | 61.30471491 |
| DNASE1 | 0.898096413 | 5.785066926 | 12.90975796 | 7.59E-32 | 7.57E-31 | 61.29968056 |
| PDE7B | -1.220201533 | 6.52169833 | -12.90876586 | 7.66E-32 | 7.63E-31 | 61.29078727 |
| CXCL13 | 2.200419944 | 6.768227189 | 12.90760343 | 7.74E-32 | 7.70E-31 | 61.28036739 |
| FOLR3 | -1.840675575 | 6.301247899 | -12.90516693 | 7.91E-32 | 7.87E-31 | 61.25852823 |
| PI16 | -1.729785607 | 6.709289588 | -12.90178519 | 8.16E-32 | 8.10E-31 | 61.22821916 |
| RCC2 | 0.5923211 | 10.4708638 | 12.89491231 | 8.68E-32 | 8.60E-31 | 61.16662995 |
| MMP1 | 2.508652424 | 6.853026912 | 12.88787153 | 9.25E-32 | 9.16E-31 | 61.10354942 |
| EME1 | 0.69913016 | 5.333769024 | 12.87930172 | 9.99E-32 | 9.88E-31 | 61.0267878 |
| TNS1 | -0.881305769 | 8.505853152 | -12.87546459 | 1.03E-31 | 1.02E-30 | 60.99242424 |
| FCER1A | -1.851892695 | 7.067357319 | -12.86910274 | 1.10E-31 | 1.08E-30 | 60.93545931 |
| FBXO32 | 1.047494368 | 6.812013681 | 12.86453164 | 1.14E-31 | 1.12E-30 | 60.8945358 |
| RHBDD3 | 0.727644913 | 6.977825396 | 12.86292775 | 1.16E-31 | 1.14E-30 | 60.88017805 |
| PXK | -0.692108826 | 6.429313798 | -12.86057978 | 1.18E-31 | 1.16E-30 | 60.85916068 |
| PTPRD | -0.992496653 | 5.810824085 | -12.85322962 | 1.26E-31 | 1.24E-30 | 60.79337692 |
| ADCY9 | -0.843871538 | 7.782962846 | -12.84344977 | 1.38E-31 | 1.36E-30 | 60.70587033 |
| PLAC8 | -1.889571559 | 8.78896896 | -12.83831099 | 1.45E-31 | 1.42E-30 | 60.65990078 |
| P2RX1 | -0.919794801 | 6.472288207 | -12.83113103 | 1.54E-31 | 1.51E-30 | 60.59568369 |
| FOS | -1.536702956 | 11.13219435 | -12.82927296 | 1.57E-31 | 1.53E-30 | 60.57906759 |
| EIF1B | -0.748610568 | 9.11900142 | -12.82437986 | 1.64E-31 | 1.60E-30 | 60.53531454 |
| RFX2 | -1.024573956 | 6.788441734 | -12.82437903 | 1.64E-31 | 1.60E-30 | 60.53530719 |
| TMEM79 | 0.872947539 | 7.342737173 | 12.8238805 | 1.65E-31 | 1.61E-30 | 60.53084977 |
| BRI3BP | 0.891318811 | 7.355723668 | 12.82171387 | 1.68E-31 | 1.64E-30 | 60.51147868 |
| SLC7A5 | 1.454493768 | 8.606970221 | 12.81567793 | 1.77E-31 | 1.73E-30 | 60.45752009 |
| EFCAB1 | -1.914435038 | 5.639904261 | -12.81490686 | 1.78E-31 | 1.74E-30 | 60.45062774 |
| GJA5 | -0.898546202 | 6.371547657 | -12.80852628 | 1.89E-31 | 1.84E-30 | 60.3936005 |
| CLDN11 | -1.437074631 | 7.125792676 | -12.8012425 | 2.02E-31 | 1.96E-30 | 60.32851443 |
| COMP | 2.385845103 | 7.573099678 | 12.79934598 | 2.05E-31 | 1.99E-30 | 60.31156998 |
| SMYD5 | 0.609544479 | 6.561464782 | 12.79923024 | 2.05E-31 | 1.99E-30 | 60.31053591 |
| CHMP4B | 0.828223994 | 9.399786715 | 12.79776119 | 2.08E-31 | 2.01E-30 | 60.29741145 |
| ACSL4 | -0.685649738 | 7.300389779 | -12.78487934 | 2.34E-31 | 2.26E-30 | 60.18235043 |
| GNPNAT1 | 0.996031949 | 6.926945378 | 12.78241945 | 2.39E-31 | 2.31E-30 | 60.16038388 |
| GTSE1 | 0.954204245 | 5.858959112 | 12.75958158 | 2.93E-31 | 2.82E-30 | 59.95652379 |
| BMP6 | -1.514706196 | 6.902500423 | -12.74996767 | 3.20E-31 | 3.07E-30 | 59.87074937 |
| CDH19 | -0.952847549 | 4.41340417 | -12.74469074 | 3.35E-31 | 3.21E-30 | 59.82368004 |
| COL5A2 | 1.362837836 | 9.395361423 | 12.74058384 | 3.48E-31 | 3.33E-30 | 59.78705251 |
| CUL7 | 0.727558989 | 6.726840316 | 12.73938439 | 3.51E-31 | 3.37E-30 | 59.77635605 |
| TRAF2 | 0.793053605 | 7.148620457 | 12.73849299 | 3.54E-31 | 3.39E-30 | 59.76840699 |
| SLC39A4 | 0.657095145 | 6.882723785 | 12.73708755 | 3.59E-31 | 3.43E-30 | 59.75587438 |
| ARHGAP29 | -0.945778494 | 6.504424886 | -12.73635922 | 3.61E-31 | 3.45E-30 | 59.74937995 |
| ACP6 | 0.705675407 | 7.857250019 | 12.73570091 | 3.63E-31 | 3.47E-30 | 59.74351001 |
| LRRC45 | 0.827044711 | 6.518317806 | 12.72971007 | 3.83E-31 | 3.65E-30 | 59.6900971 |
| SLC27A3 | -0.908830668 | 8.799033731 | -12.72318325 | 4.06E-31 | 3.87E-30 | 59.63191701 |
| RAB11FIP1 | -0.757453346 | 8.293376194 | -12.71642701 | 4.32E-31 | 4.11E-30 | 59.5717043 |
| PKIA | -0.924741617 | 6.018132226 | -12.7090615 | 4.61E-31 | 4.38E-30 | 59.50607633 |
| STYK1 | 0.891563615 | 4.862501457 | 12.70600072 | 4.74E-31 | 4.50E-30 | 59.47880869 |
| CITED2 | -0.982929996 | 10.09855321 | -12.70463581 | 4.80E-31 | 4.55E-30 | 59.46664998 |
| EFNB2 | -1.024992279 | 9.27931066 | -12.70085193 | 4.96E-31 | 4.70E-30 | 59.43294555 |
| ADIPOR2 | -0.623063478 | 9.556875303 | -12.69972138 | 5.01E-31 | 4.75E-30 | 59.42287614 |
| LMNB2 | 0.753776713 | 8.631443864 | 12.69709163 | 5.13E-31 | 4.86E-30 | 59.39945512 |
| PAQR5 | -1.258336041 | 6.204230085 | -12.69645902 | 5.16E-31 | 4.88E-30 | 59.39382135 |
| CHRM1 | -0.727386601 | 4.832734154 | -12.69615952 | 5.18E-31 | 4.89E-30 | 59.39115409 |
| VSIG2 | -1.72199166 | 8.297714423 | -12.69441961 | 5.26E-31 | 4.96E-30 | 59.37565973 |
| ZFPM2 | -1.106740603 | 6.17739012 | -12.68788313 | 5.58E-31 | 5.26E-30 | 59.31745821 |
| SOX18 | -1.371484077 | 8.600289691 | -12.68459049 | 5.74E-31 | 5.41E-30 | 59.28814471 |
| SCN2B | -1.022217468 | 5.133947218 | -12.67178567 | 6.44E-31 | 6.06E-30 | 59.174176 |
| ZNF593 | 0.60212853 | 9.001859269 | 12.66137638 | 7.07E-31 | 6.65E-30 | 59.0815628 |
| FRAS1 | -1.219979573 | 6.047607048 | -12.65522987 | 7.47E-31 | 7.01E-30 | 59.02689067 |
| SNX2 | -0.655991547 | 9.663699715 | -12.64872528 | 7.92E-31 | 7.43E-30 | 58.96904515 |
| CCDC34 | 0.911513351 | 6.31469558 | 12.64435327 | 8.23E-31 | 7.71E-30 | 58.9301715 |
| PLK4 | 1.081127124 | 6.039182646 | 12.64095199 | 8.49E-31 | 7.94E-30 | 58.89993285 |
| LEPRE1 | 0.59765917 | 7.769851149 | 12.63412718 | 9.02E-31 | 8.43E-30 | 58.83926764 |
| ACE | -0.613682474 | 5.761770545 | -12.6281185 | 9.52E-31 | 8.89E-30 | 58.78586787 |
| TMEM62 | 0.718865747 | 8.903033657 | 12.62701337 | 9.61E-31 | 8.97E-30 | 58.77604764 |
| FUT8 | 0.681006661 | 7.043146524 | 12.62203713 | 1.00E-30 | 9.37E-30 | 58.73183266 |
| GRAMD2 | -1.34967764 | 7.512822907 | -12.61627054 | 1.06E-30 | 9.84E-30 | 58.68060414 |
| RNASEH2A | 0.832044046 | 7.567669301 | 12.61626137 | 1.06E-30 | 9.84E-30 | 58.68052267 |
| REEP6 | 1.340405967 | 7.230559351 | 12.61552778 | 1.07E-30 | 9.89E-30 | 58.67400642 |
| HSDL2 | -0.82208928 | 8.198651721 | -12.5956871 | 1.27E-30 | 1.18E-29 | 58.49782522 |
| SIX1 | 1.737252692 | 6.348167327 | 12.59121791 | 1.32E-30 | 1.23E-29 | 58.45815532 |
| TFB2M | 0.62749713 | 8.070725686 | 12.58288074 | 1.43E-30 | 1.32E-29 | 58.38416734 |
| CP | 2.027256135 | 7.463743441 | 12.5755056 | 1.52E-30 | 1.41E-29 | 58.31873362 |
| HSD11B1 | -1.244676362 | 6.581022497 | -12.57538454 | 1.52E-30 | 1.41E-29 | 58.31765974 |
| ECE2 | 0.661094094 | 5.740097197 | 12.56326856 | 1.70E-30 | 1.57E-29 | 58.21019885 |
| KIAA1683 | -1.181380975 | 6.907214997 | -12.55981064 | 1.75E-30 | 1.62E-29 | 58.17953709 |
| SORBS1 | -0.730016096 | 6.313420449 | -12.55626806 | 1.81E-30 | 1.67E-29 | 58.14812826 |
| HSF2BP | 0.852495446 | 5.277151178 | 12.55529794 | 1.82E-30 | 1.68E-29 | 58.13952771 |
| CLDN9 | 1.024391991 | 5.960140298 | 12.54859069 | 1.94E-30 | 1.78E-29 | 58.08007251 |
| EXOSC5 | 0.664610718 | 7.971226412 | 12.54806154 | 1.94E-30 | 1.79E-29 | 58.07538249 |
| PPM1D | -0.672215107 | 7.638220372 | -12.54802524 | 1.95E-30 | 1.79E-29 | 58.07506077 |
| TYROBP | -1.201185541 | 11.04702666 | -12.53812534 | 2.12E-30 | 1.95E-29 | 57.98733035 |
| LHX6 | -0.7754109 | 7.005834181 | -12.53632795 | 2.16E-30 | 1.98E-29 | 57.97140538 |
| BPHL | 0.722296114 | 8.24398954 | 12.53246331 | 2.23E-30 | 2.04E-29 | 57.93716769 |
| NUP155 | 0.698840034 | 7.35649771 | 12.53200417 | 2.24E-30 | 2.05E-29 | 57.93310034 |
| EDN1 | -1.314400664 | 8.81397938 | -12.52650416 | 2.36E-30 | 2.15E-29 | 57.8843828 |
| PTPRO | -1.016316502 | 6.261752739 | -12.50608935 | 2.83E-30 | 2.58E-29 | 57.70363124 |
| SLA | -0.878579862 | 7.584446375 | -12.50533784 | 2.85E-30 | 2.59E-29 | 57.69697965 |
| PDIA6 | 0.800591476 | 9.837550955 | 12.49702593 | 3.06E-30 | 2.79E-29 | 57.62342331 |
| RPL39L | 1.180797475 | 7.263126622 | 12.49476741 | 3.13E-30 | 2.84E-29 | 57.60343993 |
| NR4A1 | -1.011399679 | 6.55849254 | -12.48897922 | 3.29E-30 | 2.99E-29 | 57.55223305 |
| WDR12 | 0.754249262 | 7.962544894 | 12.47798024 | 3.63E-30 | 3.29E-29 | 57.45495448 |
| JUN | -0.959170542 | 10.50232667 | -12.47628079 | 3.69E-30 | 3.34E-29 | 57.43992722 |
| RHBDL2 | 1.122982958 | 6.857335295 | 12.47202167 | 3.83E-30 | 3.46E-29 | 57.40226985 |
| PGC | -3.100833492 | 9.75521312 | -12.47112551 | 3.86E-30 | 3.49E-29 | 57.39434707 |
| FBP1 | -1.274172634 | 10.51572761 | -12.46973007 | 3.91E-30 | 3.53E-29 | 57.38201072 |
| STK32A | 1.512874752 | 5.700160101 | 12.46924217 | 3.92E-30 | 3.54E-29 | 57.37769753 |
| CA3 | -0.978901228 | 4.988523941 | -12.46057067 | 4.24E-30 | 3.82E-29 | 57.30105148 |
| NUP210 | 0.843192601 | 7.602949343 | 12.4560747 | 4.41E-30 | 3.97E-29 | 57.26132098 |
| DISP1 | -1.047997469 | 7.821095718 | -12.44959187 | 4.67E-30 | 4.20E-29 | 57.20404341 |
| ENC1 | 0.95633389 | 8.573793572 | 12.4456662 | 4.84E-30 | 4.35E-29 | 57.16936507 |
| GPR4 | -0.892991011 | 7.055638516 | -12.44288999 | 4.96E-30 | 4.45E-29 | 57.14484345 |
| FGD3 | -0.860803228 | 8.595842742 | -12.43914156 | 5.13E-30 | 4.60E-29 | 57.11173821 |
| OSBPL11 | -0.871320161 | 7.010581729 | -12.43427264 | 5.35E-30 | 4.79E-29 | 57.06874321 |
| ELMO3 | 0.892973019 | 7.705779412 | 12.43112976 | 5.51E-30 | 4.92E-29 | 57.04099378 |
| MEIS2 | -0.6140865 | 6.274513981 | -12.41968003 | 6.10E-30 | 5.45E-29 | 56.93992542 |
| ANXA6 | -0.807670238 | 8.181789109 | -12.41939812 | 6.11E-30 | 5.45E-29 | 56.9374374 |
| GRHL2 | 0.955284824 | 7.360856194 | 12.41903783 | 6.13E-30 | 5.47E-29 | 56.93425775 |
| PLCE1 | -1.167026586 | 6.982357662 | -12.41803378 | 6.18E-30 | 5.51E-29 | 56.92539698 |
| SERPINH1 | 0.768782081 | 10.01431181 | 12.40968165 | 6.66E-30 | 5.93E-29 | 56.85170028 |
| P2RY6 | 0.934556788 | 6.408715457 | 12.40739456 | 6.80E-30 | 6.05E-29 | 56.83152336 |
| DHTKD1 | 0.819137468 | 7.308093359 | 12.40115661 | 7.18E-30 | 6.38E-29 | 56.77649943 |
| CBX4 | 0.693962714 | 7.999304492 | 12.39741721 | 7.43E-30 | 6.59E-29 | 56.74352037 |
| ACOT11 | 0.645531873 | 6.210716976 | 12.3954915 | 7.55E-30 | 6.70E-29 | 56.72653847 |
| LPXN | -0.921346142 | 9.614286883 | -12.39155576 | 7.82E-30 | 6.93E-29 | 56.69183466 |
| LBH | -0.727069221 | 7.829101758 | -12.39096394 | 7.86E-30 | 6.96E-29 | 56.68661665 |
| SCGB1A1 | -3.711810246 | 10.28704249 | -12.38141742 | 8.56E-30 | 7.57E-29 | 56.60246007 |
| ROPN1L | -1.817415939 | 6.64389387 | -12.380109 | 8.66E-30 | 7.65E-29 | 56.59092795 |
| RRBP1 | 0.870259388 | 8.834134199 | 12.37490649 | 9.07E-30 | 8.00E-29 | 56.54507921 |
| HOMER3 | 0.732750822 | 7.563484519 | 12.3673657 | 9.69E-30 | 8.55E-29 | 56.47863816 |
| SAC3D1 | 0.751488077 | 8.288953013 | 12.36305108 | 1.01E-29 | 8.87E-29 | 56.44063023 |
| ID1 | -1.392805716 | 9.380108136 | -12.36007329 | 1.03E-29 | 9.10E-29 | 56.41440183 |
| NAALADL1 | -0.741939067 | 6.611960529 | -12.35673904 | 1.07E-29 | 9.37E-29 | 56.38503688 |
| CPSF6 | 0.6720287 | 7.424834774 | 12.35584901 | 1.07E-29 | 9.44E-29 | 56.37719888 |
| SOCS3 | -1.236563258 | 7.929720904 | -12.34413949 | 1.19E-29 | 1.05E-28 | 56.27410224 |
| TMEM45B | 1.647693787 | 7.782117548 | 12.3432093 | 1.20E-29 | 1.05E-28 | 56.26591414 |
| SSFA2 | -1.044078021 | 7.732029029 | -12.34303513 | 1.20E-29 | 1.05E-28 | 56.26438107 |
| SLCO2B1 | -1.01499473 | 9.157747383 | -12.33336426 | 1.31E-29 | 1.15E-28 | 56.17926832 |
| PLCXD3 | -1.194795701 | 5.24790896 | -12.33238872 | 1.32E-29 | 1.16E-28 | 56.17068416 |
| ITGA8 | -1.482803849 | 7.021277133 | -12.3279295 | 1.37E-29 | 1.20E-28 | 56.13144973 |
| IFNGR1 | -0.750291055 | 10.30774274 | -12.32580415 | 1.40E-29 | 1.22E-28 | 56.11275191 |
| CRY2 | -0.628197187 | 8.299549673 | -12.32531464 | 1.41E-29 | 1.23E-28 | 56.10844569 |
| SLC12A8 | 0.993230944 | 7.332011364 | 12.32172924 | 1.45E-29 | 1.27E-28 | 56.07690677 |
| CDKN2B | -0.895164909 | 6.985716479 | -12.32073975 | 1.47E-29 | 1.28E-28 | 56.06820335 |
| SLC9A9 | -0.96964113 | 7.23497521 | -12.31764769 | 1.51E-29 | 1.31E-28 | 56.04100819 |
| CHN1 | -0.734729249 | 7.373476396 | -12.31292579 | 1.57E-29 | 1.37E-28 | 55.99948389 |
| SPINK1 | 3.310000107 | 7.271209128 | 12.30924959 | 1.62E-29 | 1.41E-28 | 55.96716017 |
| GAB3 | -0.830757503 | 6.683130508 | -12.30635629 | 1.66E-29 | 1.44E-28 | 55.94172306 |
| HEBP1 | -0.624623028 | 9.848375899 | -12.29316379 | 1.87E-29 | 1.62E-28 | 55.82577092 |
| KRTCAP3 | 0.940452955 | 8.191073697 | 12.29227837 | 1.88E-29 | 1.63E-28 | 55.81799066 |
| RGS5 | -1.294175531 | 8.474887989 | -12.2890366 | 1.94E-29 | 1.68E-28 | 55.78950694 |
| EIF4G3 | 0.594500568 | 8.526234803 | 12.28159199 | 2.07E-29 | 1.79E-28 | 55.72410737 |
| WFDC2 | 1.193583379 | 6.94422662 | 12.28143758 | 2.07E-29 | 1.79E-28 | 55.72275104 |
| LAMA2 | -1.05265283 | 7.337179383 | -12.27053258 | 2.28E-29 | 1.97E-28 | 55.62698367 |
| NEDD4L | -1.151100561 | 9.038321678 | -12.26002206 | 2.51E-29 | 2.16E-28 | 55.53471517 |
| HSPB7 | -1.052582729 | 5.960685673 | -12.25832031 | 2.54E-29 | 2.19E-28 | 55.51977927 |
| MCOLN3 | -1.148193522 | 6.159207625 | -12.25824975 | 2.55E-29 | 2.19E-28 | 55.51915997 |
| TSEN54 | 0.639687969 | 7.736795641 | 12.25604351 | 2.60E-29 | 2.23E-28 | 55.49979767 |
| SMAD7 | -0.845219761 | 7.343893122 | -12.25089738 | 2.72E-29 | 2.33E-28 | 55.45464016 |
| SYT12 | 1.416401908 | 5.690137644 | 12.24485116 | 2.87E-29 | 2.46E-28 | 55.40159473 |
| LYPD1 | 1.026233863 | 5.98791283 | 12.24087806 | 2.97E-29 | 2.54E-28 | 55.36674358 |
| FAP | 1.415057172 | 7.241201452 | 12.23723292 | 3.06E-29 | 2.62E-28 | 55.33477357 |
| CD244 | -0.628504583 | 5.235124077 | -12.23266785 | 3.19E-29 | 2.73E-28 | 55.29474103 |
| STK10 | -0.66929046 | 7.519387902 | -12.23240689 | 3.20E-29 | 2.73E-28 | 55.29245274 |
| JDP2 | -0.669874386 | 7.142589088 | -12.23174752 | 3.22E-29 | 2.75E-28 | 55.28667108 |
| ASPHD1 | 0.721548868 | 5.372052191 | 12.22659188 | 3.37E-29 | 2.87E-28 | 55.24146883 |
| SLC6A16 | -0.802224983 | 5.768690777 | -12.22641947 | 3.37E-29 | 2.88E-28 | 55.23995739 |
| ST8SIA1 | -0.592452878 | 5.013617165 | -12.22349667 | 3.46E-29 | 2.95E-28 | 55.21433541 |
| ENPP2 | -1.287680258 | 9.404704274 | -12.22259442 | 3.49E-29 | 2.97E-28 | 55.20642669 |
| SLC15A3 | -0.96219187 | 9.671644854 | -12.2058587 | 4.04E-29 | 3.44E-28 | 55.05977326 |
| IQCC | 0.68393595 | 6.415761662 | 12.18954508 | 4.67E-29 | 3.97E-28 | 54.91690264 |
| C1QA | -1.311553944 | 9.538224104 | -12.18918719 | 4.68E-29 | 3.97E-28 | 54.91376922 |
| ZYX | -0.686095354 | 9.922003239 | -12.17865849 | 5.14E-29 | 4.36E-28 | 54.82160671 |
| MCM6 | 0.833752703 | 9.028428505 | 12.16826871 | 5.63E-29 | 4.77E-28 | 54.7306943 |
| SDC2 | -1.05668219 | 7.602515287 | -12.16492109 | 5.80E-29 | 4.91E-28 | 54.70140929 |
| DAB2 | -0.845236319 | 8.824873654 | -12.14783233 | 6.74E-29 | 5.69E-28 | 54.55197118 |
| FRK | 1.149674852 | 5.827664213 | 12.1433691 | 7.01E-29 | 5.91E-28 | 54.51295619 |
| APBB2 | -0.739239378 | 6.762105508 | -12.14271783 | 7.05E-29 | 5.94E-28 | 54.50726369 |
| MAP1LC3C | -1.015787609 | 5.864771761 | -12.12943135 | 7.92E-29 | 6.67E-28 | 54.39116083 |
| LAYN | -0.923030875 | 7.303724199 | -12.12731443 | 8.07E-29 | 6.79E-28 | 54.37266744 |
| DONSON | 0.862533834 | 6.625612497 | 12.11922579 | 8.66E-29 | 7.28E-28 | 54.30201817 |
| JAZF1 | -0.831322258 | 7.379862415 | -12.11406818 | 9.07E-29 | 7.61E-28 | 54.25698046 |
| HEATR1 | 0.611571502 | 8.68255804 | 12.10111556 | 1.02E-28 | 8.52E-28 | 54.14391176 |
| EFEMP2 | -0.888922614 | 9.293241848 | -12.09387089 | 1.08E-28 | 9.06E-28 | 54.08069334 |
| GPR37 | 1.697271677 | 5.520752157 | 12.08584917 | 1.16E-28 | 9.72E-28 | 54.01071372 |
| PPP1R3C | -1.314969578 | 8.026261306 | -12.08267947 | 1.19E-28 | 9.98E-28 | 53.98306764 |
| LRCH2 | -1.250103747 | 6.36884592 | -12.08106147 | 1.21E-28 | 1.01E-27 | 53.96895671 |
| MYOZ1 | -1.086318625 | 6.314406915 | -12.07719423 | 1.25E-28 | 1.05E-27 | 53.93523301 |
| KCNJ15 | -1.22500135 | 6.732562162 | -12.0589491 | 1.47E-28 | 1.22E-27 | 53.77619359 |
| MPP1 | -0.920260777 | 8.602890051 | -12.05856508 | 1.48E-28 | 1.23E-27 | 53.77284733 |
| SIDT2 | -0.660995426 | 9.722233495 | -12.05845199 | 1.48E-28 | 1.23E-27 | 53.77186189 |
| PRKCZ | -0.658742249 | 7.67437137 | -12.05770788 | 1.49E-28 | 1.24E-27 | 53.765378 |
| ATF3 | -0.799486216 | 6.619054356 | -12.04679641 | 1.64E-28 | 1.36E-27 | 53.67031999 |
| DERL3 | 1.050037936 | 6.447186266 | 12.04311514 | 1.69E-28 | 1.40E-27 | 53.63825836 |
| C1orf74 | 0.77549582 | 6.225713769 | 12.02864357 | 1.92E-28 | 1.59E-27 | 53.51226187 |
| PDE1C | -0.723084789 | 4.738747676 | -12.02805318 | 1.93E-28 | 1.59E-27 | 53.50712305 |
| IL1B | -1.512370839 | 7.406521822 | -12.0265944 | 1.95E-28 | 1.61E-27 | 53.49442624 |
| CPAMD8 | -1.123725963 | 6.688307684 | -12.02155707 | 2.04E-28 | 1.69E-27 | 53.45058796 |
| C20orf85 | -2.263371209 | 6.711082686 | -12.01699606 | 2.12E-28 | 1.75E-27 | 53.410902 |
| PLOD2 | 1.168611353 | 8.139347851 | 12.01107903 | 2.24E-28 | 1.84E-27 | 53.35942721 |
| ARHGEF3 | -0.652660649 | 9.389373277 | -12.00485703 | 2.36E-28 | 1.94E-27 | 53.30531154 |
| CCND3 | -0.593730889 | 10.32283649 | -12.00178485 | 2.43E-28 | 1.99E-27 | 53.27859593 |
| FAM105A | -1.180838245 | 7.165955811 | -12.00165421 | 2.43E-28 | 2.00E-27 | 53.27745991 |
| GNL3 | 0.585387131 | 8.074879904 | 11.99967187 | 2.47E-28 | 2.03E-27 | 53.26022324 |
| SNF8 | 0.608927069 | 8.995578606 | 11.99304508 | 2.62E-28 | 2.15E-27 | 53.20261187 |
| EMP2 | -0.983803988 | 7.921552673 | -11.96841364 | 3.25E-28 | 2.66E-27 | 52.98859829 |
| VGF | 1.086641414 | 5.80459438 | 11.96399068 | 3.38E-28 | 2.76E-27 | 52.95018972 |
| AGBL1 | -0.978127699 | 4.916909532 | -11.95809092 | 3.56E-28 | 2.91E-27 | 52.89896675 |
| ARHGEF10 | -0.835360646 | 8.089343011 | -11.94296963 | 4.06E-28 | 3.31E-27 | 52.76773246 |
| GPD1L | -0.917045162 | 9.669330069 | -11.94004368 | 4.16E-28 | 3.39E-27 | 52.74234745 |
| CEP72 | 0.699361516 | 5.784934649 | 11.93860881 | 4.22E-28 | 3.43E-27 | 52.72989973 |
| PTPLA | -1.358043138 | 7.383071609 | -11.93813493 | 4.23E-28 | 3.45E-27 | 52.72578895 |
| RAC3 | 0.962728328 | 6.393518117 | 11.9332666 | 4.42E-28 | 3.59E-27 | 52.68356141 |
| S100A12 | -1.452932914 | 5.443994891 | -11.93243734 | 4.45E-28 | 3.62E-27 | 52.6763693 |
| CMTM2 | -1.220135876 | 5.399744574 | -11.92167658 | 4.89E-28 | 3.97E-27 | 52.58306188 |
| ARHGAP6 | -0.675263733 | 5.228437981 | -11.91589696 | 5.14E-28 | 4.16E-27 | 52.53296211 |
| GIPC2 | -0.7522977 | 4.890528731 | -11.90584933 | 5.61E-28 | 4.54E-27 | 52.44589182 |
| PAQR4 | 0.745070448 | 8.549007021 | 11.88640954 | 6.65E-28 | 5.37E-27 | 52.27752617 |
| TMEM92 | 0.944049894 | 5.845422149 | 11.88496124 | 6.73E-28 | 5.43E-27 | 52.26498765 |
| HIST1H2BH | 0.954895981 | 6.286473029 | 11.88479239 | 6.74E-28 | 5.43E-27 | 52.26352586 |
| SLC29A2 | 0.795799652 | 7.192781141 | 11.88002447 | 7.03E-28 | 5.66E-27 | 52.22225316 |
| ZAK | -0.714729737 | 7.095571936 | -11.8784113 | 7.13E-28 | 5.74E-27 | 52.20829076 |
| ATXN2L | 0.585663423 | 6.15228921 | 11.87462448 | 7.37E-28 | 5.92E-27 | 52.17551813 |
| NEK6 | 0.738178004 | 8.240896949 | 11.87325967 | 7.45E-28 | 5.99E-27 | 52.16370772 |
| TBC1D2 | -0.850080784 | 8.648015226 | -11.86348307 | 8.12E-28 | 6.51E-27 | 52.07912377 |
| DEPDC1 | 1.022097046 | 4.734756144 | 11.85895162 | 8.44E-28 | 6.77E-27 | 52.03992995 |
| NOS1AP | 0.882798814 | 5.720296941 | 11.85591747 | 8.67E-28 | 6.94E-27 | 52.01369053 |
| APOA1BP | 0.607801271 | 10.33310769 | 11.84846342 | 9.25E-28 | 7.39E-27 | 51.9492407 |
| CA9 | 1.733243954 | 6.404439077 | 11.84344511 | 9.66E-28 | 7.72E-27 | 51.90586144 |
| ACP5 | -1.224005083 | 8.988120854 | -11.83906572 | 1.00E-27 | 8.01E-27 | 51.86801203 |
| GPR65 | -1.011174183 | 6.652354423 | -11.83595314 | 1.03E-27 | 8.23E-27 | 51.841115 |
| CASKIN2 | -0.719568127 | 6.284439832 | -11.83466216 | 1.04E-27 | 8.31E-27 | 51.82996013 |
| KISS1R | 1.695674552 | 6.037067003 | 11.83455201 | 1.04E-27 | 8.31E-27 | 51.82900838 |
| VAPA | -0.65376981 | 7.247101617 | -11.83137381 | 1.07E-27 | 8.54E-27 | 51.80154916 |
| PNRC2 | -0.609071579 | 8.844098481 | -11.83024081 | 1.08E-27 | 8.62E-27 | 51.79176105 |
| CTSG | -1.536708849 | 6.974436375 | -11.828047 | 1.10E-27 | 8.78E-27 | 51.77280964 |
| SORT1 | -0.719538682 | 10.00433257 | -11.81765538 | 1.21E-27 | 9.59E-27 | 51.68306273 |
| ENTPD7 | 0.633915976 | 6.15772463 | 11.81739524 | 1.21E-27 | 9.61E-27 | 51.68081655 |
| C4orf32 | -0.751232362 | 6.599700016 | -11.81734021 | 1.21E-27 | 9.61E-27 | 51.68034134 |
| ZNF692 | 0.776788491 | 7.7803875 | 11.81347652 | 1.25E-27 | 9.92E-27 | 51.64698235 |
| ITPR1 | -0.928758826 | 7.638670359 | -11.8131822 | 1.26E-27 | 9.94E-27 | 51.64444144 |
| ABLIM1 | -0.775349779 | 8.157450396 | -11.80258685 | 1.38E-27 | 1.09E-26 | 51.55298834 |
| PNRC1 | -0.665921128 | 6.956017324 | -11.79357398 | 1.49E-27 | 1.18E-26 | 51.47522404 |
| SLC25A13 | 0.760934359 | 8.037936684 | 11.78940424 | 1.55E-27 | 1.22E-26 | 51.43925621 |
| AXIN2 | -0.888719777 | 7.921895043 | -11.7878837 | 1.57E-27 | 1.23E-26 | 51.42614159 |
| PRR7 | 0.946778753 | 6.151564295 | 11.78515263 | 1.60E-27 | 1.26E-26 | 51.40258823 |
| NUF2 | 1.124432521 | 5.064370316 | 11.78464708 | 1.61E-27 | 1.27E-26 | 51.39822852 |
| CYP24A1 | 2.309752673 | 5.735097598 | 11.78279238 | 1.64E-27 | 1.29E-26 | 51.38223485 |
| SCNN1G | -1.364123581 | 6.70695563 | -11.77893868 | 1.69E-27 | 1.33E-26 | 51.34900704 |
| HHEX | -0.936486738 | 7.282649832 | -11.77393256 | 1.77E-27 | 1.39E-26 | 51.30585012 |
| FKBP14 | 0.914268817 | 8.320384606 | 11.7703946 | 1.82E-27 | 1.43E-26 | 51.27535512 |
| CEACAM1 | 0.931677834 | 7.686020737 | 11.76858782 | 1.85E-27 | 1.45E-26 | 51.2597834 |
| PPP2R5A | -0.610194514 | 9.815042612 | -11.75892661 | 2.01E-27 | 1.57E-26 | 51.17653725 |
| ESD | -0.600138547 | 8.690849811 | -11.75479664 | 2.09E-27 | 1.63E-26 | 51.14096094 |
| UHMK1 | 1.070393213 | 7.608959848 | 11.7514299 | 2.15E-27 | 1.68E-26 | 51.1119635 |
| BAALC | -0.808827745 | 5.80370825 | -11.74719642 | 2.23E-27 | 1.74E-26 | 51.07550633 |
| PDE3B | -1.061899419 | 6.231313957 | -11.74713369 | 2.23E-27 | 1.74E-26 | 51.07496614 |
| TPST2 | -0.620850089 | 8.55994487 | -11.74437045 | 2.28E-27 | 1.78E-26 | 51.05117351 |
| SORD | 0.752219678 | 6.764146612 | 11.74258868 | 2.32E-27 | 1.80E-26 | 51.03583312 |
| INPP1 | -0.770048331 | 8.635684245 | -11.73604806 | 2.46E-27 | 1.91E-26 | 50.97953006 |
| TRAF4 | 0.61890754 | 6.147636593 | 11.72651733 | 2.67E-27 | 2.07E-26 | 50.89751357 |
| STAP2 | 0.668380556 | 6.930634101 | 11.72335047 | 2.74E-27 | 2.12E-26 | 50.87026802 |
| NRIP3 | 1.277474159 | 6.69921459 | 11.72109004 | 2.80E-27 | 2.16E-26 | 50.85082299 |
| NQO1 | 1.377391125 | 8.370593926 | 11.72085471 | 2.80E-27 | 2.17E-26 | 50.84879868 |
| ASNS | 0.787175043 | 7.859190484 | 11.71942716 | 2.84E-27 | 2.19E-26 | 50.83651933 |
| CHRNA5 | 1.247038794 | 6.799742859 | 11.7155562 | 2.93E-27 | 2.26E-26 | 50.80322614 |
| SLC22A18 | 0.679340645 | 7.348232644 | 11.69944085 | 3.37E-27 | 2.60E-26 | 50.66467696 |
| SPI1 | -0.892658756 | 7.955529726 | -11.691186 | 3.62E-27 | 2.79E-26 | 50.59374169 |
| LRRC48 | -0.86418514 | 5.451183715 | -11.68526875 | 3.81E-27 | 2.93E-26 | 50.5429083 |
| RNF122 | -0.863943916 | 6.931658418 | -11.68426466 | 3.84E-27 | 2.95E-26 | 50.5342836 |
| ATP13A2 | 0.682716176 | 7.644595388 | 11.68247134 | 3.90E-27 | 2.99E-26 | 50.51888068 |
| FOXF2 | -1.085362302 | 7.626597295 | -11.67990323 | 3.99E-27 | 3.06E-26 | 50.49682504 |
| OIP5 | 1.134211749 | 5.547093862 | 11.67898197 | 4.02E-27 | 3.08E-26 | 50.4889135 |
| NOC2L | 0.613600687 | 7.302626809 | 11.67387675 | 4.21E-27 | 3.22E-26 | 50.44507672 |
| CLEC10A | -0.998209869 | 5.975936444 | -11.67008345 | 4.35E-27 | 3.32E-26 | 50.41251081 |
| NSUN5 | 0.618348665 | 7.932485947 | 11.66889058 | 4.39E-27 | 3.36E-26 | 50.40227086 |
| NFIA | -0.982799376 | 8.24681984 | -11.66737776 | 4.45E-27 | 3.40E-26 | 50.38928514 |
| MAN1C1 | -0.871320313 | 7.511564856 | -11.66349817 | 4.60E-27 | 3.51E-26 | 50.35598718 |
| EXOSC4 | 0.660724977 | 8.119560066 | 11.65493276 | 4.95E-27 | 3.77E-26 | 50.28248984 |
| CORO1C | -0.681222803 | 9.979513029 | -11.65274012 | 5.05E-27 | 3.84E-26 | 50.26367954 |
| ZNF239 | 0.905404995 | 6.224026923 | 11.64967547 | 5.18E-27 | 3.94E-26 | 50.23739116 |
| AFF2 | -0.702480999 | 5.460332678 | -11.64401866 | 5.44E-27 | 4.14E-26 | 50.18887593 |
| TCEAL1 | -0.604125999 | 6.951258476 | -11.63335624 | 5.97E-27 | 4.53E-26 | 50.09746069 |
| TBC1D4 | -0.695474762 | 7.664695676 | -11.62877488 | 6.21E-27 | 4.71E-26 | 50.05819411 |
| ALDH1A1 | -1.652905342 | 9.124426354 | -11.61235848 | 7.15E-27 | 5.42E-26 | 49.91755008 |
| VWA1 | 0.615546597 | 7.380830968 | 11.60900345 | 7.36E-27 | 5.56E-26 | 49.88881819 |
| SCEL | -1.511997722 | 6.869146636 | -11.6065375 | 7.52E-27 | 5.68E-26 | 49.86770273 |
| HKDC1 | 1.263593711 | 6.428264636 | 11.60131731 | 7.87E-27 | 5.94E-26 | 49.82301012 |
| GALNT6 | 1.219056704 | 7.078101388 | 11.59929704 | 8.01E-27 | 6.04E-26 | 49.80571618 |
| KCNE1 | -0.899958102 | 5.248484104 | -11.58997916 | 8.68E-27 | 6.53E-26 | 49.72597168 |
| BMP5 | -1.318879792 | 6.551522617 | -11.58894052 | 8.75E-27 | 6.59E-26 | 49.71708458 |
| RAD51AP1 | 1.306983012 | 6.159121705 | 11.58858534 | 8.78E-27 | 6.60E-26 | 49.7140456 |
| SEC61A2 | 0.756467938 | 5.599573306 | 11.58255959 | 9.25E-27 | 6.94E-26 | 49.66249508 |
| ALDH3B2 | 1.15016261 | 5.67561892 | 11.58042656 | 9.42E-27 | 7.07E-26 | 49.64424994 |
| C12orf49 | -0.748601814 | 8.208216867 | -11.57238273 | 1.01E-26 | 7.57E-26 | 49.57546047 |
| WDR16 | -1.077235854 | 5.087196529 | -11.57168784 | 1.02E-26 | 7.60E-26 | 49.56951892 |
| PAPSS1 | -0.68785296 | 9.153314979 | -11.56740029 | 1.05E-26 | 7.88E-26 | 49.53286287 |
| PLAGL1 | -0.791177456 | 6.550527189 | -11.56504057 | 1.08E-26 | 8.04E-26 | 49.51269142 |
| LRRC8E | 0.588635344 | 6.129723886 | 11.55415424 | 1.18E-26 | 8.81E-26 | 49.41965778 |
| MUC20 | 1.413085883 | 7.734062274 | 11.54911022 | 1.23E-26 | 9.19E-26 | 49.37656616 |
| KCNK17 | -1.10344726 | 6.629529154 | -11.53881609 | 1.35E-26 | 1.00E-25 | 49.2886502 |
| APH1B | -0.586699864 | 7.643422572 | -11.5253537 | 1.51E-26 | 1.12E-25 | 49.17373264 |
| CBX5 | 0.6305518 | 8.096152322 | 11.52018102 | 1.58E-26 | 1.17E-25 | 49.12959483 |
| SERTAD1 | -0.7824711 | 9.131818226 | -11.5117973 | 1.70E-26 | 1.26E-25 | 49.0580776 |
| NLRP3 | -1.039152643 | 6.395503351 | -11.51004021 | 1.73E-26 | 1.28E-25 | 49.04309202 |
| CASP1 | -0.818024518 | 7.760757734 | -11.50872242 | 1.75E-26 | 1.29E-25 | 49.03185366 |
| FKBP11 | 0.797277795 | 9.281864114 | 11.50574833 | 1.79E-26 | 1.32E-25 | 49.00649255 |
| STRA6 | 1.384996312 | 5.757349348 | 11.50432197 | 1.81E-26 | 1.34E-25 | 48.99433058 |
| APCDD1 | -0.956542528 | 7.839983463 | -11.50321026 | 1.83E-26 | 1.35E-25 | 48.98485195 |
| ACAT1 | -0.72681565 | 9.626352101 | -11.50195472 | 1.85E-26 | 1.36E-25 | 48.97414765 |
| TUBA1A | -0.642102289 | 11.13003718 | -11.49288698 | 2.00E-26 | 1.47E-25 | 48.89685539 |
| SEC24D | 0.808035261 | 8.069005082 | 11.4883019 | 2.08E-26 | 1.53E-25 | 48.8577839 |
| ARFGAP1 | 0.767187035 | 8.072029048 | 11.48770309 | 2.09E-26 | 1.54E-25 | 48.85268173 |
| KIF23 | 1.001175433 | 5.311537694 | 11.48068341 | 2.22E-26 | 1.63E-25 | 48.7928801 |
| WDR74 | 0.606378388 | 8.392116649 | 11.47955174 | 2.24E-26 | 1.65E-25 | 48.7832409 |
| PTGIS | -1.294242478 | 8.568631197 | -11.47901927 | 2.25E-26 | 1.65E-25 | 48.77870565 |
| PCNXL3 | 0.669031262 | 8.095772013 | 11.47786745 | 2.27E-26 | 1.67E-25 | 48.76889543 |
| SPC25 | 0.912512289 | 4.662213606 | 11.47645262 | 2.30E-26 | 1.69E-25 | 48.75684587 |
| CST2 | 1.130811449 | 5.844313801 | 11.47251845 | 2.38E-26 | 1.74E-25 | 48.72334381 |
| ZNF281 | 0.779353249 | 8.648157005 | 11.47207162 | 2.39E-26 | 1.75E-25 | 48.71953906 |
| MCL1 | -0.682861823 | 9.771948431 | -11.46815731 | 2.47E-26 | 1.81E-25 | 48.68621219 |
| PACRG | -0.720462948 | 5.020594516 | -11.46741501 | 2.49E-26 | 1.82E-25 | 48.67989278 |
| PRDM6 | -0.719900221 | 6.377848351 | -11.45837908 | 2.69E-26 | 1.96E-25 | 48.60298336 |
| SLPI | -1.748188487 | 11.46262942 | -11.45098686 | 2.86E-26 | 2.09E-25 | 48.54008612 |
| APOLD1 | -1.332473156 | 7.790920229 | -11.44884153 | 2.92E-26 | 2.13E-25 | 48.52183615 |
| HNF4G | 1.095600025 | 4.812654444 | 11.44608167 | 2.99E-26 | 2.17E-25 | 48.49836081 |
| CLDN12 | 0.774475009 | 8.797267388 | 11.43910575 | 3.17E-26 | 2.31E-25 | 48.43903607 |
| MXRA7 | -0.677571058 | 7.091629721 | -11.43539783 | 3.27E-26 | 2.38E-25 | 48.40751025 |
| FAM122A | -0.669550721 | 5.944646851 | -11.42781878 | 3.49E-26 | 2.54E-25 | 48.34308637 |
| ARHGAP15 | -0.920859556 | 8.077970585 | -11.42682131 | 3.52E-26 | 2.56E-25 | 48.3346092 |
| ERG | -0.661863694 | 5.982600955 | -11.42292393 | 3.64E-26 | 2.64E-25 | 48.3014899 |
| MAP3K9 | 0.848649938 | 6.424865894 | 11.42204338 | 3.67E-26 | 2.66E-25 | 48.29400797 |
| CDC42EP2 | -0.861154803 | 7.254739761 | -11.39757947 | 4.52E-26 | 3.26E-25 | 48.08625131 |
| CFL2 | -0.644001626 | 6.356108995 | -11.39419092 | 4.66E-26 | 3.36E-25 | 48.05749158 |
| PRKD1 | -1.092952392 | 7.40870466 | -11.39134187 | 4.77E-26 | 3.44E-25 | 48.03331401 |
| TNF | -0.953288614 | 5.820918963 | -11.38666821 | 4.97E-26 | 3.57E-25 | 47.99365883 |
| CLCN4 | -0.645493772 | 4.944699896 | -11.38560397 | 5.01E-26 | 3.60E-25 | 47.98463005 |
| C8orf34 | -0.856434241 | 6.080137045 | -11.38501499 | 5.04E-26 | 3.62E-25 | 47.97963347 |
| AHCY | 0.604097069 | 9.948541226 | 11.38429983 | 5.07E-26 | 3.64E-25 | 47.97356665 |
| ABCA12 | 1.052125207 | 4.951106176 | 11.38211134 | 5.16E-26 | 3.70E-25 | 47.95500229 |
| NUDT1 | 0.667539013 | 7.370084157 | 11.37653838 | 5.41E-26 | 3.88E-25 | 47.90773641 |
| MRPS17 | 0.675565462 | 8.791998508 | 11.37288079 | 5.59E-26 | 4.00E-25 | 47.87672147 |
| MEOX2 | -1.089260472 | 5.778007947 | -11.37200715 | 5.63E-26 | 4.03E-25 | 47.86931407 |
| HIST1H2BE | 0.747476121 | 5.782417255 | 11.37017464 | 5.72E-26 | 4.09E-25 | 47.85377752 |
| RASSF6 | 0.91422363 | 5.821190125 | 11.36985974 | 5.73E-26 | 4.10E-25 | 47.85110781 |
| PUS7 | 0.788776628 | 7.873342678 | 11.3651923 | 5.96E-26 | 4.26E-25 | 47.81154193 |
| SYNPO2 | -0.825248657 | 5.832610649 | -11.36378009 | 6.04E-26 | 4.31E-25 | 47.79957224 |
| UST | -1.187401891 | 6.843033234 | -11.36272408 | 6.09E-26 | 4.34E-25 | 47.79062212 |
| LY86 | -1.15329404 | 8.547429452 | -11.36169195 | 6.15E-26 | 4.38E-25 | 47.78187475 |
| GPBAR1 | -0.857393495 | 6.377031162 | -11.36123717 | 6.17E-26 | 4.39E-25 | 47.77802054 |
| SYT7 | 0.607113468 | 5.600952519 | 11.35644573 | 6.43E-26 | 4.57E-25 | 47.73741883 |
| IGFBP6 | -1.257024272 | 8.582649761 | -11.35630686 | 6.43E-26 | 4.58E-25 | 47.73624217 |
| SLC18A2 | -0.732065938 | 5.231639699 | -11.35204557 | 6.67E-26 | 4.74E-25 | 47.70014 |
| MAP7D2 | 1.469246413 | 5.058520713 | 11.34669459 | 6.98E-26 | 4.96E-25 | 47.65481535 |
| MT1E | -1.183117836 | 9.28621558 | -11.34531552 | 7.07E-26 | 5.01E-25 | 47.64313588 |
| TRIP13 | 1.535002557 | 7.059986242 | 11.34526605 | 7.07E-26 | 5.01E-25 | 47.64271689 |
| USP44 | -0.76123397 | 5.114316918 | -11.33438638 | 7.76E-26 | 5.48E-25 | 47.55060039 |
| LAMA3 | -1.215549443 | 7.24829916 | -11.32520006 | 8.39E-26 | 5.91E-25 | 47.47285502 |
| VEGFC | -1.055392102 | 7.300341838 | -11.32506989 | 8.40E-26 | 5.91E-25 | 47.47175363 |
| UBE2S | 0.696763186 | 7.232092747 | 11.317984 | 8.92E-26 | 6.28E-25 | 47.41180614 |
| CAMP | -1.420540316 | 6.689602785 | -11.31470523 | 9.17E-26 | 6.45E-25 | 47.38407363 |
| FBXO16 | 0.80998548 | 5.358888426 | 11.29992209 | 1.04E-25 | 7.30E-25 | 47.25908393 |
| VDR | 0.626675514 | 6.506588319 | 11.29813421 | 1.06E-25 | 7.41E-25 | 47.2439731 |
| GARNL3 | -0.706213103 | 6.071695955 | -11.2975799 | 1.06E-25 | 7.44E-25 | 47.23928837 |
| DSG2 | 1.155243528 | 8.17144317 | 11.29747768 | 1.06E-25 | 7.44E-25 | 47.2384245 |
| RBMS2 | -0.605648577 | 7.803376928 | -11.29651775 | 1.07E-25 | 7.49E-25 | 47.23031211 |
| EGLN3 | 1.421899007 | 6.71921608 | 11.29533059 | 1.08E-25 | 7.57E-25 | 47.22027977 |
| RCBTB2 | -0.646839041 | 6.951898984 | -11.29377306 | 1.10E-25 | 7.66E-25 | 47.20711835 |
| AXL | -0.709091281 | 8.958223239 | -11.29003854 | 1.13E-25 | 7.90E-25 | 47.17556471 |
| MICA | -0.758498029 | 7.077479649 | -11.28943426 | 1.14E-25 | 7.94E-25 | 47.17045957 |
| PRRG1 | -0.969411015 | 6.877140673 | -11.27715545 | 1.26E-25 | 8.80E-25 | 47.0667528 |
| CTSW | -0.942910281 | 7.113927146 | -11.27633435 | 1.27E-25 | 8.86E-25 | 47.05981981 |
| MXD3 | 0.652350529 | 6.970027255 | 11.27292116 | 1.31E-25 | 9.11E-25 | 47.03100296 |
| COL7A1 | 1.254190021 | 6.898846056 | 11.26519812 | 1.40E-25 | 9.73E-25 | 46.96581504 |
| BAIAP2L2 | 1.304871195 | 6.363968255 | 11.26464749 | 1.40E-25 | 9.76E-25 | 46.96116814 |
| ITGB4 | 0.895316442 | 7.124052777 | 11.25758965 | 1.49E-25 | 1.04E-24 | 46.90161583 |
| IFIT3 | -1.070950599 | 7.455475843 | -11.25589373 | 1.51E-25 | 1.05E-24 | 46.88730886 |
| SNAP23 | -0.663540326 | 7.874787838 | -11.25554136 | 1.52E-25 | 1.05E-24 | 46.8843364 |
| CREB3L4 | 0.950308723 | 6.540082662 | 11.25540407 | 1.52E-25 | 1.05E-24 | 46.88317821 |
| HYLS1 | 0.682933736 | 6.329226301 | 11.24310435 | 1.69E-25 | 1.17E-24 | 46.77945063 |
| MATN3 | -1.376552786 | 5.777025061 | -11.23809208 | 1.76E-25 | 1.22E-24 | 46.73719663 |
| UCHL5 | 0.745920691 | 7.641101694 | 11.23715037 | 1.77E-25 | 1.23E-24 | 46.72925901 |
| E2F7 | 1.178816909 | 5.130566726 | 11.23699699 | 1.78E-25 | 1.23E-24 | 46.72796617 |
| DTL | 1.233302661 | 6.036354412 | 11.23671565 | 1.78E-25 | 1.23E-24 | 46.72559487 |
| THY1 | 1.140183978 | 8.957985904 | 11.22851615 | 1.91E-25 | 1.32E-24 | 46.6564967 |
| TRIM2 | 1.053168621 | 8.644259051 | 11.22678283 | 1.94E-25 | 1.33E-24 | 46.64189304 |
| WWC3 | -0.600171894 | 8.896169718 | -11.22569438 | 1.96E-25 | 1.35E-24 | 46.63272314 |
| ZMYND15 | -0.705271459 | 7.025705021 | -11.22239449 | 2.01E-25 | 1.38E-24 | 46.60492519 |
| PPIF | 0.650387924 | 6.571473388 | 11.22231255 | 2.01E-25 | 1.38E-24 | 46.60423494 |
| TFAP2C | 0.972011524 | 7.386893178 | 11.22092059 | 2.04E-25 | 1.40E-24 | 46.59251045 |
| OPLAH | 0.773982175 | 7.087570851 | 11.21692022 | 2.11E-25 | 1.45E-24 | 46.55881943 |
| TTC7B | -0.631010718 | 6.321843391 | -11.21458314 | 2.15E-25 | 1.47E-24 | 46.53913934 |
| CD68 | -0.820370925 | 9.645453088 | -11.21146895 | 2.21E-25 | 1.51E-24 | 46.51291856 |
| CNGB1 | 0.941269234 | 5.866060622 | 11.20931415 | 2.25E-25 | 1.54E-24 | 46.49477774 |
| PHLDB2 | -1.048875065 | 6.589608383 | -11.20876507 | 2.26E-25 | 1.55E-24 | 46.49015546 |
| LILRB2 | -0.643403049 | 6.314036149 | -11.20309814 | 2.37E-25 | 1.62E-24 | 46.44245631 |
| MYRIP | -0.592447016 | 4.748205803 | -11.19661007 | 2.50E-25 | 1.71E-24 | 46.38786036 |
| CDC6 | 0.894447048 | 4.863997088 | 11.18662235 | 2.72E-25 | 1.86E-24 | 46.30384644 |
| TNFRSF1B | -0.750399145 | 8.299048715 | -11.18636389 | 2.73E-25 | 1.86E-24 | 46.30167286 |
| CABYR | 1.153026995 | 5.360960745 | 11.18304934 | 2.81E-25 | 1.91E-24 | 46.27380041 |
| GPI | 0.607499756 | 10.44554539 | 11.18131353 | 2.85E-25 | 1.94E-24 | 46.25920545 |
| FLRT3 | -1.310338699 | 7.708691082 | -11.17785784 | 2.93E-25 | 2.00E-24 | 46.23015289 |
| SLFN13 | 1.169049973 | 6.57659566 | 11.17424742 | 3.02E-25 | 2.06E-24 | 46.19980419 |
| PANX2 | 0.970943078 | 7.324822178 | 11.17302344 | 3.06E-25 | 2.07E-24 | 46.18951675 |
| NAV3 | -0.762515504 | 5.899244625 | -11.16965129 | 3.14E-25 | 2.13E-24 | 46.16117702 |
| JAK1 | -0.781864106 | 9.152282543 | -11.16281302 | 3.33E-25 | 2.25E-24 | 46.10372095 |
| IGFBP3 | 1.190071594 | 9.820550569 | 11.1597652 | 3.42E-25 | 2.31E-24 | 46.0781185 |
| TEKT1 | -1.660770613 | 6.199024785 | -11.155746 | 3.54E-25 | 2.39E-24 | 46.04436157 |
| FILIP1 | -1.219545426 | 7.03376383 | -11.15559821 | 3.54E-25 | 2.39E-24 | 46.04312041 |
| NR5A2 | -0.840349292 | 5.738422875 | -11.15309246 | 3.62E-25 | 2.44E-24 | 46.02207808 |
| TLE4 | -0.687623286 | 7.059545441 | -11.15277751 | 3.63E-25 | 2.45E-24 | 46.01943343 |
| TFAP4 | 0.616026351 | 6.091870255 | 11.14808675 | 3.77E-25 | 2.54E-24 | 45.98004915 |
| MB | 0.909785433 | 5.497900303 | 11.14713792 | 3.80E-25 | 2.56E-24 | 45.97208365 |
| RAB11FIP2 | -0.667370773 | 8.045388114 | -11.14552346 | 3.86E-25 | 2.59E-24 | 45.95853091 |
| RBP2 | -1.113633595 | 5.432150949 | -11.14472938 | 3.88E-25 | 2.61E-24 | 45.95186536 |
| ACY3 | 0.845522194 | 5.347675077 | 11.14446557 | 3.89E-25 | 2.61E-24 | 45.94965092 |
| GSTM3 | -1.218519013 | 7.8181045 | -11.14415182 | 3.90E-25 | 2.62E-24 | 45.94701738 |
| RACGAP1 | 0.765259185 | 6.883943702 | 11.14397275 | 3.91E-25 | 2.62E-24 | 45.94551427 |
| OLFML2A | -0.877921123 | 8.220869351 | -11.14088803 | 4.01E-25 | 2.69E-24 | 45.9196239 |
| CCNE2 | 1.015272888 | 5.302468489 | 11.14086519 | 4.01E-25 | 2.69E-24 | 45.91943222 |
| LAT2 | -0.6983756 | 7.102970721 | -11.13830425 | 4.10E-25 | 2.74E-24 | 45.89794077 |
| DYNLRB2 | -1.301100286 | 6.344573577 | -11.13816942 | 4.10E-25 | 2.75E-24 | 45.8968094 |
| LPHN3 | -0.888791446 | 5.160667702 | -11.13740326 | 4.13E-25 | 2.76E-24 | 45.89038028 |
| WNT2 | -0.992788027 | 6.923436785 | -11.13214925 | 4.32E-25 | 2.88E-24 | 45.84629818 |
| MTM1 | -0.675695404 | 6.984183386 | -11.13124302 | 4.35E-25 | 2.91E-24 | 45.83869574 |
| GNG7 | -0.765112443 | 7.25560975 | -11.11762453 | 4.88E-25 | 3.25E-24 | 45.72448781 |
| EFHB | -1.029795636 | 4.666498896 | -11.11015341 | 5.20E-25 | 3.46E-24 | 45.66186327 |
| ARHGAP28 | -0.809530971 | 5.53951617 | -11.10442582 | 5.46E-25 | 3.63E-24 | 45.61386778 |
| TMEM105 | 0.727131768 | 5.987976274 | 11.10428256 | 5.47E-25 | 3.63E-24 | 45.61266744 |
| SRPX2 | 1.35917208 | 7.951606428 | 11.10394001 | 5.48E-25 | 3.64E-24 | 45.60979741 |
| DOCK2 | -0.922559691 | 8.883851707 | -11.10213103 | 5.57E-25 | 3.69E-24 | 45.59464165 |
| C1QB | -1.135646761 | 11.3232179 | -11.10107949 | 5.61E-25 | 3.72E-24 | 45.5858324 |
| DNTTIP1 | 0.737892028 | 9.179397205 | 11.09923923 | 5.70E-25 | 3.78E-24 | 45.57041662 |
| MMACHC | 0.593853748 | 6.336477027 | 11.09900501 | 5.71E-25 | 3.78E-24 | 45.56845458 |
| TBC1D16 | 0.594294276 | 7.673239399 | 11.09541064 | 5.89E-25 | 3.90E-24 | 45.53834875 |
| SERPINB9 | -0.65104901 | 6.386794894 | -11.09018055 | 6.16E-25 | 4.07E-24 | 45.49455129 |
| LRFN4 | 0.891207311 | 6.794526803 | 11.08939132 | 6.20E-25 | 4.10E-24 | 45.48794306 |
| FAM98A | 0.606203853 | 9.102080197 | 11.08605195 | 6.37E-25 | 4.21E-24 | 45.45998517 |
| ZNF579 | 0.672198523 | 7.260228045 | 11.08565003 | 6.40E-25 | 4.22E-24 | 45.45662052 |
| FRAT1 | -0.70960655 | 6.270652045 | -11.08129313 | 6.63E-25 | 4.37E-24 | 45.4201508 |
| SLC41A2 | 1.01729935 | 6.623811069 | 11.07470014 | 7.01E-25 | 4.62E-24 | 45.36497763 |
| MESP1 | 0.980852043 | 7.150055133 | 11.07390104 | 7.06E-25 | 4.65E-24 | 45.35829159 |
| TBX15 | 1.184879654 | 5.386569854 | 11.06778808 | 7.43E-25 | 4.89E-24 | 45.30715237 |
| PSMD14 | 0.588591392 | 9.292212984 | 11.06098404 | 7.87E-25 | 5.17E-24 | 45.2502487 |
| CHAF1A | 0.630291907 | 5.952680141 | 11.05904597 | 8.00E-25 | 5.24E-24 | 45.23404347 |
| RAB32 | -0.672441527 | 8.774338907 | -11.04672986 | 8.88E-25 | 5.80E-24 | 45.1310958 |
| NOXA1 | 0.65458689 | 7.59959442 | 11.04575251 | 8.95E-25 | 5.85E-24 | 45.12292881 |
| C9orf24 | -1.465108876 | 6.506291386 | -11.04104828 | 9.31E-25 | 6.08E-24 | 45.08362445 |
| NEK7 | -0.676791537 | 7.421219019 | -11.03869082 | 9.50E-25 | 6.20E-24 | 45.06393083 |
| SPG20 | -0.607638583 | 7.036743029 | -11.0263064 | 1.05E-24 | 6.86E-24 | 44.96050996 |
| SPATA18 | -1.456245659 | 6.802535415 | -11.02546933 | 1.06E-24 | 6.91E-24 | 44.95352178 |
| GRN | -0.592012678 | 11.36312855 | -11.0244282 | 1.07E-24 | 6.96E-24 | 44.94483046 |
| MAPK8IP3 | 0.668485415 | 6.885499816 | 11.01675203 | 1.14E-24 | 7.42E-24 | 44.88076309 |
| DAPK1 | -0.893946367 | 9.286415242 | -11.01340583 | 1.18E-24 | 7.63E-24 | 44.85284196 |
| HDHD3 | 0.673778268 | 7.595094178 | 11.00920335 | 1.22E-24 | 7.90E-24 | 44.81778212 |
| CLU | -0.838948953 | 6.514706859 | -11.00842288 | 1.23E-24 | 7.94E-24 | 44.81127165 |
| ETV1 | -0.750509072 | 5.923538311 | -11.00791782 | 1.23E-24 | 7.97E-24 | 44.80705873 |
| HDAC10 | 0.704723235 | 5.902587614 | 11.00690997 | 1.24E-24 | 8.04E-24 | 44.79865212 |
| GPR56 | 0.691661332 | 8.540830043 | 11.00561707 | 1.25E-24 | 8.12E-24 | 44.78786846 |
| KLB | -0.901406445 | 5.137220231 | -11.00268172 | 1.29E-24 | 8.32E-24 | 44.76338796 |
| PSME4 | 0.632130878 | 9.283186234 | 11.00155421 | 1.30E-24 | 8.39E-24 | 44.75398557 |
| HES6 | 1.044279577 | 7.638745939 | 11.00088859 | 1.31E-24 | 8.43E-24 | 44.74843516 |
| CRY1 | -0.671883594 | 7.947927652 | -10.99405494 | 1.38E-24 | 8.91E-24 | 44.69146117 |
| PDE4B | -0.622916973 | 6.447894715 | -10.99074624 | 1.42E-24 | 9.15E-24 | 44.66388226 |
| PRICKLE1 | -0.854098838 | 8.199048535 | -10.98845788 | 1.45E-24 | 9.33E-24 | 44.64481059 |
| OSBP2 | 0.753759835 | 6.090570205 | 10.98328848 | 1.51E-24 | 9.72E-24 | 44.60173528 |
| ARHGAP18 | -0.970724539 | 7.525656149 | -10.97589029 | 1.61E-24 | 1.03E-23 | 44.54010624 |
| MPP6 | 1.014945599 | 6.084730918 | 10.96864622 | 1.71E-24 | 1.10E-23 | 44.47978187 |
| COL17A1 | 1.476824007 | 6.23799913 | 10.96207816 | 1.81E-24 | 1.16E-23 | 44.42510468 |
| DNAJC12 | 1.673102438 | 6.325788226 | 10.95930077 | 1.85E-24 | 1.18E-23 | 44.40198881 |
| SNX30 | -0.917543283 | 8.689176694 | -10.94897241 | 2.02E-24 | 1.29E-23 | 44.31605371 |
| P2RY13 | -0.764514406 | 6.065321811 | -10.94807633 | 2.03E-24 | 1.30E-23 | 44.30860008 |
| SLC6A8 | 1.125637031 | 7.362700154 | 10.94348934 | 2.11E-24 | 1.35E-23 | 44.27044999 |
| JUP | 0.814331999 | 8.965474141 | 10.94043874 | 2.17E-24 | 1.38E-23 | 44.24508265 |
| TGM2 | -0.796747124 | 8.561588582 | -10.94011398 | 2.17E-24 | 1.38E-23 | 44.24238233 |
| PLXNA3 | 0.803840833 | 7.726368141 | 10.93717786 | 2.23E-24 | 1.42E-23 | 44.2179708 |
| CCND2 | -0.906371827 | 9.371684274 | -10.93496879 | 2.27E-24 | 1.44E-23 | 44.1996064 |
| GJC1 | -0.607114067 | 6.965978854 | -10.9333319 | 2.30E-24 | 1.46E-23 | 44.18599982 |
| PIF1 | 0.742402646 | 5.563373146 | 10.93147262 | 2.34E-24 | 1.48E-23 | 44.17054602 |
| PPAP2A | -0.779004224 | 8.321384652 | -10.9116548 | 2.76E-24 | 1.75E-23 | 44.00591019 |
| EIF4EBP1 | 0.88124705 | 7.178741606 | 10.90951338 | 2.81E-24 | 1.77E-23 | 43.98812971 |
| KCTD12 | -0.793064884 | 8.358336043 | -10.90834702 | 2.84E-24 | 1.79E-23 | 43.97844605 |
| PA2G4 | 0.623091873 | 9.488836439 | 10.90534196 | 2.91E-24 | 1.83E-23 | 43.95349904 |
| INSIG1 | -0.817874701 | 7.988136489 | -10.90248649 | 2.98E-24 | 1.88E-23 | 43.92979733 |
| COL22A1 | 0.917960842 | 5.408685984 | 10.90036082 | 3.03E-24 | 1.91E-23 | 43.91215535 |
| SHCBP1 | 0.711918921 | 7.366940184 | 10.89906457 | 3.07E-24 | 1.93E-23 | 43.90139803 |
| CHRNB1 | 0.677119345 | 6.973752295 | 10.89722672 | 3.11E-24 | 1.96E-23 | 43.88614717 |
| POLE2 | 1.007892101 | 6.197263489 | 10.89146185 | 3.27E-24 | 2.05E-23 | 43.83831788 |
| LYZ | -1.457207139 | 10.60957327 | -10.89117017 | 3.27E-24 | 2.06E-23 | 43.83589825 |
| EDN2 | 0.917254224 | 5.574730997 | 10.88849432 | 3.35E-24 | 2.10E-23 | 43.81370236 |
| SELENBP1 | -1.361758948 | 10.11068427 | -10.88736084 | 3.38E-24 | 2.12E-23 | 43.80430111 |
| FA2H | 1.151587267 | 5.67961429 | 10.86211797 | 4.17E-24 | 2.61E-23 | 43.59506596 |
| ASPHD2 | 0.903873184 | 6.012712242 | 10.86030515 | 4.24E-24 | 2.65E-23 | 43.58004945 |
| HTR3A | 1.044306828 | 5.327639806 | 10.85786727 | 4.32E-24 | 2.70E-23 | 43.55985735 |
| CAPSL | -1.065995577 | 5.204152346 | -10.85124654 | 4.57E-24 | 2.85E-23 | 43.50503223 |
| MYO1F | -0.823509032 | 7.830655785 | -10.84823984 | 4.69E-24 | 2.92E-23 | 43.48014003 |
| IPO13 | 0.589851191 | 7.707647298 | 10.84669925 | 4.75E-24 | 2.96E-23 | 43.46738705 |
| COL4A3 | -0.91222523 | 6.06765041 | -10.8409767 | 4.98E-24 | 3.10E-23 | 43.42002413 |
| MYLIP | -0.695637568 | 8.909563548 | -10.83699079 | 5.15E-24 | 3.20E-23 | 43.38704237 |
| CD53 | -1.015244831 | 8.610058981 | -10.83075383 | 5.42E-24 | 3.37E-23 | 43.33544678 |
| EGR1 | -1.041921849 | 10.74111507 | -10.82927788 | 5.49E-24 | 3.41E-23 | 43.32323919 |
| DOPEY2 | 0.7444641 | 7.449484386 | 10.81628538 | 6.12E-24 | 3.79E-23 | 43.21581605 |
| PCP4 | 2.236329192 | 6.171752729 | 10.81325326 | 6.27E-24 | 3.88E-23 | 43.19075598 |
| NFE2L3 | 0.669451977 | 6.720952077 | 10.80617339 | 6.65E-24 | 4.12E-23 | 43.1322562 |
| MAPK13 | 0.643481783 | 9.591847051 | 10.80116277 | 6.94E-24 | 4.28E-23 | 43.09086649 |
| EYA4 | -0.848272705 | 4.826294407 | -10.79265793 | 7.45E-24 | 4.59E-23 | 43.02063631 |
| ROBO2 | -1.080510069 | 5.453809511 | -10.79214801 | 7.48E-24 | 4.61E-23 | 43.01642654 |
| HSPB3 | -1.021203834 | 5.289711269 | -10.78940861 | 7.65E-24 | 4.71E-23 | 42.99381225 |
| MX2 | 0.909173232 | 8.776794524 | 10.7880303 | 7.74E-24 | 4.77E-23 | 42.98243518 |
| TSPAN5 | 0.923331395 | 7.613695388 | 10.78277397 | 8.09E-24 | 4.97E-23 | 42.93905459 |
| GSTA4 | -0.911326026 | 8.312921258 | -10.78066695 | 8.23E-24 | 5.05E-23 | 42.9216685 |
| DFNB31 | 0.645559148 | 6.00380325 | 10.7794425 | 8.31E-24 | 5.10E-23 | 42.91156571 |
| KIFC2 | 0.867283641 | 7.353963758 | 10.77309799 | 8.76E-24 | 5.37E-23 | 42.85922784 |
| PITPNM1 | 0.612286652 | 8.618381112 | 10.76580987 | 9.31E-24 | 5.70E-23 | 42.79912596 |
| B3GAT3 | 0.642001568 | 7.546694723 | 10.76577383 | 9.31E-24 | 5.70E-23 | 42.79882881 |
| TGFB2 | -0.932555731 | 5.980178636 | -10.76543629 | 9.34E-24 | 5.71E-23 | 42.79604578 |
| CSE1L | 0.607719601 | 9.008641391 | 10.76212439 | 9.60E-24 | 5.86E-23 | 42.7687416 |
| TCF4 | -0.771795669 | 9.315042718 | -10.75537077 | 1.02E-23 | 6.19E-23 | 42.71307689 |
| NKG7 | -1.077603275 | 8.244340713 | -10.75188874 | 1.05E-23 | 6.37E-23 | 42.68438449 |
| IQGAP3 | 0.670652798 | 5.770126598 | 10.74207115 | 1.13E-23 | 6.89E-23 | 42.60351289 |
| PROC | 0.916061878 | 5.303628021 | 10.73701692 | 1.18E-23 | 7.18E-23 | 42.56189435 |
| ETNK2 | 0.97746628 | 5.956705625 | 10.73641187 | 1.19E-23 | 7.21E-23 | 42.55691281 |
| RAB15 | 1.009327198 | 7.207228418 | 10.73459651 | 1.21E-23 | 7.32E-23 | 42.54196746 |
| LRP8 | 0.732225613 | 6.232270096 | 10.73390167 | 1.21E-23 | 7.36E-23 | 42.53624735 |
| IL17D | -0.881360641 | 6.910137343 | -10.73052404 | 1.25E-23 | 7.56E-23 | 42.50844476 |
| AIM2 | 1.279501264 | 6.096338902 | 10.73028141 | 1.25E-23 | 7.57E-23 | 42.50644775 |
| FGFRL1 | 0.712279987 | 7.531373314 | 10.72864996 | 1.27E-23 | 7.67E-23 | 42.49302042 |
| VNN2 | -0.922657836 | 5.986652654 | -10.71557973 | 1.41E-23 | 8.54E-23 | 42.38548806 |
| EMILIN2 | -0.771070502 | 9.169295617 | -10.71217793 | 1.45E-23 | 8.78E-23 | 42.35751193 |
| CHD1L | 0.591562826 | 7.907445149 | 10.71190867 | 1.46E-23 | 8.79E-23 | 42.35529784 |
| CYGB | -0.628557101 | 8.142794843 | -10.70933666 | 1.49E-23 | 8.97E-23 | 42.33414926 |
| NUP43 | 0.592873496 | 8.402607205 | 10.70815959 | 1.50E-23 | 9.06E-23 | 42.32447164 |
| HIST1H2BC | 0.951669601 | 6.009360226 | 10.70463181 | 1.55E-23 | 9.32E-23 | 42.29547028 |
| MAOA | -1.03127659 | 9.958177798 | -10.70386858 | 1.56E-23 | 9.37E-23 | 42.28919656 |
| OSBPL6 | -0.69355575 | 6.08203679 | -10.69993754 | 1.61E-23 | 9.67E-23 | 42.25688742 |
| RRM1 | 0.602818887 | 9.387506979 | 10.69685269 | 1.65E-23 | 9.91E-23 | 42.23153742 |
| BPNT1 | 0.773390129 | 7.95705991 | 10.69556354 | 1.67E-23 | 1.00E-22 | 42.22094496 |
| MXRA5 | 1.112636256 | 9.960822298 | 10.69488159 | 1.68E-23 | 1.01E-22 | 42.21534191 |
| XRCC2 | 0.799778145 | 7.114595949 | 10.68876808 | 1.76E-23 | 1.06E-22 | 42.16512023 |
| RARRES2 | -0.857348303 | 10.47635256 | -10.6880421 | 1.77E-23 | 1.06E-22 | 42.15915744 |
| GPRC5A | -1.190060388 | 9.832876854 | -10.68655422 | 1.80E-23 | 1.07E-22 | 42.1469375 |
| ICA1 | 0.641743372 | 7.433774481 | 10.66978757 | 2.06E-23 | 1.23E-22 | 42.00929611 |
| CAPN3 | -0.754839884 | 6.442368566 | -10.66719451 | 2.11E-23 | 1.26E-22 | 41.9880194 |
| CD37 | -1.124654173 | 8.26023054 | -10.66182779 | 2.20E-23 | 1.32E-22 | 41.94399295 |
| TRIM22 | -0.857662234 | 9.267531806 | -10.65890352 | 2.26E-23 | 1.35E-22 | 41.92000838 |
| CXCL16 | -0.685682295 | 10.45909785 | -10.65714924 | 2.29E-23 | 1.37E-22 | 41.90562167 |
| LAPTM4B | 0.84529563 | 10.78493287 | 10.64757611 | 2.48E-23 | 1.47E-22 | 41.82713558 |
| PCDH17 | -0.878618997 | 9.027816963 | -10.64043012 | 2.63E-23 | 1.56E-22 | 41.7685733 |
| NCAPG2 | 0.73314631 | 5.941167963 | 10.63938923 | 2.65E-23 | 1.58E-22 | 41.76004482 |
| BTG1 | -0.59354474 | 11.48676407 | -10.63862614 | 2.67E-23 | 1.58E-22 | 41.75379277 |
| PARVA | -0.600674183 | 7.969913883 | -10.63829339 | 2.68E-23 | 1.59E-22 | 41.75106662 |
| ITGAL | -0.896633146 | 7.81870987 | -10.63311324 | 2.80E-23 | 1.65E-22 | 41.70863249 |
| SPINT1 | 0.809755082 | 8.627235149 | 10.62922637 | 2.89E-23 | 1.71E-22 | 41.67679984 |
| APIP | -0.63411178 | 7.681839721 | -10.62680407 | 2.94E-23 | 1.74E-22 | 41.65696481 |
| VASH1 | -0.630511545 | 7.939329521 | -10.61862716 | 3.15E-23 | 1.86E-22 | 41.59002633 |
| FREM1 | -0.69869582 | 5.298850755 | -10.61370033 | 3.28E-23 | 1.94E-22 | 41.54970728 |
| MTFR1 | 0.798647529 | 8.055849056 | 10.61313973 | 3.30E-23 | 1.94E-22 | 41.5451202 |
| HIST1H2AM | 0.745483043 | 4.664663649 | 10.60314686 | 3.58E-23 | 2.11E-22 | 41.46337635 |
| TBC1D7 | 0.61442527 | 7.978867093 | 10.59179897 | 3.93E-23 | 2.31E-22 | 41.37059863 |
| TARS | 0.675551707 | 9.021208859 | 10.58980315 | 4.00E-23 | 2.35E-22 | 41.35428682 |
| NOV | -1.039326731 | 6.686076609 | -10.58137753 | 4.28E-23 | 2.51E-22 | 41.28544273 |
| RAMP1 | 0.974595006 | 7.819350657 | 10.58091572 | 4.30E-23 | 2.52E-22 | 41.28167028 |
| ISG20L2 | 0.603617933 | 8.668207309 | 10.56530007 | 4.89E-23 | 2.86E-22 | 41.15415956 |
| TNS3 | -0.660052526 | 10.61818685 | -10.56510726 | 4.90E-23 | 2.86E-22 | 41.15258572 |
| GGCX | 0.696765964 | 7.946102378 | 10.5638653 | 4.95E-23 | 2.89E-22 | 41.14244894 |
| SGIP1 | -0.928386559 | 6.164864021 | -10.56343403 | 4.96E-23 | 2.90E-22 | 41.13892908 |
| DLL1 | -1.019826456 | 7.29242591 | -10.56200942 | 5.02E-23 | 2.93E-22 | 41.12730253 |
| DARS2 | 0.793984793 | 7.320959346 | 10.55862977 | 5.16E-23 | 3.01E-22 | 41.09972387 |
| WDHD1 | 0.638542733 | 5.299538622 | 10.55793158 | 5.19E-23 | 3.03E-22 | 41.09402711 |
| NLRP1 | -0.600640219 | 5.668663096 | -10.55706738 | 5.23E-23 | 3.05E-22 | 41.08697608 |
| CCDC58 | 0.840469506 | 7.485068636 | 10.55626401 | 5.27E-23 | 3.07E-22 | 41.08042169 |
| C1orf53 | 0.789896663 | 7.497489298 | 10.55023848 | 5.53E-23 | 3.22E-22 | 41.03127002 |
| BCL9L | 0.764766656 | 7.383510505 | 10.55021425 | 5.53E-23 | 3.22E-22 | 41.0310724 |
| CAPN12 | 1.107324972 | 7.105414529 | 10.54784925 | 5.64E-23 | 3.28E-22 | 41.0117847 |
| LCAT | -0.728076879 | 6.532829013 | -10.54634824 | 5.71E-23 | 3.32E-22 | 40.99954454 |
| BMP2 | -1.276452212 | 8.063353487 | -10.53125197 | 6.47E-23 | 3.75E-22 | 40.87649261 |
| EVI2B | -0.810486021 | 7.975061072 | -10.52935692 | 6.57E-23 | 3.80E-22 | 40.86105255 |
| CD58 | -0.665689866 | 8.296692152 | -10.51613023 | 7.32E-23 | 4.23E-22 | 40.75332948 |
| FCER1G | -1.078930949 | 10.07090638 | -10.51390541 | 7.46E-23 | 4.30E-22 | 40.735217 |
| KCNK5 | 1.189011862 | 7.118794043 | 10.50964837 | 7.72E-23 | 4.45E-22 | 40.7005659 |
| GMDS | 0.765275169 | 8.920210048 | 10.50908219 | 7.76E-23 | 4.47E-22 | 40.69595794 |
| WISP1 | 0.757379689 | 5.551718263 | 10.50615237 | 7.95E-23 | 4.58E-22 | 40.67211509 |
| TMEM37 | -1.036242261 | 7.331948646 | -10.5024005 | 8.20E-23 | 4.72E-22 | 40.64158781 |
| CNKSR1 | 0.636838767 | 6.635448832 | 10.50109071 | 8.29E-23 | 4.76E-22 | 40.63093206 |
| TIGD2 | 0.712913485 | 6.509717037 | 10.49869663 | 8.45E-23 | 4.85E-22 | 40.61145696 |
| CDK5R1 | 0.797554498 | 5.381590324 | 10.49502506 | 8.71E-23 | 5.00E-22 | 40.58159459 |
| ARL13B | -0.610988826 | 6.148192319 | -10.49441478 | 8.75E-23 | 5.02E-22 | 40.5766315 |
| NTRK2 | -1.145246592 | 5.326173976 | -10.48651819 | 9.34E-23 | 5.35E-22 | 40.51242684 |
| MAML2 | -0.659021153 | 6.81991009 | -10.48419304 | 9.52E-23 | 5.44E-22 | 40.49352679 |
| IRF1 | -0.875778657 | 9.489270247 | -10.48405059 | 9.53E-23 | 5.45E-22 | 40.49236899 |
| GAD1 | 0.987627601 | 4.891855386 | 10.48242492 | 9.66E-23 | 5.52E-22 | 40.47915617 |
| EPHX2 | -0.862586639 | 6.563629428 | -10.4785485 | 9.97E-23 | 5.69E-22 | 40.44765465 |
| PNMA6A | 0.893621897 | 5.558983481 | 10.47796009 | 1.00E-22 | 5.71E-22 | 40.44287352 |
| MRPL2 | 0.643336011 | 7.584737992 | 10.47766221 | 1.00E-22 | 5.72E-22 | 40.44045318 |
| KLF15 | -0.743531543 | 6.710156274 | -10.4764823 | 1.01E-22 | 5.77E-22 | 40.4308664 |
| AKAP14 | -0.846147467 | 4.245316673 | -10.47510867 | 1.03E-22 | 5.84E-22 | 40.41970648 |
| PCSK4 | 0.675145004 | 5.92807837 | 10.47136779 | 1.06E-22 | 6.01E-22 | 40.389318 |
| ULK2 | -0.689415428 | 6.366977636 | -10.47108535 | 1.06E-22 | 6.02E-22 | 40.38702387 |
| ZNF341 | 0.699450901 | 6.774032793 | 10.45942168 | 1.17E-22 | 6.62E-22 | 40.29231544 |
| GATA6 | -0.846366403 | 6.753373165 | -10.45536382 | 1.21E-22 | 6.84E-22 | 40.25937951 |
| CYP26B1 | -1.040689558 | 6.459884109 | -10.4437131 | 1.33E-22 | 7.52E-22 | 40.16485448 |
| KRT16 | 1.295764485 | 6.179026771 | 10.44302772 | 1.33E-22 | 7.56E-22 | 40.15929568 |
| CAMK2N1 | -1.19684313 | 9.522098184 | -10.44145723 | 1.35E-22 | 7.64E-22 | 40.14655877 |
| DAAM2 | -0.894427001 | 7.176366529 | -10.44089953 | 1.36E-22 | 7.67E-22 | 40.14203608 |
| ITGA5 | -0.765326694 | 9.439576457 | -10.4396156 | 1.37E-22 | 7.75E-22 | 40.13162436 |
| UTRN | -0.679818778 | 7.840078681 | -10.43743069 | 1.40E-22 | 7.88E-22 | 40.11390798 |
| MAST1 | 0.631540912 | 5.051019705 | 10.43604939 | 1.41E-22 | 7.97E-22 | 40.10270874 |
| GPC2 | 0.585473329 | 5.182933492 | 10.43560182 | 1.42E-22 | 7.99E-22 | 40.09908012 |
| SPSB1 | 0.807764722 | 8.161118239 | 10.40761618 | 1.78E-22 | 1.00E-21 | 39.87236145 |
| B3GNT4 | 0.640882618 | 5.628818535 | 10.4059697 | 1.80E-22 | 1.01E-21 | 39.85903336 |
| GRB7 | 0.657011112 | 6.95090304 | 10.40141215 | 1.87E-22 | 1.05E-21 | 39.8221467 |
| OTUB2 | 0.621155407 | 5.848647894 | 10.3980063 | 1.93E-22 | 1.08E-21 | 39.79458716 |
| FAM101B | -0.776390889 | 7.147335867 | -10.39618361 | 1.95E-22 | 1.10E-21 | 39.77984033 |
| GZMH | -0.915279982 | 7.465571202 | -10.38701647 | 2.11E-22 | 1.18E-21 | 39.70569351 |
| CARD11 | 0.669732086 | 7.750781691 | 10.37940794 | 2.24E-22 | 1.25E-21 | 39.64418089 |
| C15orf48 | 1.370209115 | 7.228210819 | 10.37461206 | 2.33E-22 | 1.30E-21 | 39.60542059 |
| KCNK12 | 1.252887775 | 5.928605279 | 10.37373943 | 2.35E-22 | 1.31E-21 | 39.598369 |
| SLC35A1 | -0.696221853 | 9.133988532 | -10.36959221 | 2.43E-22 | 1.35E-21 | 39.56486071 |
| MPHOSPH9 | 0.666810765 | 6.41547162 | 10.36739115 | 2.47E-22 | 1.38E-21 | 39.54707986 |
| TMPRSS6 | 1.126033352 | 5.785701324 | 10.36722227 | 2.48E-22 | 1.38E-21 | 39.54571569 |
| MBP | -0.604052205 | 7.830617295 | -10.36719826 | 2.48E-22 | 1.38E-21 | 39.54552178 |
| MGLL | -0.874135377 | 8.393602779 | -10.3637949 | 2.55E-22 | 1.41E-21 | 39.51803274 |
| CD163 | -1.134806071 | 8.635730734 | -10.35818902 | 2.66E-22 | 1.48E-21 | 39.4727649 |
| PLAU | 1.193651957 | 9.313075391 | 10.35787178 | 2.67E-22 | 1.48E-21 | 39.47020359 |
| IRF8 | -0.89040978 | 9.164168668 | -10.35572728 | 2.72E-22 | 1.51E-21 | 39.45289051 |
| LPGAT1 | 0.622882885 | 7.011888548 | 10.35529794 | 2.73E-22 | 1.51E-21 | 39.44942452 |
| ALDH3B1 | -0.745288978 | 7.772730665 | -10.34945673 | 2.86E-22 | 1.58E-21 | 39.40227828 |
| PIK3C2A | -0.707010931 | 7.205829585 | -10.34820693 | 2.89E-22 | 1.60E-21 | 39.39219261 |
| KRT86 | 0.863267529 | 5.999118758 | 10.34663665 | 2.93E-22 | 1.62E-21 | 39.37952183 |
| LMBRD1 | -0.67623647 | 9.823906098 | -10.34495029 | 2.97E-22 | 1.64E-21 | 39.36591547 |
| UBAP2L | 0.642966609 | 8.805430798 | 10.342953 | 3.02E-22 | 1.67E-21 | 39.34980205 |
| PUS7L | 0.667470795 | 5.99721837 | 10.33926777 | 3.11E-22 | 1.71E-21 | 39.32007543 |
| LAIR1 | -0.616132634 | 9.025993936 | -10.33026853 | 3.34E-22 | 1.84E-21 | 39.2475086 |
| NFKBIA | -0.651520307 | 12.06641648 | -10.32881069 | 3.38E-22 | 1.86E-21 | 39.23575638 |
| NFIL3 | -0.810806273 | 8.647523386 | -10.32692686 | 3.44E-22 | 1.89E-21 | 39.22057149 |
| XKRX | 1.525218019 | 6.237330394 | 10.32610736 | 3.46E-22 | 1.90E-21 | 39.2139663 |
| SOX12 | 0.6238306 | 6.083521418 | 10.32360316 | 3.53E-22 | 1.94E-21 | 39.1937841 |
| APLN | -1.019346575 | 6.45289009 | -10.32324647 | 3.54E-22 | 1.94E-21 | 39.19090964 |
| FBXL7 | -0.677439826 | 6.202616165 | -10.32182102 | 3.58E-22 | 1.97E-21 | 39.17942293 |
| CPNE4 | 1.116928113 | 4.93716934 | 10.31773584 | 3.70E-22 | 2.03E-21 | 39.14650822 |
| CLIC4 | -0.661453519 | 9.1854035 | -10.3137159 | 3.83E-22 | 2.10E-21 | 39.11412616 |
| BNC2 | -0.89114249 | 6.654427503 | -10.29507141 | 4.45E-22 | 2.43E-21 | 38.96403096 |
| BARD1 | 0.788732396 | 7.680268867 | 10.29287153 | 4.53E-22 | 2.47E-21 | 38.94633113 |
| PPARGC1A | -1.402448584 | 6.365719303 | -10.28745304 | 4.73E-22 | 2.58E-21 | 38.90274406 |
| KLRB1 | -1.031761629 | 7.374330479 | -10.27560181 | 5.21E-22 | 2.84E-21 | 38.80745611 |
| NPAS2 | 0.868488104 | 6.596232934 | 10.27422353 | 5.27E-22 | 2.87E-21 | 38.79637829 |
| CD9 | -0.75335293 | 10.72760515 | -10.2669005 | 5.59E-22 | 3.04E-21 | 38.73753388 |
| GALNT3 | 1.253923139 | 8.227369298 | 10.26349161 | 5.75E-22 | 3.13E-21 | 38.71014976 |
| IGFBP7 | -0.796471154 | 11.1671754 | -10.25194718 | 6.31E-22 | 3.43E-21 | 38.61744967 |
| SPON1 | -1.051356591 | 8.737255819 | -10.25124229 | 6.35E-22 | 3.45E-21 | 38.61179144 |
| MFAP2 | 0.621651735 | 7.067647862 | 10.24836522 | 6.50E-22 | 3.53E-21 | 38.58869903 |
| TMTC4 | 0.62473234 | 6.415076747 | 10.2348182 | 7.25E-22 | 3.93E-21 | 38.48001504 |
| RNF24 | 0.620013821 | 8.118719008 | 10.23337136 | 7.34E-22 | 3.98E-21 | 38.46841219 |
| FHL2 | 0.845373173 | 7.827594859 | 10.2180967 | 8.30E-22 | 4.49E-21 | 38.34597516 |
| DUS4L | 0.59231986 | 6.648363027 | 10.21788764 | 8.32E-22 | 4.50E-21 | 38.3443001 |
| SLC7A1 | 0.634527204 | 8.335642367 | 10.21661703 | 8.40E-22 | 4.54E-21 | 38.33412004 |
| CCDC47 | 0.7363043 | 8.07452071 | 10.2127641 | 8.67E-22 | 4.68E-21 | 38.303255 |
| CNTN4 | -0.643461472 | 4.776162407 | -10.21264889 | 8.68E-22 | 4.69E-21 | 38.3023322 |
| P2RY1 | -0.730364611 | 5.051783423 | -10.19628643 | 9.90E-22 | 5.34E-21 | 38.17133036 |
| ELMOD2 | 0.635590084 | 7.098544492 | 10.19398861 | 1.01E-21 | 5.43E-21 | 38.15294298 |
| HAMP | 1.157477656 | 6.220780088 | 10.18939393 | 1.05E-21 | 5.64E-21 | 38.11618298 |
| SLC4A11 | 1.213718353 | 6.494464931 | 10.17925843 | 1.14E-21 | 6.11E-21 | 38.0351267 |
| IGF2BP3 | 1.756079272 | 6.060996106 | 10.17757833 | 1.15E-21 | 6.19E-21 | 38.02169494 |
| WDR34 | 0.658528797 | 8.587749689 | 10.17331139 | 1.19E-21 | 6.40E-21 | 37.98758803 |
| EPHA1 | 0.698830054 | 7.497204944 | 10.16545059 | 1.27E-21 | 6.81E-21 | 37.92477568 |
| FAM57A | 0.609769665 | 8.346206894 | 10.15408501 | 1.39E-21 | 7.43E-21 | 37.83400703 |
| SNRPN | -0.718283769 | 7.547722447 | -10.15322764 | 1.40E-21 | 7.48E-21 | 37.82716215 |
| RUNX2 | 0.658211546 | 5.967916965 | 10.14709631 | 1.47E-21 | 7.86E-21 | 37.77822215 |
| FOXA3 | 1.25192663 | 5.914869407 | 10.14691327 | 1.47E-21 | 7.86E-21 | 37.77676137 |
| SGOL2 | 0.656542266 | 5.216748412 | 10.14383354 | 1.51E-21 | 8.06E-21 | 37.75218569 |
| GNL3L | 0.714561352 | 8.645873082 | 10.13972593 | 1.56E-21 | 8.32E-21 | 37.71941441 |
| XPNPEP3 | 0.644444777 | 8.239120787 | 10.12874677 | 1.71E-21 | 9.08E-21 | 37.63185771 |
| ST6GALNAC2 | -1.108465022 | 7.641499638 | -10.12630781 | 1.74E-21 | 9.25E-21 | 37.61241487 |
| FOXRED2 | 0.649797345 | 7.098913726 | 10.12146977 | 1.81E-21 | 9.61E-21 | 37.57385504 |
| IFIT2 | -0.870691525 | 8.136601859 | -10.12026811 | 1.83E-21 | 9.70E-21 | 37.56427927 |
| EGFL6 | -0.859071005 | 7.874757771 | -10.11653258 | 1.88E-21 | 9.99E-21 | 37.53451584 |
| SPIRE2 | 0.897097865 | 7.150301271 | 10.11551689 | 1.90E-21 | 1.01E-20 | 37.52642425 |
| PACS1 | 0.735725133 | 7.799879596 | 10.11505235 | 1.91E-21 | 1.01E-20 | 37.52272357 |
| MS4A4A | -0.805619318 | 6.703851301 | -10.11462642 | 1.91E-21 | 1.01E-20 | 37.51933058 |
| SLC16A6 | -0.793078647 | 5.443980471 | -10.10194396 | 2.12E-21 | 1.12E-20 | 37.41833894 |
| TTN | -0.619918362 | 4.919261016 | -10.0946316 | 2.24E-21 | 1.19E-20 | 37.36014305 |
| F2RL1 | 0.924909385 | 7.387651266 | 10.09233568 | 2.29E-21 | 1.21E-20 | 37.34187575 |
| RBM12B | 0.644307846 | 7.118509194 | 10.08859899 | 2.36E-21 | 1.24E-20 | 37.31215034 |
| TM4SF18 | -1.196518158 | 6.945540731 | -10.08792502 | 2.37E-21 | 1.25E-20 | 37.30678953 |
| MST1R | 0.899100965 | 7.478819324 | 10.08066706 | 2.51E-21 | 1.32E-20 | 37.24907256 |
| NEIL3 | 0.871862928 | 5.007933019 | 10.0780123 | 2.57E-21 | 1.35E-20 | 37.2279673 |
| RBM28 | 0.598676718 | 7.066035165 | 10.07752344 | 2.58E-21 | 1.35E-20 | 37.22408117 |
| JUNB | -0.844972978 | 10.35176432 | -10.07214085 | 2.69E-21 | 1.41E-20 | 37.18130071 |
| CTGF | -0.894552899 | 11.28295812 | -10.07109493 | 2.71E-21 | 1.42E-20 | 37.17298933 |
| PCDH7 | 0.845878944 | 5.729170516 | 10.0651142 | 2.84E-21 | 1.49E-20 | 37.12547324 |
| TGFA | 1.049322684 | 6.790027226 | 10.06066233 | 2.95E-21 | 1.54E-20 | 37.09011428 |
| FAM81A | 0.726570617 | 4.998507585 | 10.05288853 | 3.14E-21 | 1.64E-20 | 37.02839254 |
| USP53 | -0.700326655 | 6.778123003 | -10.05075503 | 3.19E-21 | 1.67E-20 | 37.0114579 |
| CASC5 | 0.70591149 | 5.049353037 | 10.03931558 | 3.50E-21 | 1.82E-20 | 36.92069328 |
| CYR61 | -1.042607656 | 10.63570897 | -10.03631827 | 3.58E-21 | 1.87E-20 | 36.89692138 |
| VLDLR | -0.618943194 | 6.541919582 | -10.03111348 | 3.73E-21 | 1.94E-20 | 36.85565167 |
| ETV5 | -0.743288065 | 8.220141891 | -10.01804548 | 4.15E-21 | 2.16E-20 | 36.75208767 |
| PCDH18 | -0.80544993 | 6.926343734 | -10.01302481 | 4.32E-21 | 2.24E-20 | 36.71231969 |
| TDRKH | 0.803684236 | 6.031056981 | 10.00831174 | 4.48E-21 | 2.32E-20 | 36.67499866 |
| RPL21 | -0.609036567 | 11.01277854 | -10.00548707 | 4.58E-21 | 2.38E-20 | 36.65263605 |
| SLC14A1 | -0.905878385 | 5.175573633 | -10.00009982 | 4.79E-21 | 2.48E-20 | 36.60999593 |
| FAM83E | 0.874337213 | 7.166747753 | 9.994904454 | 4.99E-21 | 2.58E-20 | 36.56888718 |
| SGCB | -0.687786176 | 7.052664862 | -9.990974532 | 5.15E-21 | 2.66E-20 | 36.53779963 |
| IL16 | -0.609982014 | 6.296074532 | -9.990070564 | 5.18E-21 | 2.68E-20 | 36.53064982 |
| C2 | -0.906306144 | 8.575571295 | -9.985770779 | 5.37E-21 | 2.77E-20 | 36.49664642 |
| BARX2 | 0.739478722 | 5.72222325 | 9.978535088 | 5.68E-21 | 2.93E-20 | 36.43944462 |
| C10orf11 | -0.6672426 | 7.583793168 | -9.978367495 | 5.69E-21 | 2.94E-20 | 36.43812 |
| DLL3 | 0.788677369 | 4.914344191 | 9.966805906 | 6.24E-21 | 3.21E-20 | 36.34677084 |
| FCGR2A | -0.796461676 | 7.896484926 | -9.964909236 | 6.34E-21 | 3.26E-20 | 36.33179096 |
| HOXC9 | 1.062853735 | 4.752922088 | 9.955511886 | 6.83E-21 | 3.51E-20 | 36.25759541 |
| TFRC | -0.735596256 | 10.55961535 | -9.940505914 | 7.70E-21 | 3.95E-20 | 36.1392025 |
| FIGNL1 | 0.623437721 | 6.287634396 | 9.939812707 | 7.74E-21 | 3.97E-20 | 36.13373582 |
| SLFN11 | -0.780376293 | 8.722609149 | -9.937315411 | 7.89E-21 | 4.04E-20 | 36.11404379 |
| SDSL | 0.616778498 | 7.884811598 | 9.936343876 | 7.96E-21 | 4.07E-20 | 36.10638369 |
| CSF3 | -0.853438562 | 5.85002721 | -9.929403591 | 8.41E-21 | 4.30E-20 | 36.05167554 |
| TNFRSF19 | -0.685578656 | 6.518591673 | -9.928247199 | 8.49E-21 | 4.34E-20 | 36.04256222 |
| NEGR1 | -0.660551493 | 4.863472992 | -9.917718558 | 9.23E-21 | 4.71E-20 | 35.95961657 |
| AHDC1 | 0.874742176 | 6.822666779 | 9.912735612 | 9.60E-21 | 4.90E-20 | 35.92037845 |
| CLEC7A | -0.69049438 | 5.966865101 | -9.909493945 | 9.85E-21 | 5.02E-20 | 35.8948582 |
| SCD5 | -0.587352197 | 6.459282112 | -9.909069117 | 9.88E-21 | 5.03E-20 | 35.89151407 |
| COMT | -0.587088195 | 9.15178888 | -9.903033427 | 1.04E-20 | 5.27E-20 | 35.84401194 |
| ILDR1 | 0.599466714 | 7.793719984 | 9.89703909 | 1.09E-20 | 5.52E-20 | 35.7968521 |
| KIF12 | 0.971576375 | 5.951077471 | 9.885822427 | 1.19E-20 | 6.02E-20 | 35.70865124 |
| HSD17B1 | 0.889156622 | 6.088530949 | 9.881768274 | 1.23E-20 | 6.22E-20 | 35.67678639 |
| HRASLS | 1.075477857 | 5.020245612 | 9.881752509 | 1.23E-20 | 6.22E-20 | 35.67666249 |
| FKBP1B | -0.685753615 | 6.731356165 | -9.880534725 | 1.24E-20 | 6.27E-20 | 35.66709246 |
| FGL2 | -0.984571553 | 8.790319516 | -9.875862064 | 1.29E-20 | 6.50E-20 | 35.63037851 |
| RLTPR | 0.611749333 | 5.54530984 | 9.873688683 | 1.31E-20 | 6.60E-20 | 35.61330534 |
| THNSL1 | 0.709475912 | 6.357372481 | 9.872304481 | 1.32E-20 | 6.67E-20 | 35.60243279 |
| UGT8 | 0.8143578 | 4.986359582 | 9.872190338 | 1.32E-20 | 6.67E-20 | 35.60153626 |
| LEMD1 | 1.344379427 | 5.33648338 | 9.863826625 | 1.42E-20 | 7.12E-20 | 35.535861 |
| TMEM63C | 0.758451731 | 5.231479279 | 9.849833072 | 1.58E-20 | 7.95E-20 | 35.42605144 |
| DOCK8 | -0.781109777 | 8.046505513 | -9.843337425 | 1.66E-20 | 8.36E-20 | 35.3751104 |
| CEACAM5 | 2.470219076 | 7.934708016 | 9.841225305 | 1.69E-20 | 8.50E-20 | 35.35855074 |
| SLC22A4 | -0.992407234 | 5.941804521 | -9.830529704 | 1.84E-20 | 9.24E-20 | 35.27472623 |
| NR3C2 | -0.907342645 | 7.291373609 | -9.829651281 | 1.85E-20 | 9.30E-20 | 35.26784417 |
| RHPN2 | 0.967475434 | 8.644086707 | 9.82495644 | 1.93E-20 | 9.64E-20 | 35.23106835 |
| MYEOV | 1.341990726 | 5.803063213 | 9.82029785 | 2.00E-20 | 9.99E-20 | 35.19458678 |
| IL1A | -1.224038568 | 5.347338612 | -9.810601778 | 2.16E-20 | 1.08E-19 | 35.11868943 |
| RBP7 | -0.949051436 | 6.453348668 | -9.810494176 | 2.16E-20 | 1.08E-19 | 35.11784741 |
| RGS2 | -0.974052827 | 10.62830637 | -9.810378024 | 2.16E-20 | 1.08E-19 | 35.11693849 |
| IFI30 | -0.858628025 | 10.76250763 | -9.809690924 | 2.17E-20 | 1.08E-19 | 35.11156188 |
| TLK1 | 0.618702713 | 8.075030963 | 9.808185683 | 2.20E-20 | 1.10E-19 | 35.09978403 |
| CPD | 0.781700075 | 10.03161608 | 9.806491149 | 2.23E-20 | 1.11E-19 | 35.08652634 |
| CRTC1 | 0.709374937 | 6.551720359 | 9.798407869 | 2.37E-20 | 1.18E-19 | 35.02330312 |
| WDR78 | -0.658732807 | 4.950957013 | -9.798224371 | 2.38E-20 | 1.18E-19 | 35.02186825 |
| HIST1H2BG | 0.937204184 | 5.762700122 | 9.796659348 | 2.41E-20 | 1.20E-19 | 35.00963116 |
| JAK2 | -0.681281169 | 7.07039179 | -9.793504042 | 2.47E-20 | 1.23E-19 | 34.98496302 |
| IL23A | 0.674883836 | 5.385564809 | 9.789467515 | 2.55E-20 | 1.27E-19 | 34.9534124 |
| CLEC4E | -0.648561416 | 5.230128755 | -9.780404902 | 2.74E-20 | 1.36E-19 | 34.88260468 |
| MPDZ | -0.783892296 | 7.846676109 | -9.768034988 | 3.02E-20 | 1.49E-19 | 34.78601951 |
| LCOR | 0.783467307 | 8.284909569 | 9.767468999 | 3.03E-20 | 1.50E-19 | 34.78160198 |
| KCNK1 | 1.078384024 | 7.521477612 | 9.765249493 | 3.08E-20 | 1.52E-19 | 34.76428023 |
| PENK | -1.244093853 | 5.906215508 | -9.750575384 | 3.46E-20 | 1.71E-19 | 34.64981782 |
| COL5A1 | 0.873947618 | 9.365457035 | 9.749434579 | 3.49E-20 | 1.72E-19 | 34.64092351 |
| HABP2 | 1.481525558 | 5.974031316 | 9.748439889 | 3.52E-20 | 1.73E-19 | 34.6331689 |
| TSPAN2 | -0.652285194 | 5.451878691 | -9.740341103 | 3.75E-20 | 1.84E-19 | 34.57004828 |
| CDKN1C | -0.77599764 | 8.056671255 | -9.739285368 | 3.78E-20 | 1.86E-19 | 34.56182237 |
| ENPP4 | -0.765515467 | 7.782188819 | -9.738868698 | 3.80E-20 | 1.86E-19 | 34.55857597 |
| SLC24A3 | -0.79658509 | 6.946931489 | -9.733065674 | 3.97E-20 | 1.95E-19 | 34.51337154 |
| PLA2G3 | -0.934185592 | 6.126429896 | -9.730429914 | 4.06E-20 | 1.99E-19 | 34.49284481 |
| KCNRG | -0.88908288 | 5.660970851 | -9.723177049 | 4.29E-20 | 2.10E-19 | 34.43637825 |
| PDPN | -0.669925335 | 6.952793912 | -9.719288628 | 4.43E-20 | 2.16E-19 | 34.40611567 |
| CTTN | 0.606397109 | 8.739175372 | 9.715847864 | 4.55E-20 | 2.22E-19 | 34.37934314 |
| RAVER2 | -0.743429546 | 7.29942059 | -9.714102476 | 4.61E-20 | 2.25E-19 | 34.36576448 |
| CARD8 | -0.599287995 | 7.532303556 | -9.711815769 | 4.70E-20 | 2.29E-19 | 34.34797672 |
| CABP4 | 0.84183711 | 5.871886223 | 9.709228218 | 4.79E-20 | 2.33E-19 | 34.32785179 |
| ALPL | -1.35522819 | 9.365781386 | -9.706601976 | 4.89E-20 | 2.38E-19 | 34.30742923 |
| PGAP1 | -0.706318944 | 5.854565455 | -9.704465497 | 4.97E-20 | 2.42E-19 | 34.29081768 |
| TNFAIP3 | -0.900873224 | 9.095281178 | -9.698944327 | 5.19E-20 | 2.52E-19 | 34.24789966 |
| GOLPH3L | 0.620600241 | 8.73000696 | 9.697918839 | 5.24E-20 | 2.54E-19 | 34.23992979 |
| PPP1R14D | 1.187392205 | 6.158323785 | 9.697672927 | 5.25E-20 | 2.54E-19 | 34.23801869 |
| DCBLD1 | 0.737766776 | 7.792630386 | 9.69745994 | 5.26E-20 | 2.55E-19 | 34.23636349 |
| TIMM8A | 0.680370725 | 5.870828511 | 9.695267446 | 5.35E-20 | 2.59E-19 | 34.2193261 |
| LYPD3 | 1.101427194 | 6.616468106 | 9.690022292 | 5.57E-20 | 2.70E-19 | 34.17857653 |
| FAM89A | -0.670371348 | 7.95054992 | -9.684682681 | 5.81E-20 | 2.81E-19 | 34.13710677 |
| TMEM63A | 0.715129011 | 8.278650481 | 9.678510225 | 6.10E-20 | 2.94E-19 | 34.0891859 |
| MCAM | -0.689347867 | 6.929318577 | -9.6750124 | 6.27E-20 | 3.02E-19 | 34.06203814 |
| GMCL1 | 0.662987972 | 7.739776963 | 9.673032631 | 6.37E-20 | 3.07E-19 | 34.04667513 |
| ACOXL | -0.83869654 | 5.474284529 | -9.672027797 | 6.42E-20 | 3.09E-19 | 34.03887834 |
| YBX2 | 0.976432768 | 6.116404803 | 9.667937516 | 6.62E-20 | 3.19E-19 | 34.00714573 |
| DNAJC3 | 0.726764617 | 7.190386309 | 9.663119195 | 6.88E-20 | 3.31E-19 | 33.96977535 |
| CPVL | -0.897327359 | 8.873835968 | -9.655765644 | 7.29E-20 | 3.50E-19 | 33.91276365 |
| PACSIN1 | 0.722552162 | 5.465338912 | 9.644564746 | 7.95E-20 | 3.82E-19 | 33.82597405 |
| SLN | -1.200398905 | 5.671327899 | -9.637036471 | 8.44E-20 | 4.04E-19 | 33.76767581 |
| SGCD | -0.594709345 | 5.432994412 | -9.634883249 | 8.58E-20 | 4.10E-19 | 33.75100653 |
| POU2F1 | 0.643252164 | 7.235615939 | 9.633499336 | 8.67E-20 | 4.14E-19 | 33.74029408 |
| TMEM108 | -0.890761846 | 6.862507699 | -9.630640041 | 8.87E-20 | 4.23E-19 | 33.71816411 |
| FCRL5 | 0.906263242 | 5.287944753 | 9.625701444 | 9.22E-20 | 4.39E-19 | 33.67995042 |
| AP1S1 | 0.667631072 | 7.358879795 | 9.620902087 | 9.57E-20 | 4.55E-19 | 33.6428255 |
| TRIB3 | 0.638084106 | 7.832834072 | 9.611515596 | 1.03E-19 | 4.89E-19 | 33.57024976 |
| ANKS3 | 0.63537492 | 6.473638008 | 9.610945566 | 1.03E-19 | 4.91E-19 | 33.56584371 |
| DNAI1 | -1.110043614 | 5.583390394 | -9.609275546 | 1.05E-19 | 4.97E-19 | 33.55293618 |
| SFRP1 | -1.356863 | 6.079789673 | -9.603423583 | 1.10E-19 | 5.20E-19 | 33.50771734 |
| LAPTM5 | -0.776066732 | 11.08564959 | -9.602571592 | 1.10E-19 | 5.23E-19 | 33.50113529 |
| KMO | -0.779297594 | 5.661484324 | -9.60160511 | 1.11E-19 | 5.26E-19 | 33.49366917 |
| LRRC15 | 1.101526103 | 7.272686231 | 9.596793913 | 1.16E-19 | 5.46E-19 | 33.45650926 |
| GMNN | 0.848101241 | 7.223025141 | 9.593282111 | 1.19E-19 | 5.61E-19 | 33.42939254 |
| CYBB | -0.987235383 | 9.405421088 | -9.590711304 | 1.21E-19 | 5.72E-19 | 33.40954562 |
| SLC2A12 | -0.979636232 | 6.194361444 | -9.590020242 | 1.22E-19 | 5.75E-19 | 33.4042111 |
| CYP1A2 | -0.816292933 | 5.525305989 | -9.584081908 | 1.28E-19 | 6.02E-19 | 33.35838089 |
| AIF1 | -0.685053867 | 8.304287298 | -9.575828378 | 1.36E-19 | 6.41E-19 | 33.29471141 |
| TRPC1 | -0.745018316 | 6.629004896 | -9.572954359 | 1.39E-19 | 6.55E-19 | 33.27254846 |
| JOSD2 | 0.656970931 | 7.290262343 | 9.572379257 | 1.40E-19 | 6.57E-19 | 33.26811406 |
| TMEM52 | 0.700052691 | 5.542167971 | 9.572059623 | 1.40E-19 | 6.59E-19 | 33.26564955 |
| SAMD1 | 0.663411784 | 7.000105269 | 9.571658815 | 1.41E-19 | 6.61E-19 | 33.26255922 |
| DIO2 | 0.739576182 | 5.415938574 | 9.564157249 | 1.49E-19 | 6.99E-19 | 33.2047349 |
| GPR133 | -0.903960496 | 6.052386293 | -9.564103425 | 1.49E-19 | 6.99E-19 | 33.20432011 |
| PTGER2 | -0.896165501 | 6.397019918 | -9.561715976 | 1.52E-19 | 7.12E-19 | 33.18592279 |
| IL20RA | -0.914613602 | 6.12591741 | -9.560757527 | 1.53E-19 | 7.17E-19 | 33.17853792 |
| CLDN3 | 1.217146493 | 8.518169285 | 9.559522722 | 1.54E-19 | 7.23E-19 | 33.16902438 |
| UGDH | 0.86310994 | 9.656191513 | 9.556539971 | 1.58E-19 | 7.40E-19 | 33.14604692 |
| MSH6 | 0.587463338 | 8.750298551 | 9.552453802 | 1.63E-19 | 7.63E-19 | 33.11457644 |
| CTSO | -0.750421292 | 8.874114245 | -9.541409533 | 1.78E-19 | 8.30E-19 | 33.02955785 |
| CARD6 | -0.590722707 | 6.089458883 | -9.539575569 | 1.80E-19 | 8.42E-19 | 33.01544585 |
| NET1 | 0.772945678 | 8.647024521 | 9.538360268 | 1.82E-19 | 8.49E-19 | 33.00609525 |
| OLFM1 | -0.651178361 | 6.608139372 | -9.537755271 | 1.83E-19 | 8.53E-19 | 33.00144064 |
| PMM1 | -0.592553776 | 9.262615822 | -9.53750423 | 1.83E-19 | 8.54E-19 | 32.99950928 |
| S100A8 | -1.598467386 | 10.35053473 | -9.516914691 | 2.15E-19 | 1.00E-18 | 32.84121172 |
| SFTPB | -1.626028226 | 10.86105489 | -9.514039527 | 2.20E-19 | 1.02E-18 | 32.81912341 |
| CCDC68 | -0.868926402 | 6.176002737 | -9.503310479 | 2.39E-19 | 1.11E-18 | 32.73673416 |
| DYSF | -0.653969065 | 8.330124152 | -9.498301899 | 2.48E-19 | 1.15E-18 | 32.6982924 |
| ENTPD5 | 0.771212587 | 6.522740457 | 9.498016377 | 2.49E-19 | 1.16E-18 | 32.69610134 |
| SLC2A6 | -0.731201235 | 7.238296402 | -9.497831307 | 2.49E-19 | 1.16E-18 | 32.69468116 |
| ZMYND10 | -1.194921813 | 6.116642412 | -9.495823699 | 2.53E-19 | 1.17E-18 | 32.67927636 |
| ARSE | 1.291624069 | 6.157377827 | 9.492690658 | 2.60E-19 | 1.20E-18 | 32.65523988 |
| PAK1IP1 | 0.668209905 | 7.109833851 | 9.486306784 | 2.73E-19 | 1.26E-18 | 32.60627832 |
| MIA3 | 0.626024629 | 7.005499795 | 9.482365163 | 2.81E-19 | 1.30E-18 | 32.5760579 |
| FNDC4 | 0.857309913 | 6.669828452 | 9.47721535 | 2.93E-19 | 1.35E-18 | 32.53658591 |
| COL4A5 | -0.933893232 | 6.827746864 | -9.471102705 | 3.07E-19 | 1.41E-18 | 32.48975119 |
| NR4A2 | -1.369487428 | 8.414402109 | -9.46905213 | 3.12E-19 | 1.44E-18 | 32.47404398 |
| ADAMDEC1 | 1.517166634 | 5.976082423 | 9.456640802 | 3.43E-19 | 1.58E-18 | 32.37901912 |
| CTSF | -0.598144798 | 8.571241588 | -9.456129651 | 3.44E-19 | 1.58E-18 | 32.37510725 |
| GNLY | -0.949808504 | 6.928277279 | -9.452369653 | 3.55E-19 | 1.63E-18 | 32.3463357 |
| ZIC2 | 1.341317354 | 5.655739359 | 9.449987461 | 3.61E-19 | 1.65E-18 | 32.32811079 |
| CLCF1 | 0.745218662 | 7.524859154 | 9.449238449 | 3.63E-19 | 1.66E-18 | 32.32238108 |
| CSPG4 | -0.70815917 | 6.53283816 | -9.441134194 | 3.87E-19 | 1.77E-18 | 32.26040394 |
| VAMP5 | -0.690947654 | 10.16201431 | -9.430027363 | 4.21E-19 | 1.92E-18 | 32.17551806 |
| RAB28 | -0.595002519 | 6.923688271 | -9.425655219 | 4.36E-19 | 1.99E-18 | 32.14212013 |
| ACHE | 0.676063774 | 5.228212066 | 9.425157707 | 4.38E-19 | 1.99E-18 | 32.13832035 |
| RCOR2 | 0.87894393 | 5.719824763 | 9.4186211 | 4.60E-19 | 2.09E-18 | 32.08840805 |
| KDR | -0.896291575 | 7.251302439 | -9.41459735 | 4.75E-19 | 2.16E-18 | 32.05769413 |
| NAPG | 0.590692353 | 6.893863891 | 9.414396542 | 4.75E-19 | 2.16E-18 | 32.05616153 |
| ATP1B1 | 0.654006191 | 11.45724131 | 9.40920267 | 4.95E-19 | 2.25E-18 | 32.01652842 |
| PLA2G2D | 0.724016483 | 6.702474223 | 9.407048642 | 5.03E-19 | 2.28E-18 | 32.00009556 |
| LMNB1 | 0.824642976 | 6.312642154 | 9.394999916 | 5.52E-19 | 2.50E-18 | 31.90822012 |
| CCT5 | 0.653255301 | 8.196890928 | 9.391388705 | 5.68E-19 | 2.57E-18 | 31.8806977 |
| HEY2 | -0.787347963 | 6.358079093 | -9.385202938 | 5.95E-19 | 2.69E-18 | 31.83356888 |
| TSPAN6 | 0.674706638 | 8.644069673 | 9.384101218 | 6.00E-19 | 2.71E-18 | 31.825177 |
| MMP13 | 1.44000644 | 5.067807625 | 9.383532248 | 6.03E-19 | 2.72E-18 | 31.82084335 |
| SLC2A3 | -0.863315969 | 10.00899403 | -9.381481885 | 6.13E-19 | 2.76E-18 | 31.80522779 |
| LYSMD1 | 0.712033638 | 5.878656029 | 9.378246696 | 6.28E-19 | 2.83E-18 | 31.78059294 |
| MMP14 | 0.655720582 | 6.766596061 | 9.369096229 | 6.74E-19 | 3.04E-18 | 31.71094388 |
| HSD3B7 | 0.638583953 | 8.036622495 | 9.365151923 | 6.95E-19 | 3.13E-18 | 31.68093473 |
| NKD2 | -0.72006894 | 8.730080928 | -9.363374976 | 7.04E-19 | 3.17E-18 | 31.6674179 |
| GPR116 | -1.084075748 | 10.7968014 | -9.363028292 | 7.06E-19 | 3.17E-18 | 31.66478094 |
| DMRTA2 | 0.718866023 | 5.640937745 | 9.357016274 | 7.39E-19 | 3.32E-18 | 31.61906179 |
| ZNF521 | -0.953326298 | 6.435787593 | -9.35349802 | 7.60E-19 | 3.41E-18 | 31.59231527 |
| PRNP | -0.6163046 | 10.2399446 | -9.346502587 | 8.02E-19 | 3.59E-18 | 31.53915314 |
| DTNB | 0.647097434 | 6.849250769 | 9.345488918 | 8.08E-19 | 3.62E-18 | 31.53145177 |
| GPR110 | 1.288037701 | 5.840588771 | 9.344638173 | 8.13E-19 | 3.64E-18 | 31.52498863 |
| SACM1L | -0.635412859 | 9.021308684 | -9.343837813 | 8.18E-19 | 3.66E-18 | 31.51890859 |
| RFC4 | 0.810203154 | 7.894518096 | 9.340942096 | 8.37E-19 | 3.74E-18 | 31.49691363 |
| SOX13 | -0.61459368 | 7.636300782 | -9.333812539 | 8.84E-19 | 3.95E-18 | 31.44277789 |
| SLC9A3R1 | 0.604715571 | 9.301986468 | 9.331712962 | 8.98E-19 | 4.01E-18 | 31.42684043 |
| MPEG1 | -0.681218437 | 7.18999537 | -9.31998364 | 9.82E-19 | 4.37E-18 | 31.33784679 |
| PTK7 | 0.615869839 | 7.976877654 | 9.316094997 | 1.01E-18 | 4.50E-18 | 31.30835802 |
| HECW2 | -0.870687382 | 7.783769266 | -9.309847958 | 1.06E-18 | 4.72E-18 | 31.26100096 |
| TUFT1 | 0.689578241 | 8.631505609 | 9.308461982 | 1.07E-18 | 4.77E-18 | 31.25049696 |
| TMEM55A | -0.708603301 | 6.471294181 | -9.307085702 | 1.08E-18 | 4.82E-18 | 31.24006741 |
| MMP15 | 0.801927509 | 7.446744793 | 9.306980089 | 1.09E-18 | 4.82E-18 | 31.23926711 |
| CD1D | -0.62619569 | 6.22019487 | -9.305054736 | 1.10E-18 | 4.89E-18 | 31.22467837 |
| MTHFD2 | 0.660401527 | 8.292714141 | 9.301677185 | 1.13E-18 | 5.01E-18 | 31.19909065 |
| TYRP1 | -1.065338494 | 5.161050574 | -9.299098448 | 1.15E-18 | 5.10E-18 | 31.17955852 |
| LEPREL2 | 0.652852112 | 6.950840266 | 9.294687357 | 1.19E-18 | 5.27E-18 | 31.14615547 |
| EGR3 | -0.882871001 | 6.588829346 | -9.290628932 | 1.23E-18 | 5.43E-18 | 31.11543177 |
| CD247 | -0.703601185 | 7.92521763 | -9.283103521 | 1.30E-18 | 5.74E-18 | 31.05848405 |
| SSBP2 | -0.684928222 | 8.698217093 | -9.281691427 | 1.32E-18 | 5.80E-18 | 31.04780143 |
| PTN | -0.780187645 | 7.636464037 | -9.280662228 | 1.33E-18 | 5.84E-18 | 31.04001607 |
| CLDN4 | 0.748540143 | 7.206476614 | 9.27923426 | 1.34E-18 | 5.90E-18 | 31.02921514 |
| GPT | 0.666194254 | 5.841314747 | 9.274660179 | 1.39E-18 | 6.11E-18 | 30.99462453 |
| CCL21 | -1.046694952 | 9.014210753 | -9.27391329 | 1.40E-18 | 6.14E-18 | 30.98897734 |
| PTGS1 | -0.593537288 | 6.567938043 | -9.273479405 | 1.40E-18 | 6.16E-18 | 30.9856969 |
| GSTCD | 0.600901387 | 5.369795465 | 9.270264267 | 1.44E-18 | 6.31E-18 | 30.96139139 |
| RP2 | -0.819189422 | 7.069949154 | -9.264873946 | 1.50E-18 | 6.56E-18 | 30.92065404 |
| ADAMTS9 | -0.81820643 | 7.244238274 | -9.263083843 | 1.52E-18 | 6.65E-18 | 30.90712864 |
| WFDC10B | 0.792922852 | 5.181532527 | 9.262492982 | 1.53E-18 | 6.67E-18 | 30.90266465 |
| LYL1 | -0.606988105 | 8.557758771 | -9.262396188 | 1.53E-18 | 6.68E-18 | 30.90193339 |
| RGL1 | -0.605441079 | 9.712920247 | -9.258643931 | 1.57E-18 | 6.87E-18 | 30.87358929 |
| AVPI1 | -0.698239992 | 9.540360827 | -9.256267855 | 1.60E-18 | 6.99E-18 | 30.85564443 |
| OLFML3 | -0.812393694 | 8.857547202 | -9.25389407 | 1.63E-18 | 7.11E-18 | 30.83771977 |
| ABCA4 | 1.344181537 | 6.158280128 | 9.250029301 | 1.68E-18 | 7.31E-18 | 30.80854277 |
| CRISPLD2 | -0.781278519 | 9.380133152 | -9.247945501 | 1.70E-18 | 7.43E-18 | 30.79281435 |
| SPTBN5 | 0.586532443 | 5.524787912 | 9.246150524 | 1.73E-18 | 7.53E-18 | 30.77926774 |
| HLA-DPB1 | -0.910009527 | 10.06662701 | -9.244326157 | 1.75E-18 | 7.62E-18 | 30.76550103 |
| DPY19L1 | 0.757165249 | 9.012494293 | 9.242824567 | 1.77E-18 | 7.70E-18 | 30.75417128 |
| CYP39A1 | -0.701964779 | 5.219793449 | -9.241753275 | 1.79E-18 | 7.77E-18 | 30.74608891 |
| SPAG6 | -1.36751343 | 5.754318444 | -9.233184318 | 1.91E-18 | 8.27E-18 | 30.68146166 |
| TAF13 | 0.734180929 | 7.057998989 | 9.219932554 | 2.11E-18 | 9.14E-18 | 30.58159116 |
| HIP1 | -0.786791402 | 7.669968364 | -9.215213626 | 2.19E-18 | 9.46E-18 | 30.54604936 |
| HECA | -0.682642745 | 7.644240234 | -9.210428805 | 2.27E-18 | 9.81E-18 | 30.51002303 |
| ZFP3 | -0.588061242 | 6.805201963 | -9.210036503 | 2.27E-18 | 9.84E-18 | 30.50706979 |
| ITGA4 | -0.620516979 | 6.168170016 | -9.209121489 | 2.29E-18 | 9.90E-18 | 30.50018191 |
| PRAME | 1.100095466 | 5.487338705 | 9.204515835 | 2.37E-18 | 1.02E-17 | 30.46551889 |
| BCORL1 | 0.606741326 | 6.733934862 | 9.202410811 | 2.41E-18 | 1.04E-17 | 30.44967973 |
| SEMA6D | -0.59803936 | 5.165269572 | -9.200270148 | 2.45E-18 | 1.06E-17 | 30.43357477 |
| RRAD | -1.1078849 | 7.458272362 | -9.192379814 | 2.60E-18 | 1.12E-17 | 30.3742335 |
| CLDN10 | 1.466007463 | 6.106623553 | 9.187040935 | 2.71E-18 | 1.17E-17 | 30.33409942 |
| CLIC2 | -0.867631955 | 7.644378684 | -9.176161588 | 2.94E-18 | 1.27E-17 | 30.25236166 |
| HN1 | 0.765691317 | 8.321578468 | 9.173165485 | 3.01E-18 | 1.29E-17 | 30.2298624 |
| TNFRSF18 | 0.786987495 | 5.955464396 | 9.171413565 | 3.05E-18 | 1.31E-17 | 30.21670851 |
| SCGB3A2 | -2.072730026 | 10.25726743 | -9.169502478 | 3.10E-18 | 1.33E-17 | 30.20236136 |
| PRR11 | 0.866968894 | 6.728013277 | 9.166592107 | 3.16E-18 | 1.36E-17 | 30.18051592 |
| FGD6 | 0.742903229 | 6.65869509 | 9.165892936 | 3.18E-18 | 1.36E-17 | 30.17526856 |
| ITGAM | -0.907582436 | 8.237169848 | -9.165822455 | 3.18E-18 | 1.36E-17 | 30.1747396 |
| DDIT4L | 1.201246768 | 6.572658138 | 9.162633263 | 3.26E-18 | 1.40E-17 | 30.15080766 |
| SEC14L2 | 0.652854099 | 5.348008069 | 9.162242063 | 3.27E-18 | 1.40E-17 | 30.14787243 |
| TGFB1I1 | -0.586706221 | 7.866118771 | -9.159221633 | 3.35E-18 | 1.43E-17 | 30.12521239 |
| MFI2 | 0.673015784 | 5.541996681 | 9.158478638 | 3.37E-18 | 1.44E-17 | 30.11963898 |
| CACHD1 | -1.110059197 | 7.369222811 | -9.154326266 | 3.47E-18 | 1.48E-17 | 30.08849619 |
| PDK1 | 0.702189132 | 6.652183101 | 9.149279694 | 3.61E-18 | 1.54E-17 | 30.05065901 |
| MDH1B | -0.778674625 | 4.855909995 | -9.148467533 | 3.63E-18 | 1.55E-17 | 30.04457099 |
| GALNT14 | 0.913418521 | 6.54452517 | 9.147378127 | 3.66E-18 | 1.56E-17 | 30.03640526 |
| C1QC | -0.865333102 | 10.82824744 | -9.14565227 | 3.71E-18 | 1.58E-17 | 30.02347024 |
| FAS | -0.724047982 | 6.69647784 | -9.137150245 | 3.96E-18 | 1.68E-17 | 29.95977162 |
| PCYOX1 | -0.61929198 | 9.754811606 | -9.1286209 | 4.22E-18 | 1.79E-17 | 29.89590629 |
| DNAJB1 | -0.588034155 | 9.701170912 | -9.118430526 | 4.56E-18 | 1.93E-17 | 29.81965356 |
| CCDC60 | -0.870743369 | 5.617913082 | -9.098006695 | 5.32E-18 | 2.24E-17 | 29.66698967 |
| CSTA | -1.044580093 | 6.385520566 | -9.09362322 | 5.50E-18 | 2.32E-17 | 29.63425267 |
| CLEC2B | -0.809992235 | 6.781947923 | -9.092842931 | 5.53E-18 | 2.33E-17 | 29.62842631 |
| LRRC46 | -0.99746959 | 5.898846253 | -9.085316941 | 5.85E-18 | 2.46E-17 | 29.5722468 |
| KLHL9 | -0.623383682 | 8.75882416 | -9.069011285 | 6.62E-18 | 2.78E-17 | 29.45063179 |
| PPARGC1B | -0.766645344 | 6.341008253 | -9.066054097 | 6.77E-18 | 2.84E-17 | 29.42859075 |
| SPRY2 | -0.710529247 | 8.714847314 | -9.060690873 | 7.05E-18 | 2.95E-17 | 29.38862842 |
| PDCL | -0.651279363 | 6.704529774 | -9.059470608 | 7.11E-18 | 2.98E-17 | 29.37953812 |
| CYP4V2 | -0.726192263 | 8.2810705 | -9.05626964 | 7.29E-18 | 3.05E-17 | 29.35569645 |
| GPR126 | -1.04749693 | 7.625825949 | -9.049675109 | 7.66E-18 | 3.20E-17 | 29.30659568 |
| C6orf165 | -0.728040656 | 5.220667521 | -9.040896619 | 8.18E-18 | 3.42E-17 | 29.24126956 |
| PGF | 0.686783831 | 6.373442535 | 9.040082743 | 8.23E-18 | 3.44E-17 | 29.23521508 |
| TBC1D10C | -0.653518351 | 8.075295888 | -9.034623251 | 8.58E-18 | 3.58E-17 | 29.1946106 |
| DYNC2H1 | -0.619675211 | 6.322374963 | -9.030788774 | 8.83E-18 | 3.68E-17 | 29.16610148 |
| DMBT1 | -1.39312051 | 8.263059218 | -9.027443276 | 9.05E-18 | 3.77E-17 | 29.14123426 |
| HCST | -0.685601549 | 8.638683178 | -9.020427326 | 9.54E-18 | 3.98E-17 | 29.08910373 |
| C1GALT1 | 0.860391322 | 6.858884218 | 9.016218656 | 9.85E-18 | 4.10E-17 | 29.05784465 |
| HLA-DRA | -0.655417171 | 13.04155889 | -9.014230008 | 1.00E-17 | 4.16E-17 | 29.04307763 |
| LGI2 | 0.691480806 | 5.945067771 | 9.008390763 | 1.04E-17 | 4.34E-17 | 28.99972954 |
| FCHO1 | 0.629413663 | 6.375935545 | 9.00240709 | 1.09E-17 | 4.53E-17 | 28.95532812 |
| COCH | 0.866769255 | 5.515247165 | 9.001637078 | 1.10E-17 | 4.56E-17 | 28.94961568 |
| RAPGEF3 | -0.708459519 | 6.509290668 | -8.997085047 | 1.14E-17 | 4.71E-17 | 28.9158523 |
| C10orf10 | -0.793146799 | 9.284684133 | -8.993044211 | 1.17E-17 | 4.85E-17 | 28.88588982 |
| FAT4 | -0.703307498 | 5.858181997 | -8.982117992 | 1.27E-17 | 5.25E-17 | 28.80491639 |
| FYN | -0.601044178 | 8.152837418 | -8.979182133 | 1.30E-17 | 5.36E-17 | 28.78316981 |
| ITM2C | 0.592658034 | 9.806831851 | 8.978639411 | 1.31E-17 | 5.38E-17 | 28.77915025 |
| C9orf117 | -0.661711312 | 5.535352992 | -8.972083987 | 1.37E-17 | 5.64E-17 | 28.73061126 |
| HPGD | -1.71628592 | 9.037959074 | -8.971128058 | 1.38E-17 | 5.68E-17 | 28.7235351 |
| ARL9 | 0.796163167 | 4.936203471 | 8.970216203 | 1.39E-17 | 5.72E-17 | 28.71678565 |
| MAMDC4 | 0.636945514 | 6.173636234 | 8.969596369 | 1.40E-17 | 5.74E-17 | 28.71219796 |
| GPX2 | 2.227971708 | 7.002556971 | 8.969297688 | 1.40E-17 | 5.75E-17 | 28.70998735 |
| COL1A2 | 0.891807063 | 10.99877139 | 8.969061662 | 1.40E-17 | 5.76E-17 | 28.7082405 |
| SLC40A1 | -0.761464926 | 9.24602604 | -8.960494768 | 1.50E-17 | 6.14E-17 | 28.64485631 |
| RHOB | -0.767059821 | 9.552118303 | -8.954740629 | 1.56E-17 | 6.39E-17 | 28.60230503 |
| AGR2 | 1.341762781 | 8.709309875 | 8.95400567 | 1.57E-17 | 6.43E-17 | 28.59687136 |
| C1orf168 | -0.939195937 | 4.667834309 | -8.953129684 | 1.58E-17 | 6.47E-17 | 28.59039543 |
| AP1M2 | 0.664709503 | 7.093120548 | 8.952170706 | 1.59E-17 | 6.51E-17 | 28.58330645 |
| RMI1 | 0.621860744 | 7.018443037 | 8.95137479 | 1.60E-17 | 6.55E-17 | 28.57742322 |
| CD86 | -0.630604482 | 6.960194255 | -8.933610934 | 1.83E-17 | 7.47E-17 | 28.44620549 |
| MUC16 | 1.731448592 | 6.679609348 | 8.922317745 | 1.99E-17 | 8.11E-17 | 28.36287339 |
| LILRB5 | -0.706427566 | 6.248640285 | -8.920969885 | 2.01E-17 | 8.19E-17 | 28.35293215 |
| SEC14L4 | -0.870067101 | 6.411262455 | -8.915756058 | 2.09E-17 | 8.51E-17 | 28.31448644 |
| UNC13B | -0.783176136 | 8.594542503 | -8.912389181 | 2.14E-17 | 8.71E-17 | 28.28966756 |
| RHOV | 0.821336267 | 5.891541489 | 8.903590066 | 2.29E-17 | 9.29E-17 | 28.22483389 |
| ISG15 | 0.813094697 | 9.314882261 | 8.902661413 | 2.30E-17 | 9.35E-17 | 28.21799383 |
| HAPLN3 | 0.719745748 | 6.836400207 | 8.898123531 | 2.38E-17 | 9.66E-17 | 28.18457641 |
| CD44 | -0.612089221 | 9.443510205 | -8.894495667 | 2.45E-17 | 9.92E-17 | 28.15786847 |
| CCDC17 | -0.985942798 | 6.00967808 | -8.890707242 | 2.52E-17 | 1.02E-16 | 28.12998608 |
| CCR1 | -0.802188416 | 6.851110266 | -8.878098168 | 2.77E-17 | 1.12E-16 | 28.03724067 |
| C3 | -1.012509672 | 10.05559821 | -8.86790126 | 2.98E-17 | 1.20E-16 | 27.96230083 |
| COBL | -0.976230086 | 8.046943742 | -8.862711446 | 3.10E-17 | 1.25E-16 | 27.92418115 |
| MYBL2 | 0.615192477 | 5.861544582 | 8.849385335 | 3.42E-17 | 1.37E-16 | 27.82636662 |
| SCRN1 | 0.659500791 | 9.274510242 | 8.836979497 | 3.75E-17 | 1.50E-16 | 27.73539381 |
| CILP2 | 0.655345052 | 5.383778064 | 8.833882818 | 3.84E-17 | 1.54E-16 | 27.71269874 |
| SATB2 | 0.821698442 | 5.827549051 | 8.827833706 | 4.02E-17 | 1.61E-16 | 27.66838085 |
| PDLIM4 | 0.598307499 | 5.53391684 | 8.825727456 | 4.08E-17 | 1.63E-16 | 27.65295441 |
| AGA | 0.61250603 | 8.389211718 | 8.817871904 | 4.32E-17 | 1.73E-16 | 27.59544075 |
| LCN2 | 1.429639838 | 9.845013144 | 8.8136676 | 4.46E-17 | 1.78E-16 | 27.5646732 |
| UMODL1 | 0.734101888 | 4.43639838 | 8.807981612 | 4.65E-17 | 1.86E-16 | 27.5230779 |
| SLC7A11 | 1.08077769 | 5.527317447 | 8.807151827 | 4.68E-17 | 1.87E-16 | 27.51700916 |
| ETV7 | 0.61638176 | 5.547130617 | 8.806859214 | 4.69E-17 | 1.87E-16 | 27.5148692 |
| PKIB | 0.796946404 | 5.334817899 | 8.804565053 | 4.77E-17 | 1.90E-16 | 27.4980929 |
| HHAT | 0.677808667 | 6.102313441 | 8.802212656 | 4.85E-17 | 1.93E-16 | 27.48089374 |
| TRIM59 | 0.598109728 | 5.221985266 | 8.796447122 | 5.07E-17 | 2.01E-16 | 27.43875281 |
| MID1IP1 | -0.659795933 | 8.979352428 | -8.793349424 | 5.18E-17 | 2.06E-16 | 27.41611892 |
| PRKCDBP | -0.703975814 | 9.396622178 | -8.785307847 | 5.50E-17 | 2.18E-16 | 27.35738623 |
| ELL3 | 0.651453886 | 6.032279678 | 8.777028984 | 5.85E-17 | 2.32E-16 | 27.29695758 |
| ENO2 | 0.727988757 | 8.522953577 | 8.77580415 | 5.90E-17 | 2.34E-16 | 27.28802053 |
| FREM2 | -0.972863785 | 5.845160521 | -8.774296898 | 5.97E-17 | 2.36E-16 | 27.27702394 |
| CYTL1 | -1.033921041 | 5.856900819 | -8.760824445 | 6.59E-17 | 2.60E-16 | 27.17878729 |
| SUV39H2 | 0.635454524 | 6.472868484 | 8.739261859 | 7.73E-17 | 3.04E-16 | 27.02176819 |
| RND3 | -0.814765613 | 8.899419572 | -8.737800034 | 7.81E-17 | 3.08E-16 | 27.01113244 |
| C4BPA | -1.773671584 | 10.24188835 | -8.732402882 | 8.13E-17 | 3.20E-16 | 26.97187481 |
| HS3ST1 | 0.902715729 | 6.91960771 | 8.726455755 | 8.49E-17 | 3.33E-16 | 26.92863544 |
| ARC | -0.709220839 | 6.030729524 | -8.720885625 | 8.84E-17 | 3.47E-16 | 26.88815482 |
| SPARC | -0.686351819 | 11.73982336 | -8.714789161 | 9.25E-17 | 3.62E-16 | 26.84386879 |
| SLC25A15 | 0.633312063 | 6.554163644 | 8.713466118 | 9.34E-17 | 3.66E-16 | 26.83426064 |
| PLXDC2 | -0.613329969 | 9.126960354 | -8.712985493 | 9.37E-17 | 3.67E-16 | 26.8307705 |
| TRPC6 | -0.920285046 | 6.925260614 | -8.708615611 | 9.68E-17 | 3.79E-16 | 26.79904378 |
| EDNRA | -0.799291855 | 8.916893388 | -8.705369946 | 9.91E-17 | 3.88E-16 | 26.77548608 |
| LCN6 | -0.725630943 | 5.481039351 | -8.700638468 | 1.03E-16 | 4.01E-16 | 26.74115452 |
| WNT10A | 0.723701675 | 6.06754141 | 8.698135108 | 1.05E-16 | 4.09E-16 | 26.72299519 |
| KRT15 | 1.579548448 | 7.096976367 | 8.694085926 | 1.08E-16 | 4.21E-16 | 26.69362987 |
| TMEM35 | -0.756626867 | 5.24077417 | -8.679537112 | 1.20E-16 | 4.67E-16 | 26.58819473 |
| AKR1B10 | 2.110614755 | 5.625096114 | 8.664661128 | 1.34E-16 | 5.20E-16 | 26.48051045 |
| ADAM12 | 0.74281244 | 5.524827032 | 8.658572079 | 1.40E-16 | 5.43E-16 | 26.43646863 |
| GPR87 | 1.198008398 | 5.220938338 | 8.655825157 | 1.43E-16 | 5.54E-16 | 26.41660705 |
| NAP1L3 | -0.652911169 | 4.956085093 | -8.652011307 | 1.47E-16 | 5.69E-16 | 26.38903805 |
| CCL2 | -1.064192376 | 10.33677249 | -8.647309067 | 1.52E-16 | 5.88E-16 | 26.35505836 |
| DCLRE1C | 0.593087886 | 7.235656399 | 8.64152444 | 1.58E-16 | 6.13E-16 | 26.31327402 |
| HYDIN | -0.619911023 | 4.967033811 | -8.637837681 | 1.63E-16 | 6.29E-16 | 26.28665307 |
| TNFRSF10A | 0.636693166 | 7.056439907 | 8.62558338 | 1.78E-16 | 6.87E-16 | 26.19822324 |
| AKR7A3 | 0.609002198 | 7.305052875 | 8.624546453 | 1.79E-16 | 6.92E-16 | 26.1907444 |
| TBXAS1 | -0.633025095 | 8.191035215 | -8.623302782 | 1.81E-16 | 6.98E-16 | 26.18177522 |
| VPREB3 | 0.903972054 | 5.911269162 | 8.615126645 | 1.92E-16 | 7.39E-16 | 26.12283165 |
| FOLR1 | -1.103350889 | 8.461984625 | -8.609045456 | 2.01E-16 | 7.72E-16 | 26.07901537 |
| CD1C | -0.868290682 | 6.790308979 | -8.592194336 | 2.27E-16 | 8.71E-16 | 25.95770806 |
| SNCAIP | -0.791506999 | 6.411701476 | -8.588785484 | 2.33E-16 | 8.93E-16 | 25.93318796 |
| NUCKS1 | 0.648444811 | 8.142237231 | 8.588296176 | 2.33E-16 | 8.95E-16 | 25.92966888 |
| FCGBP | 0.942125367 | 6.884552282 | 8.587348279 | 2.35E-16 | 9.01E-16 | 25.92285202 |
| TMPRSS13 | 0.706834289 | 6.144632601 | 8.573931951 | 2.59E-16 | 9.90E-16 | 25.82642201 |
| GPR115 | 0.626449919 | 4.130487846 | 8.561328007 | 2.84E-16 | 1.08E-15 | 25.73592363 |
| GLIPR1 | -0.900717215 | 8.165110356 | -8.560437213 | 2.86E-16 | 1.09E-15 | 25.72953098 |
| CX3CL1 | -0.937196346 | 9.063917718 | -8.553140214 | 3.01E-16 | 1.15E-15 | 25.67718207 |
| FKBP10 | 0.724755761 | 6.487516077 | 8.541667758 | 3.28E-16 | 1.24E-15 | 25.59493927 |
| CDKN2A | 0.977960163 | 5.948775051 | 8.539110535 | 3.34E-16 | 1.26E-15 | 25.57661744 |
| BATF | 0.667903472 | 7.633236503 | 8.534918155 | 3.44E-16 | 1.30E-15 | 25.54658815 |
| ALOX15 | -0.654441111 | 5.172127372 | -8.534714802 | 3.45E-16 | 1.30E-15 | 25.54513182 |
| PTPN13 | -0.919471964 | 7.899707383 | -8.528115611 | 3.61E-16 | 1.36E-15 | 25.49788394 |
| ZNRF3 | -0.623776364 | 7.212436093 | -8.526270948 | 3.66E-16 | 1.38E-15 | 25.48468123 |
| SLC15A2 | -0.938302267 | 7.117272814 | -8.525456617 | 3.68E-16 | 1.39E-15 | 25.47885347 |
| SLC28A3 | 0.736824981 | 5.129683191 | 8.524481386 | 3.71E-16 | 1.40E-15 | 25.47187473 |
| IDI1 | -0.642334889 | 7.911812122 | -8.522278927 | 3.77E-16 | 1.42E-15 | 25.45611596 |
| PMAIP1 | 0.953908121 | 6.305613463 | 8.50223541 | 4.36E-16 | 1.64E-15 | 25.31282981 |
| CFTR | -1.153670601 | 6.585062782 | -8.501553208 | 4.38E-16 | 1.65E-15 | 25.30795695 |
| CPM | -0.69727426 | 7.321935213 | -8.497415672 | 4.51E-16 | 1.70E-15 | 25.27840883 |
| HGD | 1.003315511 | 5.477276731 | 8.472879889 | 5.39E-16 | 2.02E-15 | 25.10338797 |
| CHST6 | 0.958974369 | 5.756624721 | 8.471494789 | 5.44E-16 | 2.04E-15 | 25.09351792 |
| PARVG | -0.656653626 | 8.345718777 | -8.470574737 | 5.48E-16 | 2.05E-15 | 25.08696235 |
| TIPARP | -0.690855901 | 9.974372737 | -8.467909663 | 5.59E-16 | 2.09E-15 | 25.06797584 |
| IFIT5 | -0.5931768 | 7.303424311 | -8.467546606 | 5.60E-16 | 2.09E-15 | 25.06538966 |
| CCDC15 | 0.642702802 | 5.379596426 | 8.467388825 | 5.61E-16 | 2.10E-15 | 25.06426576 |
| JPH1 | -0.893009114 | 7.025235689 | -8.457827812 | 6.01E-16 | 2.24E-15 | 24.99618758 |
| CD47 | -0.633302417 | 9.287239237 | -8.452200445 | 6.26E-16 | 2.33E-15 | 24.956143 |
| PIGX | 0.830085235 | 7.380051713 | 8.436651909 | 7.00E-16 | 2.60E-15 | 24.8455935 |
| FNDC1 | 1.276665407 | 7.287138356 | 8.425698995 | 7.57E-16 | 2.81E-15 | 24.76780212 |
| MAP3K13 | 0.760175825 | 7.199500822 | 8.421863164 | 7.78E-16 | 2.89E-15 | 24.74057506 |
| PLEKHA6 | 0.698554364 | 7.059248441 | 8.420966107 | 7.83E-16 | 2.90E-15 | 24.73420889 |
| ZDHHC2 | -0.628749784 | 7.217632835 | -8.413714542 | 8.25E-16 | 3.05E-15 | 24.68276355 |
| LPPR4 | -0.745478572 | 5.41687091 | -8.408415833 | 8.57E-16 | 3.16E-15 | 24.64519169 |
| HLA-DPA1 | -0.752551403 | 12.36212664 | -8.404337886 | 8.83E-16 | 3.25E-15 | 24.61628698 |
| KRT6A | 1.393561755 | 6.11503037 | 8.388614658 | 9.88E-16 | 3.64E-15 | 24.50492985 |
| APOBEC3A | -0.663335971 | 5.458966141 | -8.371135379 | 1.12E-15 | 4.11E-15 | 24.38130368 |
| FILIP1L | -0.709739879 | 8.17990366 | -8.369460528 | 1.13E-15 | 4.16E-15 | 24.36946722 |
| CDK6 | -0.719220355 | 7.526706883 | -8.3614796 | 1.20E-15 | 4.40E-15 | 24.31308694 |
| PPP2R2C | 0.811558136 | 5.069246548 | 8.354773902 | 1.26E-15 | 4.60E-15 | 24.26574396 |
| CLEC4A | -0.805466739 | 6.959660668 | -8.353052724 | 1.27E-15 | 4.66E-15 | 24.25359646 |
| CHIA | -0.935579437 | 5.605382899 | -8.33631555 | 1.44E-15 | 5.24E-15 | 24.13556096 |
| RNASE1 | -0.861315723 | 11.46666026 | -8.333961516 | 1.46E-15 | 5.33E-15 | 24.1189727 |
| CKAP2 | 0.675900348 | 6.819816952 | 8.329958741 | 1.50E-15 | 5.48E-15 | 24.0907736 |
| HOXC13 | 0.704934342 | 5.271627702 | 8.329733042 | 1.51E-15 | 5.49E-15 | 24.08918386 |
| PCSK9 | -0.831998617 | 6.430885593 | -8.322333851 | 1.59E-15 | 5.78E-15 | 24.03708296 |
| MBTD1 | 1.004349789 | 7.93503971 | 8.320833321 | 1.60E-15 | 5.84E-15 | 24.02652098 |
| DHCR24 | -0.629737298 | 10.52740128 | -8.317771956 | 1.64E-15 | 5.96E-15 | 24.00497661 |
| PERP | 0.853799721 | 8.786499484 | 8.311543463 | 1.71E-15 | 6.23E-15 | 23.96116047 |
| JSRP1 | 1.010131105 | 7.361780287 | 8.308903825 | 1.75E-15 | 6.34E-15 | 23.942598 |
| AGTR2 | -0.778460364 | 4.869599263 | -8.307878875 | 1.76E-15 | 6.38E-15 | 23.93539145 |
| EGF | 0.684071176 | 5.106350093 | 8.305147557 | 1.79E-15 | 6.50E-15 | 23.9161902 |
| FAM111B | 0.662883278 | 5.054505971 | 8.305100453 | 1.79E-15 | 6.50E-15 | 23.9158591 |
| HDGFRP3 | -0.817112195 | 7.24529437 | -8.292334181 | 1.97E-15 | 7.12E-15 | 23.82617004 |
| OMD | -1.096780179 | 5.67983558 | -8.290427179 | 1.99E-15 | 7.21E-15 | 23.81278065 |
| TAGLN | -0.594130956 | 9.004437226 | -8.277324715 | 2.19E-15 | 7.89E-15 | 23.72084363 |
| RAB3B | 0.643658796 | 4.861603593 | 8.26874762 | 2.32E-15 | 8.37E-15 | 23.66071465 |
| RSPO3 | -0.845110439 | 7.057321346 | -8.266446068 | 2.36E-15 | 8.51E-15 | 23.64458718 |
| DNM3 | -0.787971477 | 5.812330912 | -8.265716642 | 2.37E-15 | 8.55E-15 | 23.63947658 |
| IL1R1 | -0.715514828 | 8.661727723 | -8.265575156 | 2.38E-15 | 8.56E-15 | 23.63848532 |
| PLEK | -0.817198497 | 8.704850481 | -8.265455013 | 2.38E-15 | 8.56E-15 | 23.6376436 |
| NCF4 | -0.605727249 | 7.424809069 | -8.262549066 | 2.43E-15 | 8.73E-15 | 23.61728714 |
| SH3PXD2B | 0.648281867 | 7.997604165 | 8.253476984 | 2.59E-15 | 9.30E-15 | 23.55376822 |
| RELN | -0.792379392 | 5.555710992 | -8.24958707 | 2.66E-15 | 9.56E-15 | 23.5265475 |
| MSX1 | -0.786665742 | 6.802395681 | -8.235894228 | 2.93E-15 | 1.05E-14 | 23.43079913 |
| CHST7 | -0.598612845 | 7.805940213 | -8.226684143 | 3.13E-15 | 1.12E-14 | 23.36645904 |
| SLCO4C1 | -1.007881818 | 6.058234059 | -8.226539948 | 3.14E-15 | 1.12E-14 | 23.36545212 |
| BLZF1 | 0.868887112 | 9.068100149 | 8.221146881 | 3.26E-15 | 1.16E-14 | 23.32780078 |
| HSH2D | 0.634806165 | 6.776179939 | 8.218524461 | 3.32E-15 | 1.18E-14 | 23.30949875 |
| FAM3C | 0.649619975 | 8.685328359 | 8.211176313 | 3.49E-15 | 1.25E-14 | 23.25823722 |
| PDCD1LG2 | -0.59596551 | 5.384168965 | -8.209136269 | 3.55E-15 | 1.26E-14 | 23.24401131 |
| PPAPDC1A | 0.732758495 | 5.304848117 | 8.206803487 | 3.60E-15 | 1.28E-14 | 23.22774706 |
| CMKLR1 | -0.620549363 | 5.983877944 | -8.204879471 | 3.65E-15 | 1.30E-14 | 23.21433516 |
| TNIK | -0.666498102 | 6.223873146 | -8.203611648 | 3.69E-15 | 1.31E-14 | 23.20549864 |
| ICAM4 | -0.898643513 | 6.328077181 | -8.201827151 | 3.73E-15 | 1.33E-14 | 23.1930626 |
| SLC23A3 | 0.610823325 | 5.497462992 | 8.197234295 | 3.86E-15 | 1.37E-14 | 23.16106398 |
| CIB2 | 0.639175475 | 5.792712428 | 8.196726428 | 3.87E-15 | 1.37E-14 | 23.15752642 |
| CTSH | -0.778777652 | 11.53911517 | -8.191699795 | 4.01E-15 | 1.42E-14 | 23.1225215 |
| MT2A | -0.753242528 | 12.36461845 | -8.187165666 | 4.14E-15 | 1.47E-14 | 23.09095919 |
| C9orf9 | -0.690252866 | 6.741499931 | -8.176604121 | 4.46E-15 | 1.58E-14 | 23.01748707 |
| ELF3 | 0.778239712 | 9.525742202 | 8.168059346 | 4.74E-15 | 1.67E-14 | 22.95809326 |
| PALMD | -0.607731316 | 5.770201436 | -8.165579782 | 4.82E-15 | 1.70E-14 | 22.94086621 |
| SLC39A14 | 0.62827322 | 7.961250819 | 8.164878933 | 4.85E-15 | 1.71E-14 | 22.93599766 |
| SPP2 | 0.646821287 | 4.530836662 | 8.164156367 | 4.87E-15 | 1.71E-14 | 22.93097853 |
| CHMP4C | 0.734220394 | 7.281511705 | 8.16408228 | 4.87E-15 | 1.71E-14 | 22.93046393 |
| NAPSB | -0.872704345 | 7.891176096 | -8.160947037 | 4.98E-15 | 1.75E-14 | 22.90868951 |
| CD55 | -0.726048381 | 11.26222142 | -8.160470463 | 5.00E-15 | 1.76E-14 | 22.90538019 |
| IL6R | -0.630619113 | 6.79410104 | -8.155572174 | 5.17E-15 | 1.81E-14 | 22.87137446 |
| SEC24A | 0.64358183 | 7.268294987 | 8.14914897 | 5.41E-15 | 1.90E-14 | 22.82680387 |
| KIT | -1.04150479 | 8.646464747 | -8.146176756 | 5.53E-15 | 1.93E-14 | 22.80618801 |
| TNNT1 | 1.086015892 | 6.187755992 | 8.143466565 | 5.63E-15 | 1.97E-14 | 22.78739419 |
| ACSL1 | -0.664966276 | 9.336125332 | -8.140752258 | 5.74E-15 | 2.01E-14 | 22.76857622 |
| COL6A3 | 0.667034109 | 11.11480438 | 8.135070359 | 5.98E-15 | 2.09E-14 | 22.72919855 |
| FBXO2 | 0.780329797 | 7.35872287 | 8.133550837 | 6.04E-15 | 2.11E-14 | 22.71867097 |
| SLC35F3 | 0.606124676 | 5.143801809 | 8.133186048 | 6.06E-15 | 2.11E-14 | 22.71614383 |
| PDE4D | -0.959507877 | 7.273620072 | -8.131461385 | 6.13E-15 | 2.14E-14 | 22.70419703 |
| GNG4 | 1.089856531 | 5.141857069 | 8.128781796 | 6.25E-15 | 2.17E-14 | 22.68563896 |
| CTSS | -0.692535811 | 8.958276441 | -8.126509695 | 6.35E-15 | 2.21E-14 | 22.6699064 |
| GATA5 | -0.709739422 | 5.812350519 | -8.122970747 | 6.51E-15 | 2.26E-14 | 22.64540804 |
| ITGA2 | 0.927430165 | 8.645672936 | 8.121193563 | 6.59E-15 | 2.29E-14 | 22.63310832 |
| STAT1 | 0.6223822 | 10.17638251 | 8.120328742 | 6.63E-15 | 2.30E-14 | 22.62712366 |
| DNAH7 | -0.706594951 | 5.389688649 | -8.117136344 | 6.78E-15 | 2.35E-14 | 22.60503577 |
| ADORA2B | 0.75007731 | 7.520280463 | 8.116368845 | 6.81E-15 | 2.37E-14 | 22.59972643 |
| FOXA1 | 0.782365908 | 8.314587106 | 8.107399643 | 7.26E-15 | 2.52E-14 | 22.53770621 |
| GCLM | 0.745093265 | 7.275251918 | 8.100533346 | 7.61E-15 | 2.64E-14 | 22.49025975 |
| HORMAD1 | 1.109591282 | 4.834888944 | 8.0998167 | 7.65E-15 | 2.65E-14 | 22.48530933 |
| MYBPH | 0.959920921 | 5.662287455 | 8.090020802 | 8.20E-15 | 2.84E-14 | 22.41767239 |
| RCN3 | 0.634407268 | 7.51460766 | 8.070852187 | 9.37E-15 | 3.24E-14 | 22.28548733 |
| TLR7 | -0.817242043 | 6.346894226 | -8.067156826 | 9.62E-15 | 3.32E-14 | 22.26002988 |
| C3orf58 | -0.614948578 | 7.996534713 | -8.06699279 | 9.63E-15 | 3.32E-14 | 22.25890002 |
| PRSS3 | 1.033403065 | 5.728671027 | 8.066070036 | 9.69E-15 | 3.34E-14 | 22.25254453 |
| PDGFRA | -0.646262456 | 8.405210753 | -8.062720846 | 9.92E-15 | 3.42E-14 | 22.22948116 |
| PCSK1N | 1.102014789 | 7.326173359 | 8.052370012 | 1.07E-14 | 3.67E-14 | 22.1582455 |
| ZNF215 | 0.645664086 | 5.322751279 | 8.047608863 | 1.10E-14 | 3.79E-14 | 22.12550043 |
| SUSD4 | 0.692671955 | 5.572239237 | 8.047511012 | 1.10E-14 | 3.80E-14 | 22.1248276 |
| ST3GAL6 | -0.672611167 | 7.013775295 | -8.039678375 | 1.16E-14 | 4.00E-14 | 22.07098847 |
| PIM2 | 0.736318693 | 8.909861117 | 8.037972628 | 1.18E-14 | 4.05E-14 | 22.05926861 |
| MMP3 | 1.0284569 | 5.489798481 | 8.037677617 | 1.18E-14 | 4.05E-14 | 22.05724182 |
| NFIX | -0.671908909 | 9.209536051 | -8.01550289 | 1.38E-14 | 4.72E-14 | 21.90504778 |
| TMEM71 | -0.85808726 | 6.536248279 | -8.014696805 | 1.39E-14 | 4.74E-14 | 21.89952092 |
| C6orf141 | 0.923829618 | 5.668435641 | 8.014105499 | 1.39E-14 | 4.76E-14 | 21.89546691 |
| CDH6 | -0.628330752 | 5.701833293 | -8.0057977 | 1.47E-14 | 5.04E-14 | 21.83853097 |
| FBXO15 | -0.82615489 | 6.050373069 | -8.005459391 | 1.48E-14 | 5.05E-14 | 21.83621332 |
| MS4A6A | -0.778637565 | 9.108473269 | -8.003740221 | 1.50E-14 | 5.11E-14 | 21.8244369 |
| CCDC11 | -0.809426099 | 5.060065856 | -8.000113136 | 1.53E-14 | 5.23E-14 | 21.79959703 |
| SLC22A3 | -0.846268833 | 6.591747237 | -7.999821149 | 1.54E-14 | 5.24E-14 | 21.79759773 |
| KRT19 | 0.853599769 | 11.46026103 | 7.99681111 | 1.57E-14 | 5.35E-14 | 21.77699026 |
| KIF1A | 1.307838135 | 5.519639253 | 7.98355987 | 1.72E-14 | 5.85E-14 | 21.68633457 |
| GPR35 | 0.703995974 | 5.714830934 | 7.971887039 | 1.87E-14 | 6.34E-14 | 21.60656583 |
| TDRD10 | -0.784082053 | 6.191975255 | -7.963422044 | 1.98E-14 | 6.72E-14 | 21.54877049 |
| GLT8D2 | -0.857255266 | 7.657185309 | -7.961904177 | 2.00E-14 | 6.78E-14 | 21.53841177 |
| HIST1H2AE | 0.699395898 | 4.946345553 | 7.95219055 | 2.14E-14 | 7.25E-14 | 21.47215422 |
| ANXA9 | 0.639893242 | 5.289418101 | 7.951945131 | 2.14E-14 | 7.26E-14 | 21.47048094 |
| OGDHL | 0.616096932 | 4.973271524 | 7.945790513 | 2.24E-14 | 7.57E-14 | 21.4285305 |
| TAOK1 | 0.612445141 | 7.778499803 | 7.944472426 | 2.26E-14 | 7.63E-14 | 21.4195493 |
| IER2 | -0.602397039 | 8.158792997 | -7.941566066 | 2.30E-14 | 7.78E-14 | 21.39974968 |
| SRL | -0.60490692 | 5.639284003 | -7.941114316 | 2.31E-14 | 7.80E-14 | 21.39667259 |
| S100A16 | 0.698020178 | 10.12391925 | 7.931424254 | 2.47E-14 | 8.33E-14 | 21.33069896 |
| IRAK3 | -0.816128495 | 7.617535593 | -7.931202662 | 2.47E-14 | 8.34E-14 | 21.32919095 |
| SCUBE3 | 0.60097643 | 5.574197114 | 7.931058727 | 2.47E-14 | 8.35E-14 | 21.32821144 |
| SLC16A9 | 1.248959043 | 6.408356231 | 7.921779211 | 2.64E-14 | 8.89E-14 | 21.26508898 |
| PLN | -0.670030862 | 5.448635894 | -7.918813494 | 2.69E-14 | 9.07E-14 | 21.24492629 |
| LRIG3 | 0.855201811 | 7.593801915 | 7.914562852 | 2.77E-14 | 9.33E-14 | 21.21603735 |
| HIST1H2BK | 0.59356003 | 11.16921484 | 7.908437602 | 2.89E-14 | 9.72E-14 | 21.17442739 |
| STEAP1 | 0.820569461 | 6.605109191 | 7.90314422 | 3.00E-14 | 1.01E-13 | 21.13848703 |
| PCSK5 | -0.680610855 | 7.671075096 | -7.898963801 | 3.09E-14 | 1.04E-13 | 21.11011552 |
| NFKBIZ | -0.612883781 | 8.853529918 | -7.882249039 | 3.46E-14 | 1.16E-13 | 20.99678389 |
| HCLS1 | -0.649844742 | 9.641194492 | -7.872326875 | 3.71E-14 | 1.24E-13 | 20.9295898 |
| CCL22 | 0.767039557 | 6.822105819 | 7.866725303 | 3.85E-14 | 1.28E-13 | 20.89168212 |
| SESTD1 | -0.586171573 | 8.34970067 | -7.866262559 | 3.87E-14 | 1.29E-13 | 20.88855144 |
| PIP5KL1 | 0.62278102 | 5.848660572 | 7.860496892 | 4.02E-14 | 1.34E-13 | 20.84955516 |
| TREM1 | -1.028057968 | 8.528808444 | -7.845628193 | 4.45E-14 | 1.48E-13 | 20.74908511 |
| GJA1 | -0.830963691 | 9.755069404 | -7.84401688 | 4.50E-14 | 1.49E-13 | 20.73820544 |
| SLC44A5 | 0.740351721 | 4.745010178 | 7.842577143 | 4.55E-14 | 1.51E-13 | 20.72848562 |
| CD19 | 0.906030519 | 6.274250154 | 7.840122428 | 4.63E-14 | 1.53E-13 | 20.71191654 |
| SLC36A4 | 0.693128638 | 7.287740125 | 7.832996808 | 4.86E-14 | 1.60E-13 | 20.66384049 |
| CCDC3 | -0.695682874 | 8.281852035 | -7.819927805 | 5.31E-14 | 1.75E-13 | 20.57574671 |
| CDC7 | 0.701562988 | 6.032639048 | 7.815847494 | 5.46E-14 | 1.80E-13 | 20.54826443 |
| RPS23 | -0.611896655 | 10.47848137 | -7.810515841 | 5.66E-14 | 1.86E-13 | 20.51236954 |
| TRIM17 | 0.626729415 | 5.61073529 | 7.806943661 | 5.80E-14 | 1.90E-13 | 20.48833004 |
| SNX10 | -0.883620295 | 8.129846545 | -7.803228864 | 5.95E-14 | 1.95E-13 | 20.46333919 |
| COL9A2 | 0.781770122 | 7.017980846 | 7.787197758 | 6.64E-14 | 2.17E-13 | 20.35559046 |
| CHST9 | -1.30351156 | 5.312802152 | -7.786176696 | 6.69E-14 | 2.18E-13 | 20.3487331 |
| DNALI1 | -0.881028384 | 8.12585266 | -7.78118224 | 6.92E-14 | 2.26E-13 | 20.31520013 |
| BASP1 | 0.703278217 | 10.16505633 | 7.773116116 | 7.31E-14 | 2.38E-13 | 20.26107676 |
| GPR84 | 0.682875374 | 5.568448673 | 7.766818341 | 7.63E-14 | 2.48E-13 | 20.2188472 |
| CST7 | -0.727125584 | 8.10807258 | -7.757204819 | 8.15E-14 | 2.65E-13 | 20.15443181 |
| ITK | -0.729920629 | 7.451635779 | -7.74222426 | 9.02E-14 | 2.92E-13 | 20.05416995 |
| SHC3 | -0.634859222 | 6.269514561 | -7.740083381 | 9.15E-14 | 2.97E-13 | 20.03985297 |
| TNFRSF12A | 0.688716716 | 9.645954141 | 7.737845421 | 9.29E-14 | 3.01E-13 | 20.02488983 |
| SCN1A | -0.66580474 | 4.560865769 | -7.728956175 | 9.87E-14 | 3.19E-13 | 19.96548685 |
| F13A1 | -1.08615032 | 8.435976476 | -7.716381988 | 1.07E-13 | 3.47E-13 | 19.88154376 |
| N4BP2 | 0.712118189 | 7.621755138 | 7.712163867 | 1.11E-13 | 3.56E-13 | 19.85340659 |
| IL10RA | -0.611269319 | 8.278949668 | -7.711360343 | 1.11E-13 | 3.58E-13 | 19.84804792 |
| TNFSF10 | -0.70918243 | 10.71752889 | -7.701665087 | 1.19E-13 | 3.82E-13 | 19.78342263 |
| CLDN7 | 0.599364965 | 10.15108844 | 7.701511317 | 1.19E-13 | 3.82E-13 | 19.78239813 |
| PBX1 | -0.591112106 | 7.780811269 | -7.692392253 | 1.26E-13 | 4.06E-13 | 19.72166849 |
| CD4 | -0.60475659 | 8.212405609 | -7.689954638 | 1.29E-13 | 4.12E-13 | 19.70544374 |
| KCTD7 | 0.623266776 | 6.35595758 | 7.687844157 | 1.30E-13 | 4.18E-13 | 19.69139943 |
| DCBLD2 | 0.617140593 | 8.893496875 | 7.681195716 | 1.36E-13 | 4.37E-13 | 19.64717539 |
| NME5 | -0.917838389 | 6.083195162 | -7.669816816 | 1.47E-13 | 4.71E-13 | 19.57155012 |
| SYNGR3 | 0.634997807 | 5.274426484 | 7.664195958 | 1.53E-13 | 4.88E-13 | 19.53422357 |
| RASEF | 0.864285115 | 8.03484537 | 7.661901464 | 1.55E-13 | 4.96E-13 | 19.51899223 |
| VTCN1 | 1.287474925 | 5.864235641 | 7.65991949 | 1.57E-13 | 5.02E-13 | 19.50583814 |
| TDRD5 | 0.681783908 | 4.413346532 | 7.658425959 | 1.59E-13 | 5.07E-13 | 19.49592742 |
| NR2F1 | -0.697563396 | 8.462532832 | -7.649134553 | 1.69E-13 | 5.39E-13 | 19.43430361 |
| PER3 | -0.672084025 | 7.612697561 | -7.634566605 | 1.87E-13 | 5.93E-13 | 19.33779417 |
| CRIP2 | -0.633552348 | 10.05270597 | -7.615724101 | 2.12E-13 | 6.70E-13 | 19.21316672 |
| ATP6V1C2 | 0.673174116 | 4.876703165 | 7.607177034 | 2.24E-13 | 7.08E-13 | 19.15670948 |
| MEGF9 | -0.68255622 | 7.547908221 | -7.603884391 | 2.29E-13 | 7.24E-13 | 19.1349725 |
| FAM90A1 | 0.588432261 | 5.090832803 | 7.602238144 | 2.32E-13 | 7.31E-13 | 19.1241071 |
| TNS4 | 0.757111899 | 5.819546715 | 7.597717416 | 2.39E-13 | 7.53E-13 | 19.09427871 |
| C22orf15 | -0.762388572 | 5.622949154 | -7.595654272 | 2.42E-13 | 7.63E-13 | 19.08067013 |
| NRGN | -0.922129611 | 8.024650146 | -7.593425166 | 2.46E-13 | 7.74E-13 | 19.06596991 |
| MUC4 | 0.77846819 | 5.944357261 | 7.590805255 | 2.50E-13 | 7.87E-13 | 19.04869652 |
| TEKT2 | -0.687658568 | 5.742503399 | -7.56510272 | 2.97E-13 | 9.32E-13 | 18.87946915 |
| ROS1 | -1.029278585 | 7.508908551 | -7.559142527 | 3.09E-13 | 9.69E-13 | 18.84028718 |
| C3AR1 | -0.733869326 | 7.947644596 | -7.554172277 | 3.20E-13 | 1.00E-12 | 18.80763046 |
| PAX9 | 0.644199641 | 5.235582444 | 7.538837919 | 3.54E-13 | 1.10E-12 | 18.70697684 |
| TNNI3 | 0.71535401 | 5.615768795 | 7.527337794 | 3.83E-13 | 1.19E-12 | 18.63158995 |
| ELF5 | -0.922980667 | 6.222720106 | -7.524529287 | 3.90E-13 | 1.21E-12 | 18.61319224 |
| NINJ2 | -0.687990853 | 9.115086689 | -7.52361101 | 3.92E-13 | 1.22E-12 | 18.60717797 |
| SAMSN1 | -0.67473968 | 6.456608612 | -7.514170772 | 4.18E-13 | 1.29E-12 | 18.5453805 |
| TFAP2D | 0.974629113 | 4.695541481 | 7.504969442 | 4.44E-13 | 1.37E-12 | 18.48520221 |
| HSPA2 | -0.833235179 | 7.300638282 | -7.494935842 | 4.75E-13 | 1.46E-12 | 18.41964297 |
| RNF183 | 0.644887081 | 5.333978375 | 7.492077329 | 4.84E-13 | 1.49E-12 | 18.40097742 |
| CRLF1 | 1.39459968 | 7.312341532 | 7.482924546 | 5.14E-13 | 1.58E-12 | 18.341247 |
| NOX4 | 0.870470927 | 6.859210822 | 7.472170369 | 5.52E-13 | 1.70E-12 | 18.27113521 |
| S100P | 1.722149214 | 8.911680423 | 7.46308479 | 5.86E-13 | 1.80E-12 | 18.21196017 |
| TNFSF15 | 0.650901448 | 6.396315883 | 7.455264156 | 6.17E-13 | 1.90E-12 | 18.16106664 |
| HMOX1 | -0.673635182 | 8.326479513 | -7.448216493 | 6.47E-13 | 1.98E-12 | 18.11523729 |
| MBD4 | 0.671918236 | 9.883087572 | 7.443634115 | 6.67E-13 | 2.04E-12 | 18.08545642 |
| DCDC2 | -0.6656919 | 5.087502875 | -7.443015463 | 6.69E-13 | 2.05E-12 | 18.08143684 |
| BARX1 | 1.003556337 | 5.61985671 | 7.433397811 | 7.13E-13 | 2.18E-12 | 18.01897995 |
| ARPC1B | -0.640673927 | 10.11785821 | -7.427901186 | 7.39E-13 | 2.26E-12 | 17.98331196 |
| C1orf192 | -0.924599886 | 5.422701154 | -7.423766127 | 7.60E-13 | 2.32E-12 | 17.95649222 |
| KIF21A | 0.656216273 | 7.083566926 | 7.421607191 | 7.71E-13 | 2.35E-12 | 17.94249392 |
| TTC25 | -0.765086408 | 6.009276207 | -7.416733926 | 7.96E-13 | 2.42E-12 | 17.91090736 |
| LARP6 | -0.659114128 | 7.184555356 | -7.40937812 | 8.35E-13 | 2.54E-12 | 17.86325927 |
| ERN2 | 0.896154193 | 6.137783609 | 7.407897255 | 8.44E-13 | 2.57E-12 | 17.85367106 |
| IQGAP2 | -0.748518984 | 7.516089045 | -7.402661731 | 8.73E-13 | 2.65E-12 | 17.81978389 |
| GLRX | -0.621543864 | 9.132316056 | -7.383756308 | 9.89E-13 | 2.99E-12 | 17.69756666 |
| CRABP1 | 0.853850838 | 5.075086106 | 7.382648498 | 9.96E-13 | 3.02E-12 | 17.69041228 |
| NT5E | 0.784210716 | 7.383388904 | 7.382166174 | 9.99E-13 | 3.02E-12 | 17.68729762 |
| SOX11 | 0.593940737 | 4.979628561 | 7.371218303 | 1.07E-12 | 3.24E-12 | 17.61664153 |
| MUC13 | 1.290754348 | 5.234794093 | 7.352310261 | 1.22E-12 | 3.66E-12 | 17.49479645 |
| RNF128 | -0.759122561 | 6.560902104 | -7.3400975 | 1.32E-12 | 3.95E-12 | 17.41622105 |
| TRPM8 | 0.971313279 | 4.461605287 | 7.337153401 | 1.34E-12 | 4.03E-12 | 17.39729373 |
| FGL1 | 0.960029572 | 5.011920816 | 7.322747029 | 1.47E-12 | 4.42E-12 | 17.30475882 |
| INHA | 0.734297252 | 4.97220588 | 7.320487607 | 1.50E-12 | 4.48E-12 | 17.29025851 |
| IRF6 | 0.608867149 | 7.094533168 | 7.310343728 | 1.60E-12 | 4.78E-12 | 17.2251995 |
| FOXE1 | 0.673024223 | 4.790873902 | 7.302319708 | 1.68E-12 | 5.03E-12 | 17.17378453 |
| PSG1 | 0.951535289 | 5.046597394 | 7.297060551 | 1.74E-12 | 5.20E-12 | 17.14010883 |
| CYSLTR1 | -0.689301465 | 6.770167686 | -7.292704556 | 1.79E-12 | 5.34E-12 | 17.11223014 |
| CIT | 0.649049729 | 5.989002702 | 7.290491637 | 1.82E-12 | 5.41E-12 | 17.0980721 |
| CD48 | -0.760198133 | 8.283094641 | -7.283728189 | 1.90E-12 | 5.65E-12 | 17.05482027 |
| APOBEC3B | 0.922217659 | 5.933075434 | 7.279627776 | 1.95E-12 | 5.79E-12 | 17.02861311 |
| G0S2 | -0.853497147 | 8.039541085 | -7.266395791 | 2.13E-12 | 6.30E-12 | 16.94411883 |
| KLK6 | 0.787436533 | 5.107592434 | 7.259524401 | 2.22E-12 | 6.58E-12 | 16.90028658 |
| ST6GALNAC1 | 1.167047985 | 7.446711596 | 7.246765756 | 2.42E-12 | 7.14E-12 | 16.8189829 |
| HIST1H1C | 0.690278034 | 9.261046109 | 7.244804297 | 2.45E-12 | 7.22E-12 | 16.80649321 |
| PNMA3 | 0.634475036 | 5.866144532 | 7.24273724 | 2.48E-12 | 7.31E-12 | 16.79333388 |
| NUPR1 | -0.649952028 | 9.645047968 | -7.239892061 | 2.53E-12 | 7.44E-12 | 16.7752255 |
| TESC | 1.049913968 | 8.655345441 | 7.239700817 | 2.53E-12 | 7.45E-12 | 16.77400851 |
| KLK11 | -0.920321233 | 7.113234723 | -7.23645083 | 2.58E-12 | 7.60E-12 | 16.7533307 |
| PAEP | 1.461723812 | 5.808717604 | 7.234855278 | 2.61E-12 | 7.68E-12 | 16.74318168 |
| GAL | 0.96538217 | 5.174613309 | 7.229600384 | 2.70E-12 | 7.93E-12 | 16.70976823 |
| LY6K | 0.717296863 | 5.478375596 | 7.223940145 | 2.80E-12 | 8.22E-12 | 16.67379793 |
| SLC6A3 | 0.866647421 | 5.848858388 | 7.214824304 | 2.97E-12 | 8.71E-12 | 16.61591244 |
| NPR3 | -0.760778282 | 6.240099035 | -7.211568972 | 3.03E-12 | 8.88E-12 | 16.59525454 |
| PELI2 | -0.594295293 | 7.556675636 | -7.207358776 | 3.12E-12 | 9.11E-12 | 16.56854768 |
| PTPRN2 | -0.653271719 | 5.946325221 | -7.196629522 | 3.34E-12 | 9.75E-12 | 16.50054145 |
| CD79A | 0.809475779 | 6.948768221 | 7.182200371 | 3.67E-12 | 1.07E-11 | 16.40920505 |
| AGR3 | -1.316237987 | 8.028342247 | -7.174576986 | 3.85E-12 | 1.12E-11 | 16.36100531 |
| CHI3L1 | 0.738601961 | 6.941435311 | 7.16773748 | 4.02E-12 | 1.17E-11 | 16.31779485 |
| FBXL16 | 0.796258216 | 6.468002718 | 7.161921145 | 4.18E-12 | 1.21E-11 | 16.28107322 |
| GAS1 | -0.919462923 | 8.008516745 | -7.137553902 | 4.89E-12 | 1.41E-11 | 16.1274765 |
| IGFL2 | 0.775525094 | 5.163464755 | 7.136895361 | 4.91E-12 | 1.42E-11 | 16.12333099 |
| ZNF114 | 0.618322352 | 4.894520678 | 7.123920532 | 5.33E-12 | 1.54E-11 | 16.04171386 |
| TFF1 | 1.516009794 | 5.938729513 | 7.119612359 | 5.48E-12 | 1.58E-11 | 16.0146387 |
| KCNJ2 | -0.683128915 | 7.283034191 | -7.118725655 | 5.51E-12 | 1.59E-11 | 16.00906767 |
| CRIP1 | -0.610608057 | 10.92598722 | -7.117409965 | 5.56E-12 | 1.60E-11 | 16.00080236 |
| TTC18 | -0.74462901 | 5.391170593 | -7.080038843 | 7.06E-12 | 2.02E-11 | 15.76652047 |
| CEL | 0.878837477 | 5.529859439 | 7.074745534 | 7.30E-12 | 2.09E-11 | 15.7334127 |
| ECM1 | 0.763276533 | 7.748536301 | 7.061839433 | 7.92E-12 | 2.26E-11 | 15.65276912 |
| CYP3A5 | -1.299407151 | 6.349061149 | -7.059639151 | 8.03E-12 | 2.29E-11 | 15.63903195 |
| TUBB2B | 1.049251826 | 6.685424146 | 7.057389127 | 8.15E-12 | 2.33E-11 | 15.62498762 |
| CLC | -0.972896372 | 5.239448521 | -7.048707107 | 8.61E-12 | 2.45E-11 | 15.57082787 |
| KCND2 | 0.684643538 | 4.697971952 | 7.034258489 | 9.44E-12 | 2.68E-11 | 15.48080868 |
| NMNAT2 | 0.641627579 | 5.271000399 | 7.006420846 | 1.13E-11 | 3.19E-11 | 15.30777227 |
| MBOAT2 | 0.755755525 | 8.153606258 | 7.00076455 | 1.17E-11 | 3.30E-11 | 15.27267776 |
| VMO1 | -0.646571742 | 6.547550891 | -6.992280929 | 1.23E-11 | 3.47E-11 | 15.22008204 |
| LRP2 | -0.792904459 | 5.655119601 | -6.959744335 | 1.51E-11 | 4.23E-11 | 15.01882185 |
| SHROOM4 | -0.676968749 | 7.874555551 | -6.95858629 | 1.52E-11 | 4.26E-11 | 15.01167194 |
| TCN1 | 1.642117742 | 5.492732016 | 6.957320494 | 1.53E-11 | 4.29E-11 | 15.00385781 |
| INPP4B | 0.782319215 | 7.153004779 | 6.95690968 | 1.54E-11 | 4.30E-11 | 15.00132197 |
| GJB1 | 0.688400422 | 5.855275059 | 6.946064148 | 1.65E-11 | 4.60E-11 | 14.93441745 |
| TOM1L1 | 0.609397056 | 6.761873077 | 6.936639222 | 1.75E-11 | 4.87E-11 | 14.87634201 |
| SMOC1 | 0.590247169 | 5.468769585 | 6.934348093 | 1.77E-11 | 4.93E-11 | 14.86223352 |
| MOCOS | 0.756651066 | 7.18708383 | 6.930988235 | 1.81E-11 | 5.03E-11 | 14.84155046 |
| ZKSCAN1 | 0.630855824 | 6.55163058 | 6.928612785 | 1.84E-11 | 5.11E-11 | 14.82693203 |
| DUSP4 | 0.778768011 | 5.955912832 | 6.920564622 | 1.93E-11 | 5.37E-11 | 14.77743281 |
| E2F5 | 0.636603731 | 7.154724295 | 6.908435055 | 2.09E-11 | 5.77E-11 | 14.70291565 |
| HAS1 | -0.764266723 | 5.720166665 | -6.899499138 | 2.20E-11 | 6.09E-11 | 14.64808333 |
| ADH1C | -1.078828887 | 6.415525726 | -6.889103926 | 2.35E-11 | 6.49E-11 | 14.58436583 |
| ANXA1 | -0.587374901 | 11.98761005 | -6.883494275 | 2.44E-11 | 6.70E-11 | 14.55001246 |
| EDIL3 | -0.617281981 | 6.503538516 | -6.879381581 | 2.50E-11 | 6.87E-11 | 14.52484022 |
| CNR1 | -0.705241327 | 5.138882577 | -6.874515188 | 2.58E-11 | 7.07E-11 | 14.49506999 |
| PLCB4 | -0.67842701 | 5.572748614 | -6.867428734 | 2.69E-11 | 7.38E-11 | 14.4517478 |
| RGS16 | -0.67322479 | 8.178768838 | -6.861043563 | 2.80E-11 | 7.67E-11 | 14.4127426 |
| GFRA3 | 1.104990161 | 5.870474588 | 6.847993355 | 3.04E-11 | 8.31E-11 | 14.33311042 |
| HOXC10 | 0.679824873 | 4.858998559 | 6.838683981 | 3.22E-11 | 8.79E-11 | 14.27637695 |
| GNA14 | -0.626271361 | 6.515290537 | -6.829573388 | 3.41E-11 | 9.29E-11 | 14.22091316 |
| GJB6 | 0.989141282 | 4.823828582 | 6.823348989 | 3.54E-11 | 9.64E-11 | 14.1830532 |
| C11orf70 | -0.743907737 | 5.882024277 | -6.820480419 | 3.61E-11 | 9.81E-11 | 14.16561417 |
| SERPINB5 | 1.337676754 | 5.410465622 | 6.804012523 | 3.99E-11 | 1.08E-10 | 14.06561095 |
| CCL24 | -0.591400348 | 5.107607601 | -6.801728427 | 4.05E-11 | 1.10E-10 | 14.05175543 |
| MTMR12 | -0.627300054 | 7.474631048 | -6.794406907 | 4.24E-11 | 1.15E-10 | 14.00736698 |
| RAB38 | 0.681069212 | 7.978647032 | 6.788642848 | 4.39E-11 | 1.19E-10 | 13.97244731 |
| NPC1L1 | 0.689237287 | 5.267411657 | 6.762155522 | 5.17E-11 | 1.39E-10 | 13.81228086 |
| ARHGAP20 | -0.727383024 | 6.196648074 | -6.748555355 | 5.62E-11 | 1.51E-10 | 13.73023261 |
| KIAA0125 | 0.609170872 | 5.422944441 | 6.745583389 | 5.73E-11 | 1.54E-10 | 13.71232034 |
| LY6D | 0.781929718 | 5.401392154 | 6.732302845 | 6.21E-11 | 1.66E-10 | 13.63235314 |
| APOC1 | -0.707443423 | 10.54217979 | -6.689251215 | 8.09E-11 | 2.14E-10 | 13.37397525 |
| ANGPTL4 | 0.671981059 | 6.66142521 | 6.667437654 | 9.24E-11 | 2.44E-10 | 13.24355799 |
| VIL1 | 0.811210456 | 4.930781971 | 6.660308715 | 9.65E-11 | 2.55E-10 | 13.20100891 |
| IRX2 | -1.01076242 | 8.502725859 | -6.644126149 | 1.06E-10 | 2.80E-10 | 13.10455675 |
| CEBPA | -0.615794323 | 9.02883621 | -6.633516377 | 1.14E-10 | 2.99E-10 | 13.04142053 |
| STEAP4 | -0.91376254 | 7.644832431 | -6.629639781 | 1.16E-10 | 3.06E-10 | 13.01837174 |
| PITX2 | 0.614106441 | 4.817124455 | 6.627925784 | 1.17E-10 | 3.09E-10 | 13.00818436 |
| NETO2 | 0.742742235 | 7.668304359 | 6.625447325 | 1.19E-10 | 3.13E-10 | 12.99345698 |
| CH25H | -1.021437569 | 7.943834745 | -6.619647834 | 1.24E-10 | 3.24E-10 | 12.95901256 |
| ANPEP | -0.91734919 | 7.941016681 | -6.61810073 | 1.25E-10 | 3.27E-10 | 12.94982802 |
| CXCL5 | -1.09319782 | 6.164545955 | -6.605790012 | 1.34E-10 | 3.51E-10 | 12.87680478 |
| SYT13 | 1.040169155 | 5.232187229 | 6.604276161 | 1.36E-10 | 3.54E-10 | 12.86783253 |
| SCNN1B | -0.754750103 | 8.377560628 | -6.594557857 | 1.44E-10 | 3.75E-10 | 12.81027327 |
| RAPGEFL1 | 0.638263425 | 6.81818212 | 6.576839479 | 1.60E-10 | 4.16E-10 | 12.70550459 |
| UCHL1 | 1.070249147 | 7.355934614 | 6.567347891 | 1.69E-10 | 4.40E-10 | 12.649473 |
| IFIT1 | -0.591757946 | 7.427289178 | -6.56012675 | 1.77E-10 | 4.58E-10 | 12.60688759 |
| CXCL14 | 1.147303145 | 7.579356742 | 6.539940464 | 2.00E-10 | 5.15E-10 | 12.48804033 |
| KLHL6 | -0.598396539 | 6.711633367 | -6.52775643 | 2.15E-10 | 5.53E-10 | 12.41644772 |
| TMEM125 | -0.644823028 | 9.736622059 | -6.525069025 | 2.18E-10 | 5.61E-10 | 12.40067102 |
| NFE2 | -0.593842414 | 6.48863687 | -6.519277733 | 2.26E-10 | 5.80E-10 | 12.36669024 |
| PLA2G2A | -1.023011482 | 6.210338223 | -6.491252339 | 2.67E-10 | 6.84E-10 | 12.20258974 |
| DLX5 | 0.654105307 | 4.985364846 | 6.482622983 | 2.82E-10 | 7.19E-10 | 12.15217497 |
| CILP | 0.982614816 | 6.412869184 | 6.482222149 | 2.82E-10 | 7.21E-10 | 12.1498345 |
| INA | 0.706573818 | 4.795477633 | 6.47838302 | 2.89E-10 | 7.37E-10 | 12.12742374 |
| HOXB7 | 0.664923885 | 6.857468481 | 6.464063248 | 3.15E-10 | 8.00E-10 | 12.04392635 |
| PRRX2 | 0.605742881 | 7.302524665 | 6.45777379 | 3.27E-10 | 8.30E-10 | 12.0072998 |
| PHYHD1 | -0.672993658 | 7.325749785 | -6.453904829 | 3.34E-10 | 8.49E-10 | 11.98478317 |
| MT1X | -0.708146395 | 10.63964256 | -6.441396142 | 3.60E-10 | 9.11E-10 | 11.91205895 |
| CDH17 | 0.975478167 | 4.688843739 | 6.430232977 | 3.85E-10 | 9.72E-10 | 11.84725303 |
| RNF39 | 0.687680299 | 6.570805194 | 6.429931793 | 3.85E-10 | 9.73E-10 | 11.8455058 |
| QPCT | 0.65090061 | 6.942690229 | 6.415764232 | 4.19E-10 | 1.06E-09 | 11.76339138 |
| FOLR2 | -0.731533381 | 8.168446465 | -6.411006008 | 4.31E-10 | 1.09E-09 | 11.73584561 |
| KRT6B | 1.076442378 | 5.557036989 | 6.399253809 | 4.62E-10 | 1.16E-09 | 11.66788153 |
| SERPINE2 | 0.724990798 | 8.899980231 | 6.394812635 | 4.74E-10 | 1.19E-09 | 11.6422239 |
| TP53 | 0.586496593 | 6.869199285 | 6.379748394 | 5.18E-10 | 1.30E-09 | 11.55530132 |
| RBP1 | -0.617442521 | 8.46753171 | -6.364649967 | 5.67E-10 | 1.42E-09 | 11.4683472 |
| HMCN1 | -0.594570782 | 6.046416301 | -6.358668902 | 5.87E-10 | 1.47E-09 | 11.43394728 |
| CCL5 | -0.645481157 | 9.903005718 | -6.355015977 | 6.00E-10 | 1.50E-09 | 11.41295042 |
| CCNA1 | -0.666767182 | 5.060638051 | -6.353285103 | 6.06E-10 | 1.51E-09 | 11.40300482 |
| CDH15 | 0.619149787 | 6.242351024 | 6.350741396 | 6.15E-10 | 1.53E-09 | 11.38839266 |
| SELL | -0.759601813 | 7.19601454 | -6.342338956 | 6.46E-10 | 1.61E-09 | 11.34015895 |
| RDH10 | 0.643510063 | 8.744141463 | 6.3324298 | 6.85E-10 | 1.70E-09 | 11.2833423 |
| SLC4A4 | -0.647563736 | 6.343047585 | -6.30155502 | 8.20E-10 | 2.03E-09 | 11.1067746 |
| TMPRSS2 | -0.754963811 | 9.073260987 | -6.294762249 | 8.53E-10 | 2.11E-09 | 11.06802163 |
| SPRR1A | 0.80347544 | 5.564236582 | 6.28917618 | 8.82E-10 | 2.18E-09 | 11.0361783 |
| VSNL1 | -0.643374695 | 5.658212173 | -6.286472444 | 8.96E-10 | 2.21E-09 | 11.02077391 |
| C5 | -0.822228251 | 8.293747215 | -6.24910894 | 1.11E-09 | 2.73E-09 | 10.80844771 |
| PLA2G4A | 0.871398997 | 6.448691402 | 6.24040968 | 1.17E-09 | 2.87E-09 | 10.75915983 |
| HIST1H4H | 0.692378797 | 6.308565279 | 6.238234944 | 1.19E-09 | 2.90E-09 | 10.74684703 |
| PIK3AP1 | -0.597778436 | 6.898366742 | -6.233769405 | 1.22E-09 | 2.97E-09 | 10.72157523 |
| POSTN | 0.847318884 | 8.005773229 | 6.222972119 | 1.29E-09 | 3.15E-09 | 10.66053104 |
| WBSCR27 | 0.58717774 | 6.926176269 | 6.204677375 | 1.44E-09 | 3.50E-09 | 10.55729548 |
| DPYD | -0.5991857 | 8.379085779 | -6.177126353 | 1.69E-09 | 4.08E-09 | 10.40229526 |
| IER3 | 0.625602582 | 10.34464454 | 6.176178098 | 1.70E-09 | 4.10E-09 | 10.39697047 |
| FGB | 1.374207822 | 5.260280601 | 6.154377283 | 1.92E-09 | 4.64E-09 | 10.27473511 |
| VSIG1 | 1.13459314 | 5.464261864 | 6.110659247 | 2.47E-09 | 5.91E-09 | 10.03067782 |
| CCL18 | -0.950423351 | 9.01206588 | -6.09050521 | 2.77E-09 | 6.60E-09 | 9.918647702 |
| MMP10 | 1.00826832 | 5.026718601 | 6.089726371 | 2.78E-09 | 6.63E-09 | 9.914324468 |
| BANK1 | -0.598836486 | 6.938777822 | -6.08532032 | 2.85E-09 | 6.79E-09 | 9.889875626 |
| SLAMF7 | 0.664532951 | 6.495681875 | 6.079854047 | 2.94E-09 | 7.00E-09 | 9.859563892 |
| CEACAM7 | 0.613089138 | 4.410961402 | 6.07771564 | 2.98E-09 | 7.08E-09 | 9.847712024 |
| CHGB | 0.936998116 | 5.124371742 | 6.039188492 | 3.70E-09 | 8.74E-09 | 9.634767558 |
| S100A2 | 0.837025773 | 7.042386596 | 6.038653721 | 3.71E-09 | 8.76E-09 | 9.631819657 |
| BACE2 | 0.617737947 | 8.642704713 | 6.021872922 | 4.08E-09 | 9.60E-09 | 9.539425535 |
| KRT14 | 0.926396463 | 5.316912638 | 6.011650324 | 4.33E-09 | 1.01E-08 | 9.483244435 |
| CR2 | 0.586723799 | 4.776241085 | 6.010937074 | 4.34E-09 | 1.02E-08 | 9.479327516 |
| WDR72 | 1.098013584 | 5.457181128 | 5.998963508 | 4.65E-09 | 1.09E-08 | 9.413630125 |
| SCGB3A1 | -1.344731549 | 10.69862868 | -5.980026137 | 5.17E-09 | 1.21E-08 | 9.309944235 |
| FHOD3 | 0.678749879 | 5.820939274 | 5.967086041 | 5.56E-09 | 1.29E-08 | 9.239250556 |
| TNNI2 | -0.589511124 | 6.027026231 | -5.960574644 | 5.76E-09 | 1.34E-08 | 9.203725757 |
| TFPI | -0.680096746 | 9.127254247 | -5.958660499 | 5.82E-09 | 1.36E-08 | 9.193288699 |
| ARL14 | 0.760904189 | 4.543531569 | 5.957671563 | 5.86E-09 | 1.36E-08 | 9.187897517 |
| GDF15 | 0.917419442 | 9.401460428 | 5.956050683 | 5.91E-09 | 1.37E-08 | 9.179062898 |
| ATHL1 | 0.778375711 | 8.434899561 | 5.953920613 | 5.98E-09 | 1.39E-08 | 9.167455961 |
| NUBPL | 0.594799454 | 8.803223109 | 5.948699116 | 6.16E-09 | 1.43E-08 | 9.139018122 |
| OR7E37P | -0.586819965 | 7.603392713 | -5.937012546 | 6.57E-09 | 1.52E-08 | 9.075444494 |
| CALML5 | 0.669841442 | 5.34418708 | 5.928662997 | 6.89E-09 | 1.59E-08 | 9.030087381 |
| ELOVL6 | 0.644761159 | 6.108496731 | 5.916576898 | 7.36E-09 | 1.70E-08 | 8.964526118 |
| CLDN2 | 0.954002611 | 5.658422537 | 5.908613041 | 7.70E-09 | 1.77E-08 | 8.92138675 |
| NPTX2 | 0.768759286 | 6.018053465 | 5.905102056 | 7.85E-09 | 1.81E-08 | 8.902383447 |
| PKP2 | 0.794679605 | 5.965679138 | 5.895841057 | 8.26E-09 | 1.90E-08 | 8.852303074 |
| F5 | 0.639323221 | 6.123066191 | 5.889993762 | 8.54E-09 | 1.96E-08 | 8.820716528 |
| HLA-DQA1 | -1.036736906 | 10.68753072 | -5.886706158 | 8.69E-09 | 1.99E-08 | 8.802968642 |
| GABBR2 | 0.701527562 | 5.185805048 | 5.866860285 | 9.70E-09 | 2.21E-08 | 8.696007346 |
| SLC27A2 | 0.817618226 | 6.669647915 | 5.857366633 | 1.02E-08 | 2.33E-08 | 8.644946756 |
| CLDN1 | 0.862047138 | 8.669259258 | 5.85603085 | 1.03E-08 | 2.35E-08 | 8.637767918 |
| FXYD3 | -0.701117713 | 7.891494324 | -5.849249491 | 1.07E-08 | 2.43E-08 | 8.60134423 |
| CYP4X1 | -0.796347966 | 7.246315955 | -5.83735962 | 1.14E-08 | 2.59E-08 | 8.537566931 |
| SERPINA1 | -0.71079253 | 10.10469213 | -5.823183018 | 1.23E-08 | 2.80E-08 | 8.461665169 |
| BCAS1 | 0.79593806 | 5.943729862 | 5.784606653 | 1.52E-08 | 3.43E-08 | 8.25590782 |
| PPP1R14C | -0.688827286 | 7.706403505 | -5.780506618 | 1.56E-08 | 3.51E-08 | 8.234106464 |
| AGT | 0.841444809 | 6.038816372 | 5.763742844 | 1.71E-08 | 3.84E-08 | 8.145102202 |
| CASC1 | -0.783287175 | 5.228778463 | -5.755043767 | 1.79E-08 | 4.02E-08 | 8.099001404 |
| HMGCS2 | -0.936741495 | 5.826061053 | -5.730096632 | 2.05E-08 | 4.57E-08 | 7.967118047 |
| HIST3H2A | 0.671585721 | 6.173848059 | 5.708890712 | 2.30E-08 | 5.11E-08 | 7.855391182 |
| QPRT | 0.611235041 | 8.333417074 | 5.706018754 | 2.34E-08 | 5.19E-08 | 7.840286583 |
| GZMA | -0.648237108 | 7.900063213 | -5.686788662 | 2.59E-08 | 5.74E-08 | 7.739313855 |
| SNAI2 | -0.624922165 | 7.573950295 | -5.683906022 | 2.63E-08 | 5.83E-08 | 7.724202523 |
| RND1 | -0.861792917 | 7.038084633 | -5.662812175 | 2.95E-08 | 6.50E-08 | 7.613821198 |
| GGH | 0.614312123 | 7.083506104 | 5.646805055 | 3.21E-08 | 7.07E-08 | 7.530289091 |
| GZMB | -0.598408969 | 8.279865056 | -5.56185634 | 5.05E-08 | 1.10E-07 | 7.090338181 |
| NAPSA | -0.982326935 | 12.41274691 | -5.560357672 | 5.09E-08 | 1.10E-07 | 7.082627284 |
| MAGEA12 | 0.591162158 | 4.742520463 | 5.557842714 | 5.16E-08 | 1.12E-07 | 7.069691373 |
| SPRR3 | 0.85454637 | 4.994247447 | 5.526904022 | 6.08E-08 | 1.31E-07 | 6.910962208 |
| CDA | 0.627246396 | 7.569203981 | 5.509891522 | 6.65E-08 | 1.43E-07 | 6.824001631 |
| TDO2 | 0.597194253 | 5.677391128 | 5.504990211 | 6.82E-08 | 1.46E-07 | 6.798990575 |
| MFAP5 | -1.023834524 | 6.573447649 | -5.499017757 | 7.04E-08 | 1.51E-07 | 6.768539167 |
| SPINK4 | 0.637800218 | 4.824666226 | 5.498898819 | 7.05E-08 | 1.51E-07 | 6.76793303 |
| SPRR1B | 0.930033825 | 5.701812327 | 5.456608567 | 8.79E-08 | 1.87E-07 | 6.553120518 |
| ACE2 | 0.649056056 | 5.450642415 | 5.411096496 | 1.11E-07 | 2.35E-07 | 6.32352584 |
| COL14A1 | -0.64567876 | 7.012534418 | -5.356644698 | 1.48E-07 | 3.09E-07 | 6.050997876 |
| FGF9 | -0.658277456 | 5.865726431 | -5.33549671 | 1.64E-07 | 3.43E-07 | 5.945791515 |
| FOXQ1 | 0.739542544 | 9.253214745 | 5.329640511 | 1.69E-07 | 3.53E-07 | 5.91672153 |
| TFF2 | 0.841814553 | 5.090194412 | 5.312235185 | 1.85E-07 | 3.85E-07 | 5.830484195 |
| CNTNAP2 | 0.612204228 | 4.920357588 | 5.248059941 | 2.57E-07 | 5.27E-07 | 5.514619944 |
| MT1G | -0.665399823 | 9.483913277 | -5.226303847 | 2.86E-07 | 5.87E-07 | 5.408291232 |
| CAPS | -0.654526258 | 7.966189277 | -5.217971204 | 2.99E-07 | 6.10E-07 | 5.367668239 |
| SOX9 | 0.71937619 | 7.427874476 | 5.176353392 | 3.68E-07 | 7.48E-07 | 5.165615327 |
| KLK12 | 0.758850287 | 5.416627391 | 5.172946088 | 3.74E-07 | 7.61E-07 | 5.149135125 |
| C4BPB | 0.604658481 | 5.65860579 | 5.14220487 | 4.37E-07 | 8.82E-07 | 5.000874243 |
| C1orf116 | -0.666453688 | 9.635980963 | -5.132552212 | 4.58E-07 | 9.23E-07 | 4.954479148 |
| SCTR | -0.667907501 | 7.582867503 | -5.13081691 | 4.62E-07 | 9.30E-07 | 4.94614653 |
| SLC34A2 | -0.838500365 | 9.707054027 | -5.11834652 | 4.91E-07 | 9.87E-07 | 4.886337995 |
| SLC1A7 | 0.805493752 | 6.004434181 | 5.084153668 | 5.82E-07 | 1.16E-06 | 4.722997819 |
| CD1A | 0.754220621 | 6.303232654 | 5.075424983 | 6.08E-07 | 1.21E-06 | 4.68145356 |
| CYP4F11 | 0.752343829 | 5.269403404 | 5.002435777 | 8.68E-07 | 1.71E-06 | 4.336502549 |
| SCIN | 0.659976529 | 7.125012497 | 4.939428826 | 1.18E-06 | 2.30E-06 | 4.042249412 |
| MUC15 | -0.695510985 | 6.13818916 | -4.926101171 | 1.26E-06 | 2.45E-06 | 3.980426699 |
| SLC16A14 | 0.68132175 | 6.582322699 | 4.919804722 | 1.29E-06 | 2.52E-06 | 3.951270578 |
| TNC | 0.607820965 | 8.525718923 | 4.90560786 | 1.39E-06 | 2.69E-06 | 3.885651449 |
| LRRC31 | 0.689559337 | 5.60501467 | 4.896578581 | 1.45E-06 | 2.80E-06 | 3.844004177 |
| NELL2 | -0.615843924 | 6.602369508 | -4.879374987 | 1.57E-06 | 3.03E-06 | 3.764840192 |
| CCL19 | 0.670550644 | 8.284695519 | 4.85457009 | 1.77E-06 | 3.39E-06 | 3.651130571 |
| WDR66 | 0.891318931 | 6.549272181 | 4.838698681 | 1.91E-06 | 3.65E-06 | 3.578641833 |
| C8orf4 | -0.623730475 | 9.809674832 | -4.807216493 | 2.21E-06 | 4.22E-06 | 3.4354755 |
| MMP7 | 0.875201957 | 8.777693827 | 4.800088634 | 2.29E-06 | 4.36E-06 | 3.403176077 |
| PTHLH | 0.64351038 | 5.250476968 | 4.798851861 | 2.30E-06 | 4.38E-06 | 3.397576036 |
| APOH | -0.660428382 | 5.528021165 | -4.754237651 | 2.84E-06 | 5.36E-06 | 3.196420389 |
| MYCN | 0.587597746 | 5.422165122 | 4.732305128 | 3.14E-06 | 5.92E-06 | 3.098142514 |
| PLA2G7 | 0.64384554 | 7.783887101 | 4.706242798 | 3.54E-06 | 6.65E-06 | 2.981884186 |
| TMEM27 | 0.608514358 | 5.482992415 | 4.627128217 | 5.10E-06 | 9.46E-06 | 2.632473958 |
| PTGS2 | -0.795654354 | 9.172331694 | -4.5585837 | 6.96E-06 | 1.27E-05 | 2.334024793 |
| ATP13A4 | -0.63065223 | 6.77627184 | -4.531914426 | 7.85E-06 | 1.43E-05 | 2.218982172 |
| CAPN9 | -0.629006289 | 6.117419596 | -4.41411316 | 1.32E-05 | 2.38E-05 | 1.718086203 |
| NELL1 | 0.58669911 | 5.313595543 | 4.355997684 | 1.71E-05 | 3.03E-05 | 1.47535893 |
| TMC5 | 0.595826509 | 7.875355239 | 4.319921088 | 2.00E-05 | 3.52E-05 | 1.326143746 |
| GRP | -0.600286333 | 6.207148947 | -4.301935186 | 2.16E-05 | 3.79E-05 | 1.252172767 |
| OLFM4 | 0.826529152 | 4.43247692 | 4.301647598 | 2.16E-05 | 3.80E-05 | 1.250992272 |
| CEACAM6 | 0.660717677 | 12.1128307 | 4.281667795 | 2.35E-05 | 4.12E-05 | 1.169154018 |
| HLA-DQB1 | -0.709433616 | 9.369948537 | -4.233625535 | 2.89E-05 | 5.02E-05 | 0.97378687 |
| TNFRSF17 | 0.616276525 | 6.051287508 | 4.187048802 | 3.52E-05 | 6.07E-05 | 0.786294236 |
| CA12 | 0.614811244 | 7.208069787 | 4.151964837 | 4.08E-05 | 6.99E-05 | 0.646313631 |
| IGJ | -0.624047302 | 10.67034921 | -3.925261964 | 0.00010292 | 0.000170909 | -0.232169435 |
| GSTA1 | -0.663125684 | 6.688555902 | -3.903088092 | 0.00011242 | 0.000186168 | -0.315658828 |
| CXCL9 | 0.621589759 | 8.517197973 | 3.853205006 | 0.000136914 | 0.000225277 | -0.501883355 |
| PIGR | -0.695534191 | 10.18996825 | -3.849619354 | 0.000138857 | 0.000228264 | -0.515184125 |
| SLC6A14 | -0.632755034 | 6.975160569 | -3.805252808 | 0.000165154 | 0.000269928 | -0.678811273 |
| MSLN | -0.723340805 | 9.526556955 | -3.534359486 | 0.000459375 | 0.000720214 | -1.639592257 |
| FGFBP1 | 0.590001988 | 6.74647013 | 3.511513727 | 0.000499349 | 0.000780161 | -1.717590913 |
| SERPINA3 | -0.591653274 | 9.247937396 | -3.375518116 | 0.000813073 | 0.001242736 | -2.172071403 |
| CCL20 | 0.606492653 | 7.431705646 | 3.170338589 | 0.001646691 | 0.002448448 | -2.825700761 |
