# Supplementary material for: Exploring the association between rheumatoid arthritis and non-small cell lung cancer risk: a transcriptomic and drug target-based analysis
Source: Hereditas. 2025 Feb 27;162:28. doi: 10.1186/s41065-025-00396-6 (PMC11866852; doi:10.1186/s41065-025-00396-6)
Supplement: Supplementary file 6 — Supplementary Material 6 [file 41065_2025_396_MOESM6_ESM.docx]

**Supplementary Table S2**

**Gene-Drug Interaction Table**

| **search_term** | **match_term** | **match_type** | **gene** | **drug** | **interaction_types** | **sources** | **pmids** |
| --- | --- | --- | --- | --- | --- | --- | --- |
| TEK | TEK | Definite | TEK | CETIRIZINE |  | DTC | 22406116 |
| TEK | TEK | Definite | TEK | LOPERAMIDE |  | DTC | 22406116 |
| TEK | TEK | Definite | TEK | VANDETANIB | inhibitor | ChemblInteractions |  |
| TEK | TEK | Definite | TEK | REGORAFENIB | inhibitor | TALC\|TdgClinicalTrial\|ChemblInteractions\|MyCancerGenomeClinicalTrial |  |
| TEK | TEK | Definite | TEK | CABOZANTINIB |  | ClearityFoundationClinicalTrial |  |
| TEK | TEK | Definite | TEK | AMPICILLIN |  | DTC | 22406116 |
| TLR4 | TLR4 | Definite | TLR4 | PRAVASTATIN |  | PharmGKB | 12742999 |
| TLR4 | TLR4 | Definite | TLR4 | INFLIXIMAB |  | NCI | 12847679 |
| TLR4 | TLR4 | Definite | TLR4 | NELFINAVIR |  | NCI | 15388451 |
| TLR4 | TLR4 | Definite | TLR4 | ALCOHOL |  | NCI | 11431739 |
| TLR4 | TLR4 | Definite | TLR4 | SAQUINAVIR |  | NCI | 15388451 |
| TLR4 | TLR4 | Definite | TLR4 | TACROLIMUS |  | PharmGKB | 24820765 |
| TLR4 | TLR4 | Definite | TLR4 | METHOTREXATE |  | PharmGKB | 20136356 |
| TLR4 | TLR4 | Definite | TLR4 | RITONAVIR |  | NCI | 15388451 |
| CXCL2 | CXCL2 | Definite | CXCL2 | ALTEPLASE |  | NCI | 18199827 |
| CXCL2 | CXCL2 | Definite | CXCL2 | DEFEROXAMINE |  | NCI | 17883261 |
| IL6 | IL6 | Definite | IL6 | SILTUXIMAB | antagonist\|antibody\|inhibitor | MyCancerGenome\|ChemblInteractions\|TTD | 8823310 |
| IL6 | IL6 | Definite | IL6 | ETANERCEPT |  | PharmGKB | 24253594 |
| IL6 | IL6 | Definite | IL6 | IFOSFAMIDE |  | NCI | 9260581 |
| IL6 | IL6 | Definite | IL6 | RIBAVIRIN |  | PharmGKB |  |
| IL6 | IL6 | Definite | IL6 | LEVOFLOXACIN |  | NCI | 12714806 |
| IL6 | IL6 | Definite | IL6 | GEMFIBROZIL |  | NCI | 8941582 |
| IL6 | IL6 | Definite | IL6 | NELFINAVIR |  | NCI | 15388451 |
| IL6 | IL6 | Definite | IL6 | SAQUINAVIR |  | NCI | 15388451 |
| IL6 | IL6 | Definite | IL6 | RITUXIMAB |  | PharmGKB | 26384320 |
| IL6 | IL6 | Definite | IL6 | INFLIXIMAB |  | PharmGKB | 24253594 |
| IL6 | IL6 | Definite | IL6 | CISPLATIN |  | CIViC | 21273582 |
| IL6 | IL6 | Definite | IL6 | FENOFIBRATE |  | PharmGKB | 16607077 |
| IL6 | IL6 | Definite | IL6 | FENTANYL |  | NCI | 9527747 |
| IL6 | IL6 | Definite | IL6 | ADALIMUMAB |  | PharmGKB | 24253594 |
| IL6 | IL6 | Definite | IL6 | INSULIN |  | NCI | 17392554 |
| IL6 | IL6 | Definite | IL6 | LINEZOLID |  | NCI | 14561977 |
| IL6 | IL6 | Definite | IL6 | METRONIDAZOLE |  | NCI | 12111578 |
| CXCL12 | CXCL12 | Definite | CXCL12 | CYCLOPHOSPHAMIDE |  | PharmGKB | 27173875 |
| CXCL12 | CXCL12 | Definite | CXCL12 | CHLORAMBUCIL |  | PharmGKB | 27173875 |
| CXCL12 | CXCL12 | Definite | CXCL12 | VINCRISTINE |  | PharmGKB | 27173875 |
| CXCL12 | CXCL12 | Definite | CXCL12 | ALEMTUZUMAB |  | PharmGKB | 27173875 |
| CXCL12 | CXCL12 | Definite | CXCL12 | RITUXIMAB |  | PharmGKB | 27173875 |
| CXCL12 | CXCL12 | Definite | CXCL12 | PREDNISONE |  | PharmGKB | 27173875 |
| MMP1 | MMP1 | Definite | MMP1 | RIBAVIRIN |  | NCI | 16699498 |
| MMP1 | MMP1 | Definite | MMP1 | COLLAGENASE CLOSTRIDIUM HISTOLYTICUM |  | TdgClinicalTrial |  |
| MMP1 | MMP1 | Definite | MMP1 | MEDROXYPROGESTERONE ACETATE |  | NCI | 9436888 |
| MMP1 | MMP1 | Definite | MMP1 | LAMIVUDINE |  | NCI | 15309715 |
| MMP1 | MMP1 | Definite | MMP1 | PENTOSAN POLYSULFATE SODIUM |  | NCI | 1384503 |
| MMP1 | MMP1 | Definite | MMP1 | LEFLUNOMIDE |  | NCI | 16762150 |
| MMP1 | MMP1 | Definite | MMP1 | TRIAMCINOLONE |  | NCI | 12123742 |
| MMP1 | MMP1 | Definite | MMP1 | DOXYCYCLINE CALCIUM | inhibitor | ChemblInteractions |  |
| MMP1 | MMP1 | Definite | MMP1 | DOXYCYCLINE | inhibitor | ChemblInteractions |  |
| MMP1 | MMP1 | Definite | MMP1 | SIROLIMUS |  | NCI | 16914544 |
| MMP1 | MMP1 | Definite | MMP1 | LEUPROLIDE ACETATE |  | NCI | 9433928 |
| MMP1 | MMP1 | Definite | MMP1 | HYDROCORTISONE |  | NCI | 7592884 |
| FOS | FOS | Definite | FOS | ALCOHOL |  | NCI | 16750178 |
| FOS | FOS | Definite | FOS | PACLITAXEL |  | NCI | 9588740 |
| FOS | FOS | Definite | FOS | BROMOCRIPTINE |  | NCI | 11680511 |
| FOS | FOS | Definite | FOS | PHENOBARBITAL |  | NCI | 9187311 |
| FOS | FOS | Definite | FOS | PILOCARPINE |  | NCI | 9661997 |
| FOS | FOS | Definite | FOS | NIMODIPINE |  | NCI | 8301097 |
| FOS | FOS | Definite | FOS | THROMBIN |  | NCI | 2119237 |
| FOS | FOS | Definite | FOS | BACLOFEN |  | NCI | 11301212 |
| JUN | JUN | Definite | JUN | QUINAPRIL HYDROCHLORIDE |  | DTC |  |
| JUN | JUN | Definite | JUN | CLOTRIMAZOLE |  | DTC | 16680159 |
| JUN | JUN | Definite | JUN | VINORELBINE TARTRATE |  | DTC |  |
| JUN | JUN | Definite | JUN | ATOMOXETINE HYDROCHLORIDE |  | DTC |  |
| JUN | JUN | Definite | JUN | CLOFIBRATE |  | DTC | 16680159 |
| JUN | JUN | Definite | JUN | FENOFIBRATE |  | DTC | 16680159 |
| JUN | JUN | Definite | JUN | TROPISETRON |  | DTC |  |
| JUN | JUN | Definite | JUN | GEMFIBROZIL |  | DTC | 16680159 |
| JUN | JUN | Definite | JUN | CIPROFIBRATE |  | DTC | 16680159 |
| JUN | JUN | Definite | JUN | CINNARIZINE |  | DTC | 16680159 |
| JUN | JUN | Definite | JUN | AZELASTINE HYDROCHLORIDE |  | DTC |  |
| JUN | JUN | Definite | JUN | DIPHENHYDRAMINE HYDROCHLORIDE |  | DTC |  |
| JUN | JUN | Definite | JUN | VINBLASTINE SULFATE |  | DTC |  |
| JUN | JUN | Definite | JUN | METHIMAZOLE |  | DTC |  |
| JUN | JUN | Definite | JUN | MECHLORETHAMINE HYDROCHLORIDE |  | DTC |  |
| JUN | JUN | Definite | JUN | CUPRIC CHLORIDE |  | DTC |  |
| JUN | JUN | Definite | JUN | SERTRALINE |  | DTC | 16680159 |
| JUN | JUN | Definite | JUN | BUPROPION HYDROCHLORIDE |  | DTC |  |
| JUN | JUN | Definite | JUN | COLCHICINE |  | DTC |  |
| JUN | JUN | Definite | JUN | TRIFLUPROMAZINE HYDROCHLORIDE |  | DTC |  |
| IL1B | IL1B | Definite | IL1B | ASPIRIN |  | PharmGKB | 19448967 |
| IL1B | IL1B | Definite | IL1B | INFLIXIMAB |  | PharmGKB | 22960943 |
| IL1B | IL1B | Definite | IL1B | MORPHINE |  | PharmGKB | 27649267 |
| IL1B | IL1B | Definite | IL1B | ALTEPLASE |  | NCI | 8615653 |
| IL1B | IL1B | Definite | IL1B | LANSOPRAZOLE |  | NCI\|PharmGKB | 16815316\|21054464\|14638340 |
| IL1B | IL1B | Definite | IL1B | OMEPRAZOLE |  | PharmGKB | 16815316\|21054464\|14638340 |
| IL1B | IL1B | Definite | IL1B | HYDROQUINONE |  | NCI | 7589278 |
| IL1B | IL1B | Definite | IL1B | NICARDIPINE |  | NCI | 1888883 |
| IL1B | IL1B | Definite | IL1B | OFLOXACIN |  | NCI | 3260587 |
| IL1B | IL1B | Definite | IL1B | MELATONIN |  | NCI | 8077674 |
| IL1B | IL1B | Definite | IL1B | PENTAMIDINE |  | NCI | 8370344 |
| IL1B | IL1B | Definite | IL1B | DIACEREIN |  | TdgClinicalTrial\|TTD |  |
| IL1B | IL1B | Definite | IL1B | RALOXIFENE |  | NCI | 12773123 |
| IL1B | IL1B | Definite | IL1B | TILUDRONIC ACID |  | PharmGKB | 16257277 |
| IL1B | IL1B | Definite | IL1B | PENTOXIFYLLINE |  | NCI | 8048000 |
| IL1B | IL1B | Definite | IL1B | RABEPRAZOLE |  | PharmGKB | 16815316\|21054464\|14638340 |
| IL1B | IL1B | Definite | IL1B | VERAPAMIL |  | NCI | 2686646 |
| IL1B | IL1B | Definite | IL1B | RILONACEPT | binder\|inhibitor | ChemblInteractions\|TTD | 23319019\|23553601 |
| IL1B | IL1B | Definite | IL1B | ERYTHROMYCIN |  | NCI | 2534682 |
| IL1B | IL1B | Definite | IL1B | RISEDRONIC ACID |  | PharmGKB | 16257277 |
| IL1B | IL1B | Definite | IL1B | THYROGLOBULIN |  | NCI | 2788696 |
| IL1B | IL1B | Definite | IL1B | GLUCOSAMINE |  | TTD |  |
| IL1B | IL1B | Definite | IL1B | CANAKINUMAB | inhibitor\|binder\|antibody | MyCancerGenome\|TdgClinicalTrial\|ChemblInteractions\|TEND\|TTD | 19169963 |
| IL1B | IL1B | Definite | IL1B | PRAVASTATIN |  | PharmGKB | 14515062 |
| IL1B | IL1B | Definite | IL1B | ACITRETIN |  | NCI | 2954576\|1431212 |
| IL1B | IL1B | Definite | IL1B | USTEKINUMAB |  | PharmGKB | 28696418 |
| IL1B | IL1B | Definite | IL1B | HYDROCORTISONE |  | NCI | 2162889 |
| IL1B | IL1B | Definite | IL1B | CEFACLOR |  | NCI | 3260587 |
| ACP5 | ACP5 | Definite | ACP5 | FILGRASTIM |  | NCI | 1724107 |
| ACP5 | ACP5 | Definite | ACP5 | STREPTOZOCIN |  | NCI | 14640894 |
| ACP5 | ACP5 | Definite | ACP5 | ALTEPLASE |  | NCI | 1436243 |
| TNF | TNF | Definite | TNF | CARBAMAZEPINE |  | NCI\|PharmGKB | 15565432\|11294926 |
| TNF | TNF | Definite | TNF | CEFOTAXIME |  | NCI | 8354907\|10989981 |
| TNF | TNF | Definite | TNF | PYRIDOXINE |  | NCI | 16277693 |
| TNF | TNF | Definite | TNF | AMPHOTERICIN B |  | DTC |  |
| TNF | TNF | Definite | TNF | MILTEFOSINE |  | NCI | 7883777 |
| TNF | TNF | Definite | TNF | RIFAMPIN |  | PharmGKB | 22151084 |
| TNF | TNF | Definite | TNF | PROPYLTHIOURACIL |  | NCI | 15119959 |
| TNF | TNF | Definite | TNF | METHIMAZOLE |  | NCI | 8491516 |
| TNF | TNF | Definite | TNF | HYDROXYCHLOROQUINE |  | NCI | 9002011 |
| TNF | TNF | Definite | TNF | ETHAMBUTOL |  | PharmGKB | 22151084 |
| TNF | TNF | Definite | TNF | MEROPENEM |  | NCI | 8354907 |
| TNF | TNF | Definite | TNF | DIGOXIN |  | DTC |  |
| TNF | TNF | Definite | TNF | GEMCITABINE |  | PharmGKB | 31616045 |
| TNF | TNF | Definite | TNF | ADALIMUMAB | antibody\|inhibitor | TdgClinicalTrial\|ChemblInteractions\|TEND\|PharmGKB\|TTD | 12044041\|16720636\|16909270\|23057546\|15022409\|26244882\|12847678\|18050183\|18713756\|19365401\|22760475\|24192118\|14532145\|17343250\|22960943\|15046527\|22129793\|11752352\|12190096\|18438841\|15200343 |
| TNF | TNF | Definite | TNF | INSULIN |  | NCI | 16125526\|9287059 |
| TNF | TNF | Definite | TNF | CYCLOSPORINE |  | PharmGKB | 18444945 |
| TNF | TNF | Definite | TNF | ALTEPLASE |  | NCI | 8615653 |
| TNF | TNF | Definite | TNF | PENTOXIFYLLINE |  | TTD |  |
| TNF | TNF | Definite | TNF | THALIDOMIDE | inhibitor | TdgClinicalTrial\|TEND\|TTD | 8755512\|12046682\|12167383\|12105857\|12102294\|11752352\|12113124 |
| TNF | TNF | Definite | TNF | LACTULOSE |  | NCI | 11226652 |
| TNF | TNF | Definite | TNF | RABEPRAZOLE |  | NCI | 16815316 |
| TNF | TNF | Definite | TNF | LENALIDOMIDE |  | ClearityFoundationClinicalTrial\|TTD |  |
| TNF | TNF | Definite | TNF | STAVUDINE |  | PharmGKB | 20887379 |
| TNF | TNF | Definite | TNF | DIDANOSINE |  | NCI | 9430255 |
| TNF | TNF | Definite | TNF | SORAFENIB |  | PharmGKB | 22736425 |
| TNF | TNF | Definite | TNF | GLIMEPIRIDE |  | NCI | 14686960 |
| TNF | TNF | Definite | TNF | MIDAZOLAM |  | NCI | 16406030 |
| TNF | TNF | Definite | TNF | BUPIVACAINE |  | NCI | 15781526 |
| TNF | TNF | Definite | TNF | GOLIMUMAB | inhibitor\|antibody | TdgClinicalTrial\|ChemblInteractions\|TEND\|TTD | 21079302 |
| TNF | TNF | Definite | TNF | GENTAMICIN |  | NCI | 14565862 |
| TNF | TNF | Definite | TNF | SPIRONOLACTONE |  | NCI | 16837769 |
| TNF | TNF | Definite | TNF | ETANERCEPT | inhibitor\|antibody | TdgClinicalTrial\|ChemblInteractions\|TEND\|PharmGKB\|TTD | 16720636\|16909270\|23057546\|26244882\|12847678\|18050183\|18713756\|19365401\|22760475\|24192118\|17343250\|22960943\|10375846\|10405518\|10206649\|22129793\|11752352\|12190096\|10338381\|18438841\|10357816 |
| TNF | TNF | Definite | TNF | ISONIAZID |  | PharmGKB | 22151084 |
| TNF | TNF | Definite | TNF | CERTOLIZUMAB PEGOL | inhibitor | ChemblInteractions |  |
| TNF | TNF | Definite | TNF | METHYLENE BLUE |  | DTC |  |
| TNF | TNF | Definite | TNF | ATORVASTATIN |  | PharmGKB | 18997459 |
| TNF | TNF | Definite | TNF | INFLIXIMAB | inhibitor | TdgClinicalTrial\|ChemblInteractions\|TEND\|PharmGKB\|TTD | 16720636\|16909270\|23057546\|16456024\|26244882\|12847678\|18050183\|18713756\|19365401\|22760475\|16052578\|24192118\|12110154\|17343250\|22960943\|15481318\|15691299\|15674127\|15804598\|22129793\|11752352\|25311255\|12190096\|18438841\|15691217\|17673491\|15695296\|17642244\|16622728 |
| TNF | TNF | Definite | TNF | CARBOPLATIN |  | PharmGKB | 31616045 |
| TNF | TNF | Definite | TNF | OMEPRAZOLE |  | NCI | 16815316 |
| TNF | TNF | Definite | TNF | RISPERIDONE |  | NCI | 15567770\|11545247 |
| TNF | TNF | Definite | TNF | PYRAZINAMIDE |  | PharmGKB | 22151084 |
| ITGAL | ITGAL | Definite | ITGAL | FLUOROURACIL |  | NCI | 8888499 |
| ITGAL | ITGAL | Definite | ITGAL | EPOETIN ALFA |  | NCI | 9310190 |
| ITGAL | ITGAL | Definite | ITGAL | ETOPOSIDE |  | NCI | 9389690 |
| ITGAL | ITGAL | Definite | ITGAL | THROMBIN |  | NCI | 8598231 |
| ITGAL | ITGAL | Definite | ITGAL | MYCOPHENOLATE MOFETIL |  | NCI | 14550820 |
| ITGAL | ITGAL | Definite | ITGAL | CYCLOSPORINE |  | NCI | 14550820\|10515383 |
| ITGAL | ITGAL | Definite | ITGAL | SIROLIMUS |  | NCI | 10515383 |
| ITGAL | ITGAL | Definite | ITGAL | CYCLOPHOSPHAMIDE |  | NCI | 9389690 |
| ITGAL | ITGAL | Definite | ITGAL | BUSULFAN |  | NCI | 9389690 |
| ITGAL | ITGAL | Definite | ITGAL | LIFITEGRAST | antagonist | ChemblInteractions\|TTD | 27883115 |
| IL1A | IL1A | Definite | IL1A | OLANZAPINE |  | PharmGKB | 21107309 |
| IL1A | IL1A | Definite | IL1A | RILONACEPT | binder | TdgClinicalTrial\|TEND | 23319019\|23553601 |
| IL1A | IL1A | Definite | IL1A | HYDROXYCHLOROQUINE |  | NCI | 8336306 |
| IL23A | IL23A | Definite | IL23A | GUSELKUMAB | blocker\|inhibitor | ChemblInteractions\|TTD | 26714681 |
| IL23A | IL23A | Definite | IL23A | USTEKINUMAB | inhibitor | TdgClinicalTrial\|ChemblInteractions\|TEND\|PharmGKB\|TTD\|FDA |  |
| IL23A | IL23A | Definite | IL23A | TILDRAKIZUMAB | inhibitor | ChemblInteractions\|TTD |  |
| IL23A | IL23A | Definite | IL23A | RISANKIZUMAB |  | TTD |  |
| HLA-DPB1 | HLA-DPB1 | Definite | HLA-DPB1 | ASPIRIN |  | PharmGKB | 15007363\|15784113\|19392989\|9179433 |
| HLA-DPB1 | HLA-DPB1 | Definite | HLA-DPB1 | CLOZAPINE |  | PharmGKB | 11266078 |
| HLA-DRA | HLA-DRA | Definite | HLA-DRA | NIVOLUMAB |  | CIViC | 26822383 |
| HLA-DRA | HLA-DRA | Definite | HLA-DRA | CLAVULANIC ACID |  | PharmGKB | 30664875 |
| HLA-DRA | HLA-DRA | Definite | HLA-DRA | ATEZOLIZUMAB |  | CIViC | 26822383 |
| HLA-DRA | HLA-DRA | Definite | HLA-DRA | PEMBROLIZUMAB |  | CIViC | 26822383 |
| HLA-DRA | HLA-DRA | Definite | HLA-DRA | AMOXICILLIN |  | PharmGKB | 30664875 |
| HLA-DRA | HLA-DRA | Definite | HLA-DRA | FLOXACILLIN |  | PharmGKB | 30664875 |
| CD86 | CD86 | Definite | CD86 | PAMIDRONIC ACID |  | NCI | 15611247 |
| CD86 | CD86 | Definite | CD86 | BELATACEPT | antagonist\|inhibitor | TdgClinicalTrial\|ChemblInteractions | 18269922 |
| CD86 | CD86 | Definite | CD86 | INDOMETHACIN |  | NCI | 9070321 |
| CD86 | CD86 | Definite | CD86 | ROXITHROMYCIN |  | NCI | 10877454 |
| CD86 | CD86 | Definite | CD86 | ABATACEPT | antagonist\|inhibitor | TdgClinicalTrial\|ChemblInteractions\|TEND\|TTD | 18041889\|17020493\|16971318\|16932686\|17212998\|11752352\|16357751\|16573350\|20080922 |
| CD86 | CD86 | Definite | CD86 | DEXAMETHASONE |  | NCI | 15718366 |
| CCL2 | CCL2 | Definite | CCL2 | RISPERIDONE |  | PharmGKB | 24495780 |
| MMP3 | MMP3 | Definite | MMP3 | PRAVASTATIN |  | PharmGKB | 10190398 |
| MMP3 | MMP3 | Definite | MMP3 | LISINOPRIL |  | PharmGKB |  |
| MMP3 | MMP3 | Definite | MMP3 | CHLORTHALIDONE |  | PharmGKB |  |
| HLA-DQA1 | HLA-DQA1 | Definite | HLA-DQA1 | AZATHIOPRINE |  | PharmGKB | 25217962 |
| HLA-DQA1 | HLA-DQA1 | Definite | HLA-DQA1 | MERCAPTOPURINE |  | PharmGKB | 25217962 |
| HLA-DQA1 | HLA-DQA1 | Definite | HLA-DQA1 | LAPATINIB |  | PharmGKB\|FDA | 24687830\|21245432 |
| HLA-DQB1 | HLA-DQB1 | Definite | HLA-DQB1 | TICLOPIDINE |  | PharmGKB | 17339877 |
| HLA-DQB1 | HLA-DQB1 | Definite | HLA-DQB1 | CLAVULANIC ACID |  | PharmGKB | 10535882\|30664875 |
| HLA-DQB1 | HLA-DQB1 | Definite | HLA-DQB1 | NEVIRAPINE |  | PharmGKB |  |
| HLA-DQB1 | HLA-DQB1 | Definite | HLA-DQB1 | AMOXICILLIN |  | PharmGKB | 10535882\|30664875 |
| HLA-DQB1 | HLA-DQB1 | Definite | HLA-DQB1 | LAMOTRIGINE |  | PharmGKB | 19668019 |
| HLA-DQB1 | HLA-DQB1 | Definite | HLA-DQB1 | ASPIRIN |  | PharmGKB | 26366802\|15784113\|19392989 |
| HLA-DQB1 | HLA-DQB1 | Definite | HLA-DQB1 | FLOXACILLIN |  | PharmGKB | 30664875 |
| HLA-DQB1 | HLA-DQB1 | Definite | HLA-DQB1 | CARBAMAZEPINE |  | PharmGKB | 24399721 |
| HLA-DQB1 | HLA-DQB1 | Definite | HLA-DQB1 | ACETAMINOPHEN |  | PharmGKB | 21545408 |
