# Supplementary material for: Exploring the association between rheumatoid arthritis and non-small cell lung cancer risk: a transcriptomic and drug target-based analysis
Source: Hereditas. 2025 Feb 27;162:28. doi: 10.1186/s41065-025-00396-6 (PMC11866852; doi:10.1186/s41065-025-00396-6)
Supplement: Supplementary file 7 — Supplementary Material 7 [file 41065_2025_396_MOESM7_ESM.docx]

**Supplementary Table S3**

**ceRNA Interaction Network Table**

| miRNA | Gene | lncRNA |
| --- | --- | --- |
| hsa-miR-1200 | HLA-DPB1 | LINC01123 |
| hsa-miR-1200 | HLA-DPB1 | RP11-22M7.2 |
| hsa-miR-1200 | HLA-DPB1 | RP11-627G23.1 |
| hsa-miR-1200 | HLA-DPB1 | CTD-2008P7.3 |
| hsa-miR-1200 | HLA-DPB1 | LINC01043 |
| hsa-miR-1200 | HLA-DPB1 | RP11-1129I3.1 |
| hsa-miR-1200 | HLA-DPB1 | LINC00689 |
| hsa-miR-1226-5p | ITGAL | RP11-655H13.2 |
| hsa-miR-1226-5p | ITGAL | CTB-60B18.18 |
| hsa-miR-1226-5p | ITGAL | RP11-256I23.1 |
| hsa-miR-125b-2-3p | ATP6V1C2 | RP11-333E1.2 |
| hsa-miR-129-5p | HLA-DQA1 | RP11-166B2.5 |
| hsa-miR-129-5p | HLA-DQA1 | AC006548.28 |
| hsa-miR-129-5p | HLA-DQA1 | RP3-508I15.22 |
| hsa-miR-129-5p | HLA-DQA1 | RP1-283E3.8 |
| hsa-miR-129-5p | HLA-DQA1 | LINC00662 |
| hsa-miR-129-5p | HLA-DQA1 | RP4-794I6.4 |
| hsa-miR-129-5p | HLA-DQA1 | RP11-67K19.3 |
| hsa-miR-129-5p | HLA-DQA1 | REV3L-IT1 |
| hsa-miR-129-5p | HLA-DQA1 | RP11-486O12.2 |
| hsa-miR-129-5p | HLA-DQA1 | RP11-69I8.2 |
| hsa-miR-129-5p | HLA-DQA1 | SEPSECS-AS1 |
| hsa-miR-129-5p | HLA-DQA1 | RP5-1125A11.7 |
| hsa-miR-129-5p | HLA-DQA1 | RP11-189E14.3 |
| hsa-miR-129-5p | HLA-DQA1 | RP11-848P1.3 |
| hsa-miR-130a-5p | ANGPT1 | AC068489.1 |
| hsa-miR-130a-5p | ANGPT1 | LINC00664 |
| hsa-miR-130a-5p | ANGPT1 | AC084219.4 |
| hsa-miR-130a-5p | ANGPT1 | LL22NC03-27C5.1 |
| hsa-miR-130a-5p | ANGPT1 | RP11-210M15.1 |
| hsa-miR-141-3p | IL1A | RP11-830F9.6 |
| hsa-miR-188-3p | CXCL12 | COL4A2-AS2 |
| hsa-miR-188-3p | CXCL12 | AC084219.4 |
| hsa-miR-188-3p | CXCL12 | LINC01002 |
| hsa-miR-188-3p | CXCL12 | RP11-431K24.1 |
| hsa-miR-188-3p | CXCL12 | CTB-186H2.3 |
| hsa-miR-299-5p | CD86 | AC011284.3 |
| hsa-miR-323b-5p | IL1A | MUC2 |
| hsa-miR-323b-5p | HLA-DPB1 | MUC2 |
| hsa-miR-335-3p | ANGPT1 | LINC01122 |
| hsa-miR-335-3p | ANGPT1 | RP11-96K19.4 |
| hsa-miR-335-3p | ANGPT1 | RP11-146D12.2 |
| hsa-miR-335-3p | ANGPT1 | RP11-335L23.4 |
| hsa-miR-335-3p | ANGPT1 | CTA-392E5.1 |
| hsa-miR-335-3p | ANGPT1 | SLC8A1-AS1 |
| hsa-miR-335-3p | CCL5 | LINC01122 |
| hsa-miR-335-3p | CCL5 | RP11-96K19.4 |
| hsa-miR-335-3p | CCL5 | RP11-146D12.2 |
| hsa-miR-335-3p | CCL5 | RP11-335L23.4 |
| hsa-miR-335-3p | CCL5 | CTA-392E5.1 |
| hsa-miR-335-3p | CCL5 | SLC8A1-AS1 |
| hsa-miR-371a-5p | TGFB2 | CTD-2281E23.3 |
| hsa-miR-616-3p | CXCL5 | RP1-17K7.1 |
| hsa-miR-650 | ITGAL | RP11-458F8.4 |
| hsa-miR-650 | ITGAL | RP11-304L19.13 |
| hsa-miR-650 | ITGAL | CTD-2283N19.1 |
| hsa-miR-650 | ITGAL | CTA-243E7.4 |
| hsa-miR-650 | ITGAL | RP5-1039K5.19 |
| hsa-miR-650 | ITGAL | RP5-892K4.1 |
| hsa-miR-650 | ITGAL | CTD-2013N17.7 |
| hsa-miR-650 | ITGAL | LINC00265 |
| hsa-miR-650 | ITGAL | RP3-395M20.8 |
| hsa-miR-650 | ITGAL | AIRN |
| hsa-miR-650 | ITGAL | LINC00689 |
| hsa-miR-650 | ITGAL | RP11-138B4.1 |
| hsa-miR-650 | ITGAL | AC074212.5 |
| hsa-miR-650 | ITGAL | RP5-1171I10.5 |
| hsa-miR-650 | ITGAL | RP5-1014D13.2 |
| hsa-miR-650 | ITGAL | LA16c-313D11.12 |
| hsa-miR-650 | ITGAL | RP11-66B24.2 |
| hsa-miR-650 | ITGAL | RP11-378E13.3 |
| hsa-miR-650 | ITGAL | EIF3J-AS1 |
| hsa-miR-7-5p | TGFB2 | RP11-338K13.1 |
| hsa-miR-7-5p | TGFB2 | RP11-394A14.2 |
| hsa-miR-7-5p | TGFB2 | RP11-932O9.4 |
| hsa-miR-7-5p | TGFB2 | CDR1-AS |
| hsa-miR-7-5p | TGFB2 | CTA-243E7.1 |
| hsa-miR-7-5p | TGFB2 | RP11-830F9.6 |
| hsa-miR-7-5p | TGFB2 | AC006019.3 |
| hsa-miR-7-5p | TGFB2 | LINC00662 |
| hsa-miR-7-5p | TGFB2 | FLJ35934 |
| hsa-miR-760 | ITGAL | RP11-394A14.2 |
| hsa-miR-760 | ITGAL | AC006019.3 |
| hsa-miR-875-3p | CXCL5 | RP11-64K12.8 |
| hsa-miR-875-3p | CXCL5 | CDR1-AS |
| hsa-miR-875-3p | CXCL5 | PCBP3-OT1 |
| hsa-miR-875-3p | CXCL5 | FRMPD3-AS1 |
| hsa-miR-888-3p | CXCL12 | FAM95B1 |
| hsa-miR-888-3p | CXCL12 | RP1-27K12.2 |
| hsa-miR-892a | HLA-DPB1 | GAS6-AS1 |
| hsa-miR-892a | HLA-DPB1 | C22orf34 |
